# The Rho GTPase Family Genes in Bivalvia Genomes: Sequence, Evolution and Expression Analysis

**DOI:** 10.1371/journal.pone.0143932

**Published:** 2015-12-03

**Authors:** Xue Li, Ruijia Wang, Xiaogang Xun, Wenqian Jiao, Mengran Zhang, Shuyue Wang, Shi Wang, Lingling Zhang, Xiaoting Huang, Xiaoli Hu, Zhenmin Bao

**Affiliations:** Ministry of Education Key Laboratory of Marine Genetics and Breeding, College of Marine Life Sciences, Ocean University of China, Qingdao, 266003, China; Chinese Academy of Fishery Sciences, CHINA

## Abstract

**Background:**

Rho GTPases are important members of the Ras superfamily, which represents the largest signaling protein family in eukaryotes, and function as key molecular switches in converting and amplifying external signals into cellular responses. Although numerous analyses of *Rho* family genes have been reported, including their functions and evolution, a systematic analysis of this family has not been performed in Mollusca or in Bivalvia, one of the most important classes of Mollusca.

**Results:**

In this study, we systematically identified and characterized a total set (*Rho*, *Rac*, *Mig*, *Cdc42*, *Tc10*, *Rnd*, *RhoU*, *RhoBTB* and *Miro*) of thirty *Rho GTPase* genes in three bivalve species, including nine in the Yesso scallop *Patinopecten yessoensis*, nine in the Zhikong scallop *Chlamys farreri*, and twelve in the Pacific oyster *Crassostrea gigas*. Phylogenetic analysis and interspecies comparison indicated that bivalves might possess the most complete types of *Rho* genes in invertebrates. A multiple RNA-seq dataset was used to investigate the expression profiles of bivalve *Rho* genes, revealing that the examined scallops share more similar *Rho* expression patterns than the oyster, whereas more *Rho* mRNAs are expressed in *C*. *farreri* and *C*. *gigas* than in *P*. *yessoensis*. Additionally, *Rho*, *Rac* and *Cdc42* were found to be duplicated in the oyster but not in the scallops. Among the expanded *Rho* genes of *C*. *giga*s, duplication pairs with high synonymous substitution rates (Ks) displayed greater differences in expression.

**Conclusion:**

A comprehensive analysis of bivalve *Rho GTPase* family genes was performed in scallop and oyster species, and *Rho* genes in bivalves exhibit greater conservation than those in any other invertebrate. This is the first study focusing on a genome-wide characterization of *Rho GTPase* genes in bivalves, and the findings will provide a valuable resource for a better understanding of *Rho* evolution and Rho GTPase function in Bivalvia.

## Introduction

Rho (Ras homolog) GTPases are important small G proteins of the Ras superfamily (including Ras, Rho, Ran, Sar/Arf and Rab), the largest signaling protein superfamily found in all eukaryotes [1, 2]. According to previous studies, Rho GTPases can be categorized into nine subfamilies: Rho, Rac, Cdc42, RhoDF, Rnd, RhoUV, RhoH, RhoBTB and Miro [3–7]. As prominent regulators of signaling pathways, Rho proteins can control several vital cellular processes, including cytoskeletal dynamics, cell cycle progression, and cell transformation [8–10]. These factors are also involved in growth-promoting and anti-apoptotic processes as well as in the regulation of gene expression via the activation of signaling molecules such as serum response factor, nuclear factor-kappa B, stress-activated protein kinases and cyclin D1 [6, 11, 12]. Among these multiple cellular roles, a major function of Rho is translating extracellular stimuli into the maintenance and reorganization of the actin cytoskeleton [8]. By regulating actin polymerization, branching and bundling, Rho GTPases are capable of controlling the remodeling of the actin cytoskeleton into distinct architectural elements [8]. The spatial and temporal expression of *Rho GTPases* regulates the construction of these elements into a key controller of the mechanical processes of cell motility and phagocytosis [9, 10]. In addition to these essential roles in cytoskeleton maintenance, cell movement, cell morphology and endosomal trafficking, Rho GTPases are indispensable to the innate immune response [10]. Regulated expression of these proteins has also been reported with regard to phagosome maturation and formation [13], pathogen clearance [14] and intracellular signaling pathway stimulation among a growing number of species [7, 15].

Similar to other Ras-like proteins, Rho proteins typically consist of a conserved structural backbone of five G-boxes that are involved in GTP-binding and GTPase activity [16], and Rho family members are characterized by the presence of a Rho-specific insert domain located between boxes G4 and G5 that is involved in binding to effectors and regulators [17]. Hence, given the lack of this Rho-specific insert sequence, Miro proteins have been considered in some studies to be a separate family of Ras GTPases, with no effect on the actin cytoskeleton or cell morphology [18, 19]. Additionally, the molecular weight of atypical Rho GTPase proteins, RhoBTBs, is much larger (67–83 kD) than those of conventional Rho proteins (~20 kDa), with one or more additional BTB (Bric-a-brac, Tramtrack, Broad-complex) domains [20]. Mitochondrial Rho (Miro) GTPases harbor two GTPase domains interspersed with two EF-hand motifs [21]. Despite the extensive knowledge on these proteins, the genes and sequence features of Rho proteins have been studied in only a limited number of species, and the universal extent of such features in animals requires further confirmation.

*Rho* genes were first isolated from the marine gastropod *Aplysia californica* in 1985 and were subsequently identified in the human genome [22]. Since then, more than 20 unique *Rho* genes have been found in various species [23]. These *Rho* genes originated from an ancestral *Rac* and were gradually distributed among different subfamilies, of which five (*Rac*, *Rho*, *Cdc42*, *RhoBTB* and *Miro*) are present in bilaterians, with six (*RhoUV*) appearing in ecdysozoans [6]. Two extra *Rho* family members (*Rnd* and *RhoDF*) are found in chordates. Vertebrate-specific *RhoH* was the last member identified, completing the entire *Rho* gene family [1]. In taxa after protochordates, additional *Rho GTPase* genes arose without further expansion of subfamily members as a result of whole-genome duplication, gene duplication and retrotransposition [1, 24]. On the basis of current studies, *RhoDF* and *Rnd* appear to be present only in chordates [1]; however, additional corresponding information from invertebrate species are necessary to support this notion.

Although *Rho* genes were initially identified in invertebrates, the genome-wide understanding of these genes in across taxa is limited. Indeed, only limited studies have been conducted in a few species, including *Caenorhabditis elegans*, *Drosophila melanogaster*, *Litopenaeus vannamei* and *Strongylocentrotus purpuratus* [1], in which the importance of invertebrate *Rho* genes was determined through both *in vivo* and *in vitro* experiments. For instance, transfection of a recombinant plasmid containing the *A*. *californica rho* gene into oyster hemocytes was able to reduce the β-adrenoceptor-induced apoptosis [25]. In *C*. *elegans*, several studies have suggested that Rho GTPases are involved in neuronal migration, axon extension and endocytic recycling [12]. Up-regulation of the *Cdc42* gene was also observed in response to the production of Cd^2+^-reduced reactive oxygen species (ROS), apoptosis and DNA damage in the shrimp *L*. *vannamei* [26]. *Drosophila* Cdc42 is believed to be necessary for dorsal vessel closure and participates in embryonic heart development [27]. As a key factor during the early embryonic development of sea urchins, the Rho-dependent signaling pathway plays important roles in the regulation of serine/threonine Rho-kinase (ROCK) [28]. All of these studies emphasize the biological and functional importance of *Rho* genes; however, a systematic analysis of the complete *Rho* family has not been undertaken in any invertebrate species.

Mollusca is the 2^nd^ largest phylum in the animal kingdom, comprising approximately 200,000 described extant species [29]; bivalves represent one of the most important classes and are well known for their dramatic species diversity, wide geographic distribution, and great economic significance [30]. In this study, we performed an analysis on a complete set of *Rho GTPase*s in the genomes of three bivalve species: the Yesso scallop *Patinopecten yessoensis*; the Zhikong scallop *Chlamys farreri*; and the Pacific oyster *Crassostrea gigas*. Orthologs and paralogs were established through a phylogenetic analysis, and the expression profiles of the genes were analyzed using multiple RNA-seq datasets. To our knowledge, this is the first genome-wide characterization of small GTPases in mollusks, and the results will facilitate a better understanding of *Rho* evolution and the function of Rho GTPases.

## Materials and Methods

### Identification of bivalve *Rho GTPase* genes

The transcriptome and whole-genome sequence databases of *P*. *yessoensis* (SRA027310 and SAMN03654043), *C*. *farreri* (unpublished) and *C*. *gigas* (GSE31012 and AFTI00000000) were searched using the blastp or tblastn algorithm at the National Center for Biotechnology Information database (http://www.ncbi.nlm.nih.gov/blast/) to identify *Rho GTPase* genes in bivalves using invertebrate and vertebrate Rho protein sequences from NCBI (http://www.ncbi.nlm.nih.gov), GJI (http://genome.jgi.doe.gov/) and Ensembl (http://useast.ensembl.org) as queries (S1 Table). The captured candidate cDNA sequences of *Rho* genes in bivalves were then aligned with the genome database using GMAP (http://research-pub.gene.com/gmap/) to obtain their genomic structures. ORF (open reading frame) finder (http://www.ncbi.nlm.nih.gov/gorf/gorf.html) was used to predict amino acid sequences. To further verify the gene identifications, the deduced protein sequences were then analyzed using the blastp algorithm for similarity with known genes. The putative isoelectric (PI) points and molecular weights were computed using the Compute pI/Mw tool (http://web.expasy.org/compute_pi/). The DNA Sequence Polymorphism program DnaSP (http://www.ub.edu/dnasp/) was used to estimate the number of synonymous (Ks) and non-synonymous (Ka) substitutions as well as the Ka/Ks ratio among the *C*. *gigas* expanded duplication genes identified in the downstream analysis.

### Protein alignment and phylogenetic analysis

The identified bivalve Rho GTPase proteins were aligned to previously compiled lists of Rho small GTPases using the Clustal Omega multiple alignment program (http://www.ebi.ac.uk/Tools/msa/clustalo/). Conserved domains and motifs were first identified by simple modular architecture research tool (SMART) (http://smart.embl-heidelberg.de/) prediction and further confirmed though sequence alignment with Rho GTPase proteins. Human Rac1, RhoA and Cdc42, with the complete protein architecture resolved, were used as reference sequences. The secondary structure depiction of human Rac1 was used for protein structure annotation. A multiple alignment of the sequences restricted to the core Rho domains was performed using ENDscript (http://endscript.ibcp.fr/ESPript/cgi-bin/ESPript.cgi). DNAstar (version 4.05) was used for amino acid sequence identity calculations among bivalve Rho proteins. The summarized identities of bivalve Rho proteins are illustrated using a heat map generated by heatmap.2 in R (http://www.r-project.org/). The Maximum-Likelihood (ML) algorithm in the MEGA 6.0 software (http://www.megasoftware.net/) was used to construct phylogenetic trees of the *Rho GTPase* genes, and aligned sequences were bootstrapped 1000 times to derive the confidence value for the phylogenic analysis.

### Genome-wide expression analysis of bivalve *Rho GTPase* genes

The expression profiles of bivalve *Rho* genes were constructed using RNA-seq datasets of *C*. *gigas* (GSE31012), *P*. *yessoensis* (SAMN03654043) and *C*. *farreri* (unpublished). After trimming, high-quality reads from multiple RNA-seq datasets were mapped onto the deduced bivalve *Rho GTPase* genes using a TopHat protocol [31]. The total number of reads matching gene regions were counted for digital expression value calculation as RPKM (reads per kilobase per million mapped reads) using ‘HTSeq-count’ script [32, 33]. The Rho RPKM values from the RNA-seq datasets, including different developmental stages (zygote, blastula, gastrula, trochophore, D-shaped larva, early umbo larva, umbo larva, later umbo larva, spat and juvenile) and adult tissues (adductor muscle, hemolymph, digestive gland, gill, mantle, female gonad and male gonad) (S2 Table), were Log10 transformed and subsequently used to create an expression heat map, with the Euclidean distance as a similarity metric and average linkage as a clustering method [34]. A comparison of gene expression levels between duplicated genes in *C*. *gigas* was performed using *t*-tests (two-sided, paired), and *p* values ≤ 0.05 were considered to be statistically significant. Furthermore, RNA-seq datasets from the digestive gland and gills subjected to different environmental stresses in *C*. *gigas*, including digestive gland from oysters challenged with heavy metals (Zn, Cd, Cu, Hg, Pb and Zn+Cd), gills challenged with heavy metals (Zn, Cd, Cu, Hg, Pb and Zn+Cd), salinity (from 5‰ to 40‰), temperature (from 5°C to 35°C) and exposure to air (up to 11 days), as well as adductor muscles challenged with exposure to air, were analyzed. The differential expression levels of *CgRho*s were analyzed using edgeR packages (http://bioconductor.org/packages/release/bioc/html/edgeR.html) (S3 and S4 Tables). As the RNA-seq experiments analyzed in the present study were performed by other authors, we do not provide the experimental details here; details can be retrieved from the supplementary online material for the paper describing the *C*. *gigas* genome [35]. To confirm the RPKM values for the RNAseq datasets, three *PyRho* genes were randomly selected, and their corresponding expression levels in developmental stages and adult tissues were analyzed using real-time PCR (RT-PCR). The detailed methods and the results of the RT-PCR analysis are provided in S1 File.

## Results

### Identification of bivalve *Rho GTPase* genes

**Fig 1:**
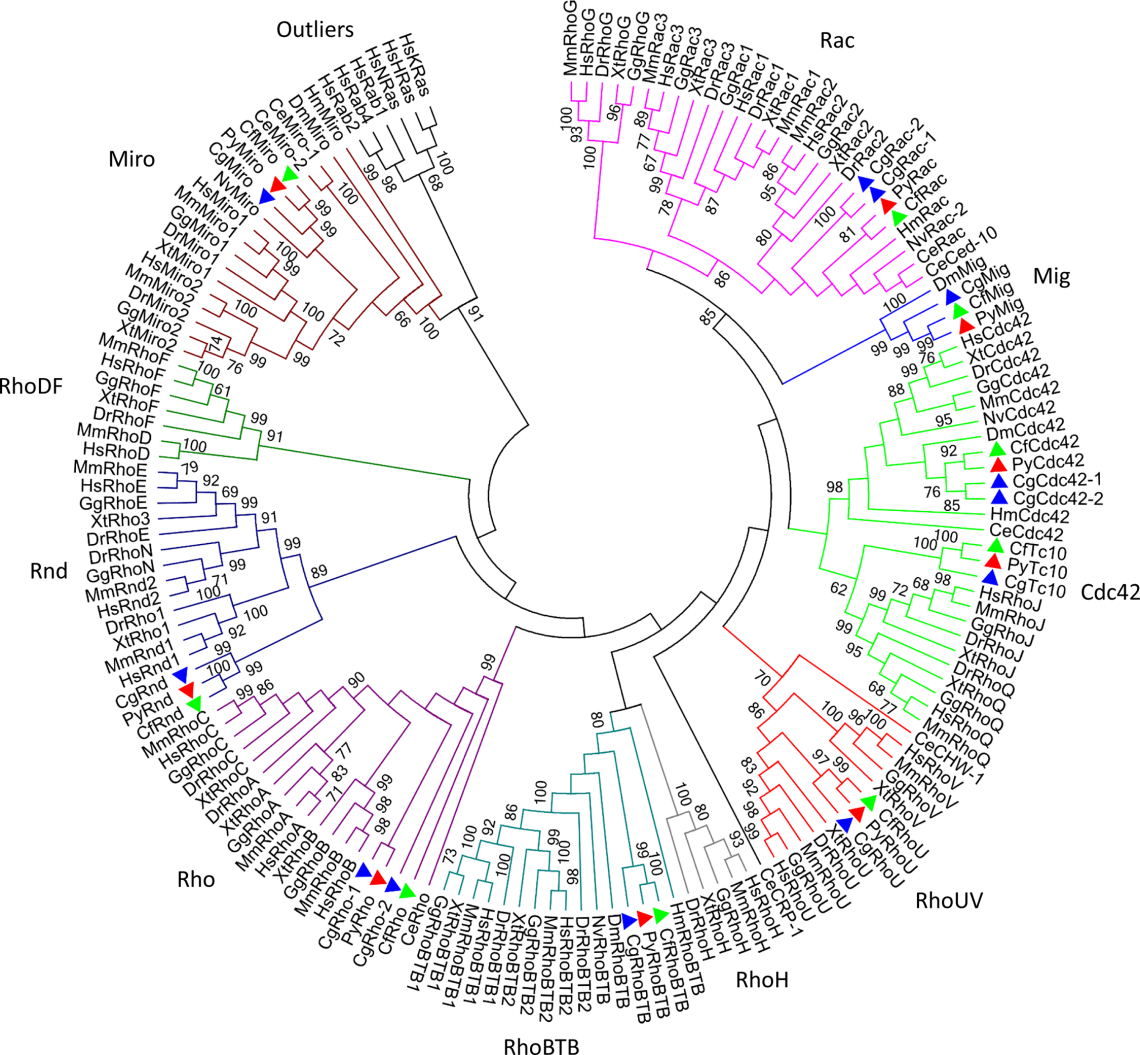
Phylogenetic tree of Rho GTPases. Construction of the phylogenetic tree was based on the amino acid sequences of Rho GTPases from selected species of mammals, amphibians, fish, drosophila, nematodes, cnidarians and mollusks using the Maximum-Likelihood (ML) algorithm in MEGA 6.0. The detailed accession numbers of the protein sequences are presented in S1 Table. The topological stability of the ML tree was evaluated by 1000 bootstrapping replications, and bootstrapping values higher than 60 are indicated by numbers at the nodes. Rho GTPases from *Crassostrea gigas*, *Patinopecten yessoensis*, and *Chlamys farreri* are marked with blue, red and green triangles, respectively.

**Fig 2:**
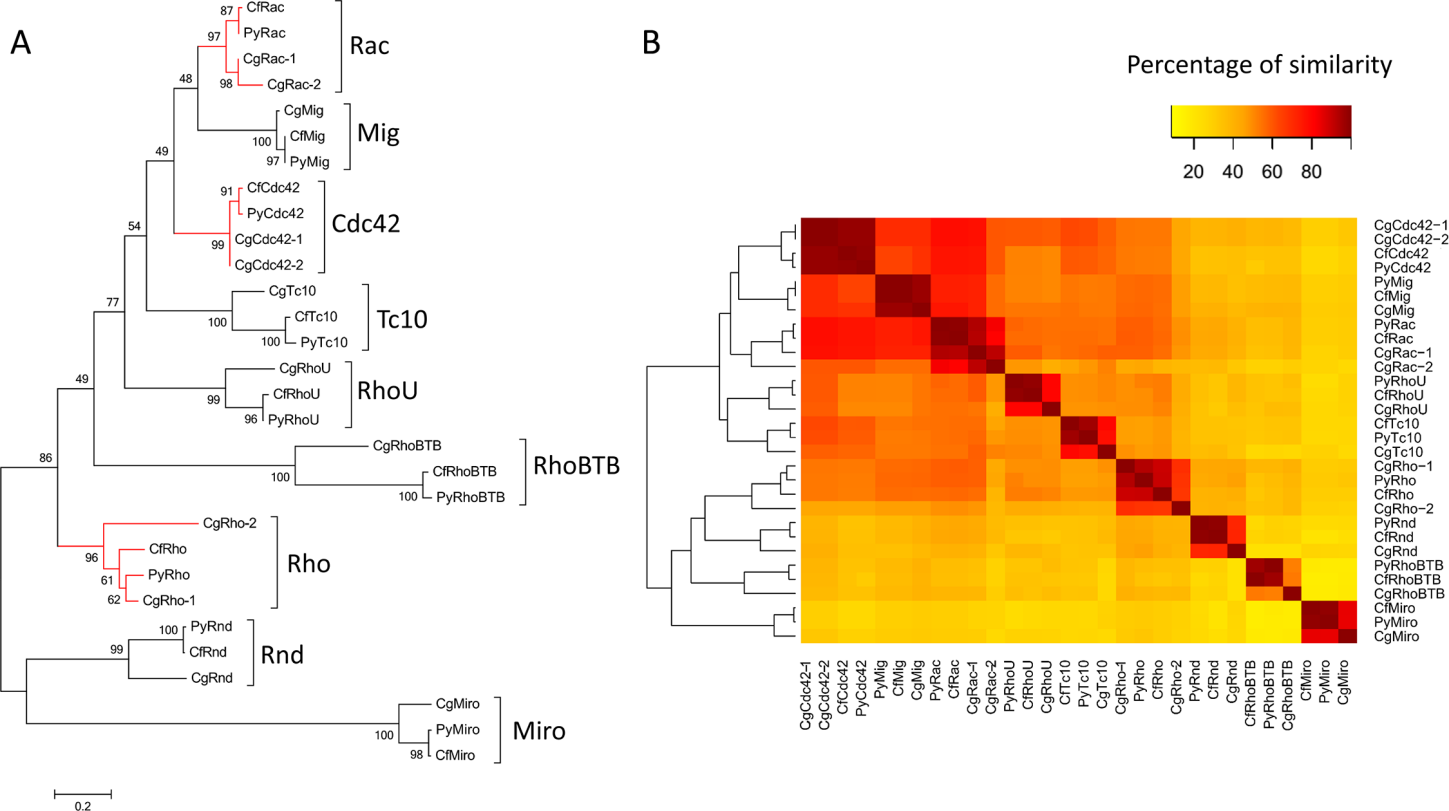
Detailed phylogenetic tree of Bivalvia Rho GTPases and the corresponding amino acid similarity heat map. An unrooted phylogenetic tree (A) was constructed using Rho sequences from *C*. *gigas*, *P*. *yessoensis*, and *C*. *farreri* with the same protocol as in Fig 1. The summarized amino acid similarity of bivalve Rho proteins is presented using a heat map (B) generated by heatmap.2 in R. The detailed accession numbers of the protein sequences are shown in S1 Table. The Rho genes duplicated in bivalves are marked with red lines.

**Fig 3:**
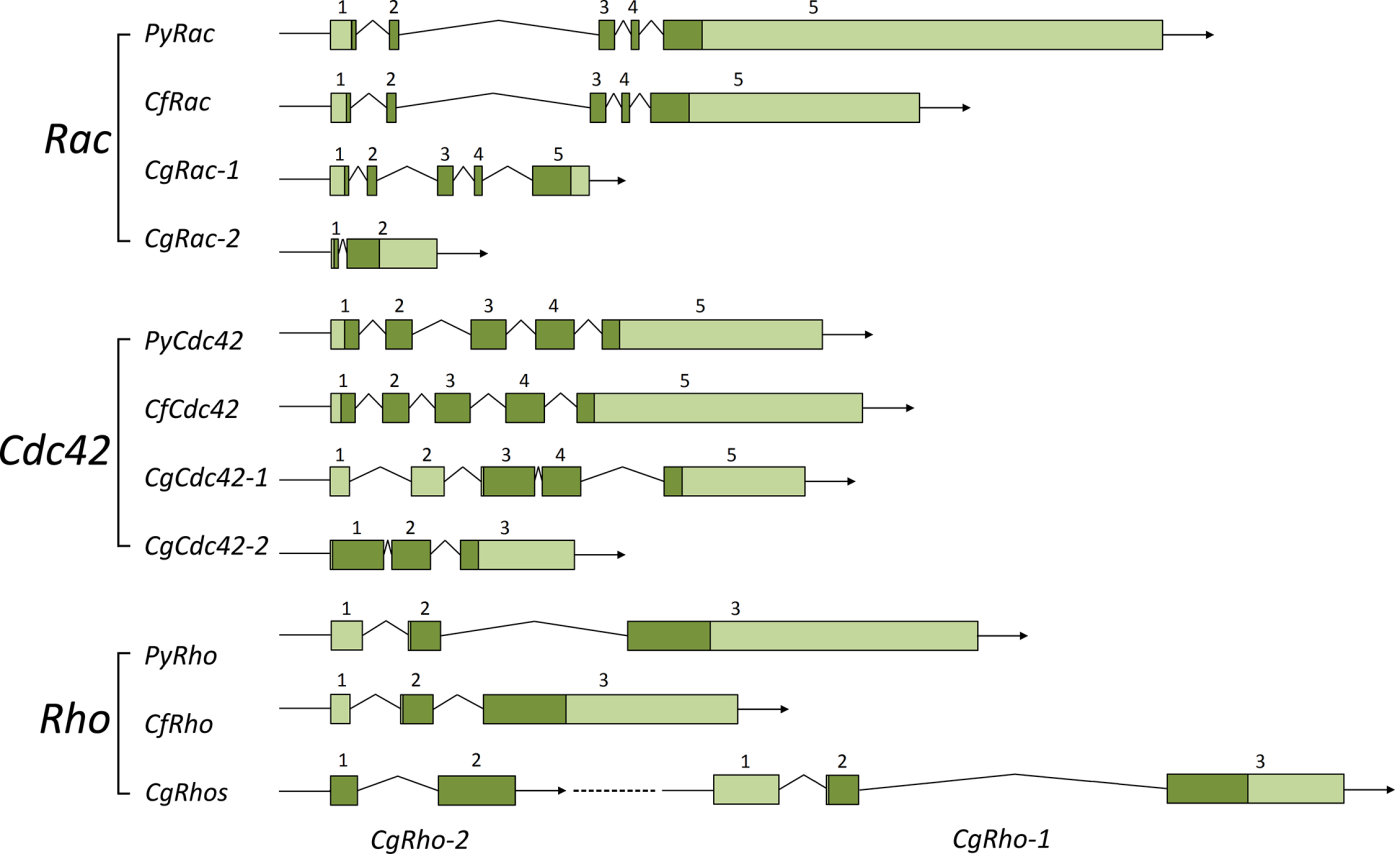
Gene structure of *Rho GTPase* genes. Exons in the ORF (open reading frame) and UTRs are shown separately as dark-green and light-green boxes, and introns are shown as folded lines; the exon numbers are marked. *CgRho*-1 and *CgRho*-2 are repeated in tandem. The detailed accession numbers for the protein sequences are shown in S1 Table.

**Fig 4:**
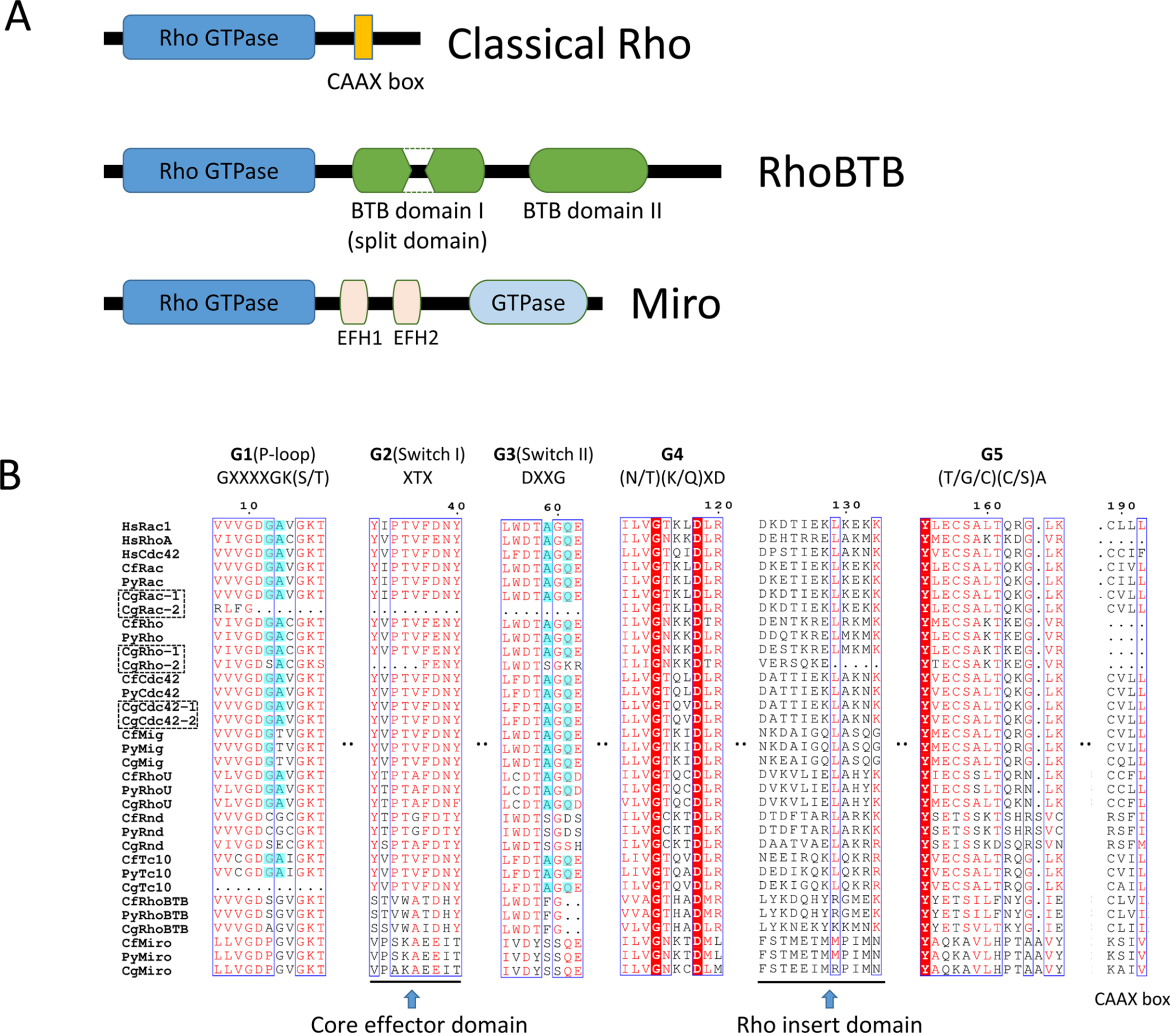
Analysis of the protein sequences and structures of Rho GTPases. (A) Schematic representation of atypical Rho GTPases. Boxes in color are characteristic structures: the Rho GTPase domain (blue), CAAX box (orange), BTB domains (green), EFH domains (pink) and second GTPase domain in Miro (light blue). The first GTPase domain resembles Rho GTPases, and the second is more related to the Rab family of small GTPases [21]. (B) Sequence alignment of the Rho family. The amino acid sequences of Rho GTPases were aligned using ClustalW. The highlighted (cyan) residues are important for GTPase activity. The characteristic structures are marked. The amino acid sequences of RhoBTB and Miro proteins are truncated at the C-terminus. Red shaded and red letters indicate identical and similar amino acids, respectively.

After bioinformatic scanning in both whole-genome and transcriptome databases, nine, nine and twelve *Rho GTPase* genes were identified in the genomes of *P*. *yessoensis*, *C*. *farreri* and *C*. *gigas*, respectively (Fig 1, Fig 2 and S2 File). Members of the *Rho* family, *Rho*, *Rac*, *Cdc42*, *Mig*, *Tc10*, *Rnd*, *RhoU*, *RhoBTB* and *Miro* were found in all three species. The gDNA, cDNA and predicted amino acid sequences of *P*. *yessoensis Rho*s (*PyRho*s) and *C*. *farreri Rho*s (*CfRho*s) genes were submitted to GenBank under accession numbers KT037718 to KT037753. The ORFs of *PyRho* genes are 576 to 2121 bp in length, encoding 192 to 707 amino acids, and those of *CfRho* genes are exactly the same as the corresponding *PyRho* ORFs; *CgRho* ORFs are 330 to 2088 bp in length, encoding 110 to 696 amino acids. The predicted molecular weights of these Rho proteins range from 21.37 to 81.51 kDa, with PI from 5.39 to 9.51 (Table 1). The gene/cDNA, the first introns, which are often related to gene expression [36], ORFs, and 5′ and 3′ UTRs of the bivalve *Rho* members are summarized in Table 1 and Fig 3. In general, the scallop *Rho* genes are longer than the genes in the oyster. The number of exons could be divided into three categories: most of the *Rho* genes contain relatively few exons (2–7), though nine exons can be found in *RhoBTB* genes. In addition, the number of exons in *Miro* genes are 2–3 times (19–20) comparing those of other *Rho* genes. The length of the first intron in different bivalve *Rho* genes varies within a range of 247 to 28,375 bp. Analysis of the genomic structure showed that all of the exon-intron boundaries in the *Rho* genes are consistent with the GT/AG rule for splicing [37]. Domain analysis showed that all bivalve Rho proteins harbor a conserved GTPase domain consisting of five alpha helices (α1-α5), six beta-strands (β1-β6) and five polypeptide loops (G1-G5) (Fig 4, S1 Fig), similar to the Rho proteins in other species. Rho insert domains of various lengths and low conservation, which are regarded as a signature sequence distinguishing Rho proteins from other Ras subfamily members, were also found in the bivalve Rho GTPase proteins (Fig 4B). The CAAX box was observed in bivalve Rho subfamily proteins, including Cdc42, Mig, RhoU, Rho, TC10 and RhoBTB (Fig 4B). In addition to the conserved structures shared by most Rho GTPases, the RhoBTB and Miro proteins possess extra C-terminal extensions [38]. Similarly, BTB domains were identified in bivalve RhoBTBs, and EF-hand (EFH) motifs and additional GTPase domains are present in Miro proteins. Although most of the bivalve Rho GTPase proteins have a structurally complete Rho GTPase domain, incomplete G1-G3 loops were also detected in *C*. *gigas* Rhos (Fig 4B).

**Table 1:** Sequence attributes of *Rho GTPase* genes of Bivalvia.

	Gene length (bp)	cDNA length (bp)	5' UTR (bp)	3' UTR (bp)	ORF length (bp)	First intron length	Exon No.	Intron No.	Protein length	Protein weight (kDa)	PI
*CfRac*	22702	4358	137	3645	576	1280	5	4	192	21.37	8.59
*PyRac*	20100	4342	166	3600	576	1615	5	4	192	21.4	8.59
^**1**^ ***CgRac-1***	**6350**	**896**	**142**	**178**	**576**	**757**	**5**	**4**	**192**	**21.28**	**8.40**
***CgRac-2***	**894**	**647**	**9**	**308**	**330**	**247**	**2**	**1**	**110**	**12.37**	**9.51**
*CfRho*	4290	1606	122	905	579	1321	3	2	193	21.92	7.55
*PyRho*	20748	2796	179	2041	576	1204	3	2	192	21.84	6.62
***CgRho-1***	**9297**	**1434**	**365**	**493**	**576**	**1279**	**3**	**2**	**192**	**21.66**	**5.65**
***CgRho-2***	**-** ^**2**^	**-**	**-**	**-**	**546**	**-**	**-**	**-**	**182**	**20.43**	**8.47**
*CfCdc42*	11814	4504	52	3774	678	698	5	4	226	25.25	8.14
*PyCdc42*	13215	1811	73	1060	678	719	5	4	226	25.35	7.55
***CgCdc42-1***	**6468**	**1530**	**303**	**654**	**573**	**1612**	**5**	**4**	**191**	**21.31**	**6.16**
***CgCdc42-2***	**2078**	**1094**	**15**	**506**	**573**	**205**	**3**	**2**	**191**	**21.31**	**6.16**
*CfRhoU*	23751	5364	227	4402	735	3025	3	2	245	27.15	8.43
*PyRhoU*	27995	5611	469	4407	735	3045	3	2	245	27.06	8.43
*CgRhoU*	9938	1083	226	131	726	2504	3	2	242	26.81	6.31
*CfMig*	26164	1267	143	503	621	14139	6	5	207	22.91	6.99
*PyMig*	45594	1002	153	228	621	28375	6	5	207	22.91	6.99
*CgMig*	9346	1102	171	346	585	3945	6	5	195	21.67	8.13
*CfTc10*	21355	2372	37	1732	603	7624	3	2	201	22.99	8.68
*PyTc10*	19397	3753	121	3029	603	7126	3	2	201	22.98	8.48
*CgTc10*	4247	2439	1400	505	534	992	3	2	178	20.28	8.58
*CfRnd*	44477	2924	361	1846	717	17837	4	3	239	26.26	8.87
*PyRnd*	39133	3509	391	2401	717	17768	5	4	239	26.24	8.87
*CgRnd*	10474	1607	114	815	678	238	6	5	226	25.45	8.85
*CfRhoBTB*	29470	2381	160	100	2121	13447	9	8	707	81.38	5.39
*PyRhoBTB*	27295	3435	330	984	2121	11340	9	8	707	81.51	5.73
*CgRhoBTB*	-	-	-	-	2088	-	-	-	696	79.49	6.26
*CfMiro*	31691	4432	304	2205	1923	2169	20	19	641	73.07	5.73
*PyMiro*	30800	4616	345	2348	1923	2112	21	20	641	72.96	5.9
*CgMiro*	7199	2563	262	438	1863	389	19	18	621	70.55	5.78

^1^Sequence attributes of the duplicated genes in oyster are bolded.

^2^The gene has incomplete information in this regard.

### Phylogenetic analysis and interspecies comparison of Rho proteins

A phylogenetic tree was constructed to determine the identities of the *Rho* genes in the scallops and oyster using known Rho proteins from mammals, amphibians, fish, drosophila, nematodes, cnidarians and mollusks (Fig 1). This phylogenetic analysis indicated that the Rho family of small GTPases can be divided into nine major subfamilies: Rho, Rac, Cdc42, Rnd, RhoDF, RhoUV, RhoH, RhoBTB and Miro. Mig, which is absent in vertebrates, also formed a relatively independent branch (Fig 1). According to the phylogenetic clusters, the corresponding members of these subfamilies were categorized in the scallops and oyster (Fig 2). Rho proteins from bivalve species were first grouped together, and the clades generated were then formed into larger clusters. Such relationships are consistent with the phylogenies of these invertebrates [39]. For instance, *P*. *yessoensis* is phylogenetically closer to *C*. *farreri* than to *C*. *gigas*, as are their *Rho* genes (Fig 2A). It is worth mentioning that Mig proteins are only found in the genomes of invertebrates, including *C*. *elegans*, *D*. *melanogaster* and mollusks (Table 2). On the basis of all the sequence information to date, bivalve Rho proteins can be roughly divided into three groups: a group consisting of Rac, Cdc42 and Mig, which shares high sequence similarity (51.8%-72.3%); a 2^nd^ group (TC10, Rho and RhoU) with similarities that are slightly lower (33.5%-48.2%); and a 3^rd^ group containing Rnd, RhoBTB and Miro, with the lowest sequence similarities (8.9%-22.6%) of the investigated groups (Fig 2B). Comparisons within the bivalve groups showed that both *P*. *yessoensis* and *C*. *farreri* harbor nine *Rho GTPase* genes without duplications, whereas *Rho*, *Rac* and *Cdc42* have expanded only in *C*. *gigas*, with Ka/Ks ratios of 0.15, 2.45 and 0, respectively. Fig 3 shows a schematic of the duplicated gene structures.

**Table 2:** Rho GTPases in different species.

	*H*. *magnipapillata*	*N*. *vectensis*	*S*. *domuncula*	*C*. *elegans*	*D*. *melanogaster*	*C*. *gigas*	*P*. *yessoensis*	*C*. *farreri*	*S*. *purpuratus*	*B*. *floridae*	*C*. *intestinalis*	*D*. *rerio*	*G*. *gallus*	*X*. *tropicalis*	*M*. *musculus*	*H*. *sapiens*
*Rac*	*HmRac*	*NvRac1/NvRac2*	*SdRac*	*CeRac/CeCed-10*	*DmRac1/DmRac2*	***CgRac-1/CgRac-2***	***PyRac***	***CfRac***	*SpRac1/SpRac2/SpRac3/SpRac4*	*BfRac1/BfRac2/BfRac3/BfRac4*	*CiRac1/CiRac2/CiRac3a/CiRac3b/CiRac4*	*DrRac1*	*GgRac1*	*XtRac1*	*MmRac1*	*HsRac1*
												*DrRac2*	*GgRac2*	*XtRac2*	*MmRac2*	*HsRac2*
												*DrRac3*	*GgRac3*	*XtRac3*	*MmRac3*	*HsRac3*
											*CiRhoG*	*DrRhoG*	*GgRhoG*	*XtRhoG*	*MmRhoG*	*HsRhoG*
*Cdc42*	*HmCdc42*	*NvCdc42*	*SdCdc42*	*CeCdc42*	*DmCdc42*	***CgCdc42-1/CgCdc42-2***	***PyCdc42***	***CfCdc42***	*SpCdc42*	*BfCdc42*	*CiCdc42*	*DrCdc42*	*GgCdc42*	*XtCdc42*	*MmCdc42*	*HsCdc42*
						***CgTc10***	***PyTc10***	***CfTc10***	*SpTc10*	*BfTc10-1/BfTc10-2*	*CiTc10*	*DrRhoJ*	*GgRhoJ*	*XtRhoJ*	*MmRhoJ*	*HsRhoJ*
												*DrRhoQ*	*GgRhoQ*	*XtRhoQ*	*MmRhoQ*	*HsRhoQ*
*Rho*	*HmRho1/HmRho2/HmRho3*	*NvRho1/NvRho2*	*SdRho1/SdRho2/SdRho3*	*CeRho*	*DmRho*	***CgRho-1/CgRho-2***	***PyRho***	***CfRho***	*SpRho1/SpRho2*	*BfRho1/BfRho2*	*CiRho*	*DrRhoA*	*GgRhoA*	*XtRhoA*	*MmRhoA*	*HsRhoA*
													*GgRhoB*	*XtRhoB*	*MmRhoB*	*HsRhoB*
												*DrRhoC*	*GgRhoC*	*XtRhoC*	*MmRhoC*	*HsRhoC*
*Rnd*						***CgRnd***	***PyRnd***	***CfRnd***		*BfRnd*		*DrRnd1*	*GgRnd1*	*XtRnd1*	*MmRnd1*	*HsRnd1*
												*DrRnd2*			*MmRnd2*	*HsRnd2*
												*DrRnd3*	*GgRnd3*	*XtRnd3*	*MmRnd3*	*HsRnd3*
*RhoDF*															*MmRhoD*	*HsRhoD*
										*BfRif*	*CiRif*	*DrRhoF*	*GgRhoF*	*XtRhoF*	*MmRhoF*	*HsRhoF*
*RhoUV*					*DmRhoU*	***CgRhoU***	***PyRhoU***	***CfRhoU***	*SpRhoU*	*BfRhoU*		*DrRhoU*	*GgRhoU*	*XtRhoU*	*MmRhoU*	*HsRhoU*
													*GgRhoV*	*XtRhoV*	*MmRhoV*	*HsRhoV*
*Mig*				*CeMig*	*DmMig*	***CgMig***	***PyMig***	***CfMig***								
*RhoH*												*DrRhoH*	*GgRhoH*	*XtRhoH*	*MmRhoH*	*HsRhoH*
*RhoBTB*	*HmRhoBTB*	*NvRhoBTB*			*DmRhoBTB*	***CgRhoBTB***	***PyRhoBTB***	***CfRhoBTB***	*SpRhoBTB*	*BfRhoBTB*	*CiRhoBTB*	*DrRhoBTB1*	*GgRhoBTB1*	*XtRhoBTB1*	*MmRhoBTB1*	*HsRhoBTB1*
												*DrRhoBTB2*	*GgRhoBTB2*	*XtRhoBTB2*	*MmRhoBTB2*	*HsRhoBTB2*
*Miro*	*HmMiro*	*NvMiro*		*CeMiro1/CeMiro2*	*DmMiro*	***CgMiro***	***PyMiro***	***CfMiro***	*SpMiro*	*BfMiro*	*CiMiro*	*DrMiro1*	*GgMiro1*	*XtMiro1*	*MmMiro1*	*HsMiro1*
												*DrMiro2*	*GgMiro2*	*XtMiro2*	*MmMiro2*	*HsMiro2*

Rho GTPases from bivalves are marked in bold.

**Fig 5:**
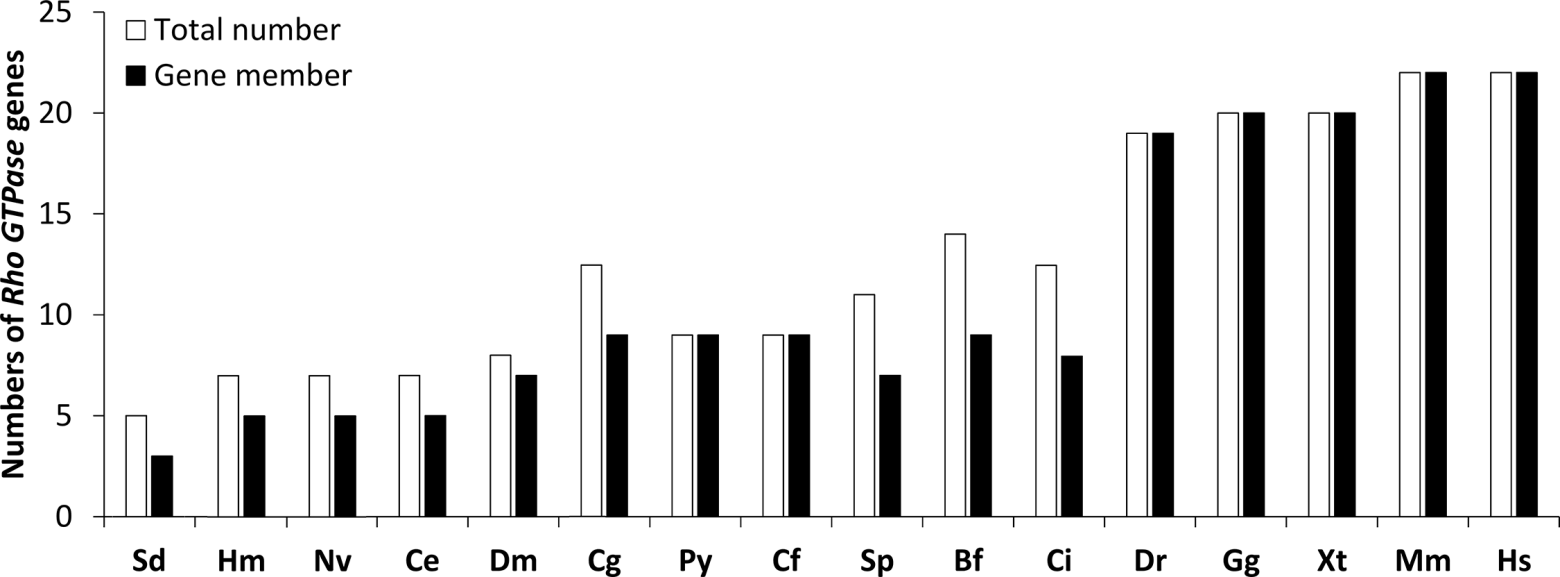
Interspecies comparison of Rho subfamily proteins. Species abbreviations: Hm, *Hydra magnipapillata*; Nv, *Nematostella vectensis*; Sd, *Suberites domuncula*; Ce, *Caenorhabditis elegans*; Dm, *Drosophila melanogaster*; Cg, *C*. *gigas*; Py, *P*. *yessoensis*; Cf, *C*. *farreri*; Sp, *Strongylocentrotus purpuratus*; Bf, *Branchiostoma floridae*; Ci, *Ciona intestinalis*; Dr, *Danio rerio*; Gg, *Gallus gallus*; Xt, *Xenopus tropicalis*; Mm, *Mus musculus*; Hs, *Homo sapiens*. The white bars indicate the total number of *Rho* genes; the black bars indicate *Rho* gene members.

Interspecies comparison analysis indicated that Rho subfamily proteins expanded notably during evolution (Fig 5). As shown in Table 2, *C*. *gigas Rac*, *Cdc42* and *Rho* have been duplicated, similar to *Branchiostoma floridae Rac*, *Rho* and *TC10*, *D*. *melanogaster Rac*, *C*. *elegans Miro* and *Nematostella vectensis Rac*. In general, an increase in both *Rho* family members and total gene numbers can be observed from invertebrates to vertebrates (Fig 5). In invertebrates, the increase in *Rho* gene number was accompanied by the emergence of new gene subfamilies before the appearance of Bivalvia. Following the emergence of Bivalvia, the number of gene subfamilies decreased slightly due to the loss of *Mig* in Echinodermata and all taxa thereafter. In vertebrates, the *Rho* gene number increased dramatically due to the expansion of genes within the original subfamilies, including the *RhoA*-related subfamily (*RhoA*, *RhoB* and *RhoC*), *Rac*1-related subfamily (*Rac*1, *Rac*2, *Rac*3 and *RhoG*), *Cdc42*-related subfamily (*Cdc42*, *RhoJ* and *RhoQ*), *Rnd* subfamily (*Rnd*1, *Rnd*2 and *Rnd*3), *RhoBTB* subfamily (*RhoBTB*1 and *RhoBTB*2) and *Miro* subfamily (*Miro*1 and *Miro*2) (Table 2).

### Spatiotemporal expression of *Rho* genes in bivalves

**Fig 6:**
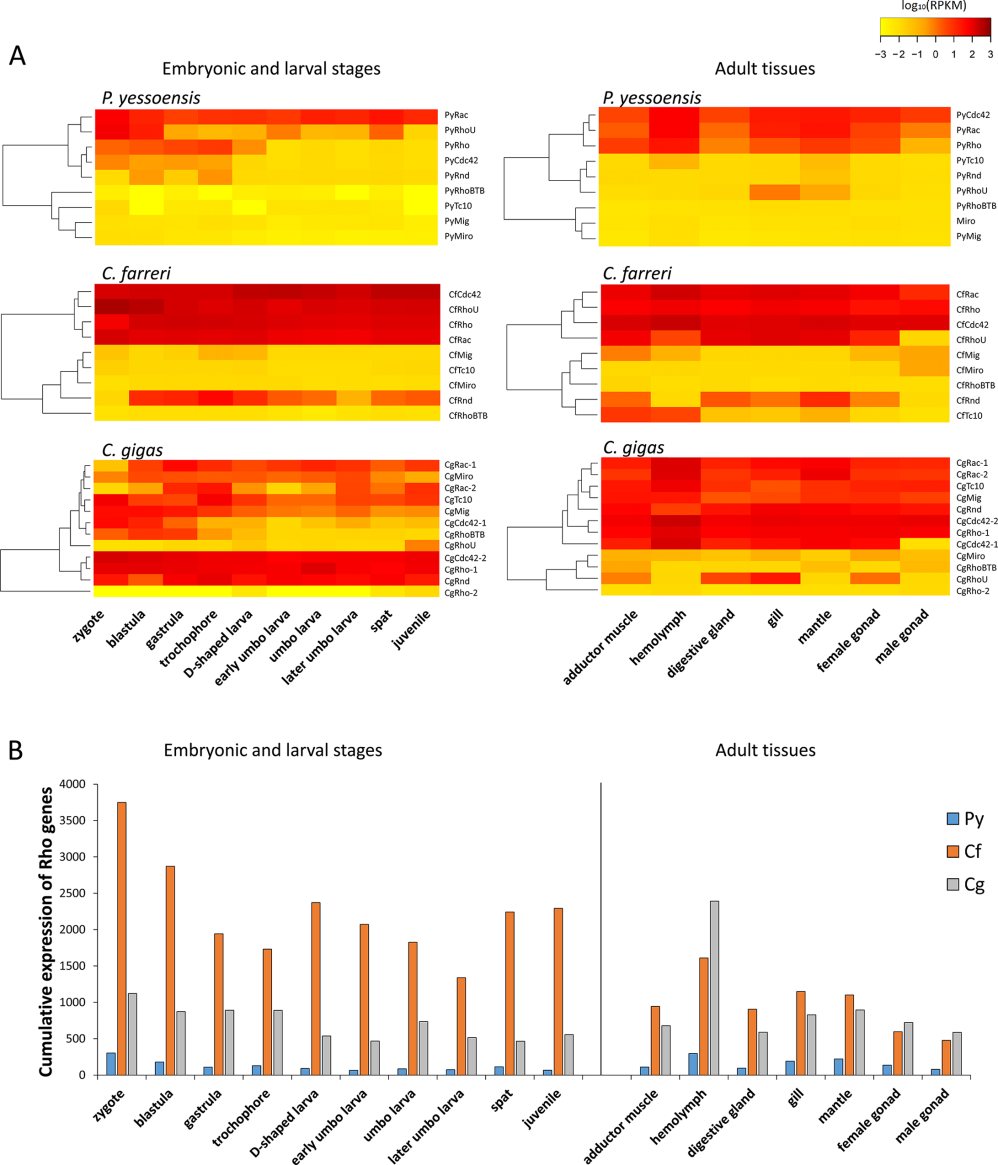
Expression analysis of Rho genes in bivalves. (A) Heat map summarizing the expression of *Rho GTPase* genes during embryonic and larval developmental stages and in different adult tissues. RPKM values were modified by Log10 transformation. (B) Cumulative expression of *Rho GTPase* genes in different development stages and adult tissues of *P*. *yessoensis* (Py), *C*. *farreri* (Cf) and *C*. *gigas* (Cg).

**Fig 7:**
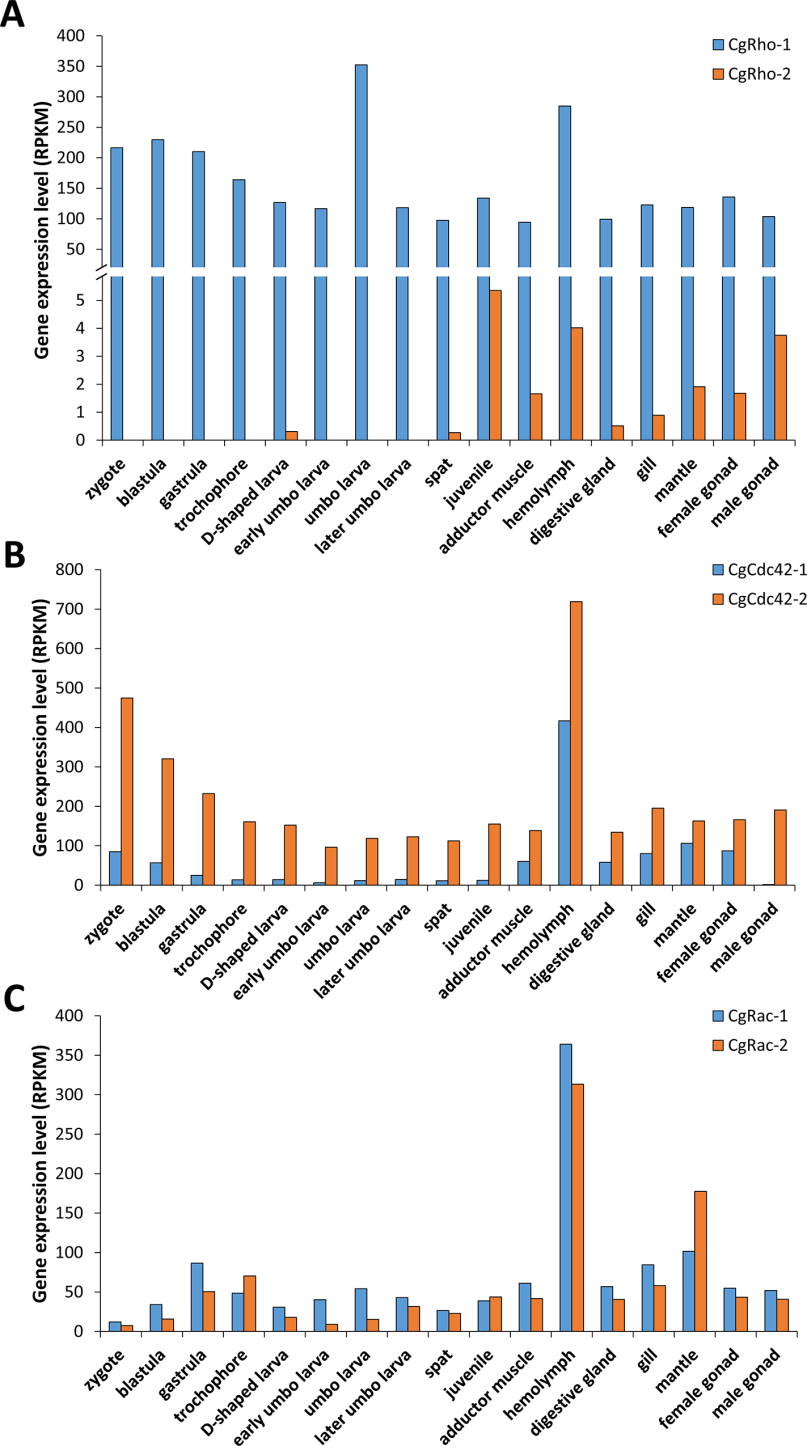
Spatiotemporal expression of duplicated genes in *C*. *gigas*.

RNA-seq datasets for different developmental periods and adult tissues of *C*. *farreri*, *P*. *yessoensis* and *C*. *gigas* were analyzed to detect the expression patterns of bivalve *Rho GTPase* genes (Fig 6, S2 Table). Among the ten developmental stages examined, *C*. *farreri* scallop *Rho* genes could be divided into two groups according their expression levels: a highly ubiquitously expressed group, including *CfRac*, *CfRhoU*, *CfRho*, *CfRnd* and *CfCdc42*, with an average RPKM for all stages > 36; and a rarely expressed group, including *CfMig*, *CfTC10*, *CfRhoBTB* and *CfMiro*, with an average RPKM for all stages < 8. A similar expression pattern was also observed in *P*. *yessoensis*, in which *PyRho*, *PyRac*, *PyRhoU*, *PyCdc42* and *PyRnd* were relatively highly expressed according to both RNA-seq data (with an RPKM ranging from 7.54 to 56.03) and corresponding real-time PCR validations (S1 File, S2 Fig); *PyMig*, *PyTC10*, *PyRhoBTB* and *PyMiro* were negligibly expressed, with an average RPKM < 1 for all stages. In contrast to these scallops, the expression pattern of *Rho* genes in *C*. *gigas* was more complicated. Overall, *CgRho-1*, *CgRnd* and *CgCdc42-2* showed high level and ubiquitous expression, with an average RPKM > 100. The expression of *CgMiro*, *CgMig*, *CgRac-1* and *CgRac-2* was also broad but at lower levels, whereas *CgRho-2* was barely expressed in all of the stages analyzed. The levels of *Rho* gene expression changed during the development of embryos/larvae, with *CgMig*, *CgCdc42*s and *CgRhoU* decreasing and *CgRac-1* and *CgRac-2* increasing in the gastrula and trochophore stages, respectively (Fig 6A and Fig 7).

When comparing the expression of bivalve *Rho* genes among tissues, the hemolymph demonstrated the greatest number of highly expressed *Rho* genes, followed by the digestive gland, gill, mantle and adductor muscle (Fig 6A). In *P*. *yessoensis*, a total of six *Rho* genes were expressed, with an RPKM ≥ 5 in at least one tissue, including *PyCdc42*, *PyRho* and *PyRac*, which were expressed at an RPKM > 50. In *C*. *farreri*, the expression levels of *Rho* genes were higher than in *P*. *yessoensis*; all of the *PyRho* genes were expressed with an RPKM ≥ 5, including *CfCdc42*, *CfRho*, *CfRhoU* and *CfRac* with an RPKM > 100. Again, the expression of *C*. *gigas Rho* genes was rather distinct from those of the scallops. Apart from *CgRhoBTB* and *CgRho-2*, all of the *C*. *gigas Rho* genes were found to be highly expressed, with an RPKM > 20 in at least three tissues. Furthermore, the integral *Rho* expression of *C*. *farreri* and *C*. *gigas* was much greater than that of *P*. *yessoensis* during both developmental stages and in healthy tissues (Fig 6B).

### Expression levels of duplicated *Rho* genes in oyster

Differential expression patterns were further analyzed among *Rho* duplications in *C*. *gigas* (Fig 7). In general, the most significant differences in expression were observed among *CgRho* duplications (two-sided, paired *t*-test, *p* = 1.45E-07), followed by *CgCdc42* (*p* = 3.73E-06) and *CgRac* (*p* = 0.124) duplications. The integrated expression of *C*. *gigas Cdc42-1* was significantly lower than that of Cg*Cdc42-2*, and during developmental, the RPKM of Cg*Cdc42-2* ranged from 96.29 to 474.58, approximately tenfold higher than that of *CgCdc42-1*. Although differences in expression between these two genes was smaller among adult tissues, no *Cdc42-1* transcript was detected in male gonads. *CgRho-1* was ubiquitously and highly expressed, not only during developmental stages but also in adult tissues, whereas Cg*Rho-2* mRNA was only barely detected in juvenile and adult tissues. The expression patterns of *CgRac*s were similar, with slightly more *CgRac-2* than *CgRac-1* transcripts in the mantle and more *CgRac-1* than *CgRac-2* transcripts in other tissues.

### Expressions of *CgRho* genes in response to environmental stresses

To examine the expression patterns of *CgRho*s in response to environmental stresses, RNA-seq datasets from *C*. *gigas* treated with salinity or temperature changes, exposure to air and heavy metals were analyzed. *CgCdc42*-2, *CgRac*-2, *CgRho*-2, *CgRhoU*, *CgRhoBTB* and *CgRnd* were found to be sensitive to at least one of these challenges (S3–S6 Figs). The expression level of *CgRac*-2 gradually increased with a rise in environmental temperature, whereas *CgCdc42-2* tended to be down-regulated after treatments with both heat and cold (S3A Fig). *CgRho-2* was also significantly up-regulated with low-salinity treatments (S3B Fig). The expression of *CgRnd* and *CgRhoU* in the gills was markedly increased when the oysters were exposed to air, and *CgRhoBTB* expression was decreased in the muscle (S4 Fig). In contrast to responses to changes in temperature and salinity as well as hypoxia, *CgRho* genes did not appear to be very sensitive to heavy metal stresses (S5 and S6 Figs).

## Discussion

The Ras superfamily of small GTP-binding proteins is the largest family of signaling proteins in eukaryotic cells [2] and can be divided into five major families according to their corresponding divergence in sequence and function: Ras, Rho, Arf/Sar, Ran, and Rab [40]. Among the entire superfamily, the Rho family is involved in signaling networks that regulate actin, cell cycle progression, and gene expression [4]. Despite their importance, no analysis of Rho families or characterization of *Rho GTPase* genes has been undertaken in Bivalvia. In this study, we identified a complete set of *Rho GTPase* genes in the genomes of the bivalve *P*. *yessoensis*, *C*. *farreri* and *C*. *gigas*. We also analyzed the protein structure, phylogenetic relationships and transcription patterns of these *Rho* genes to provide insight into their gene identities, evolution and expression.

After extensive data mining in all existing RNA-seq assemblies, full-length transcriptome databases and genome assemblies, nine to twelve *Rho* genes were identified in the three bivalves and categorized into nine *Rho* gene subfamilies: *Rho*, *Rac*, *Cdc42*, *Tc10*, *Mig*, *Rnd*, *RhoU*, *RhoBTB* and *Miro* (Fig 1). Among these, the *Rho*, *Rac* and *Cdc42* subfamilies are thought to be the foundation of the *Rho* family [3]. Highly conserved features of *Rho* genes are found with regard to their structure and function throughout eukaryotic evolution [3, 41]. In addition to these three subfamilies, atypical *Rho GTPase* genes, including *Tc10*, *RhoU*, *Rnd*, *Mig*, *RhoBTB* and *Miro*, were also found in mollusks. These proteins are involved in a broad spectrum of biological processes, such as cytoskeletal dynamics, T-cell signaling and protein ubiquitylation [6]. Interestingly, *Mig* is absent in Echinodermata and subsequent taxa. By comparing *Rho* gene family members between bivalves and *S*. *purpuratus*, a notable expansion of the *Rac* gene was found, and according to a protein similarity analysis in bivalves, Mig proteins are closely related to Rac proteins (Fig 2B). Since the cooperation of *Rac* and *Mig* was reported to participate in the control of axon outgrowth and guidance in *Drosophila* and nematodes [42–44], evidence suggests that the newly emerged *Rac* genes in *S*. *purpuratus* might constitute a supplementary strategy to fulfill the *Mig* functions. Overall, except for the subfamily members *RhoDF* and *RhoH*, which were confirmed as first appearing in chordates [1], all existing invertebrate *Rho* genes can be found in Mollusca. The results of interspecies comparison in this study indicate that bivalves might possess the most complete set of *Rho* genes found in invertebrates.

Compared to the examined scallops, in which only one copy of all of the *Rho* genes was found, an extra copy of *Rac*, *Cdc42* and *Rho* was detected in the *C*. *gigas* genome. As shown in Table 2, some *CgRho* genes, such as *CgCdc42*s, were duplicated, suggesting that *Rho* gene expansion in *C*. *gigas* might be another example of oyster gene family expansion compared to other bivalves, a pattern that has also been reported for other oyster gene families [34, 35, 45]. Furthermore, the expression of duplicated genes, including *CgCdc42*-2, *CgRho*-2 and *CgRac*-2, was found to be regulated under different environmental challenges (S3–S6 Figs, S4 Table). Such duplication patterns in *C*. *gigas Rho* genes might be relevant to its environmental suitability. Indeed, oysters are remarkably resilient against harsh environmental conditions, including pathogen infections, fluctuations in temperature and salinity, and prolonged air exposure [45], and numerous expanded genes with high sequence, structural and functional diversity have been reported to be involved in oyster stress responses through complex interactions [34, 35, 45]. In addition, previous studies have shown that *Rho*, *Rac* and *Cdc42* subfamily members play crucial roles in the innate immune response, including the maturation and formation of the phagosome [13], clearance of pathogens [14] and triggering of intracellular signaling pathways [7, 15]. Therefore, the expanded oyster *Rho* genes might be relevant to the oyster’s immune response and adaptation to highly stressful and fluctuating environments. However, our understanding of the precise mechanism of *Rho* duplications in *C*. *gigas* is still fragmentary, and further studies are needed to fully elucidate the functional diversification of bivalve *Rho* genes.

However, duplicated *C*. *gigas Rho* genes with different protein structure than their original ones were also found. Structurally, all Rho GTPases share a characteristic Rho-like GTPase domain [5], which is also a characteristic that distinguishes them from other small GTPases. Most of the bivalve Rho GTPases exhibited all of the characteristic features of Rho GTPases, whereas proteins with incomplete structures were observed among the expanded *C*. *gigas* Rho duplications (Fig 4). In addition to *C*. *gigas* harboring conserved critical residues (Fig 4), incomplete G2 loops (containing three critical residues substitutions) were detected in CgRho-2, and the G1-G3 loops were completely absent in CgRac-2. The G1 loop (also known as the P-loop) is capable of binding to a phosphate group [23], and the G2 (Switch I) and G3 (Switch II) loops contain conserved residues responsible for Mg^2+^ and phosphate binding [23]. These functional loops contain residues important for GTPase activity and the core effector domain [23]. Within this context, it is noteworthy that most of the *Rho* genes duplicated in *C*. *gigas* still encode proteins with incomplete Rho GTPase structures compared to the original type (Fig 4). Variations and peculiarities have also been reported in duplicated genes in other species [46, 47]. Gene duplication is one of the main processes responsible for expanding protein functional diversity, whereas sequence variation, domain shuffling and domain recombination are major scenarios associated with specific changes in protein function [48, 49]. These variants can be benign, have subtle influences on phenotypes or be associated with disease [46]. Accordingly, further analyses are needed to explain the structural incompleteness of the functional domains of *C*. *gigas* Rho proteins, especially the duplicated ones.

In this study, differences in spatiotemporal expression were detected in the duplicated *Rho* genes of *C*. *gigas*. A two-sided, paired *t*-test revealed significant differences in expression of duplicated *CgRho* and *CgCdc42* genes, with *p*-values much smaller than 0.01 (1.45E-07 and 3.73E-06, respectively); however, the overall differences in expression between the two copies of *CgRac*s were not statistically significant (*p* > 0.05). A Ka/Ks rate analysis was performed to further explore the potential selection status of these oyster genes (S5 Table). According to the Ka/Ks rate of the three pairs of duplicated genes, a strong purifying selection pressure can be deduced for *CgCdc42-1/CgCdc42-2* (Ka/Ks = 0) and *CgRho-1/CgRho-2* (Ka/Ks = 0.15). In contrast, a high Ka/Ks ratio (Ka/Ks = 2.45) was observed between the duplication of *CgRac-1/CgRac-2*, which may indicate that the gene has experienced positive selection [50, 51]. In addition to the selection status, the Ks value can also be adopted for an estimation of the generation time of duplicated genes [52–54], with more ancient duplications leading to a higher Ks rate and vice versa and duplications with Ks <1 usually defined as recent duplications [55, 56]. In *C*. *gigas*, the Ks rate of duplicated genes was 0.026 for *CgRac*s, 0.084 for *CgCdc42*s and 1.534 for *CgRho*s, respectively. Newly duplicated *CgRac*-2 was found with the most incomplete exon-intron (with three exons missing) and protein (with three G-boxes missing) structures, which indicated that it might be functionally incomplete or even be under a pseudogenization trend in the oyster genome. Nonetheless, based on its selection status (Ka/Ks = 2.45, strong positive selection) and high expression patterns (RPKM > 50 in at least five tested tissues or developmental stages, Fig 7), the *CgRac*-2 gene might be undergoing or have undergone sub-functionalization or neo-functionalization compared to the original gene, as occurs with other duplicated genes [57, 58]. The Ks patterns appear to be related to the corresponding divergence in expression between *CgRho* duplication pairs. In fact, the duplication pairs with higher Ks ratios displayed greater differences in expression. For instance, *CgRho-2* had the largest Ks (1.534) and the greatest difference in expression (*p*-value = 1.44E-7) than its duplicated gene (Figs 3 and 7), whereas the corresponding expression difference was not significant for the most recent duplications, *CgRac-*1/*CgRac-*2 (Ks = 0.026) (*p*-value = 0.124). Previous studies indicate that expanded genes generated by duplication are less likely to have strongly correlated expression profiles than those that remain in one-to-one relationships among species [59]. In addition, it is believed that expression divergence and coding-sequence divergence both increase with the age of duplicate genes [60]. Such data suggest the existence of a general trend for paralogous genes to become more specialized in their expression patterns since duplication, with decreased breadth and increased specificity of expression [59], consistent with our observations of duplicated *CgRho* genes.

## Conclusions

In summary, a total of thirty *Rho GTPase* genes, encompassing *Rho*, *Rac*, *Mig*, *Cdc42*, *Tc10*, *Rnd*, *RhoU*, *RhoBTB* and *Miro* subfamily members, are herein described in three bivalve species, including nine in *P*. *yessoensis*, nine in *C*. *farreri* and twelve in *C*. *gigas*. Our results showed that bivalve *Rho* genes might represent the most complete set of *Rho* genes in invertebrates. The scallops exhibit *Rho* expression patterns similar to those of *C*. *gigas*, whereas more *Rho* mRNAs were found to be expressed in *C*. *farreri* and *C*. *gigas* than in *P*. *yessoensis*. Gene duplications were found in the *C*. *gigas Rho* gene family, and duplication pairs with higher Ks ratios displayed greater differences in expression. This is the first genome-wide investigation of *Rho GTPase* genes in Mollusca, and our findings will assist in a better understanding of the role of Rho GTPases in Mollusca and in elucidating *Rho* evolutionary history.

## Supporting Information

S1 Fig Sequence alignment of the Rho family. The amino acid sequences of Rho GTPases were aligned using the same procedure as that in Fig 4. The characteristic structures, including alpha helices (α1-α5), beta-strands (β1-β6), polypeptide loops (G1-G5), Rho insert domain and CAAX box, are marked.
(TIF)

S2 Fig Relative expression levels of *PyRho*s in embryos/larvae and adult tissues analyzed by RT-PCR (A, B) and their corresponding expressions constructed using the RNA-seq datasets as RPKM values (C, D). (TIF)

S3 Fig Expression of *C*. *gigas Rho* genes in response to temperature (A) and salinity (B) variation. The temperature of 20°C and salinity of 30‰ (boxed) were used as the controls. ‘*’ represents significantly different gene expression (*p* ≤ 0.05).
(TIF)

S4 Fig Expression of *C*. *gigas Rho* genes in gills (A) and adductor muscles (B) after exposure to air. ‘*’ represents significantly different gene expression (*p* ≤ 0.05).
(TIF)

S5 Fig Expression of *C*. *gigas Rho* genes in response to heavy metal exposure. The digestive gland (DG) and gills from *C*. *gigas* which have been challenged with heavy metals (Zn, Cd, Cu, Hg, Pb and Zn+Cd) for 12 hours and 9 days were used for *CgRho* gene expression analysis.
(TIF)

S6 Fig Expression of *C*. *gigas Rho* genes in response to chronic exposure to zinc. DG, digestive gland.
(TIF)

S1 File Real-time PCR analysis for the confirmation of corresponding RPKM values from RNAseq datasets of three randomly selected *Rho* genes from *Patinopecten yessoensis* are provided. (DOCX)

S2 File Nucleotide and deduced amino acid sequences of Rho GTPase genes from bivalves. (DOCX)

S1 Table Sequence information used in this study. (XLSX)

S2 Table RPKM values of bivalve *Rho* genes during development and in different adult tissues. (XLSX)

S3 Table RPKM values of *C*. *gigas Rho* genes in response to different environmental stresses. (XLSX)

S4 Table Differential expression analysis of *C*. *gigas Rho* genes in response to different environmental stresses. (XLSX)

S5 Table Ka/Ks values for bivalve *Rho* genes. (XLSX)

S6 Table Primers used for RT-PCR in this study. (XLSX)
