# Supplementary figures and images for: The Rho GTPase Family Genes in Bivalvia Genomes: Sequence, Evolution and Expression Analysis
Source: PLoS One. 2015 Dec 3;10(12):e0143932. doi: 10.1371/journal.pone.0143932 (PMC4669188; doi:10.1371/journal.pone.0143932)

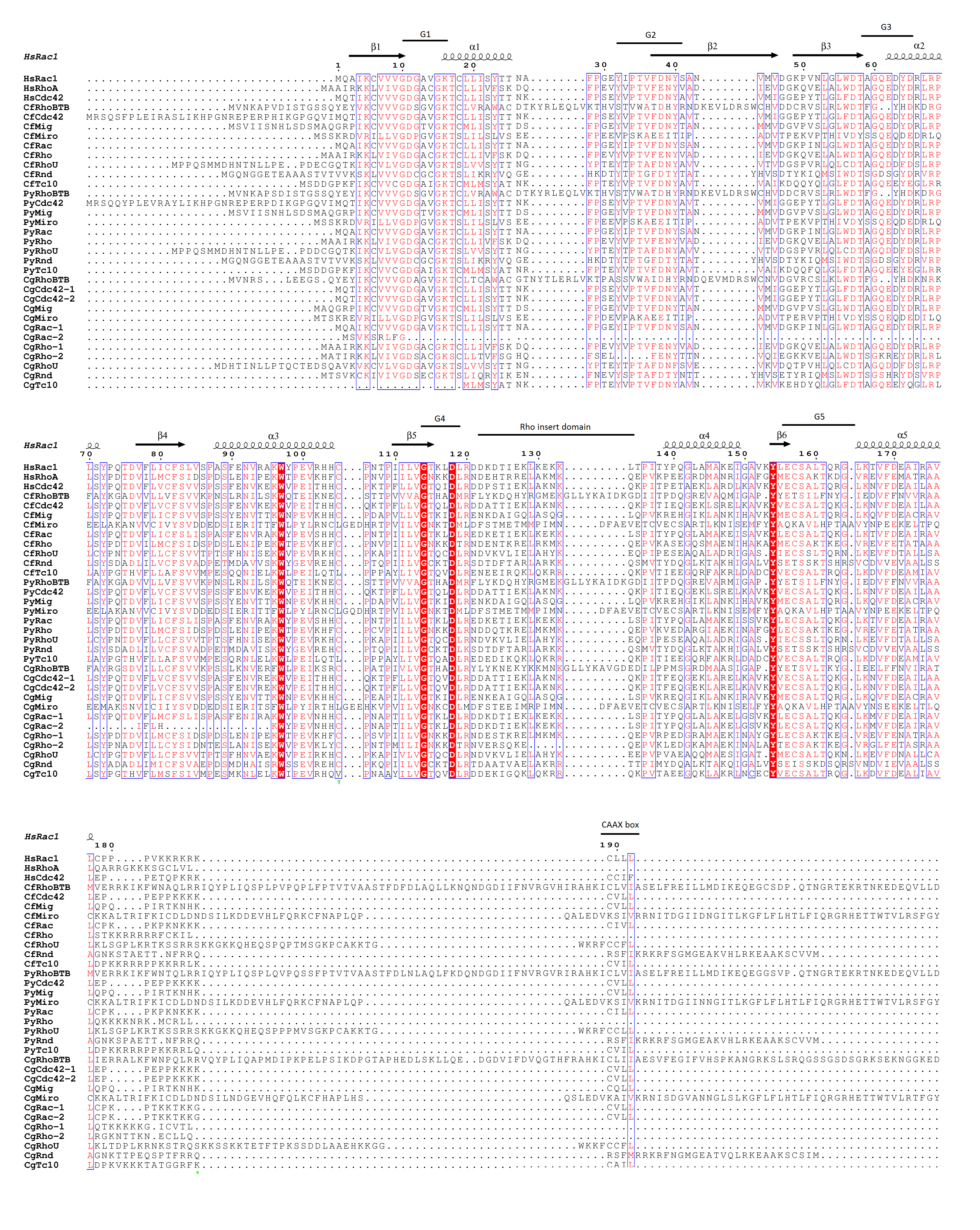

Supplement: S1 Fig — The amino acid sequences of Rho GTPases were aligned using the same procedure as that in Fig 4. The characteristic structures, including alpha helices (α1-α5), beta-strands (β1-β6), polypeptide loops (G1-G5), Rho insert domain and CAAX box, are marked. (TIF) [file pone.0143932.s001.tif]

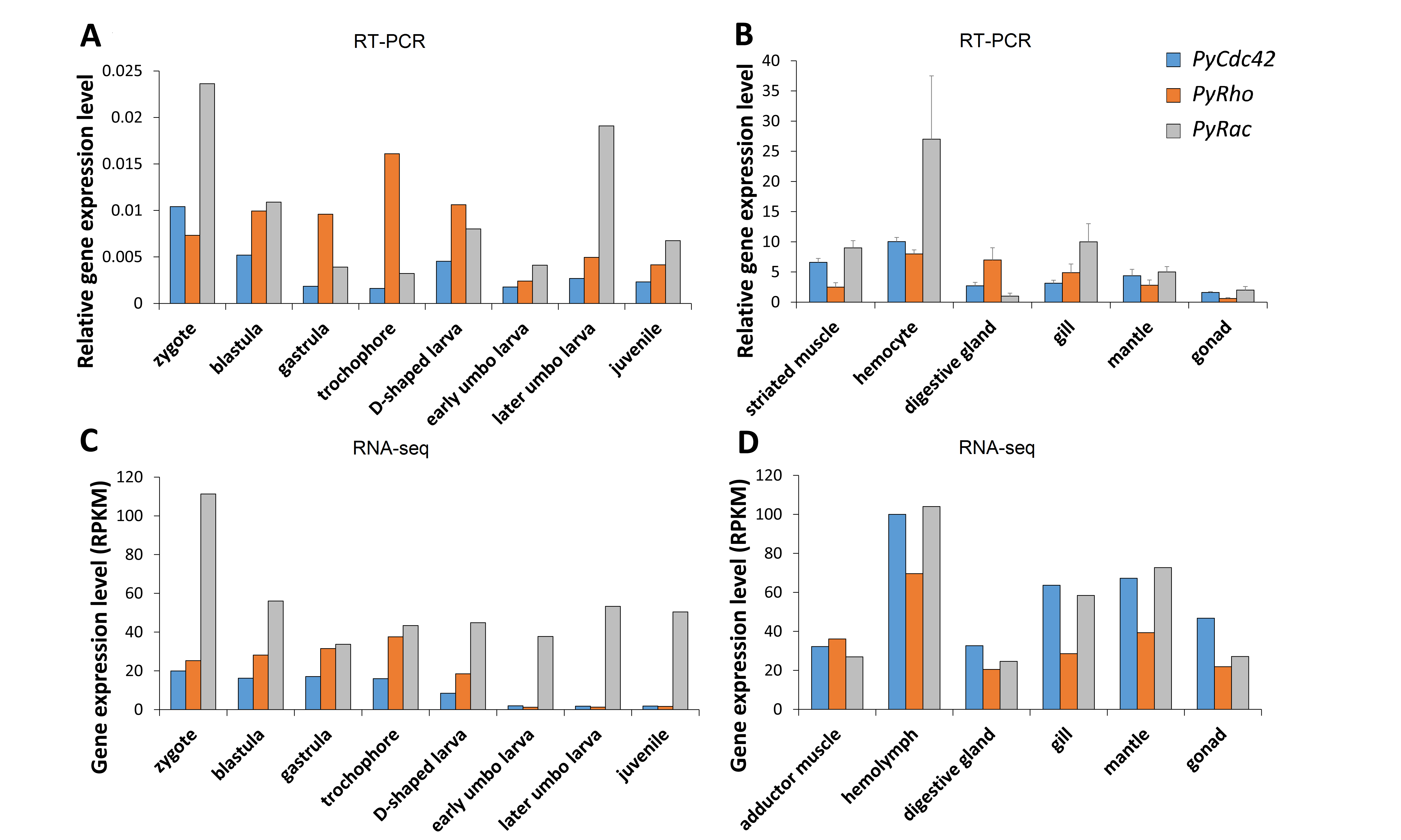

Supplement: S2 Fig — (TIF) [file pone.0143932.s002.tif]

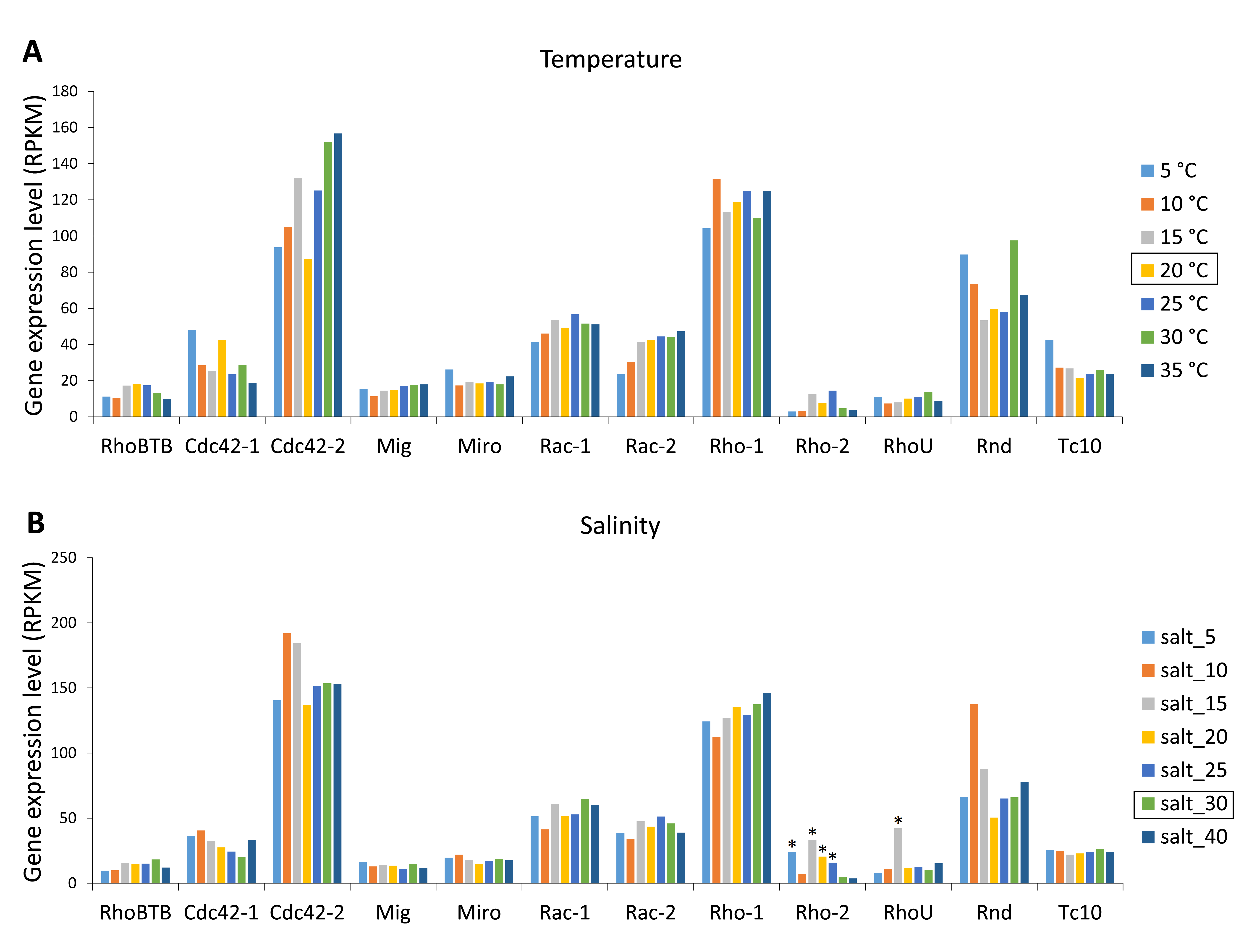

Supplement: S3 Fig — The temperature of 20°C and salinity of 30‰ (boxed) were used as the controls. ‘*’ represents significantly different gene expression (p ≤ 0.05). (TIF) [file pone.0143932.s003.tif]

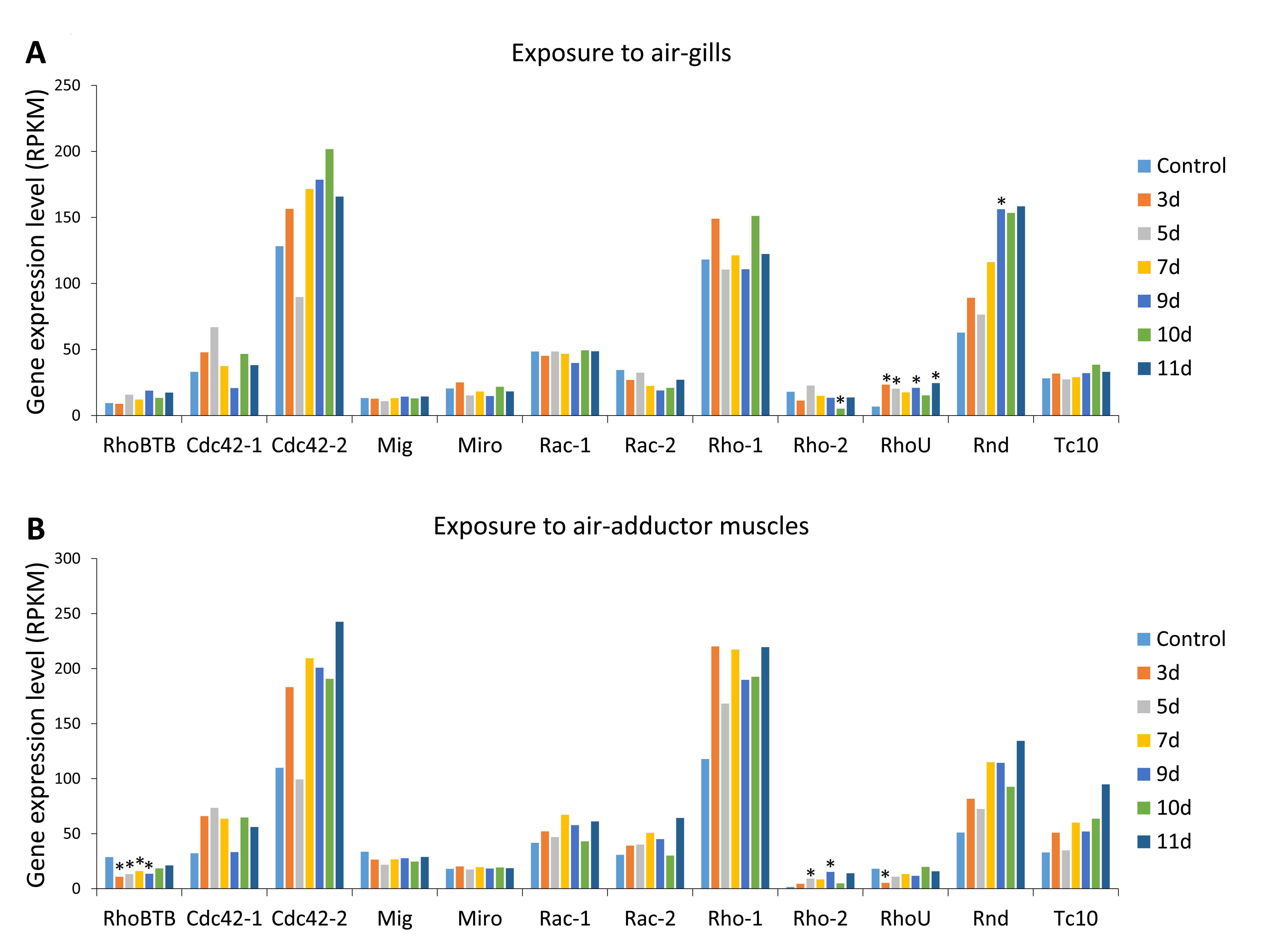

Supplement: S4 Fig — ‘*’ represents significantly different gene expression (p ≤ 0.05). (TIF) [file pone.0143932.s004.tif]

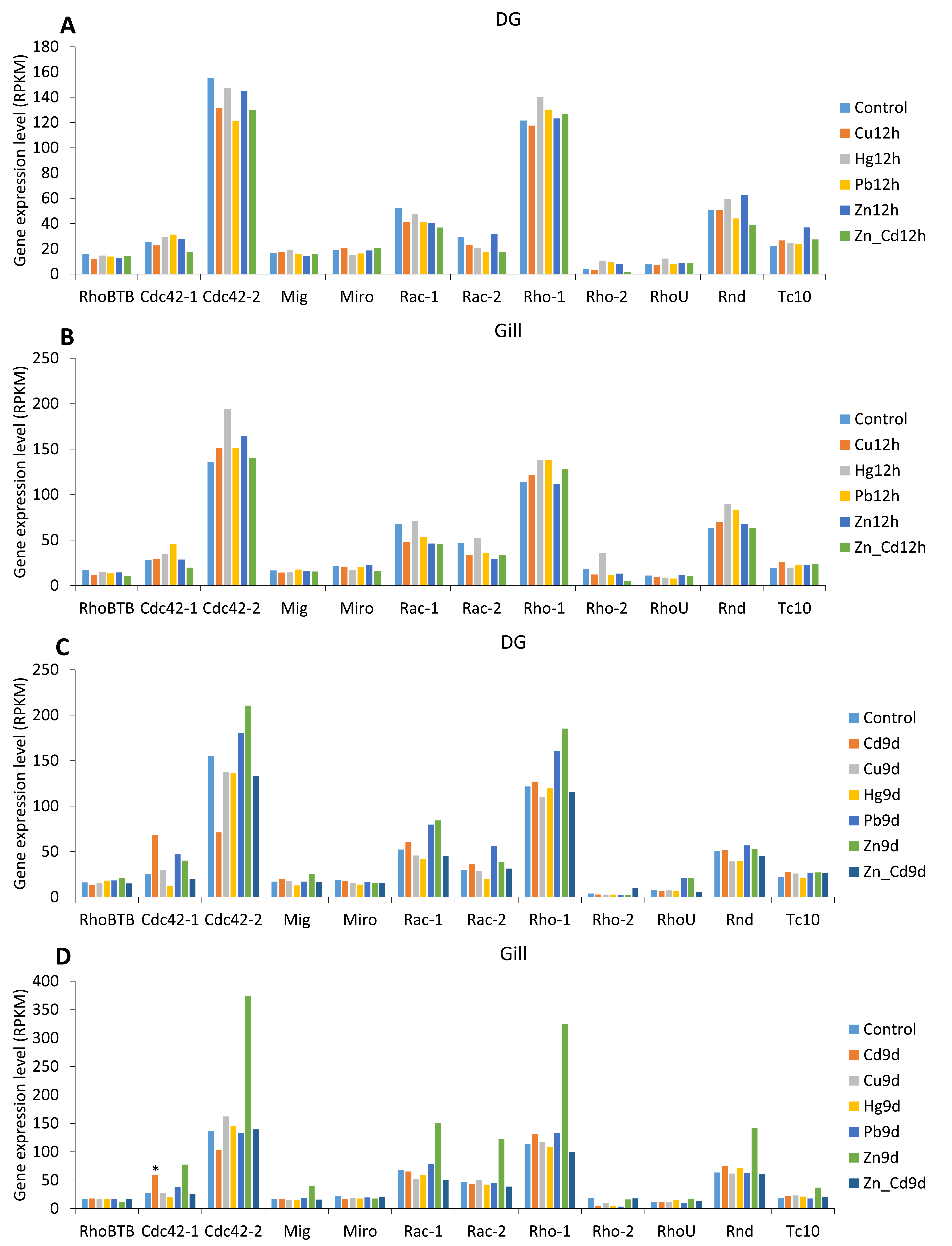

Supplement: S5 Fig — The digestive gland (DG) and gills from C. gigas which have been challenged with heavy metals (Zn, Cd, Cu, Hg, Pb and Zn+Cd) for 12 hours and 9 days were used for CgRho gene expression analysis. (TIF) [file pone.0143932.s005.tif]

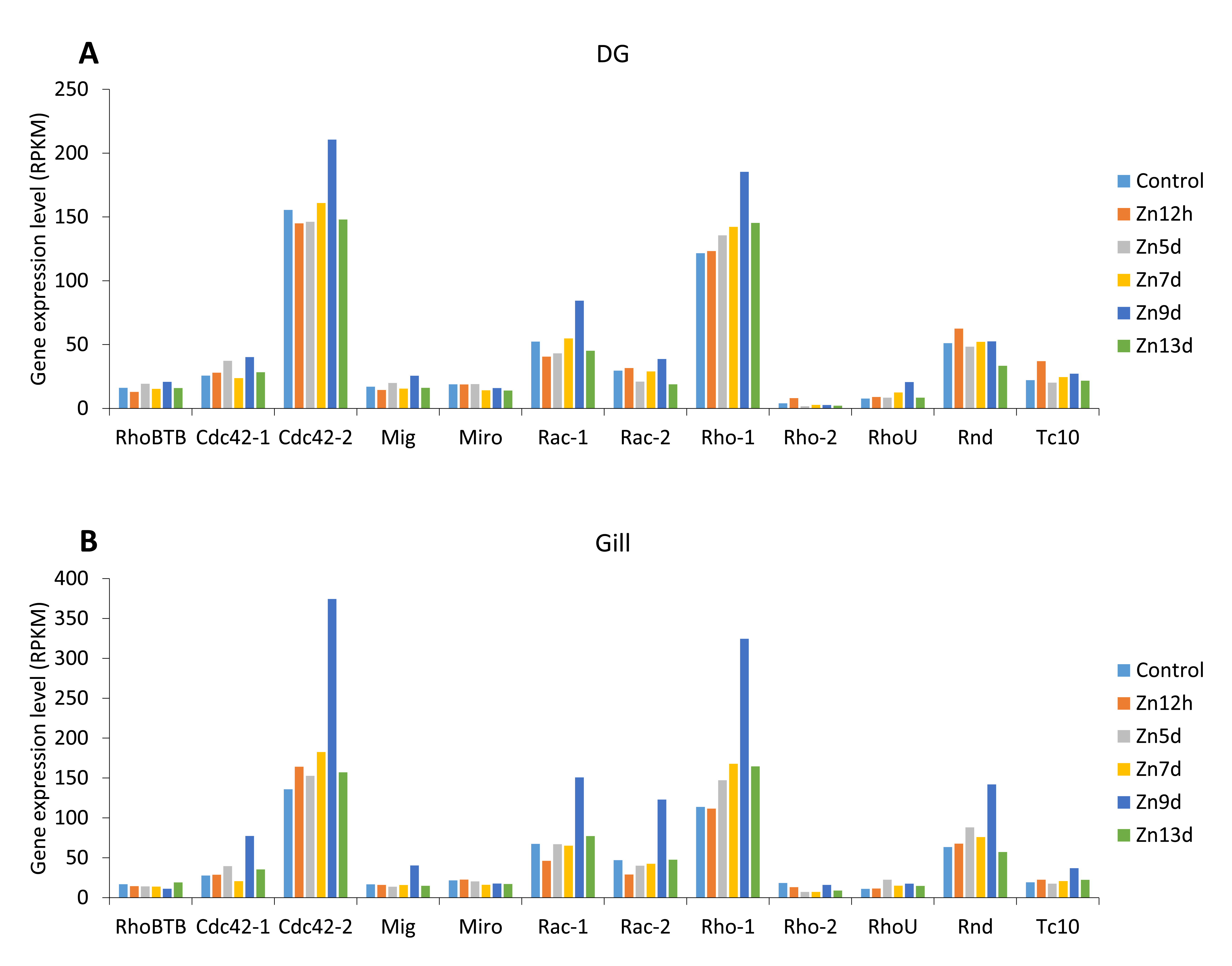

Supplement: S6 Fig — DG, digestive gland. (TIF) [file pone.0143932.s006.tif]
