# Supplementary material for: The Rho GTPase Family Genes in Bivalvia Genomes: Sequence, Evolution and Expression Analysis
Source: PLoS One. 2015 Dec 3;10(12):e0143932. doi: 10.1371/journal.pone.0143932 (PMC4669188; doi:10.1371/journal.pone.0143932)
Supplement: S1 File — (DOCX) [file pone.0143932.s007.docx]

**Supplementary file 1**

# Materials and methods

## Sample collection

All the scallops used in this research were obtained from Zhangzidao Fishery Group Co., Dalian, China. From six healthy Yesso scallops, tissues of mantle, gill, gonad, adductor muscle, hemolymph and digestive gland were dissected, immediately frozen in liquid nitrogen and then stored at -80 °C. Embryos and larvae were collected in the hatchery of Zhangzidao Fishery. Fertilized eggs, blastulae, gastrulae, trochophore larvae, D-shaped larvae, early umbo larva, later umbo larva and juvenile scallops were collected. All the sampled were temporarily stored in RNA wait (Solarbio, Beijing, China) and preserved at -80 °C.

## RNA isolation and cDNA synthesis

The total RNA was extracted from adult tissues and embryos/larvae of Yesso scallops following the method of Hu et al. [[1](#_ENREF_1)]. The method consisted in using guanidinium isothiocyanate to extract total RNA and digesting the residual DNA with DNase I (TaKaRa, Shiga, Japan). The first-stand cDNA was synthesized according to the manufacturer’s instruction of M-MLV Reverse Transcriptase (Invitrogen, CA, USA). A control reaction without reverse transcriptase was performed for precluding any DNA contamination. In brief, the reaction was performed in a 20-μL volume containing 2 μg DNase I-treated total RNA as template, 0.5 μM Oligo (dT)_18_ (TaKaRa, Shiga, Japan) as primer, 1× reaction buffer, 20 U RNase inhibitor (Invitrogen, CA, USA), 1 mM dNTP (Invitrogen, CA, USA), 200 U reverse transcriptase, added in the indicated order on ice; cDNA was synthesized by incubating at 42 °C for 90 min and the reaction was terminated by heating at 70 °C for 5 min. The cDNA was diluted to 1:30 and stored at -20 °C.

## RT-PCR analysis of PyRho GTPase genes

The expression levels of *PyRho*s in adult tissues and different developmental stages were analyzed using real-time quantitative reverse transcription PCR (qRT-PCR). The first stand cDNA of adult tissues from 6 Yesso scallops, and of embryos and larvae was used as template. Primers (Table S6) were designed for amplification of *PyRho*s cDNA fragments. Genes encoding DEAD-box RNA helicase-like protein (HELI) were used as reference genes for all the samples [[2](#_ENREF_2)]. For each PCR reaction, three technical repeats were performed.

All of the qRT-PCR reactions were performed in a total volume of 20 μL containing 1× Real-time PCR Master Mix containing SYBR Green dye (TOYOBO), 0.2 µM/L of each primer and 2 μL cDNA mix, with a LightCycler 480 system (Roche). The qRT-PCR amplification was performed as follows: initial denaturation at 95 °C for 10 min, followed by 40 cycles of 95 °C for 15 s and 60 °C for 1 min. To exclude the possibility of non-specific products in the PCR products, a dissociation analysis was performed by subjecting the samples to a constant decrease in temperature (from 95 °C to 60 °C). The PCR products for target genes and the reference genes were purified and sequenced by Sangon Biotech to verify the specificity of qRT-PCR products. The results were analyzed using Real-time PCR Miner (<http://www.miner.ewindup.info/>).

# Results

## Spatiotemporal expression of *PyRho*, *PyCdc42* and *PyRac*

As shown in S2 Fig., transcripts of *PyRho*, *PyCdc42* and *PyRac* were detected over all developmental stages same as the RNA-seq result. The transcripts expression level of *PyRho* was gradually increased before trochophore larvae stage, and decreased after this stage. The transcript expression level of *PyCdc42* was continued to decline since fertilization. A similar expression level of *PyRac* was found in the zygotes and layer umbo larvae, which was much higher than that in the other stages. A relatively lower expression of *PyRac* was detected in the gastrulae and trochophore. *PyRho*, *PyCdc42* and *PyRac* gene transcripts were widely distributed among all the sampled adult tissues of Yesso scallops with higher expression of in hemolymph. These expression patterns of the *PyRho*s genes were similar to which performed with their corresponding RPKM values.

# References

1. Hu X, Bao Z, Hu J, Shao M, Zhang L, Bi K, et al. Cloning and characterization of tryptophan 2, 3‐dioxygenase gene of Zhikong scallop *Chlamys farreri* (Jones and Preston 1904). Aquaculture Research. 2006;37(12):1187-94.

2. Feng L, Yu Q, Li X, Ning X, Wang J, Zou J, et al. Identification of reference genes for qRT-PCR analysis in Yesso Scallop *Patinopecten yessoensis*. PloS one. 2013;8(9):e75609.
