# Supplementary material for: The Rho GTPase Family Genes in Bivalvia Genomes: Sequence, Evolution and Expression Analysis
Source: PLoS One. 2015 Dec 3;10(12):e0143932. doi: 10.1371/journal.pone.0143932 (PMC4669188; doi:10.1371/journal.pone.0143932)
Supplement: S2 File — (DOCX) [file pone.0143932.s008.docx]

**Nucleotide and deduced amino acid sequences of Rho GTPase genes from bivalves**. Capital letter: coding region; Lowercase letter: non-conding region; Lowercase letter highlighted in yellow: untranslated regions (UTRs).

The sequences and exon/intron annotations were used for reference building in STAR mapping.

**CfRhoBTB**

1 aggatgtgcatgaaaactgatagaaactgcattgttcaatatatatttttctcatttttatctaacaaaactccaaacatcaataatcattacacataccctgca

106 gtggctagataatgtgactacaaaagcatattttgactttgtgtaaagttttgacttggaaaaggatattttagagaaattgattaatttctctcaaaatgaagg

211 agtttccctaatgagggaggattacctatgagtataaactagcacataccattaattaaatgctgaattttttaaaaattattttatttctcagaccaaaacttt

316 cattaaatcataaaactaattaaaatgctctcagtttgtgaacttaattcatatgtgggttttaattgaaaaccttcaaatgaagtaaaaaaaaagtggatttca

421 ccatttcaacttggttttaacaatgtaatttaaatgcaacttcatcaaagtaaaatggtcccctccttttatttgttttcctgcttcaggctgccggtaatcaga

526 tagctgttataattaaacaactaaacaagttttgtttattgtcttttcagggcttttcagagcatttttttgtgacagaaaattggctccatgctccattgaact

631 ttttccatatttaagccaaattcacaaaattttcagctaaaaattagaagcaccttatctttttgataataaatgatacatatgcctcataaaagttaaaactat

736 cagaacaatgtataattgctgaaataatgaatctttactaatgaaataaccccttcataaaattacccaaatgatctgccttaaaaaatggaaatgactgccatg

841 acagacatctcaattatcaattatctgtttctaaagcctgcatgcatggttaaatttagaattaatactgatgttgccatgtttactacaaattccctatttggt

946 catgtcacatctatatatagaaactaactatatcagaaaaataattcattatggcagtcaatttagattttgcttgccttgatcatataacaacatatttgccag

1051 agtttcacgtcaagttaaacattgtagcggaactggactgggtcaatggttggttggttatattgtttaacgtcctatcaacagctaaggtcatttaaggacggc

1156 ctcccctgtatgcaaaatgcatgtgtgtagtgcatgtctgtgtgttttgggaggcaggggtgtgttcgtgtattgtctccttgtaatagcgcggatttgatgccg

1261 actttacagtgctatctcactgaaacatcctgtcgaagatacagcagaacatcccacccggtcacattatactgacaacgggcgaaccagtcgtcccactcccat

1366 aatgctgagcgcttagcagaagtagaaactaccatttttatagactttggtatgtctcggccaggggacacaaccaaaagctttcctcacaggggcgaacgctca

1471 acatatggacaaaagtgaggcattgtcaagggagatgataggaaatggaaagttggttataaagaagagaaaagataaaatcccataattagttgcctcttacga

1576 tcatgcaatgggggcagcaggtacaattcttacaccctacctgcagggggtctgggtcaatggtctgcgaccagatagctcaagtggtagagtgtgtctagagtt

1681 cagaacgtcccgggttcaaaccccggcctggctgtgcatttttgctctccagttacaaaagtggtgcctgtaaccaacccttctttcaagcacttggtttatgct

1786 tggcaaggggatacccaaacctcggttgtaaatagttggtgttttcagaggcgaagacttagaaaaaggagggaggaatgtagcggaactggactgggtggatca

1891 ttggcgaccaaatagctcaagtggtagaatgtagaaggtctcgggttcgaaccccggtctggccgtgtttttttcctctcctattacagtatattgggagataac

1996 cagtaatacctaatgcttatttgacctgtctgattgacacctagtgatttactgtatttatttttttgtccatgcagttttacatgaacttgcaaccaaacacaa

2101 cattgggagaccagtaaaagggtctcataataattagtctgcagcaagattaaagacactactgaagggatgattatgcaacgatatagaacttggttcaattaa

2206 cattgttatgctgattatgttggaatgtagaattcatatatataatatatatatgaaacatcctgcattttattacatgtagctttaatttattttcttcttaca

2311 tgcttcttccagtgttgcagtctctgcatttggaataacatccatctaatcatttaaaattaaatggccaatatgatagagaataaattttctttttaaaatttg

2416 tacaaaagtttaatgtaataatttgaaggtgtaattggtgaaccagatactagaatctatggtaaataaatttatttaaaacatattagaaaactaaaatcaacg

2521 aaaacttgattcaaaatgatatgcaactaaaacttgatatgaatttctaaatgaattgatgaaaaaaacacgtaggcttgcacaaaaaaaaaagattattttcaa

2626 tgaatattttaaattattgtcatgatattaggcaacctatctagggggagacttgatttcagatcctgaaatttagtattttcagcgtaagcatttgctttagtt

2731 aaggtttcatctaaaagaacaattcaaatttgtcattcagagtgattatttaaggtggctcaacacacctaggaacttactcagtaacttcttagaccttagtat

2836 agcatctaggttagttagctcattatgttttttccgaaaaccatggaaaatgcaaatttttaaaaataagatttcattagcttttttgatgctgcataatgatgc

2941 atttttatattaaaaataatgacaagtaaaatttgtttcaaaattttatttcaaactaatttttttccaaaaaaatacccccaaaaatgtcaaaaaccgtcatac

3046 attgtatattaaaaactgacggttaaatatctatatttctttctgtaaaatattctacaaatatcatacctttaatggaaactttgaaattgaatgaaaaataaa

3151 ctatgaactacatatcatcaattaaaaaaaaatttacagtgaaacccggaactttttttctgaaggcatttttagccagtgcactgttgaatttgatatataaag

3256 ttaattttcgaccacttcacactatacaaccatttaccaatcatttctacataaaaaaaaaaaaacacaaaatgtataaatatcaaaattatgttttgtttgatg

3361 tgcaagaatgtaatcataacaatgtttcaaatatgaacaaaaccagagtcgggcactacaacgcacaggggctaggcatggacctacgtcactaagaaatgattg

3466 ccaggtccatttttgaaccccgtcgagtatgacccgtcccacgactacatatctttctatttttgaaagatatgtagtcgtgggccgggtcacactcgacggggt

3571 tcaaaaattaacctggtaatcatttcttagtgacattatataggtccgtgcctagctcctgtgctacaacgtagagatacagaactggggcaggactgatatttt

3676 ggttattgtctcgaaaattggcacgattaacaaacttgcagttactgtacgcatggtcatggtgcacatgattattgtaatctttagtggttatctctgtccctt

3781 tcaagatctaatagtcgacgttttatttagcaacacagtcagtgtttcttcgttcgtttgacaacactatcggaaacgccttcattgtaaagcgttcaagtgatt

3886 gagtttgtttttgtagttttcaaagttggagagttactttataaggtgaagggcacttttcgcccggaactgttcagtctgctatacttttttcggaagtacgtc

3991 ataaaaatggacgctgatgacgatgtgtcgagtaaccttaatagacaatttaattttgagcttgcaaaatcccccatggaaattttaaaagctggcaagcatctt

4096 atatcaagtgacaaatgataagttacatcaaatcaggattaacattatgttccatcttgaaaaacaagtgaataacaataccaaattaaatttcatcatcaaata

4201 tatatatgcacatttcaataaataatctgtggcagttttgaaagaccatggaggtgcagaaaaaaacaccaaaaaacacttattgcctgtacacagtattaaccc

4306 cagggtacggtgacaacgagttatattaacagctgcgatgattcataccgctcaacatgagtcaagtgtccaacaggtgaaatcaaccctaatcccgacactgtt

4411 gtacagaagtcgcgagaaaccgaaatgattcctgaagttattgacccctgacaacaaacacaatgttttcaatcgtaagatttgctatcggcctaaacatctaag

4516 tgtaatttgatgtgatatggtccccgatttatcaataaataaatagaaattataaccggaagtatatagctggtagtgacactgacaagagaaaatgggcgggtt

4621 gccgataacaaacagattatttcagatttgcgcatgattggagtgtgcttaacattaatccaaactattaattgattaagaaaagaccagtctcaccaatccgtg

4726 tctgacttttcacccacaacctctcggtaactcttaataagagcatcttgatttttcctcatatcaagcgccatcctgctttctgcatgtgcttgtgtactacgt

4831 caccaattcaggggtagtgtcacggtttgcactcaccctcgggcataatatacactgcgatctctccagaaaggaaagtcttgcaaactggaatgaccgtcggac

4936 attctccggtcaagaacaggacacgcttgaccgacctcggctaagcctggtaattatctaattacgtaatgtttagtggtcattttacggtttgttatctcataa

5041 tgcgctcctgccgtcaatcagctgtcagcgaggccacggtttgtttacaaacatagtctaggaatatgaatggcgttgcaaaaataggccactgtgaacgtatac

5146 cactctacgggcaatgtttagacataacgtttttataagctatgtaaacggatttgattatgtttaaatcataaagattgaaaacaacttaatcagatatcgccc

5251 tagaattcgaaagatggcttaaataatgtgaaaaattgaaatgtctgcataatattttgaagcggtttctaaatatagaatttatgcaactaatataggtaaaat

5356 tattactttattaaagaaaaaaactgtgaataagttgtacctctttacatttatgattttattataaaattcttcataaaagtgtgagaaattgataatttagat

5461 ctaggaatttttttatattatccaaacttcaaagtctacttattattattggatgcttggtgtacaaaatgtacaatttatactgcaattttcaatgtattgcat

5566 catttgcattagttattacttgtctgtcttaagcctggaatatcattatgctgatttagatttagtctaattccatatatattaaaacttttcttatttatactt

5671 actttaaattggaaacacacacgcatatatctatctatatataatatatagattaattcccatattataaaaaaatatgttagcaccagtatttgcgtaaaattt

5776 actcagatcgattcaaagatttcattgtgagagtctttattagttatgatccctggtattaaattcgaatcagtggatttgatttgtgcttatagtcaccagctg

5881 tcgctgttcgtgaattaggttgatggattggtatatgtattactgttacaggtttcaacctgttgccctttccaatgtacacatactgtacacacatgtcgtcgt

5986 caatttaaaactgtcattgtcaatacacataacggaaacaattcacaccatgtcaccatttatcatatacatccgaccatttattgacttttaaatctgatacca

6091 atgtcagtgttcattgtgtaaatgtttacttacagctatctagatatacttttaattgctaatctgcaataaatgttgtcattaacttatattacatctgacata

6196 aaacagtcgtaactaaactattctaaagagcaaacatgcaagcaattacctgataattgtatcaatgtaattgtatgctttccaatagattgaaaatccgaactg

6301 aaaaaaaaggactgtaccaggggtactttttccaacggattggtcaaggcacaatcacctgtcaatgctccagataagatgacatacatgtattacatttaattc

6406 acacacgataattcacaaatcacacaaaagaataaaatcaacttttatgaattcatctttatctctgggttcaacttagccgactgtagttacttacgcttatag

6511 tgaatcactgagacacatgcagagtatgcaaatcagtgttaattaagagacaagctctaattattgttagagaccagctttgataataaaccagttttgaatcta

6616 aatttccttgggtggtctccttgtactgatttatctgtacttacattgaagcaatatcatactctaaaagagaaccgcccaattggtcaattaatcctggtgaaa

6721 tagatttgacagttctgggaaaaaccataattgagtttaaattatatttatactgtcagaggagttcaccaaaacctgatatacttgtttgtgtgactatggagt

6826 tgattgaatttaatatttaaaaaaaaaataaaaaaaagggttgaatttaatatttaggaaaaaaaacgaaaaaagggtatcagaatcaacagattttaaaatatt

6931 aaaattatgaaaagaactaccgtggtagctaacctacaagtgcttcacatagagcaacagttggtttcattgacatggtattagataaagtgttcactatactac

7036 agctagagtattcatggagatgtagggcttactgctgcctactgaattatttattactattgggaaaaggtgggttccacagagttataggataaatgctgtcca

7141 ttgcataatttatgaataatagtatcaactaatgattgtaaaattagataaataaaacaaatctggtttgttttccaaaactttttctgcaattcattgcatgta

7246 tgtcactttgccaaattatgaagagctgactttcattaaattttcattaattattgaaatttcttgatatggagtttagagcttagtaaataaattcaataaata

7351 aaacaaatctggtttgttatcttaaacatttttctgcaattctttgaaggtatatcactttgccaaattttgaagacctggctttcattaaatttttcattaaat

7456 taatcattgaagttccttgagatggagttcagagcttaattaggttaatttatacaaattattacattaaaatctgtatcgaagatttagcaccttacgcatgag

7561 tacttggattggagggggtgtgatctgacaggattggagtaatctgggtgggagcaatgtacagttaattgagctgaagcaaccttggtgggacatacatgtata

7666 tcagtcacataattatggtgaatctgggagaggaggagggtctatagtcaaagaatttgaagcaacctacatgtgaattggtcatggataacaagcattatcagg

7771 atgtggtgcacactggttaaaaaagtgacaggatggcaccagattttaactatattgatgctctcaccgtttgaggtgattgaactaggtgcttggcacaggcac

7876 cactgtatctgtaatagaaaaaatgtacagattctccaaactatagatggcctcttaccgtttgaggtgattgaggtacatttgtaggtgtttgtccaatctgat

7981 taaaatgcagatccttattatcattccattacataaggatctgacaacatccaataagaagtcatgttttggtgacctttgcactcccagtacgtgttcactggg

8086 caaattgtccagtttgtgatgacagctacagcaagttattttgtcacaaaatgcacatagttgttttttgcgacgaaatgacttaaattatagattgggttagca

8191 agcatttaacccaccaatattcacatgcgcgatcctgtaagatttgattggctaatgcaaaaggtcgccagaacatgacccctatgggatgttgtcagatcctat

8296 gtatgcggagagtaacagagcaacaataactgaatattaaggatctgcatttaatcagattggtgtttgtctcaaatacctctttgttatcaatacaatatctta

8401 tgactacactgagattcgaacacagggcctctaaactttaggtcgactttggatgtaaagccattaagctacctaccagggatctgtcccaggacgcacttactc

8506 cttattttaaacagaatttgtgttgaagatgtttccatagccttttgaagcatagaaaatataaaggacctcaatttttcaaaggaggaggtccattcaatctat

8611 acaaattaaagggaccctacatgtatattgtctgaaaaggaccccattcttgaaatacctagtgcaaatccagataaacacttgtaacatttaactgtgatggtt

8716 aaatgttattaatttgagttttttatttaaaataggaaagaatttatgggcatatacactcagcttggtatattttcctgtcaatattttgcacaaaacttgtca

8821 ttgacaaatgtgtgttaattatgtagaaaaaatgattgtttgtatattatgtaaattaactgctgttagcctaaattcttctcgcatgactgtatttagaatgat

8926 ttcatttattcttatgagacagtttaatttgtaagagtgatgtatatttcacaggcctagggaacagacttacatagatatgtgtatacatatatgtatctatat

9031 atatactcttatgtatgttttacatttggggacaaaacactgcaagttttcctggaaatttgcctgtttttttttttttgaggttatttatctaacttgcaaatt

9136 taacaaaataagtttatgtaaacaaatgtccttttcaccataatgttgcaagacctgacaggtgtattgtacttccaactcaatacttgtgctttacctaggcta

9241 aggaaatacaacatttccaaacccattactcataattctaaaaaaggactcttctttttaaaaaatattagatactttcttgggaaatttaaaaatataaatata

9346 aaagcataaatcatgtactttttacattgagtttttaggacacctgatctttagctcatttatatgtcatgttttcgaaatgaaatttcttttcgctgactaaac

9451 ttaaagttgtgaaccattcacagtaaaaatatatgttaaatattaaaaaacagttttctattttggtcgggttataaagaaatagttaattcaatgtcccatata

9556 tagatgaatactggcccctcaatttacactgctcaactggacgttatcaattcataaatttgaaggtatggaaaattatttcttaaagatatttgatacttgtaa

9661 ttaataatcatggatgctattattatttcaaaattttttttattaaaaatcaacttgacattatcaattcataaactggaaagtatggaagattatttcttaaag

9766 atatttgatacttgtaaatattaatcatggatgctattattatgcatgcaaaaaaaaacaccaccagttaaaatcatatgatacacatctgtctctcatgaatga

9871 tctatgcttgtctgagatcttctgtatcatttttgtttaaaattcatccgacaggggaattgtacagtagaatatgttgcctgaacttattatgtaaatgagatg

9976 ggatatatgtgtatagctagaagcagttcacttgtcatgttgtatatatatatatatatatatacacatgtatcatattttttccttgtaacaactgtttttgta

10081 tattgtcgcaatcttaacgtttatacttgttatcacagctgagctccacttcagccgatatcacacagtctgacattctgattaaaatcatatcctatatatatc

10186 tcaatgtattatgtaaggaacatcattatcatcatatatatatcatttcaggaacaggttacatacagagagtaggatatgattttaattggcttgcacagacag

10291 ctaaaggatagggcctttgatagcctagagtgaggtcatttccatgtgacatgctttcaaacttattatgtagttgaaaagataaaagggggtggaggagtccaa

10396 cgtcccgtgggtggtactgatcgatagatgtgttggtggaggaatcctgggtggatacaggagtattctagcagagctccatagaccataggtaataacaacttg

10501 atgaaggattggagcatcaatgccaaggattggttttgaagacggttggcctagccaactgaaacatgtcaacttatcaattccaaatcaacagaatccttggcg

10606 ttgatgctccatccttcatcaagttgttattatgtagttgttaaatgaattttgatagtgttaaataaacttataaaaaatgtaatgcatatcatctaattatat

10711 aagtacacagttacaaagtgggaggtaaggtggtgcagtggatagtgcacttgcctttcaccaagacggcgggggttgactctccgatacattatgaaaaggtac

10816 atgtatgatctgacctgcctgaccatatgggttttctccaggtactctggtttcctcccacaccaagaacccaccccccacccttacacttacatctgggccaac

10921 aagagtctgttgacattaccaaataatataagtggaatattcaataacttcttgttctattgctgtaaaacgaaaaaaaaaaaattgatattaagtaattattat

11026 atcatacaattgtaaataaataatacaatatagaataccatggataaatctcaataaatatgagcattgtgaagacgttctcctagatagattggcaacatcgaa

11131 gacttgatctaatgatcatgattattatgaaaaaagccctgtatttttttcatgaatattcatttaactaaaagggttctgaatttgtgtccatttctgtatttc

11236 atatgatatgtatatttatgagctctattacaaggtttctacatttggcatttgcttcttaatgtgtttttggctatatcatgtatagtgtattaacatctcaca

11341 ggcctatccttgtttatttaaagaaacatatatatactttttatggtatgatgcttttatggtgtgttgcagcgtccagttttcgtctggcatcatctttgtact

11446 ttatacaacttcttctgaataactaaaaggcctgtggtgatgatatttggcctgtaacatcctgggatgaagggatacaaataaatatcattaagcaaaaggtca

11551 agggtggtgatatttggcaaggtgcatcctgaaatgaaaggccttgtttggaaatctaggtctccgggtcaaatttaattaaatgatttaaaaaaaatatattaa

11656 cttgtttaaaaaaatttaagtcattaattctgatattttgcttgaagctttatttttagtaactttttaggctgcagagattaatatataccagatgaatgatgc

11761 aggcctattgggcctgttgttcatatttggaaggaaatatgctgcatataaatgtactatgtgtacacgtttttaatcaaaacagtatggaatctccaatttaga

11866 acttgttaaagagctttatttatggggcacctgtacataagtaattaagtatttcactttaagtaccttgttagtttgatggtacatatatatatcttagtcagt

11971 ctgtctacagctggcttacaattctcattctttataaaatgtatgcaaagtgcttgtaacagttagttgggtttgtgtaactgagttgggttcaagtatacagtc

12076 tatggggttagaattacactcatttgcatccatgtttttatagaaacttgctgtagcaagttcttcatctttgaaggaattctatctcataactctatggagggc

12181 agaaggtctagcaaagtccttcatttatatggtatcatgttctgctgagcaagcaatattaaactttatttattcttttatttagacaaatttaattttaaatca

12286 ggttgaacaagtgtgttttaaattgataatcaattcattctttctttagacagatttttaaatcaggtttaatatgtgtttttgaaaatgtttaaaatatataaa

12391 tgtaaaacaaagactcgcttggtaattgagactttagattcagcttatcagtcaactctgatttcatcaaaatatatgtgtattttctattggcttaggattata

12496 tgggtgagtgagtaatctcaatacatgcatacaattcttcgaccaattaggcgttggcctttaactaacatgatattgtaattataagtcacttttacgtacatg

12601 ctatatatataagtgtgtgcttgacgggcccggatagtacttcagtgttctgtctttaaaatcacagcacaaacagtgtaatatgtactgtaaccatgttatcca

12706 caatccaatggtgtcgcactgggcagtaaaggtaaatatgtaactataaggttagcactgagtgtttgggtggcgcagtgtttgggtggcgcagtgtttgggtgg

12811 cagtgtttgggtggcgcagtgtttgggtggcacagtgtttggttggcgcagtgtttgggtggtgcagtgtttgggtggcacagtgtttgggtggtgcagtgtttg

12916 ggtggcgcagtgtttgggtggtgcagtgtttgggtggcgcagtggatagcgcactttccatcactctggcagccggagtaattcccagataggacatgaaaaggt

13021 tttgggaatttttttaagttaatataacttgtttcacaattgttgtaaaataaataaagtttgtatatttatatatagcactgaagtaaaacttcagtgctatat

13126 aaatatacagtgatacatagttaacatggaagagaagttgtaatttttgtactgtgaatatttctgttgtatagatgtacctggctggccactatctcttgaata

13231 tttaataattaaaaaaaaattctatctctaaaaattttgatccctaggatgtctgatcctaagttctaaaccaaggggagataagctagtggtaaaaatgtagca

13336 gaggaaatattcacaaaaacttattaagggctggtagcgagtctaggatgtacccatctttggaggggtttatcatggcatatcagtttgtttgtcaacaatttt

13441 cagtgattttttggggtacatttgaagccgaataaaggccccttccttcccctagagaaattttgctatattttcccaatttcatgacatgttcatattttccca

13546 atcagaaaatatgagacgtatatgaacagggacatttttctcatattttctgattgggaaaatatgagaaaaatgtccctgttcaatttgaggaaatgttgattg

13651 ttgaaaaaaactaggaaaatggtcatatatacatctcatttatttgaagcttaataaataagcataaaacaaatatctgctcatgctatttttctgtgtttcaaa

13756 tgaaacaacacatttccccatatggcagaaaataccgctgtattttcccaattgaaaaggtacgtacaggcccctgtataagaaaatcactgattttgtcttcgc

13861 tggttaattttagtaagaggtacgtttcaatttctgtgtgattcttctcaatacattataacccaacaggtttatataatgactagtaatgtacaagtaaagagt

13966 cagtgtaatgtatatatgaatgtcacatctatcatcagccactttcagattaacctgtgtgaatatctattcagaaaaaaattgtacggggaccacatgtacata

14071 gcgtagttctatagtgtgttgccttaagcttattgactacaaagtcacagggcaagaaccagatttattaaaagcaatgagtatcttacttattggaaatgagaa

14176 agtaatgaccaagaggtgaattataattcacatgttggatgtccaaaaattataatctaattagcgggtatatgtacattgtacatataattagacttaattaat

14281 taatgaaatttaaattcacctttatcgccttcattacggtctttttgcagaaaacaaagaaaagttttacctatcctttgttttgacttgttataatatatttca

14386 atttttaaaaacatacatgtccatcacaggcatgcaggtcaaatttttcactaacatacaaaccaaaagtacgtatactggccctgggtcataggatcatcagac

14491 ctgggattgtttcctatccagggttacattatgtgttaggatccaacaggtaagaggtaatggtatagagcaaactatgaaggagttgttagaaacactgaccca

14596 taacaaagaggacacctctacatgcaaagggcccaaatgggatgtggtcaggctatgtccactggtcactcccctgatatatctgtgtagtcatcactttatgta

14701 gatcccgccgacaaaacatggactcatgtcagtgagaccaagcccgtcagggtggagtcacggaggcagcaaaaaccgcttaccaaccaaccaaccaaccaacaa

14806 acaaactttatcaagctccataatgtgatttgggattgaatagcacacttcacaaattttgtagaatactctctaataataagtgaaatgaaaatataacactgt

14911 attttaaagaagaatgtgatagagattttttttaaaacatatcaaaattcattcttaataaaaaaaattgtcacagtattttgcaccagaaaattaactctatta

15016 tatgtaggtgtgagattcaacctgtccatgtataagaaacttaagtactggcttactaatgtagataaataagctgtcaggattttctgagtgatttactttaag

15121 ctgtactattttgacagaaagtttgtggtgctcctttgaagttcatatacaagtgtgtacagtacaggtctgtccattagtaggtatgacctgtgtgccccatca

15226 catacattgtatagcttataactgtatggtacagatcatattatgccccgccataaaatggggcgggcggagggggggggggcatatagtgttgactatatctgt

15331 cgcgtcctctcccttcccaattcaacttttacacctagcaacttcattctttgacagatttccattaaactttatacataggtcgagtatgactctacctctagc

15436 atgggtgtgtttcaggcttaaaaggccaaggtcaaggtcactgtcactaaatatagaaaatatggaatcaagtggcagcgggggcatttatgtcttacagccatc

15541 ttctagtcagtaaaactactaataacccctgatagttgagtggtgctgtgggtagtcgagtggtgctgtgggtagttgagtggtgctgtgggtagtcgggtggtg

15646 ctgtgggtagtcgagtggtgctacatgtgggtagtcgagtggtgctacatgtgggtagtcgagtggtgctacatgtgggtagtcgagtggtgctacatgtgggta

15751 gtcgagtggtgctacatgtgggtagtcgagtggtgctacatgtgggtagtcgagtggtgctacatgtgggtagtcgagtggtgctacatgtgggtagtcgagtgg

15856 tgctacatgtgggtagtcgagtggtgctacatgtgggtagtcgagtggtgctacatgtgggtagtcgagtggtgctttgggtagcacactcccattccaccaatg

15961 tgaccagggttcaattcccaggccccggtttcatcaacgttccttaacttaaggaaactccataacttaaaattttccataggaaagcattacagaatgataagg

16066 aaaaaagccttagttaaggaactttccttaacttctgtaatgctttcctatggaaaattttaagttaaggagttaagttgggagtaactgagtgtcattaagttg

16171 aggagttaagttaaggaacattgatgaaaccggggccttatcacggatcggccatgaaaatgtttgaggtcacatgccagacaacttgggttttctcagagtact

16276 tcagttccacccatattaggacccccatattataacattcaggacaacaagcgtgatttatataagctaaggtaaattgtttcataattgttgtaaaataaagtt

16381 tatattttactagtaaaactatggtaattatatatgtaacaggaaagagaatagtgtgcctccaccgggagtcgaaccctcgatctccaggttactaagtcggac

16486 actctaccaatgagctaaagggaaattcttcctagctcgaagctagaaggtgacctaaatcctatgtacaattaaaacctgccacaattttaacatgtgcatacc

16591 tgagaattatcagctaaattatttgtttacatggttccagcttggtgtgaaggttacagcatgcctggagcaatggactgtgtcaattaattctcagtgtctgta

16696 taatatgttacaagaatgcagagtgcaaactgataattaattactggccatggcagcaaccagatcctagcccctgaattttattttataacttcgtaattaaaa

16801 tactcacgaaactcagcagctttcccttcagacatggtatgaaacattttatttgacatgaaaatgagtgatgattgtaaattattcagacacatgttttcacca

16906 aaaatattcatgtttgcaattaatttttagtctaattgccaaaagaacaagtgggtgcatgctattccgcactgcttatgtatggccatcatatatgttcagatt

17011 ttcctttaaaaactacttttccagagtagctgatgagattttgagaacttcctttagctggggcaaaggaaaccatacatggtgcacatgtataatatagaaatg

17116 ttatcatgtttataactattaaagtgggcaaaatacagcaaagaaccctgtagaaacggttgtggtaccgaaatgactcaattaaagaatcagctacgcttcctc

17221 aacacttggtcatagatgtctcaagtgtttgattatagatgtccaaacttaaattcaaagtgttggacataaagagcaatctcttcaagcggcagaaaaaggaag

17326 ccatctggatacgttgcagtgggccagttctaaatcgagacgagggggctaaaatactctcgcacatctatgaccaagtgtacatgttgagaaagcgtagctgaa

17431 ttgttgccgaaatcgacatcaattgtatggacaactcgtacacattttacaccttttgtgtacatacgcttcattggtcatttgtctgatgaaggtgacggggta

17536 catatcgtactagtcaccgaaacgtcacatagaaaaactttttggttgtgaaataagaacttcaaataacagtttctttgtaaattgcattcgtggtatcatttg

17641 taggggatttaattcgcaatatttgcagccacaaatgctggcaaaaactcaatatctattgaaggaaattaatttttaccattaattttactctcaatatatgaa

17746 tatgttgttaattatgtcttccctgtaaaatttctagctgcttcctatgtccagctagggagaacttctctttttctcatttggtagagtttaagtactgtaacc

17851 aagaggtcctaggtttgactcctagcagaggcattgaaaaataattacctctgtaactttggtacccatgtagggactcagaaaggagactcgacagaaatattc

17956 aacaatacttggaaagaaatcgctatgaccccttgaaaactttaagttctttcttagagggggcgtatgccacccttgtaaaatactagctgcttcctacgtctc

18061 agctagggagaatttctctagctaatttgatacagcataagacttgaaacaagaggttcctgcaggttcaactcctggcttaggcattgaaaaactatgacctct

18166 gttacaaattgttacaaataaacacagtgaactttgtttacccatttactgccaaataatgaaaataaaacaattcaattcaatgtcatattttactttattttc

18271 acaaaacacatgtaataaatttcatcaccaaagttcaaaagtacactcttttgagcttgatattttcacctagccatatcgcttgaaatgtattccttacaatat

18376 taaactgtattgtcttaaaatctttcatgtacaggttaaccttgatatctttccagatatgaatataacccaggctgacattgaatatttccgctctgccagcgc

1 M V N K A P V D I S T G S S Q Y E Y V K C V V V G D S G V G K T

18481 cacctacagtaATGGTAAACAAGGCGCCGGTCGACATTAGTACTGGGAGCAGTCAGTATGAGTACGTCAAGTGTGTCGTCGTCGGCGACTCTGGCGTCGGCAAGA

33 C L V R A W A C D T K Y R L E Q L V K T H V S T V W A T D H Y R N D K

18586 CTTGCTTGGTGCGCGCCTGGGCCTGCGACACTAAATACAGATTAGAACAGCTGGTCAAGACCCATGTGTCCACAGTTTGGGCCACTGACCACTACAGGAATGACA

68 E

18691 AGGAGgtcagttgatattggtgcatcggtcagtcagggcatccagttgacccttgagttttaccaatttcagaagtcgaatcaccaatttctcagaaatatgaga

18796 ggtcaaaacaaatttcaaatagattatcccacaaatctgaagagtcaaatctgattttggggagtcaaaaacatactttcaaggttccattagataccttgtcag

18901 tatacaatttctgctgaccgctctatatcttaaattaatcagtttataagtagtttcttgtaaagaaagggatgattcatacttaaacgtttaaatataagaatt

19006 ttttgttgtaattttatagtgcctgaactattttgtgacctagttatctttataatgatcataaatcataatgttttaagagtttttcgtcagtttgtcagttta

69 V L D R S W C H V D D C R V S L R L W D T F G Y H D K

19111 taaattcctttgcttttttcgtaaagGTTCTTGATAGATCCTGGTGCCATGTAGATGATTGCCGAGTTTCTCTACGATTATGGGATACATTTGGTTACCATGACA

96 D R G F A Y K G

19216 AAGACAGAGGATTTGCCTACAAAGGgtaaacagtcataaaaacatttgatgtgttgacgctctgatagcttgaaatttttgtacttctcaattacctgtagttcc

19321 atttattagctcacctgcccgaagggcttgtcagcttatgggatactgtggcgtccggcttccgttgtccatcgtccttccgtcaacttcttcattaaaatgacc

19426 ttcacctactttcaatgtcacaggggtcaattatgttaaaaacttaaacaatcttctcctcaagtttcaaaaggcccaaggtcatgatatttgatttgtaggtac

19531 ctcggatggagtgctttaatgtttcctcattaaaatgaccttgacctactttcaaattcaccaggatcgtatccgttaaagctgcaaacattgaaatcttagtat

19636 gcttttggttaagaatcatgatactttggtcttctttaacttgtagaccagctgtaattcatatttttcgcaacagtaatgtggttaaatgtcaacttcaaaatg

19741 ctagcaggagttataggtcaatgcaggttaaaaactacaaaattgaaaattgtgttttagtttccaaatttgatctgaagatacctactttacatatcaatttca

19846 tgtaacaaaagaaataagtgagcaattcaggcccccaatttttttttggggggtacatttggagccgaatataggcgccttaccctaaagaaattttgttatatt

19951 ttcccaatttcatgttcacattttcccaatcagaaaattaaaaaaaaataattccatgtttaatatcaggaaatgttgattgaaaaaactagtaaaatggccata

20056 taaacatcttatttgtttgaagctaaataaataaatgtaaaacaaatatctgttcatgctctttttctttttcaaaagaaagaacatcttttcctaaatggcgga

20161 aaataccgctgtattttcccaattgaaaaggtacaggcccctgtataaaaagggtaagaaaatcactgggccctctgggtctcttaattgtatataattaaaaaa

104 A D V V L L V F S V V K P N

20266 aatgaaaatgtaatttatcatctaggtagttgcttatattacccaagtatgtcacttttttcagGGCTGATGTTGTACTTCTGGTATTCAGTGTTGTAAAGCCAA

118 S L R N I L S K W Q T E I K N E C S T T P V V V A G T H A D M R F L Y

20371 ATTCACTGCGCAATATTCTTTCGAAATGGCAGACAGAAATAAAGAACGAGTGTTCCACGACCCCCGTTGTTGTGGCTGGGACTCATGCGGACATGCGTTTTCTGT

153 K D Q H Y R G M E K G L L Y K

20476 ACAAGGACCAGCATTACCGGGGCATGGAGAAGGGTTTGTTGTACAAgtaagtcatcttcatcatctaactcagaaatatttaacgttctgacccaatttggcagc

20581 aaagaggaaataatggcataacaaattttgaccttgatttagcctcatcttcaccaaactgggttaggttgagcaattttgataaaagtttaaacccagttttta

20686 tggttcattctgtaacaggttaaatccatccatttgattggttgtgtttgatattaaagtcattttcccattctatcacatcttaaagaaatgtctgtgtacttt

20791 tttgcttgaaatatacatcattttgttactagctgttatattaacgccgtgtgagccgaaggctcatggtgaccttttgtaagtcttggtatggccttttataaa

20896 taagatctttgacaagtttgaaaattggaccaattcaatcgctgtgtacagagttattgccctttgtatttgtactgtcattggacattggagggtaaaggagag

21001 tcgtattgagcacccttggctggcaaagttggtactcctatgaccagcatgatagtcgaccattgtgagcatattctgtacaattataaaaattaacatgattct

21106 ttgtagagcagtcttatacatattgacataaatatgagatatttaaataagaagctataggtttgcatgaataaatgtttaaaaactacagtatcgtcaatgggg

168 A I D K G D I I T P D Q G R E V A Q M I G A

21211 aactgtacatttaaccatggaaacaatatgtgtttcagGGCAATAGATAAAGGTGACATCATTACCCCAGACCAAGGACGTGAGGTGGCGCAGATGATTGGTGCT

190 P Y Y E T S I L F N Y G I E D V F F N V V R A A M V E R R K I K F W N

21316 CCGTACTACGAGACCAGCATCCTATTCAATTACGGCATCGAGGATGTCTTCTTCAATGTAGTGAGAGCTGCCATGGTCGAGAGACGGAAAATAAAATTTTGGAAC

225 A Q L R R I Q Y P L I Q S P L P V P Q P L F P T V T V A A S T F D F D

21421 GCACAGCTACGTCGTATACAGTATCCCTTGATCCAGTCACCTCTGCCTGTTCCTCAGCCTTTGTTTCCAACAGTCACTGTGGCAGCATCAACATTTGATTTTGAC

260 L A Q L L K N Q N D G D I I F N V R G V H I R A H K I C L V I A S E L

21526 CTAGCACAGTTGTTAAAAAATCAAAATGACGGTGATATCATCTTTAATGTCCGCGGCGTACATATCCGCGCACACAAAATCTGTTTGGTTATTGCTTCCGAGCTT

295 F R E I L L M D I K E Q E G C S D P Q T N G R T E K R T N K E D E Q V

21631 TTCCGGGAAATTCTCTTGATGGACATAAAAGAACAAGAAGGCTGCAGTGATCCTCAGACAAATGGAAGGACAGAGAAAAGAACTAACAAAGAGGATGAACAAGTG

330 L L D N E D I L E E S P I I D A N S N C S D D R L V R H G G R D I I S

21736 CTTTTGGATAATGAAGATATTCTTGAAGAATCTCCAATCATTGATGCGAATTCGAACTGTTCGGACGACCGTTTGGTACGGCATGGTGGACGAGACATCATAAGC

365 A R Y L N H A A F E K I E T V S N K N S T G E G V E Q T V V T V T Q E

21841 GCAAGATATTTAAACCATGCAGCATTTGAAAAAATAGAAACTGTAAGTAACAAGAACTCGACAGGTGAAGGTGTCGAGCAGACGGTTGTCACGGTTACGCAGGAA

400 I T P Q A F Q C V L E Y M Y T G R V R E E Y S Q L L E V Q Q A A E L L

21946 ATCACGCCGCAGGCTTTTCAGTGCGTCCTAGAGTACATGTATACTGGACGAGTGCGAGAAGAATACAGTCAGCTATTGGAAGTACAACAAGCTGCAGAGCTTCTC

435 K L F P M L V A L S N L Q T Q E T Y L N L G L E K R F H T D R V D K L

22051 AAGTTGTTTCCAATGCTGGTGGCTCTGTCTAACTTACAGACCCAGGAGACATACCTCAATCTGGGTCTGGAGAAACGCTTCCACACCGACAGAGTTGATAAGCTT

470 Q E L I L E Q G L L N D

22156 CAAGAGTTGATTCTTGAACAGGGGCTCCTAAATGgtaagttgagttcccataggggctcctagagggtaagttgagttcccataggggctcctagagggtaagtt

22261 gagtccccatagaggctcctagagggtaagttgtgtgaagtccccataggggctcctagagggtacatgtaagttgagttcccacaggggctcctaaagggtaag

22366 ttgagtttccacaggggttcctaaagggtaagttgagttctcataggggctcctaaagattaagtcattttgttagctcacctgcccgaagggcaagtgagctta

22471 tgccataccgcagcgtccgtcgtccgtccgtcaacttttcctttaaaacgctactagtcctgaatggactagcagattttgaccaaatttnnnnnnnnnnnnnnn

22576 nnnnnnnnnnnnnnnnntttagactttgacattataatttatacctgtgttttacagacatccactttgaggtagatgacggagtggtcggcgcccacaaacccc

22681 tgctcatcgcgcgatgtgagatgatgtgtgctatgtttacagacaatttcctggaggcttccgctcatgtggtttgttttaattccttacttccactttaagacc

22786 ttttcatgctaacattcttaaaccctttaccatagcttacaacaaactgttgattgtttgtcataatcataatgttaaggctgattggttgagacatctgacatt

22891 ctgctactcctgttgtctggtactcgacctgttggttgaggcatctgacattctgctattcctgttgtcagctactcgacctgttggttgagccatctgacattc

22996 tgctactcctgttgtctggtactcgacctgttggttgagacatctgacattctgctactcctgttgtcaggtactcgacctcttggttgaggcatcggacattct

23101 gctacttctgttgtctggtactcgacctgttggttgaggcgtctgacattctgctacttctgttgtatggtactcgacctgttggttgaggcatctgacattcag

482 I H F E V D D G

23206 ctactcctgttgtcagctactcgacctgttggttaagacatctgacattctgctactcctgttgacctgtgttttacagACATCCACTTTGAGGTAGATGACGGA

490 V V G A H K P L L I A R C E M M C A M F T D N F L E A S A H V

23311 GTGGTCGGCGCCCACAAACCCCTGCTCATCGCGCGATGTGAGATGATGTGTGCTATGTTTACAGACAATTTCCTGGAGGCTTCCGCTCATGTGgtttgttttaat

23416 tccttacttccactttaagaccatttcatgctaacattcttaaactctttaccatagcttacaacaaactgttgattgtttgtcataatcaaaatgttaaggctg

23521 attggttgagacatctgacattctgctattcctgttgtcaggtactcgacctcttggttgaggcatcggacattctgctattcctgttgtcagctacttgacctg

23626 ttggttgagccatctgacattctgctcctcctgttgtcagctacatcgacctgttggttgagccatctgacattctgctagtcctgttgtctggtactcgacctg

23731 tgggttgaggcatctgacattcagctactcctggtgtcagctactcaacctgttggatgagccatctgacattctgctattcctgttgtcaggtactcaacctgt

23836 tggttgaggtgtctgacattctgctacttctgttgtatggtactcggtactcgacctgttgggtaagacatctgacattctgatactcctgttgtcagctactca

23941 acctgttggttgagccatctgacattctgctactcctgttgtcaggcttgacctgttggttaattgagacatctgacattctgctactcctgttgtcagctactc

24046 aacctgttggttgaggtgtctgacattctgcttctccaaatcaccaaatgcaatctcacacttctataacccaaactactgtatatctatttttagatgggaatt

521 I

24151 tcctaactacccaactactgtataattatttttagacaggaattacttaactacccaactactgtatatctagttttagatgggaattccctttctgtttatagA

522 P L P D V T C E V F G V L Q E Y L Y T D K I Q S L D S V D Q L A L I A

24256 TTCCTCTACCTGATGTGACCTGTGAGGTTTTTGGTGTCCTACAAGAGTACCTATACACAGACAAGATACAGAGTCTGGACTCTGTGGACCAGCTGGCACTGATTG

557 V A N R L C L P R L I S L V E D Y V V M E L S R A A R C D E D I L E E

24361 CTGTGGCCAACCGTCTCTGTCTGCCCCGACTTATCTCCCTGGTGGAGGACTATGTTGTGATGGAGCTAAGCCGAGCTGCTCGATGTGACGAAGACATTTTGGAGG

592 V L M L I E P S Q

24466 AAGTGCTCATGTTGATTGAGCCCTCCCAGgtaattaaggatcaatagaatctcatcctaccttgagggtgtgactaatagaatcttcccctatcttgagggtgtg

24571 acagaaaaatcaccaacccgaggggtgatattcttggttgtctaacccgaggctatgctgagagttagacaaccaagaatttcacccagagaatactggaatctt

24676 ccactactgtagggtgtgatgaagaaatctccaactgaaggaataattatcttgattgccctgattgcctgtttaactgatacttggtctaggaatgatcaacat

24781 attgtcctgaagaaacataatgttgtacttaattatttttctcatattggtagggatgcttgacatcattatctcctcttcatattttgtcagcaaaataaatac

24886 tgtaagactcagatgacttttatttggtctgtgggttgcttgttagttgtttctctctcatcctattttgatcacaaatctgctgagaagtggtaattacatgaa

24991 ataaatgatacttctgaagtgcatgtatagaaaccatcccaacaacacaacagtggtcaagagaggtcaagagaggtcaacagtggtcaacataccaagtctact

25096 gtttccttataagatattttactgacaagttctcatctagttctgcatatttaacagctaagtcaaaatgatattgaaagtttctgtgcatatataattggctat

601 F H N A L Q L A A W C Q H Y V C V H Y R E A S K R F F R E L

25201 tttgttttacccatagTTCCACAATGCACTCCAGTTAGCAGCCTGGTGTCAGCATTACGTATGTGTCCACTACCGTGAAGCCAGCAAAAGGTTTTTCCGTGAGTT

631 R S L Q K E N L A L I E E N Q W P P V W

25306 ACGTAGCCTACAGAAGGAGAACTTGGCGTTGATTGAGGAGAACCAGTGGCCTCCAGTGTGgtaaatatctaaatattagggctgacaaaggtataccggtcaatt

25411 atgtcccctcacatcaaaagtttgtggagagataagattcggccaattatgtatgaacgtatgatacatttttgttagtcgttctaacctcttcattcctctaca

25516 gaatttcacaaaatttcacacatttgctaagcattacaaaacattgaagcactttgtatgtcatgttttcaatattgaaagccaagttactgttactatttttag

25621 agagaaaataatgttgcgtctcttgcgacctcattttagcccaatgtgtgagggtatctgtatcactggtgatgccttgttagttataaaatttaaaaactgaat

25726 agtttagatataattaattaaacaaatattttctcaattatgaatataaagaataaaagaaatgattgtcgtttaatgattttctttaactgatcatgtgctaca

651 Y I K E K E R Y D Q L M G Q K T P S L H I Q Q K A Q F S R W Q Q C K G

25831 gGTACATAAAGGAGAAAGAACGATATGACCAGCTGATGGGACAAAAGACTCCCAGTCTTCACATCCAGCAGAAGGCTCAGTTTTCTCGCTGGCAGCAGTGTAAAG

686 S C F C F C R R S K V L V E E E N Y D F P M

25936 GATCCTGCTTCTGCTTCTGTCGTCGGAGCAAGGTGTTAGTGGAGGAAGAAAATTACGACTTCCCCATGtgaaaactgctgacaggggagataattcagggctttc

26041 agttaactttacccttaatcctttcacacctatgtaacttaaatagactctgccatgcttttgatttggaagagtctattattgtctacagtggtgaaagggtta

26146 aaccttatcactagttacataaacactataaccatcacaaaatgtttaaatactcaaatttatccacttttgatgatattatacgccaaggaaatgcaacaagcc

26251 agaaagaaaccattggaaatttgaacccttcttcaggtgagctccctgtagttatagaacgtcactgtatatcaatacccctccatagtatgacaaagcctgctc

26356 ctcaaagacaatgtcacatgctgttgtgcgagtaagattgttagaaaatgtctgtgatagtgctactcgtctgtaataaaagtggttagaaactttaagtcagaa

26461 actttaagtcttacattaaagaggtcaaatgacctggctaactttgtaattcctgacaggtttctgttaaaaagaaaaaaaatgttgatgtgtcataacaatctg

26566 gtcaaataacagaggaaacctgtagaatattgatttatagcgtgaaacttgattctgcgatatcgttatgatgtatgtgaatttagacatataaaatcatgtgat

26671 gtaaagtctttgatgtgtttcatactgaagttgtgataaaatatgtatgatatatacctgtatcattataacaattgtgacccttattgtgatcaacttgtttgc

26776 ctctccatccttgacgtcgccttgttttgtattaccatagcaacagttgtttccacttgtctgtcccttgtgatattgtcaacacgcacaatttgaaaaaaatat

26881 gtcgtaaatgtcatggaaatgttaaggtttatgtccaaatgttatttcattcttcaattccatgtgtaattagtgtgtgatgtttcaatgactagtacgatatgt

26986 actctgtcaccttcatcagacaaatgaccaatgatgcacatgcacactaaggtgttaaagtgtacgagatgaccataaaatcaatggtcgcttcgtctacatcat

27091 aagcatagtgtgcgaaacgaccaatgtaaaagaatgtgtaaacgaatcttaagacgatttggtcacatttaagtgtagtacatacaggtgttatgatgtacgaga

27196 tgacagctacgctttgtttagtacttggtcatagatgtgcgagagtctatgggccccctcgtctctattcaacattggcccactacagcatatccagaaggcttc

27301 ctttattatatcataccagtcatggaaacatcacacattaatttgttattggttgtaaaataagaactaattttcacatgctacttaccaacctgaagaaaatgt

27406 ttcaagatattgtcaattccgtgttgaaatggataggataaaaatgtggattatttcacaaattttgattgtcaagagaaatatttatagcattgacaattacaa

27511 ttcggacattaacctttgactttttcatgattctagggtgaggtcttgtcattgacccctgaagacacatttgaactcttccagttcaaaggttggaatagttca

27616 tattgacacttgaggggtaaatgagttaattataactacaacttattctacatctagagtagatatttttgtaactattcattagcatgatgtaatctattggtt

27721 aatcatgtttgaatcagtttcgttgaaaatgttaaacacataatttgctatataacatgaaaatatgtatagatatattttaaaattgttatttgataaatcatg

27826 ttaattaatgaatcaaaggttgatataggcccagtgccccatttaagattatttggttgttattgggaaaatgtatacagtggaacttcgttaactcgaactcga

27931 cggaacccacagaaaacttcgagtttacagagtgctcgaagtatttattactaagataaagagataatcttaccaggactgtcaaattacttttgagttaagtgg

28036 ggtattcagagttcgacttaccggtaatgaagttctactgtatttctctgtaaaataaccctagttttgtgcctgtgaatcgtaatacatgaagataaacaagtc

28141 atgtttccatgactactatgataaaaccttgttatactgccaccacattgccagacacttttgtcagtataaagaggttgattgtaaattgagggaaatgcatag

28246 agaaattcttaaatcaagaaagaggaataggaaaaaatctcggcaccaaacatggaggaaaagggcaaatatcaaggttttactacagtttaagaatgatatatt

28351 ctagatattatattcatcctgtaaaaatatttatccactttatatttaaaaacaaaatgtgtatgaagacagaaaatgtcataatgtatgtaaatgtttgtcttc

28456 tgaggtagtctagctggtgtgattatatactcgtctgtgatattttgtgttaaataaggaattatagtcaaattgtctctcaaattactcaatgttgattttaat

28561 gtacaaataaatcaaactactgcaatataatgctaaaagattttgaacaaaatctgtcaaaataaaagctgtattttgtattatatattgtaaaatttcaaatcc

28666 agtatgcatttgaaaatagctttcatgtgaggttatatttcatataaagttattgctgtcagtctcagtatacattaatctaggagaatcgactcacccggggat

28771 ggagggaggatggggatttgggggctttgtttacttattaaattatacactatatacttagttgcttagtgtacatagttatatacctatcctatatttttcttg

28876 gcttacctttgctttactatatatagtactggtattgtttttagctcaccttctctaaggcaagtgagcttatgccgtactgcagcatctgttgtcaatcgtcaa

28981 tccatcaacttttctcttttaaacgacttctcatggagaaccaagagacctagggtcatgatatttggccagtaggttcctgggatggagtgctataatatttgt

29086 cattaagatgaggttgacctacttccaaggtcacaggggtcaaatatattaaaaactttaacaatcttcttcttaagttctataaggctcagggtcatgatattt

29191 ggccagtaggtacctgggatggagtgctataacgtttgtcattaaaatgacgttgacctaattttttggtcacaggggtcagatatgtaaaaaaaaattaacaat

29296 cttcttctcaggttccgtaagaaccaagaatattatatttagtaccatggatagagtgttatgatattttcttttcaaagtgaccttgacctagttttaatgtga

29401 caagggtctcataccttaaagcttcaaacaatgatatcctaagatgcatttggactagagtcatgatact

**CfCdc42**

1 ccacaccagacatctatctgtcaaaatgtccaagtctggtgtcagggccagaggaaaatcgagttgatacagctttgaacagtgcgatccttatatacatgtata

106 tagccattctatgggttttgttttgaaacgttgatgatgtaacagttgctgggtaaggaaacgcttgctaaattagttctgaccaatacatgaatcacattgcat

211 cagctttcaattaaattgtatttgcacttgtcgtcaataatgaaatgcaatctattttctgtgtgcgctatctccaaactccgatatccgtccgagtcagtgtgc

316 aaagagagacaactcctggttttttttatgaaatacaatgtcgatgtatatatccgtacatctgtaaatgttactttgtatatatacacagacgaataatcggaa

421 ttaaagcttatagtattcgggtttttttttttttactatttgaaaattgtataacaaccgtgaaaatggcaattccttgaaatgtgaacttaatatgcactacca

526 ttatttcagtaacaaaataagtttccatcgacttcaaccattacatagcctgaatgaaaagatataaatatgtataaaacaagaaccaaaatcaattatccaatt

631 gttgtaatgaagtttattgcaaaaaaatatatatacacatacataaaaataatgaagataaatgaatgacaacattaataaaaaagaaaacagagagatagatca

736 tttttatttccccatttgcatgagattacaacgaaattaacttaaaagacaatttttatcagttgtacaagctctcaaaaatgttttaatattttagtccagaac

841 aagcgaaaattatagcgattttttttttgctcgacatggtctagggaaaaatgagagttggagatctttttttttattattattttcatgtttcattttttccct

946 tttttatacagataaatgacatacaataaataagttaaataacaattttaaatttagatatgaatatatgtatttccattttgcatgcacaatcaaataaatagc

1051 ctctcgatcagtctcaagtgtgttcagattcatcagtagagataagataatatcaattttcactcgatggacgttctgtaaacaaatatcagaaactttaaatat

1156 aacgctattgaccgacatttatcttacacacaaagacattcacacttgtacgggttagacgcaatatcacttagacatgtataaggtaaacaatacatatatcga

1261 gatttcatcagcatgaaaatcattggacacattgttaagacagaacatatccagttttgaaataaaacagatttcattgtggtaaagttaatagacttacaaaat

1366 tcgtatcgggaaatggtggcaacttgctccgttacgtacatcatttggataaatacaaataagtttctgtatttctgaatctaattactcacagaaccttgaacg

1471 aagcagggaaagaaagcgaaatgttgccatagatgatagtaacattctataaacatatctgtgaaaagattaacacgttggttttttttttcatttccatgtgca

1576 acttgcgtatacatcattatctatttagcagacatcatccattatgaacaaaagtccaacaaacatagcttacagacattcatcatcatcatcatcatcatcatt

1681 tgaatcatacaaggctctactttcagtaccgtgggggaaaaaaaaaacaatgtaaacgatgttaggtcacatatatatatatttacatttccactgcacagagcc

1786 tatatatttctgtataacatacactgggccattatgacgtcgatacgcttatgacgtcataaatgtaacgttggcgatggcactgcttcaccgtcaactggtggc

1891 tgttctctgtctggatttaccctgacgttaaaacgtattaagaccaataaatgtctttatttctgcatcaactgcgtgttgctttatacaaaatgtaaatagaag

1996 tattgtttgtcatatttttgacgtttattggtgtatagcgcgcaccattcaatatgtaggtaagccaatgtatgttatacaccaataaacgtcaaacatatgaca

2101 aacaatcctttaatatgttatactgtctagatctacatgaaacggctcttttggttaaaaaaaataataaagtgacccctactgtaaccaccgactttgttaaca

2206 attccattctataacattttcatctctagtcgtaatcaaattcagtcaaacacgcataaaaatataaacagagacgcgaataacatatattacatttacatctgt

2311 gccccacaggtacttggggtcaggacctaaatttataatcaaatggtctaattttaattagattacagttgatccgatattcaagggcttgctcccaatatcacg

2416 attttcgtgcaagatgtcggctactgacagacaaactcacaacacagggttttgagagatgcagattgatgaaaattatggggaagttttatggtcgtcatagtg

2521 atctcattgggaaatattctgtttcgttatgtaatatgaagtcggacgtgtttgatgcatgttagtatacctgttggtaccgttgttcaccgtgttctcatttgg

2626 gaacgtgacaatactttaccggattgactaagatttacatggcgggtgtcgtcgatgaagcagaagacgcttactcttccggaacacctggtcttattctcttgt

2731 actttttcaggagtctccgtgtatatctttcttttgttcctattttgccttgattttatcgactttgagtttagattatgattcggttgaaatgttggtattttc

2836 ttttttgtttttttaatttgtaacatcacttcttgtctatttatgcacatatacaacactgaaatcgatttacaagcgcttttaataaaacacccgtttctggtt

2941 gttatactgatgatagtctatattattttaagaagcattcaatctataattcgaaatgatgtatttgtattctataataaaacgttattgtcctaatataacaat

3046 cataaacggatgttttattaaaagcgttgtatatgtgcataaataaacaaaaagtgatgttacaaattaaaattagaccatttgattataaatttaggtccttac

3151 cccaagtacctgtgctgtgccctaaataaaagacaaaactatcagtagaatgtatgttacctataacgtttatcgcttaggacaataacgttttattatagaata

3256 caaatacatcatttcgaattatagattgaatgcttcttaaaataaaccgacctagtgtatccaaaaatagaacactggagattactataaaatgtgacgatcata

1 M R S

3361 caactctcctctcattctgacgaaggaatgagtgtagggttgcttccggagcgatttatatacgactactaaaaataacttattacatcagttcgcgcATGCGCA

4 Q S F P L E I R A S L I K H P G N R E P E R

3466 GTCAGTCATTTCCGTTGGAGATTCGAGCCAGCTTGATAAAACACCCCGGAAATCGAGAACCAGAAAGgtacaaataggctaaaatgtgtaatatatatgttgcat

3571 ttcacttcatatgattattttggaaatcattgaagatgtttgtataaatgaagggcttatacatgatgtaaatgcgacactgttctcaaacaaacatgagccaag

3676 ccccttatcaactgacatcgagaaaatggcggacatgatatcgtccgactacctactgacattacgttcagaataatgttattttaatgcttcctttacacgtgt

3781 gcaggcctaaacgcatttgttattgtacctgtttgtaaagttaaacaataattatcatatttaatatacccgtggaagataaataagtcggtaaagtcatgataa

3886 tatcgaatttaatttatttagcctcacccatttatttgagggaaaatgtgtatgtagtaggcacctaaattttaattgatcagtcggtttcataaagttgttgtt

3991 ggttgtttgctttacacgaggaagtaaggatccaagaaatgttagtcgccgaaaatgttcagatttgtgatatgagatactcgaagtgacaaagctcctctgaag

4096 atttagtttgtactgattggtctaactataaaccttatctatgtattgaacctcagctttactttgaccttaactgaaaagaattcgaagccaacaaaaggtaag

26 P H I K G P G Q V I M Q T I K C V V V G D G A V G

4201 agttaattttgtaatttgtttttttttcagACCTCATATCAAGGGACCTGGTCAAGTCATCATGCAAACAATCAAATGTGTGGTTGTTGGTGATGGAGCGGTGGG

51 K T C L L I S Y T T N K F P S E Y V P T

4306 TAAAACCTGTCTGCTCATCTCCTACACAACAAACAAGTTCCCCTCCGAATATGTCCCTACAgtaagtatacatattgttgtttagttaacatcagataactaaaa

4411 agcatgaaagacaaggtgaagcccaccccccctcccaaaaaaaaagaagggggggggctgggctaaacttaacctgcaaaagttattacaaatagcagtccattt

4516 atagcattaagtcttttgagtggaagacagacattgacagctagcaatatatagtgtcttgtgatattgtatatatatttagattatctttgattaagatgcagc

4621 ctggatagaaatgtgcttcctgttgaacctgggtaaatcataagtattttatcttttaatcatgaatttgtttttgtgacatgtgaacttggagcactgctttat

4726 cttctgtagaatttatatatgcgaggaaagaatctttccttccccagacaatcagtgaagatactgccaccagaaattccatcagcagacaccctatcctccttc

4831 aaataagcaatcttatatatccttcaccttttctgtttctttccattttatgccacaaccttcatatgacaaaatcttcaatcagttcaaggtcattatttggta

71 V F D

4936 gaagtagaggaatgatagaatttctaatgtaattcttttctgatattttattttcttcctttcagtatgttaatcaaaatatgttttcactttacagGTTTTTGA

74 N Y A V T V M I G G E P Y T L G L F D T A G Q E D Y D R L R P L S Y P

5041 TAACTATGCAGTGACAGTAATGATTGGTGGGGAACCCTACACCCTGGGTCTCTTTGATACAGCCGGACAAGAGGATTACGACAGACTAAGACCCCTGAGTTACCC

109 Q T D V F L V C F S V V S P S S F E N V K E K

5146 ACAAACAGATGTGTTTCTAGTGTGTTTTTCAGTAGTTTCTCCTTCATCATTTGAAAATGTGAAAGAAAAGgtaatgatatgtcaaaatgcatatcacacatacag

5251 tgtattatatttaattctgtagaaaactgtttgagacagaagaacaataagtgtctctaatgtaatgtatgaactggtcccggctacacagtgaaatataatatg

5356 atcagaaactgcataaaaagctaaattgttgtagactagatattgattttgattatttcaaaaggtttctgatgcttgttcattatatctgatatgatattgaga

5461 aattaataaacatctgttatgtgtttgattcttggtattaaattttaatttaaaagttgtaacagtcaatcataattttaaatggttgatatgatacttgagcag

5566 gtccttgaaaaaaaatggttatggaattagaaagaaatatatgacacctttttataatactagctgctttctatggagaatgtctcattcaatagagtgtagact

5671 agtaaccatgaggtcctgggttcgactcctagcagaggcattgaaaatatacaaataacatcatatttgtaggttatgctgtaacatcattttaaacatctttga

5776 agttttaaggatataattttctctgatggattactgttaaattttctggtgttttcttttatagtgataagtgtacttaacttggttcatgttggactttctaaa

5881 ttaatgttttgaatagagctatgcttttgttcatatatgccaaaaactttcgtacatgatgcagatgttatttacaggtgaatttagttacacctgtagtatatt

5986 gatttaggttttatagtcattaaagctgaatctatttacagtaaattcgtagataactcttagctgatcattgtaaggattggtttcccaggaaaacgaaatatt

132 W V P E I T H H C

6091 cataaatgtaacaaccttgtatttttgtaaaaaaaataatgtaatctgtaatattttattaatctctttcattcacagTGGGTTCCCGAAATAACACATCATTGT

141 Q K T P F L L V G T Q L D L R D D A T T I E K L A K N K Q K P I T I E

6196 CAGAAAACTCCATTCTTATTGGTTGGAACACAATTAGATTTAAGAGATGATGCCACAACTATAGAAAAATTAGCCAAAAATAAACAGAAGCCTATTACAATCGAA

176 Q G E K L S R E L K A V K Y V E C S A L T Q

6301 CAAGGAGAGAAACTGTCCCGAGAACTAAAAGCTGTGAAATATGTGGAATGTTCTGCCTTAACACAGgtgagaaaaagttcagctgactatatatgtttgttataa

6406 gtgtgtcaaattacaaggttcccattcatgtagaaatgtgtatgatttgcacatatttcaaatatatcatgaacagattaaaacagtcctttgtaatctgcacgc

6511 tcaaaaaattgtgagtctcacatttaaaatatcctcaaagtcatttggtattccatctcgctgtaatgtaatttacaaaaggtgaaaaacctgtccaaaagtcaa

6616 aaaccaaagttgcagggttttcccatttggttaggaggttggtcatgaaaacacttcaataagtaaattgttatgacccactcaaaatgttgtaacaaaccacac

6721 tgcatgttttctttacagtctgtttgaactctgatgttacaatatatcaaacagatggcattgattaatgaaatgaaattgacactcataatatttgccactgta

6826 aaatatatttaaacgatttcaaccaaatggcatgaggagttaaattgattaaggctagataatcagtcgaaaaacaccagtcatcggtgtgtttaaagcactctt

6931 ttgtaggaaagaatttagatattccacaggtttcatatcttgttgttagctataccctattaggaacttaaattttgacaacaatagtaatggatcttaatttca

7036 agtatttaacaaggttgcaatcattatctattttttttaatagcattagaatcaacctttgacagggtttgatttattcaaataatgtttttaattgatgtgaaa

7141 taaattaaaaccaaaagagttaatagaaagatgctgttaggaaatacacatgtaatacattgatgtaaagaatcatagttttaaaaaaatattgttttaactctt

198 K G L K N V F D E A I L A A L E P P E P P K K K K C V L L *

7246 acagAAAGGACTAAAGAATGTGTTTGATGAGGCCATTCTAGCAGCACTTGAGCCCCCAGAGCCACCCAAGAAGAAGAAGTGTGTGCTGTTATAGacataatttat

7351 gacaaggaaattaattacactcaaaactaaactgacaaagacaactgcaagagctgggagcaacaggaaaagcaaacagaacaagcaccatgtcttagcatagtt

7456 ttaatacagtcaaattgtgtaatgataactcattctgtggctgaaattgacctgttcttcactgaaacattgatgcccttatgttctttgcaactattgcaagca

7561 aacatttaaatttcaataattaaagaaaaaaagttttttttctatttcgatttttgaaaatatgcaaaaagattttatttaaacaccagtaccgaaggattcaat

7666 atctctacataccattatagtcatggaattgttttcatcacatgtaacacagatttcagtcatgtgaactgtgaactgtgaactctacgtgtgtgaaccctttgg

7771 ttggaatctgtcactgctgtgttactatatatatactattaaaaagaattaacattttattaatagaatgccctatatatatataattatattctcgttaagaaa

7876 attctgtttgtattaaacacagctggccatgaattataagattggataaaaagtttatgtggaaatgtatttttaaacaagattaatattaaggattcatttaat

7981 attagaaattatctggttttgatttttgtctggaaatcactatatatctaatatctatccatatatgcatgtttaatacaggtatcacatattaaatgtgtagaa

8086 atgcatgtgtgtgaaaaactgtgctgaaaatatttaagataaagtattcggtgaccttttacctctaaatgttatctttaactctctcctaccactgtctctttg

8191 ttaccgtgacgatgtgacagtacctacacagtcttaccattgtgtgtatgattattggtatatgattcagatttgcctgcatataaaacaagtgctattacataa

8296 ggacggacacattagtcagtatatctccacattgtctaggatttgtctccggacctatttgttcacaaagaacaactggtttgtctttttttaaaggtaacactt

8401 tggtaatatgcaggccttgcatagttttgtcctgaaagcagtatcagataactaggtccgaccttatttcataaataacatcttgtccaagccgttctgttatga

8506 tagattgttataataaaataaataaaacatattctaaggacctgcttctagtcttattattaaaaatttcccattgaaatatatttcaaattatactattcaaag

8611 tgcaacacttttataccataatttagatactgcaaaacgatactgtaagtcctttttctctaaacactattcttaatatctagtaaaaaggagtatgaaatagtt

8716 ttttgcattacttgatacaagaaaagacagcggattatttctttcggtagaagataataaaaataattgagacatgaaaaaaatgaaatgcttataatttgatga

8821 ttatacacaagttatggagattttttaataaaaaaaactacactatcaaataattactcataggggtgttgctaaaataatagaagtccaattttgacaatatta

8926 tgttacttgtaagactttcctggtcatgtggagacaagtcttgttaatctgtcatttactgcatgtgtgtagaccaaagcttcctgtatgtgttgtgttgtgatc

9031 gtaggttgaaagaagtgtgcaagaagaaagtgtgtccatattatcatttagtatgtattactatgacaacaatacaaccctgaatcatactaagtttgcattggt

9136 ccataaagctacatacaaagtaaatggataattgtatttcatcgctcgatttgctctgagcatttatttctgagcatttttttttttactagcttgaatatgata

9241 ttgtcaaaaagttttgatcatacttcattatatttccgtttgtaaggaaacaatcacttctatttcactggagaaaacaatattttacatattttttatgaattt

9346 ttcgaccatagttatagagggaacataaaagttcatgtatcataattaggagtaaaagaatatatttgtgtatatacctatctaactttgttaagtgttcggaca

9451 tttctctactgcatttattgttttattaaaaggctttgggtgcaataattaaaaagattaacccctttgaattaataataccagtaagtgctaaagccatgaaac

9556 aactttcataaataaataacaaatgatacactgggatacagtgttcatattaatttgtttagtagtctacatattataaattggggtcaagcatacattcatgct

9661 atgttataaaattgttaaagatctgtaaaaaggtaaataatgagatttatgtatcacaaattattgtaatatattaacattttaaatctgaatattctggaatat

9766 ctaatatttacacttgtcaaaaaaatccccacccaaatatgttatatgcaggattgaaagctgtataattatattcctttgtaaatatctcattgtatttgtata

9871 gacttagatactgttctacgaggagtaaaagtcctttttcttcctgaaaacattcattatctgttgaaattactactttttttactaaggatttgaacatttggc

9976 catgtcatatactctgaaaagataattttggatcgtgaaagacaagaaaatggtgaaattttataatatgaggagttttggtctggttcattacagaacctatat

10081 tttcatgtaacatacacagaatgttctttgttcttgcttaaaagttcaggaaatgtatttcttttttcctaatacatgtatcactaactactgtattagttcttt

10186 agctataaatctactacatttgacaaaatccttcaccaaataaagacctgtgttttggtctgcaagaaatgtagaaaaaatagaactttggtagaagtaaataaa

10291 atgctgtaatacttcaaataaatgaatgacttggtccaattatcaatgactaatttggtttgatcacagtattcatagtcatcaacattttgttcccaaaatggt

10396 attatgttatatatgtttgtattgttcacacaataggaagtctattgtatatatgtcatggttagataatattattatgtgtatttagaaagcgacatattgtat

10501 atacctttctctgcctgttgctattatttaatgttactgcaataccgcttcgtcttcatatacagaaataaaaatactgagaagaacgttctgacaagctttccc

10606 tgagaaaacatgtacaaagcatgcattgcaaaaatcaatatcatcttgttcatgttttgacatcttgcttatttatgttcaatggaaagttaattttaattgtgc

10711 caaacttgtcctttcccaattatcttttgcttgaagaaactgtccatatttctgctaaatgcatttgcacctttttttctgttaaatatttctgccaagtcattg

10816 tttggttaaaaaattttagtgacccgtttgggtaaatattttccaaatggtattgtaatcatttttcttttgatatgaaatgcttaaaaaagtaagatttcttgg

10921 tagcaatgtcagtgtttaattatttttcatggcaacctaagaaatctcagaacttgcattttttttcctctcttttccataaaaataaagaatccactaagaaag

11026 cgaagcattgtgaacattgtactcgtacagatatgtataaaaaagtctgtggctatctgacaattaaaattgacactattaatggaaaaatttgtcatttctctt

11131 tttaagcaaccaggaacaagataagtgtcatctacgacgccattctttgtacattgttgtgttgtctatcaatttactgttaacaaagccagaaaaaaaatctgt

11236 tgggatttcatttaaaatataaagaggcaatgctggcctagtggttaaccttcaccactagccctccatcactgggttacaggtcataggcctatccagggcagt

11341 taccagatataaactgtcagtcggtgctttatatctgggaacagctttcctccaccaccaaaacctgactcatcctttaataaatgatcattgctgtaaataggt

11446 gtaaaacactaacaaacctaatgtaatcattacatttgaaaatcgagtaatttaattgtaattataatcacaaatatttaaagtaattgtaatgtaatcaattac

11551 tttacaatgtaattgaccacatatctggtccttttgtaaataactatccttgtgaaacatgatttatcaaggagaaacaagaaaccatgtttcttcccaacaagg

11656 gtattattagctcacctggtccataccgtggcgtccgtcgtccgtccgtccgtcaacaatttctttaaacgacttcttcttcctaaccacctatcagaattaaac

11761 taaatttggctggaagcatccctaggtggtggggattcaaaatcctacaaacgg

**CfMig**

1 accatattgtatctactacacatgtaccatattgtatctactacacatgtaccatattgtatctactacacatgtaccatattgtatctactacacatgtaccat

106 attgtatctactacacatgtaccatattgttactatggccctacgtgtgcctacatgtgaaatacataagggatcacactggcaaataggaaatacgataaaatc

211 aagcaattgtcatgtaacagtttttaactaccatgcgtaagaatagtttataattccccggttttcggcaataatgtgttgagacattattgcttatctatatta

316 caacatctaatttcttggcattgatcacgtgacataaataaaatgtcatgtgatctcacgcgctcgcgttcccaggtcataatttcacttggaacatctatagaa

421 aaattgcccagtcggggccttacatctgaaatcaaagtgactgtggcagcagacaacggcctgatcatattcactattatgatatatatttttttatttctgtat

526 tttattactatttgtggccacagaggtattaaaatatgactttttatttttgggaaattagttttggtttcacaaaagcgaaatatatgatatgtaatttaatac

631 gggaacgtcgctatgaacttttgagtgacattgtcttttcacttttaccagaaaagtgtatatattacgtctctgatgtatttcatcgacaaaacttctcggcag

736 tgacatttcaaagctttggttgtctttcgtcaacacacgaatattgcaaattttactacttaaactcgtcaatatagaatagacagttgacgtagaaaaaatatg

841 gaaatatgggctcagttgttagaagaatgatctgcttaatacacgttaacgatatttttcaactttttataatttcgttaatatacaagatagaactttataaat

946 tttacagatatatttactctaattaatactttatatctgaaagtttgcgacctcagctgattgtttcaacaacaaaaaaatgaaatttgcactgatcacagatta

1051 agctaatcgcactttgaacaactgggccatgataattaagatttcccgtctggaagatttcgaaggctcgcaaaaaaaacaacaaaaaaactcgtttcataaaat

1156 ctcatgggaaatgataccagaaagatacttttgatcacataactaagaaaacttgtattttcctatctcgaaacattttcttcatgttggtaatgttggtatata

1261 acataacatgtgaatacttgttcttatgtcacattgatttcatggtcatctcgtaaacttaaggtgacggacctggtcacagggagataactcttcattaggtct

1366 catagacgtttgtaatctatgttaaaaattgccaagcgtcaaagtgagacataatgtttatgtcagatgacaagaaaataaacataaaaaatcgaaacaagaatg

1471 gaagatgaactaaaccaattcataaacatgtttttggctttatttttcgatattcaaagttaaaagtaacataattatcaaaacaactttaaaattaagagaata

1576 tattactcataaatttcaataaattggagaaaaagaagggcagggtgcctttttaccaatttatctagaaaaatatattggatacccacatttttttaattttta

1681 gtttgtttgcctatacataattaatttgtaaccaatttttcatccaaattaaacgacacaattttttgaaatttctgttttaatatgagcaatatggtatatgat

1786 gggtcaaaattttcccgtttttccgcatatttccagctgtaaaatttgcatctaatgttctgtgtgcctcttacgcatttaaatgatacgacaacttacattaag

1891 caaaattatcagtgcttgaaagtaaataaatgaaaaaaatcattttgtaaagccatttttggcccacactcatgtttgcttgttattcggcaatctgccaaattt

1996 agctatttttgatactcagcagagaaaatactgtgtggtccatctcaaggaaattttcggaaaacatgtgtctcnnnnnnnnnnnnnnnnnnnnnnnnnnnnnnn

2101 nnnnnnnnnnnnnnnnnnnnnnnnnnnnnnnnnnnnnnnnnnnnnnnnnnnnnnnnnnnnttaaaaaaaaaaaaaaaaaaaaaaacccacaaagaaatcgaatta

2206 ttttctccttaaccctttttttttgtttgttgcaatttttgagttctagcatcacagcagtaagatatgtactcagaatattttagcatttttattgaggagagc

2311 atgatattaagttaaaatatattgttgtccgatccgtatttatatttaaaattaaaataggattaaacaaggaaatataattgtaattaattttcgctttttata

2416 acatcacaaatgtcacgacgtattcagactttgaactaaaggccaaatattagtgtgtgctatgtatgaaatgatgcattaagtgatgaaagtgaaatgtggtaa

2521 gacatgtgatcaaatcacgacaaatcaaatgtttagaagccttatttaactgaaaaaaaggctgatcaaagtgtacaccaaccattttaatgtttaaacgataat

2626 cttacttaatttcagtattacgtcattatatgccataaccagcaacaaataatttcatgaagtatgtgttaagaattgaatatagatgctataattcaattaatg

2731 cgtgaatttggccttcattgaatcgataagctttcatacaggcatttaaagttttttgaataaaaaaattgaaaatacggatgaatgaaaacccaataattaatt

2836 acaactttctatttactgatttatggatccaaagtggctaaacttagctgtatgtgatggtccacgtttcggaacacccgacctaatcagattataagatcaacg

2941 gtaacaccaatctttgtattttagtatattctacttccagagtaagagggttccaaagagggggactgaatctaaacccgcttctattcgtgacagtgtcagtaa

3046 aacatctaggccttactgctacagtactccactaggcctaggcatagctttttttgttctttttaatttttgcaaggatataataaattaaaatagttttgctat

3151 taatgtagtatttttatgaatgctcactcattattgtgtcacagtttgacatacgatatatctatttttggcctttaccatttttaactttaaaaataaaccctt

3256 agtcgtacgtaggcctagataacttatagatattttatctggacgcatcagcagccgcaacaccggaagttactttgggatccgcagaagccggcggagcaaata

3361 catcattttgattggacgctaaaaatagctagcattgatgaaataaaatggcagctggaaggtttggcttctgacacagtggaatcttatgaaaatcaaccatgt

3466 gctattctaagatgctttaatatgaatttgaaagtgtccaacatacattgttttaatcaggtaggtgaaaatccgttcgtatatgttgggagggcgatgagtaac

3571 catccgaagggttcaccaaggaggcgatgggttggtagaccgacttcggtgagctggctgatgtgtgcgagtcatcatgtcggaatggtgtctaagaaactaaag

3676 aaatgacatcagtgtcaatatacatatcacgcattaggccagactcgataacgtgtcatgtccctcctaatttttacgatagaaaatgtggcttcttttgtctcc

3781 gaggcggtgttgagtctacacaaagaaacgcttgttgaccagactgttcgagacaagtgtcacatatatcacaatttacatgttcaggatatttagtgaatttgt

3886 gttgcaaccagatgtttaaatgccggcaaacacttttagctaggtgtttattgtggcgacacccatatgtctatgacagaatacataactgacacatatgtacag

3991 tacacacgtctgttatttagtttactgtatgcgtgactcatatggtggttggtatcaccttgaccagcgtctctgcttcctcacatgttgataacgaacatgtaa

4096 ctcgacccatgatctgtgatgatttcgggtagggtatcactatcactggtgtcctaacaaaacaatctatctgtgaagtgtgatagttataaatatgtaagttgt

4201 cagttcaataacctcatgattgggaccccatttgaaataagccattcgttgctttaatgggttatcccaggtaaaagaaacgaaatatatgaacttttatgtttg

4306 tgtactttttttacccgaataaacattattattatctttttaatgatggaagaatttagtattttatatacaagaagtttttattttacctgtaacatgaaacat

4411 tagcactgaagagcttttcttatgaaagaaaattattatgcctattatttagggcttgatatagctgcttcataccgtctcggctaggtagattttctctttagc

4516 tcaattggtagagcgtaatattagtaaccaaggggtccagggttcaattcctagtggaggcattgaaataaaataatttataacctctattacatgttttattga

4621 tagcagaaacttaagtgggttttttggggtcgattggttgagtggttaactattccatgcggctttcctgggtttcgttgtttatggaatattcataaaaagtgt

4726 ttttctcctgtcctcacaactccaccaaaccaggtggggtataatatgatctggagttaatcattatgttatagtatttatcctgtacaattgactataaattaa

4831 ttgatgcagctgtttgaagcacagtcccactacaaacagaacacaacacatctccagcataaacagtattacacacatagtgagatattagccaggtgacatagg

4936 actagtagttagcacttgaaaaggcagctcttcaaactctcatttgtaattgtattgaatattgtaatatgcttgttatatctgtttattgtaaagtgctttaaa

5041 atggctttattctgactagatagggtgctgtatcgaatgttttaatgttttttatcatctagttcattctttttttcagatagtctgattttaattattggtcgg

5146 gttgcacaaagtgcaatccaggatataatatggtgaatatagggtgcgccgcaatgtgggcgggtggatggggaaatagggtacctgttttcaagtgttttgtag

5251 aatcaacttctcctacagtgtttgtcgtagaactgtgaaactttgtaggatgtttacatatatgttgaagatgtgcacctcatagagtcatattttggatttgtt

5356 ccgtttttttggtgtggattttctggacttggaaacctaagtcctttttcggttacctgtatctttgggaatgagggtacctgttttgtagaatcaacgcctcct

5461 acagtgtttgtcgtagaactgtgaaactttctaggatgtttacatatatgttgaagacgtgcacgtcatattttggatatgttccgttttgttgtgtggattttc

5566 tgaacttggaaacctaagtcctttttctgttatctttgggaatgagggtacctgttttgtcgaatcaactcctcctacagtttttgacgtagaaccgtgaaacct

5671 tgtaggatgtttacatatatgttgaagacgtgcacctcatattttggatatgttccgtttttttgtgtggattttctgaacttggaaacctaagtcctttttcgg

5776 ttatctttgggaatgagggtacctgttttgtacagtttttgacgtagaaccatgaaatataggatgtttacatatatgttgaagatgtgcacctcatattttgga

5881 tttgttccggttgtttttgtggattttccagacttggaaacttagtaatttttcggctatctttgggaatgagggtacctgttttgtagaatcaacgcctcctac

5986 agtttttgacgtagaaccgtgaaaccttgtaggatgtttacatatatgttgaagacgtgcacctcctcctcaactgatatttacaagtgttatgatattgttggg

6091 actctctgatattccgcagacagctttattcaatttaaatactgtcagacagtgtacatgtattaaacataatgagctgttcctcatcacaacactgtgttcgag

6196 catggtcattttacctgtgggctgcatgtgttgtattggtatgatatacctgtcacctgaccactatatatacactatacaaatcccaagacgaacaggattttt

6301 acagtaatattctagttagttggaaacataatatagagatctgtgtcaagataaaaaacaacacatctggggcacaaatcttgcatcttgaaaatagctgaatgt

6406 atttactgcatataatcttagttagcacctgtattcacatatattaatatccaaaaatgtatgtgtatttgggaacaattaactgtgatccctttcttaccattg

6511 atccttcccagtccccattccatattgcttatataccctctgctatactctgctggggctgcggtggccaagtggttaaggcgtctgacatctgctaccactagc

6616 cctccaccgctgggtcgcggatttgaaacctatgtggggcagtcgtcaggtactggccgtccgttggtgtgttttcttcgggaattccggctttcctccaccaac

6721 aaaacctggcacatccttaaatgaccttagctgttaataggacgtaaaacgaaaataacccaaccctaaaccctgctattcccctaccagtcatcttgagcaata

6826 ccaattatatcatacgagcaaagtcctattgtcacatgtaaatgaaggtccattgtattttcacccgtttctacatataccttattgatttggtgtagttattga

6931 tacatttgtatctgagtcatgcacatgcattgtcgcattaggattttattagatttaaatacaaggcttcgttaggctgtccaagaccatattatcataaaatag

7036 ttatgtatagatttgtagtgtatagatagtatatacatctgtatgtatagtggtattatggagcatgtgtcagtttgaaagaataacccaaacgtgggttgtaaa

7141 tattgttgtattttgggatgaagacttagatatatatatattagctaggaaggttaaggaatgctgaaactggacttgggcaaagtaaatgaggaccaggtccag

7246 tatatatatacttaaattgatctgtaacaggaaaatacatggtcagaccataaccaggattccagccatggaccctaattaaagcagatgctcaaaaccaattta

7351 gctaccttggcttataggtcaccagggagtaacacatgtattgtccagttctgttgcacatgtattgtccagttctgttgcacatgtatagtccagttctgttgc

7456 acatgtatgtattgtccagtcctgttgcacatatatatagtccagttctgttgcacatatatatatagtccagttctgctaaacattatacatttgtaactagta

7561 catgtatatatgtattcaaagtttattcagcgctgtatattcacacaaacttttttttcgcttcacaaatttagattatatttacatattacaacattaaacaat

7666 aatgtgtatgctgatgttattacatatcccttcggtaagtgtgtaaccgaaggcttatttaagtgaacaattcatctttaattcgcgatgtcaagtgacttaatt

7771 atataaacctgccatgtgtcttaattaggtaaacaaacatggatcagcctaattacattgaagctgaccagctagaatcttaaactttagattgtatgttatgaa

7876 ttagacccctggtttactgtatataagaggataaacatggtaaatataattaagtgatgatccaattgtcgatcaattataatcaacaattgatcgattgtagct

7981 aaaaaactgattaatcaattgcagttttgaataatcgattgtcgttgtgaaagtttgatttaacagaaaatgttttgtgtccttaatccagtttcaatgtacaaa

8086 tttatgttcttgtaattttagtgaaattattttcttttgctattgtttcacagttttgttatttcaaagttgattaaaaattgttcagttgaacagcagtagttg

8191 tttgtataatgttgatctgtattcatggtacaaattacaacatattattgtatcttttagcatatatttattttttaaaaaaaggcaaaaaaattgttttaaatg

8296 ttttaatcacttttcaaaactgaataacaatttgattttcctaataattgattggaagagcacattcgataattccaattaattgattgtcttattatatatata

8401 attccatccgattcccaacaccagtaattaatatattacctgtttcacaagcttggccatgcattgatgtgataacgttttgtcatcttcaacaattttgtcact

8506 ataatctgtaagttttaagatttaatttagatcagagttcacttgtctgaggcagtatatatatattatatgcttcatgcactatgtattttcagtgtacataca

8611 tgtatgaaaaagttgtgtggcttgctagcttctccaaccataatatggtgaatacctttcctatcagtgtttattttccatctattagctttgattttgtagtac

8716 actgttacaaaaatctaaaaaattttggtgccaagcttatatttacttaaggaatgcctatctagtgatgatatatgtataaaccatttaaaatgtgaaaatgca

8821 aaaaaaatgagatttaattaagttgatctagatactttctgtcttgaaattcccaaatgtgcattgtggcaaccatcaagaaattaacatatttctatctatgac

8926 gtcagaggtgacaaataattataatgcatgtaccgtattggtcgtaacagcatcaggtcattttataaagtggcatatgcatatacatgtatttgagtctttatt

9031 cagaaaaaaatgcaaacatgagaaagtacaatgtcattaaagagaaaaatcaatcacattcccagggaaaattccaaattggatgctcaatttcatgatcaggat

9136 gcatataccggtacatgtatatatgtatagtcattgtgtttcgtccatcgttgagccccgtccgccgtgggtaaacttttcacatttcaaacttaatcttcacaa

9241 gttccacctgtagcattgagttgaaacatacctgaaatgatccagagatgatcctgaccaagtggtagtgttattattttggatcgaccagccatgcaggcaacc

9346 atcttgaatttgaaaaatacattttaaacttcctctgtagttccactggtgccagcgagctggaaattggtggaaatgttaaggaaggggagccgataaagtgtt

9451 gttgtcgcctcaaaaaatctttgacatggctgtcaaagcggccattttgtaacatgattgcctaaccatggttttcctgaacaacacctatttcaaatttcttct

9556 caagttccactggtgggatgtagctctatcttaccagatataatccttagatggtcctgaccttgtttgttatgtttttggtcagccggaaatccaagatggttg

9661 ccaattgacagccatcttgaaaaaacacatctaaaacttcttttccagttccattggtgccagcgagctggaaaatggtgaaaatgtaaaggaagggaagccaac

9766 aaagtgttgtttttcggccttgtgaaaaactttgtcatggcagtcatggcagccattttgtaacatgatattgtgcaatggttttcttgcaattaaacaacactt

9871 acggtattttaaattctttctgatagcttggttgacaaataataacccactgctgaccagaattaccatcatagtaaacatattctgggaatatttcattgaaac

9976 tttaaaagttttccctttctcagtctttgaagtcagatgaccatttaggcccatggacctcgatcttgtttaaattgttacactgtgagactgtagattatacat

10081 gtaatatatacatatgatactattttgttaccacagatctatatggttataatggaaattttaggaacagagtgctcaaatcattccatgccgtgcgccagctag

10186 cttagcttacaagccccccaaaaaagaaaaggttgcgttcacttacaaggggagctacggtaatgtaataaaaatcctattaaaacaaacctcctcgtatcatca

10291 tttaaaggtcgatgggtttcataatgggagtgatctccagaggtcgaatatcagaaatgatgacaaatattttgatttgcagtcattgtgttttttattggcctg

10396 gtatctggtttcagtgatggctgatcatgttatgctgtgaaaggttgccacacggctatctgtaactataatacagtgatataccagtgtatactgtatactgtg

10501 agaggaagtcccaggcagtctgccagcctcctgtacagcactacaatccattcagataccctaaaaattacaggaacactgtatcttatataccatttagtaaat

10606 tttggacttttaatttttgaagaactcttctgaatggtttttagaacagtacaagaatgctattttcacagaattttttttggagtgtaggcatatacacaatca

10711 atataataaatgcaaagttaacaatctgaatgcacattgtttttaaaatcatgtatgttcaaattttaacattggtctagcagatttatttttgcaaggcgactc

10816 ttttcatagacattatttgatgcatcacagctgaacagtatggaatatgaacttcttttaacattcaatataatttaaagttatcacatcaggtaatttagaaga

10921 aattaaagaggcttaaaacattttccgttataagctgtacatacagtgtatatatatctgtacacatgtcctatgaagggaaagtggtcatatgcacagttgcct

11026 cgtacaatcatgcagtgagatacagcagcaggcctattattctgtaatacatgcatgtatcctgaaaattgaaaagaatcacagattaatatatatatatgtgta

11131 taagaaactgtttgtttaatctgtcgaaaattaaacaaaacaagatctaagaaaatgtggggcatcaaattcaattaaaaaaaagttaatttgtatacaaagaat

11236 acagcttaaacatggaagtgaccccccaacctacaaacaaagaacaacacatcagattaaaaaaattatttgactttttaacattttttttttcataatatattt

11341 tctgctataaagaggcttgagaaagcatggtctgatctacaatgtatgtaatcactagtaagtgtgataacactgaaaatgtgtgttggagtacaccctatagct

11446 attagatggtggagatggtacagtatgagattgtatttgtcgagattacatggtatttgtcatgtagtaagtgttaggaaaccatctgtgacccatgtctattgt

11551 tagggagtctggtgaaaaatggattcccctttgaagagtttgagacgatgattcacagcctggtagagttttgcctccccctcctctgactcctccaacacagca

11656 ctagttcttatcaaagtggtgtatttacacaggatagcatgactgtgtcagattacaaggggaagggctgtagctcagtggtaccttatggcatgtcagtccaac

11761 atacaacttttagatgtgtggaacattcacttagagacacttttgatttttttaaatatatcctgcgttattaagtacatgtatatactgaggggaggtcaacag

11866 ggaagaattaaaatttattacaacatatttctagttctttgtgtgtatactgagagtgtttagcttcaggaattttatttaactgaaatttaaacatctatataa

11971 gttaacgctagactggtgtctccatagctcaggatgcagaatgttgcgtcctggatgcatcattgtcaatcagtacacatgtatgaacactagtactgtatgatg

12076 catctgcggcctcatccgagctgcactaggtatcaccagttggtccaatggtggtgtttgggaagtcgagtgatcatggacatgaattcagatattgtgtagcta

12181 tgagctaatcaatgattagccaacaggtaattataaactgaccaagtccgaaagtacatatagtatatacacaaggtgcacatggtcaagctttcacataacaag

12286 atcacttgcatttgtttgtcagaataaagatagttcatgttgaatctattacttttgtatatatgctactcacaagtaaaaatacaatgcaggtaacattaggat

12391 tatatgttatgtcagattagtggtatgtttccttctgctttgtggttttatatatcacctacatgtaatataagtatatattactgatacatttgattaccagtg

12496 tatattcactcagggatactaattgtacatgggcacatctatattttgtggtattttattctagtccccaatatatagtgttgaccatgtaccggtacatcctat

12601 cgcgtcgcatcctgattaaatttttacaccaagcaacttcattctttgacagatttccatcaaactgtatacgtgtacatgtacatagtcaagtatgactatacc

12706 tttgacatgggtgtgtttcagggttcaaaggtcaaggtcacttgctaaatatagaaaatcagggatcaagtggcattgggggcatttatgtcttacagacatttt

12811 ctagtttattattaagtctcccaaacaaatagagactaattgttttcattctgtttctgattagctcacctggcacaaaatcttagtgcatggtgcggtatccat

12916 catagtcgaatacatgttgatttcccttcagtacatgctcacacataagtaccgaagcgaaatcaaccatgtgtatccaaccgtgggcatcaatcccagtccagt

13021 gtcaacattttgctttaaacaacttcttctgaattaccaagaatcggcaggctgatgatatttagcctgtagcatgctggcatgaaggcctaccaagttagtttg

13126 ttcaaatgaaagaccttcacaaatgcaaggtcacaggggggtcgaatatgtttaaactttaaaacaatttcctctacatagccattagccaagaagtttaaggta

13231 gtgatatttggccagtagcatcctgggatgaaggctatagggctaccaagtatgttaaaatgaacaactttagcatattttcaaggtcacaggggtcagatatgt

13336 tgaaatatttaaagaactttttctgaacaaccaaaagcttcagggtggtgaaattttgccagcatcatgaagggatggaaggcaaccaatgcaaatttgttcaat

13441 taaaggacctttgcccatattcaaggtcataggggtcaatagtcttaaaaacatttaaattactgaaatgattccgaataaccataacaaaatgggggaccatgg

13546 tgacaaatttgctgagttcattgcatctaattgaaaaattagatttaagccaagctaatatcttattaagtttattttttgaaaaaaaaggtgaattaaatcaaa

13651 ataattgaattgtggtacaaatattgcaatatgtatcaataaggattgtgacagctctactaaggtcataggggttaatgatcttaaaaaaccttcaaacatctt

13756 ctgatttactaaacgtctaagggtgttaatcttagacctgtaagtgccatgctgacattgaggcataggactgccaaatgtgttcaaatgaatgaccttgatctc

13861 atgacttatgttcaaggtcacgggggtcaaataagggctaccgattgcaagtttgttcagatgattgatgtttaaagattgaaagcatattatgcatggttcatg

13966 taatttgtagcctaagaaaaaatagataggtcttggaaaaagtaaaagtacaggtctaggacagatatctagctttacatttgtatgttgaatggtgattgacac

14071 cccttgagaaatttcaaacacagatgcggaaagggacaagtggagggaagatagctaacttaattttgtgaatattttatatgtttctgaccacatttgtcataa

14176 aattaactgaacattttgaatgttgattgacactcccttgagaaatttctgaatcccccccctcccccaactccattcaaaccctctctggaaccaggtaattaa

14281 tagttagatacggaggactaacaaaatcttgtctattcataattgtcgacccattaaccaggcccagctccactatctctgagggaaccagagcatcagtctctc

14386 atttaggatccagtaggagatttctatagcaggaccataacatctaatttactcccgctattatagaggtcaaaggtcaaggttataatttggggaatgttggtg

14491 ggtctttgtcagaaaagcaattactgtgttacattcatgtcaaaaacaataatgatgactatattttacatgaaatatgaaactgtatggtaatatatatacaag

14596 tcatgatcatgaataatgatgataggcaaatgagggcagcatacgtacatgaaccaccctgccagctttgagtggaagaaaccttatagtcaagactgacaaagt

14701 cagactcataaactcttacagtggtatagatattgataagttatattgatggatattaaatgataaagaattacttctgtttttgcatacactgtattagtcttc

14806 ttggtataacagatgatccccagatttctgcagacaatttttgtgttacccaccctaatataatggagcttatgccgaagactggcacagaatgatttttcctca

14911 tctatcttgcatatatatatattgatccctttcatttaattttcaagtatctacaattgcaaggctccaagaagcaaatacagcaggtcatgctaccaaaatctg

15016 agaaatatctgtagaactgtatttatattgatgtgttgattaaatttaatcataattgcatttaatttgctttattaataattgtgaattacaggccctgtttgc

15121 atcttatttgcatcttgcttctttaaataatacctttcaaaaatcacctaaaatacttttcatattcattgtagctagggtcatgatgtttggcagtacagatga

15226 tattaaggttaaccttgaccaacttagaagcgatcagtttcccactatgacaatgagacaaacactgacatatatacagtataacatattgcagcctcccaaaat

15331 acacatgtctatatacttggaacatgcatcctacacacaggggaggccatctttgaatcaccacagcttttgttaggagttttaataagcaataaatcaaagaga

15436 ttttgttgaaaagtcatgatgacaatagtcataacaatatgttagctatatacagtggaactttgttaactcaaactcgacaggactcacagaaaactttgactt

15541 aaccaagtgttagaagtatttataactaagataaagagataatcttactgggaccagcaaagtacttcgagttaagcggggtattcaagttttctgagttcgagt

15646 taacgaagtttcactttacaataatagaccaagtggcatgctcgggtgaagttctatataattacctatttctttatagctaattacctcaaattcatcctcttt

15751 cacagcggtcaaatgaattgaaatctttatatttctatttgtttaattagaatgtaacttatttttacatagacaagggattataattataagcgcacacacaaa

15856 tcctgaaaacaaattatcagcattttcttccaagaacaattgtagccaattgattggaaaagacatttgcatattgaggtatttgagcttccctgtggcatatgc

15961 tgggacttcagtgggtcaacagttgatgtattgatttttcctcaaaccctaatgctcctgtcatttttaatttgtctgttaatatccccttatacagattatttt

16066 ctacataaatatggcaaaggactcaattgttgtatataataacaatgtatataaatcatttttgtttttataagatcaacaagtatttatcttataattagatta

16171 tcttataattagatattagtaatttgactttaaataaattaaattttaaattttatatgatgaaattaaattatattactgttaaaattttatgattaagaggcg

16276 ggtggaagaggattgttcaaatcttgtatggctctgcaatgtggtggtagctataagtggttaaggcgtctgacattctgcaatcacttatcctccaccactggg

16381 ttgcgagtttaggacctatgtggcacagttgcaaggtactgactgaaagccaggggtttttctccagaactctggctttcctctactaccaaaacctggcatgtc

16486 tctaaatgcccaaatagctgttaattggacataaaacacaaaccattaaaccaaaccgtaacatgtacatttgtcatagaatcaggcatctgacattctgctgcc

16591 actagccctccaccacttggtcgtaggtttgaggttgacatggggcagttacaaggtactggccgttggtcggtggttttctccgagaactgtggctttcctcca

16696 acaccaaaacctggcacatcataacaggacataaaatacaaacaaacttcaatcaaatccagtgctggaataagcttaacttagaaaatttaggaatactgcatt

16801 aataatgcaaataatcattggcacaaatcttatttttcttagaattgttgagacagaccaaatagtcttattaatctgcaccgtaaatatgattaattcatctcc

16906 ttgcttttaaggtaatgttcaaatcattgaagaaaaatcatttatgtaacaagctttaacttggtatgttaatttgttgcccaagagaagaaaattgaaacccta

17011 tgaattttaaacagtcaactgaaagtcaaagttgctttaacacaacagaatttcacataaaagataatatgtctggatgctaaatttagaatgaatgatcacaaa

17116 gaactcacactgtgtaggttgcaagttcaaagatcaaggtcattgttatttaaccctttaaccactgtagacataatggactcattcatatcaatgcatggaaga

17221 gtctaataacgtgttataggggtgaaatggttaagatacgaaagtttttttcatctgtaccagatacctttagaacaactggcagcccttagctaaaagacttgt

17326 gcatggttaggattttgtccaagcatattcaatgttatgattatttgccctataaaagattaaaaggtattctgttgtggtttgtctaaggtcaaggtcaatgca

17431 tctaaaaataaaactacatggaataaaaatttttaggtaggcactttcaacctcaattacagaagaaatcattttataagctaagcctcatttagcgaaaaatat

17536 tctcatgtatcagcaaaaaacattcctcttgggtttttcctggggatatgctactctggtagctctcttattattaaatttgcatgatgtgaccacatgttaatt

1 M S V I I S N H L S D S M A Q G R P I K C

17641 gatactttgcttcattctttgcagatctgacagaaatttgacggATGTCTGTGATTATTAGTAACCATTTGTCGGACAGTATGGCTCAGGGGCGGCCTATCAAGT

22 V V V G D G T V G K T C M L I S Y T T D S F P G E Y V P T V

17746 GTGTGGTAGTGGGCGATGGTACTGTTGGCAAAACCTGTATGCTCATCTCCTACACAACGGACAGCTTCCCTGGGGAATATGTTCCTACTGTgtaagtgtatctca

17851 gtgctgctccaggcacagtatgcagtagatatatatacattcctaatttgtttttgacaagctttcaaaaaaaaaaaaataatggaaactacattgctgcctcat

17956 aaatttaaacagcctcagaaaactgacagaatctcagaatattgccccagcatttcgtcttgtaatgaagttggaagtttattttaatgtgtacaattttgatag

18061 tgcaacattattttactggaattatgtagaaaattggctagctagacatcaaacagtgtattcaaattgtctaagctcctagacttgatttgccaagaaaatgtt

18166 accagatgtttgtggtaactgcagctatcacatggatgtaaacacttttccagaatgtttatatataatacagtttaaaagaaaggggaaaaaatctgaaatttt

18271 agcttcattaatatttaaggcacctgtaatgaatcactttgtctttttgtccgtcatcaataaacatttatatttgtactcatcagaaacttcatgtctgatgtt

18376 gatgaaaaattaggttatcaagtctgaggtgtagaatcagaatttagactcaagtgtgccttaaaccgcccaaagggggagggggctgaagggcccggccccatg

18481 attatgtcagaggtcagagagcaaagccttcaaatgatatctcctccgagttaaacagtttggatatagtttatatattatgccactatattgagtcattcttaa

18586 agataagtgatcagtatttattctgaagaaggagctgacccccaagggggcaaggggcagggtcatagtataaggggtcactgatgacaagacatataaatatct

18691 tcaactatatattatgattaatatacaccaacaatattaatccataatgatccttagatgataggactcaagaagacgcttaatcctttcaccactataaatcat

18796 aatggacttctccatttcatagcaaggtagggtctattgaagtcgcataggggtgaaagagttcatgtcctcatgatcacctccttccagggtgaaggtgatgag

18901 ggttatacgcccacacaacacatgtacttaattatactattattttgtgcgagggtacaaagattcttaataaacatatgtataaaattataacttatgtttctt

19006 ttaccatacacctgaacaagttagggtttatagtatataccttggttttcccacctgcatgtactgatcaagacttttaaatcaattcagaagaactcaaatctc

19111 ttaggaggtagttccgttgagtcatgaacaatttaatgaacactctatagttccccaggaactagttatagcttggtttacattgaaaattatctgcaaagtttt

19216 tgaagagaaacaaaatttttcaattagttgtacagggtcattcttggtaagcacatcgtgctactagatttgaaaaagaaagaccatgtaatttaggtgggtgtt

19321 ttcaggccctttgtgcatcttgcgttgttgagtttcacccaaggtcaaacattctgagagatgtttgtctgctttccatataatgcttcttaaaagtgatctgta

19426 caattaaaaaattaaaattcaagtttaatgcttagttttggaaaaatcaataagatttacattaaaagatggtattacaaattagaatggcagaaagcacattat

19531 tcgttcgccaggtccaaaggaccattaatttaaagtatcaagtgagcctctgccatactgaggcgcctgttgtccgttcgtcaacaaattcttcaaacaacttct

19636 taaccactgcatgattagaatttaactggaagcatatcccaggctaggtgatggggattcaaaattgtataaatagtatggctgacctcctaggggtgttgaggg

19741 acagggccaaaattaaaggtgcaattatattactacagtctatacttttaatatgttaaaagttactgaggaaaatctctagatctttaattaaatgaaatgtga

52 F D N Y T A N M M V D G V P V S L G L W D T A G Q E D Y D R L

19846 ttatatttacagGTTTGATAACTACACAGCTAACATGATGGTGGATGGGGTCCCGGTCAGTCTGGGACTGTGGGATACTGCTGGTCAAGAAGATTATGATAGACT

83 R P L S Y P Q

19951 TCGGCCACTTTCCTACCCACAAgtgtgtaatttaatgtgtaaactattgatgtataccacgcactgaagtctatttatccagcatatacaccaactgtatttaaa

20056 gtatggtagagttacatgtgcagtctggtataaaagttgcttattagcaactcgccctgcatccttggtccggcgtccataaacaattcttgttattgctatttc

20161 ttggaaagtactaaagggttcattctcaaacttcaaatgtaggttccccttgggccatagttatggattgtactgttttttgaactgatcccgaaaacaatatgg

20266 ccgacagtggccatcttggattttgatagtaaaagtttgttattgcaatttcttgaaaagtactgaagggatcattatcaaatttgatatgtgggttcctcctga

20371 gccatagttctgcactgtaccttttttgaactgatctgataaacaatatggccgacagccgccatcttgcattttgatagtaaaaaattgctttgaaagaacaat

20476 gtcgatcacaatagtatgaaatgaaaggtttcatcaaaagtaaccaccaaatgcatttactgttgacataacccaataaattagatcagaaaagaagagaaaagt

20581 tcaaacttacatataattaatgaagatttaacaatggtgggcgccaagatccctctgggatctcttgttttcttttttccaatgccagatgaaacctgtaagggt

90 T D V F L I C F S V V

20686 ggattcatgtaggctggaaaaaccactcaacctgccaaccaactaaccaaccaattttttcctattttccagACGGATGTATTCCTGATATGCTTCAGCGTTGTG

101 S P S S Y E N V T T K W N P E V K H H C P D A P V L L V G

20791 AGTCCCTCCTCGTATGAAAATGTGACGACAAAGTGGAACCCGGAGGTCAAGCATCACTGTCCTGATGCTCCTGTGCTCTTAGTCGgtaatgtgtctgagggaaga

20896 tggaatggtgggaagggataggaaataaatggtaggggtggtagtttgtgacatcattacccaaagtgtgaatagctcagctgcttataaaaagtatgagcctct

21001 ctcttggtgtttgtgatctcacgccgacatttttgttaaaatgacttcctctttaaacagaaattatccacagtcatgtaaattggctagaaagtggtatgaatt

21106 gctgtagatttcctcatattaatgaccttgacctatctttaaggtcacaaagaaataggtcaaatttttttttttaggggttgcttattctggttgttgaaatga

21211 gagaaaaaatgtctttcatgcatcataaaggttgtggtagaaaatggaaattctaccttcacaattttcccatttcacaaaactctcaataacccctgggtctca

21316 tctaatttgaaaaccccttagctggaatttctaagtggatattttcttgcaatgtcaggataaacctacaaagcgctgtttttatggttgacaaaaacttcacct

21421 ctgaaatgctcacaaaggtaacttgcttctatctacaaatgaaacagatcatgtttggtaattcatttgagttgatcgtgagttgatataggaaaagaacctgat

130 T K I D L R E N K D A I G Q L A

21526 tatttaaaaaatttattctttccattttgataatgaaaaatttcctttctttccagGTACAAAAATAGATCTTCGAGAAAACAAGGACGCCATTGGACAACTGGC

146 S Q G L Q P V K R E H G I K L A N K I H A V K Y M E C S A L T Q R G L

21631 TTCTCAGGGTTTACAGCCGGTGAAACGGGAACACGGCATCAAACTCGCAAACAAAATCCACGCCGTTAAGTATATGGAATGTTCAGCTCTCACGCAACGTGGATT

181 K Q

21736 AAAGCAGgtaagtttctgtataaaatggaaaatgaccctttaacctagcaaagtatagagaactccatagggctctgtgatgtatcacttctatattaatttgta

21841 tttcatgtacaggtatagacatgtttgtcgtttatagacttttatatagtggacacacttaaccatactgatgttaagatattttaactgattaaatcaaaattg

21946 aaattaaaacaagaatgacttccaataattttttctccatgcattttatgcaattaaaacataatatcactattcattcttgggtgccatgttgtacaccgacca

22051 cctgtttgcttcaaattttatgaagcaagttgaacagtgttatttgattggaatttgggatatatttgtttagattgaatgattttgtatatatcaatgaaaaaa

22156 tgttataaaaaaaattcttttgaaattggtctcagcgtcagtacaatgcaagggaaataactcagcagaatttctaaaatttcgtaataagtagatattcaaaac

22261 aatacaaggtatgagtaatgtgtgttctggtacaatttatatttatttgattgaaaaaaaatggatgtttattgacagtgatgaatgtttgtttgtgtatatttg

22366 acaaaaaaaatatggatgttaaatgacagggtgattgatgtttgtgtatatttgattaaaaattggaagtttattaacggtgatgaatgtttgtgtatatttgac

183 V F D E A C R A V L Q

22471 aagtttgtgtatatttgacaaagaaatggatgtttactgacggtgaataatgtttgtttgtgtttatttgacagGTCTTTGATGAAGCATGCAGAGCAGTGTTAC

194 P Q P I R T K N H K C V L L *

22576 AACCTCAGCCAATTAGAACGAAAAATCACAAGTGTGTTCTCCTCTGAttggtggtacatcatttaaactgtagccatgtgacctaatgtggcaattttaaaactt

22681 atcatcagcggctgaatctcttctgtttcctgattggctgttcataatgtcataataacttggatatattctttaaagtgaatggccattcagtttatatttgtg

22786 gtgacgacagcatttcctgttgatcgtttcacagacgctttggacaacagtgtgtaaagtcatggatatctgatcctgtgtattttctgaggatcatcagttaat

22891 gaaatgtaccgataaagtgtaatgggtgaagcctggaattctggccccaatttctcagaaaaaggctcagacttaattctgtagtaaattggttttgtcttttta

22996 ccatctgtaactacgaaaagcattggaaatttatcaaaaatgctatttaaattttttgtccctatgagaccctttcacccctatgtgactttactaaactcttcc

23101 atgcattgatctgaatgagtccattatggtctacagtggtaaaagggttaaatcggggaaactttcagattttttttctgaaatgacaccatatcaaattacaag

23206 ggaaaccaggaattgttttcaaatagaagatatggataattgattttggttttcattaaaagaagtgttttaacgacaaaaaccaaaagtttgagctaaatgggg

23311 ccagaagccagtagggctgtaacgattcacagcacttacaatttgagttacgatttgattgattcttgattattttggttcttctgatatttttattcaattgtt

23416 tcggctgtaaatgaaaattttggcaattttaaccaataaccatccataatactgtaaacgttcatattttagcggtaatctaattttagcagttttcatgctgtg

23521 gtaattactctttcaataaattagattgtagaaaaatgtcaatggatcaatttatcattgtgctaatcaactcaaatatgaaaatgtgctaaaatttgagggtgt

23626 caaaatgagtacatatgcagtactttattggaagtcatatatatatatggtaagtgttgcagtgaaaagggtgcttacctttgacaaagtacagtaccagaaaaa

23731 gtgttgggtcacctgcccaatcacactgaactctccaggttatctggtgtcctcccacatcaagattctcttcatggtaacatcagggccaacaaaagtgattgc

23836 ttagaattgtcaaaacctgttttataaatgttgtgaaatgaactaagtttttatatagtcagtgtttacgctatgaatataatcaaatttgaaaattttctaatc

23941 aagaatcaaaccaaatgtaatcaattgtatcttgaactgtataaattgctacagcactaggagctagtggtataaaaggaatcgaagtgctgctgctgattcagt

24046 tggagccagcgaacttccctccacaaactctctcggaaccccagtgccagctaccactgaacgtttgaactgaacaggaatgtagtgtcttatcaatgatgaaaa

24151 tatgtgttcgtgatatgaacgatgaagaagaaaccccagttatttctattgccatatatgtatctagaatgtttcatgtgtgtatttatatatatctcatctttt

24256 gtgactccacggattttgctgaatctttctaatcaattttataacctgtgtgaaagaggattctttagataagaggatgttggcctgttgaactgttatgtgatc

24361 attgtacagccttagttacgtacacacacaaggggtgtggcacagatcccaaactccactgcccatatatatatacatatattgtaccctgggacttgggatctg

24466 taccacgcccctgtggttacatggtttcggttaaaatcagaaaatttctacatgtgacttctattgtgttgctatattatatcgactagtcagagcatgagcaat

24571 gtcttcttagatttaatgttcaggaaggttttttttctgaaaataggaaaaaaaatagaaataatgtagaaatgaaattatagaatttaagcttgaatacatagg

24676 aaaaaaacagacttgtttatgtaaagtttatgtaaaggcagccccttgtacatttatttgtcagaatgatagctatgtatgctatccttcagttgtgtaagaaac

24781 cttaatcatttgtggctctgcactgaattttacaagctttttcatggaactggtttacgttttaaacaattttctttttaattgagttttccaatgtgagcaaat

24886 gttgtataataagcatggttgcaccgagatcaatgtttacatcttcaatttacgatactttgatcaggaaataaaaaaaaaattattatctctcaataagttgtc

24991 aaattttatcagtactattatgaactgtatagagatattagcaatacacagagtatttcaggatcatgtttttgatatttttgtgaaaaatttgtttgatattac

25096 cacaagtccagagaaggatttcttgtgtaaatgtgtaaactagtgctggctttctctgcccctcataacatagctactgtaaacagtctcccctctcgcttgtta

25201 taagctgcgcctctccttttttgggcattaaatggaaatgacctaagcgtccttttcctcggacatgctctattctggccctatcctgttaataaagtaaagtac

25306 accttatatctgacacttcagtctggcaatgtcaaaacagtgtccaattgatttagaaaattaagaattctccccatgaatatgtccgacagttataacaccttt

25411 acagcaatagaccagacctactttttgattaagtgtggttttgctctaactaagatttgtataagatgacattaataggtggtttggatgctattctcccaccct

25516 tcagggacatgtcttccgttttagctgagaaagatcagcttgtaacctcatagcgtgagatagccttgtgccgattaataggtatgtgttccgttatcacatcat

25621 gaagcgtgtcaccagtaattgtgatgtcatcaaaagtggatgaagataagcttaccaacatataattgtgtttacaaacatggcagactattgccacaggagttt

25726 ttgactaaaataataggatggaagaaaaagtaatcatcccctaccttgagagtgtaacagagaaatctcaaaccttcggggtgattcatttttgaatatctaagc

25831 agagatagaagtgttcagtcgattggctgatttcagccatttttagaattccagtgtaatgtaaacatttaatttttcccatttaaaagatagagtaacctaatt

25936 ttcagcatttttgcccgaagtccattttcatctctccttaagagacgagggttagctcttgggggttagacaactagaaaataatcacgcctggggttggagatt

26041 tttctgtcataccctcaagtttgggaatgattctattagtcttgttatcatctatagtattgacgatactgatcaggacagtcagtaagtgaagaaccattttat

26146 gtatgaccaaacctcgatg

**CfMiro**

1 agatttgtgttatcacatgttattcttatgtaatgattaattgataaaatatcactttaaacaaatcaattgatcacattaaagaagcaatttcattaactgcaa

106 gttaaaaatggtttaatttatttatgaattcccacttcttttagtacaccatgcagagaccctaatccacactgtagatgaacagagcatacaaaatgtgctgta

211 ccataacactacacacaggccctctaacaaacaagtaatacctacacataaccttattatggacaaacaataaacaatcaatctgttcaaagtttgataattatc

316 attaggttgtagcctaagcaaggtcacatgtgtgagattataatggacttgtacaagtctatcaattagagtgctattgtaattagaaaggaatgacaggtaaat

421 taaagaataacttaccttctcacctttactaggagactgtaactgtaccaatatggatctgagagagccgtgttttttaaccaccactgtatatagaatagatcc

526 tgttctaacaaatgcttgaagccaattgaactttaacaattttacagtgttagcatttgcatttatttaaagggagcgctattttgaagtgacccgttaatacct

631 tcagattgtggtggggataaatttaagtctgtgtttatactggtagacggtctcgtggtcacagacctaaatcgtaccacacaggtatgaacctgtaagccaaat

736 catgtatacctaccactaatctggtcttagacacccatcaatccttcaaaacctagtatgtacacaacaatttgtacgaaccagtaccagtaaatacatgttttt

841 aatgttattaatgttttgtgataggttttatctagattacatgggacatacattactttttcacttcttacctgtaaaactcaatcatgtagctaatgtaatggt

946 atacacattaatattgctgacaatcccatcatttcttgattttcacatctgttttattgtttagaaataaataaaactttccaaaactaatgtaccatttgtcag

1051 acaaggtaaccacggtaacaaataaaaaggagcagctttttcctttcacattcagacactctgtacacttccataatgacaaaagggagttcatattcaatttta

1156 ttttgcagcaatgttttaaaaatatatgatttcattaaaacattttgaagggcaggaatccattggaatttagcatgggcatcagttgacagaagtgtttttata

1261 ccctactacagagctactagctctgtaacttacagagagatggatggacaggtccaaacagttgtaacctgtgaatattctgtgtttttattttactgtgctatt

1366 taaacaatgattttaaataaacctgacaagttaagtattttaataattaaagatctcttaacaaacttcaaagtcaaatgaggtgcatactaggcctgtattgct

1471 cacctggacatgtaagcaagcacttgctctgccccttattcccccagggggtatgtaggcaccttgattcatatttttacttttccaatacatcattttcccatc

1576 tcctaagaataccttgaattaagttaatttctatctattgattctatgagcctaagctgttttaagcaatctggtcaaatattgtcctttttcgtcaaattccaa

1681 tacaagtcgcacaaaacatttgatggaaattgacattaaaaaataccacttgttgtgaaagaaaaattctgaaatgagttgaattacaaatcaagcaatgaattt

1786 cataggttttttcaaatattcctattgcaaaatttcaacaagtaaaaatgtctatttctctttcaaatacatttcaatagatgttataacagactcctgttttgt

1891 aattcactgctacccctggtgacatgacatcacagacatcacttgacaatatcttcaaattatgaatttttacaaacaggctagttgtttaattttacctgacca

1996 tgatgaatttataatcttcagtgaaagcacttcaactttttcaaaagaaaggcaccctggagtaagtgataaatttgattaccaacagaaataaatgtaaaatta

2101 atctgtaaacaaattggagtctcttcaatctttttgcttattttgatataaacaggggttaatccgggccagggacactaaccttgtaaggtcttacgtttatac

2206 acagtgagattatttcgctgagtattcacagcagagaaagggataagtcatcgaccgttcgcaggtatccattataccaacaaaataacacggtgcttagacatt

2311 ccctaatacgatacaaatatatctagacagatattgaatgtatttaatcacttttcattgtacaactatactgacagctaaataagattgtaagcacatattaat

2416 tacagtaaaactgttgttaaatatcacatgctacagcatagttataattattaacacatcaaatttttttaaacattaataaacattgttgtttaagactttcca

2521 acatgtcaatagttaatacattgcaacaacatgttgaaatgggacatgccagtgaattaagtagctagcataattggatcaatgccatcttattgtgtattttat

2626 gctaatagtcaggttatgaaacttgatatacagtcaaagtatctagggtttgaaccaatatacaatgtacatattaattatgttgaatttttcacagagaagacc

2731 atatcctagaaaattgtacaggggtataaagtgttgacttcagtaaacctacagtgatttgaactganttaaaaacctgtcaatagttttggaactaaccggagc

2836 atcatttacaatggtcctagaattatttactttgctggttatttcggctaaagaatttcttctgtttttccggacacaaagaataacgcgacgcttctcacgttc

2941 gtccagaaatggcgtccttccgcatcgagggttattaacaactgttccacgggttacataacgtttccaaacatctgaaatggtagatttcgagacacttaaaac

3046 atccgcaaccactgattgcttggttcctgccttaatcaatttgatgatagcttccttttcaccaaccgtcagttcaacacttcgccgacccatctttctttagca

3151 gactgtcagaaaacatcagcagatgtcacgtacgtttagtgacgtagatgacgtcaacagtgacgtcaccttcccgctggtgtacggtaataagcgaacaagtaa

3256 cctattaagattttatccgtttttttgtgcgcctcacggtagtcgacggcgcttaagattagccatttgtccgaatacttctgtccgatctctgtactaaccata

3361 tgatataatacatgttgagtatttcataggaaattgtatcaggaagttcaaagttaaattacagttcttggtaatacaacatcaaaatatttcaaaaacagaaag

3466 aaactggccttcaatcaaatgcaacatacagatagttatagatacatgtatgtatactgacctcgttgccttttacaaaaattaaatgtgtataagtttatacag

3571 ttacacattgtaaaactgtgacattgtgtgaatatactgtgtgtttacatgctaaatgatttttcaaatgttttaaatgcatagatccaagtgtatcaatatgat

3676 ataatacacgttgagtatttcataggaaactgataaatgtaagaattataatacatggatcaaattattccacctcaacaaagaaaaaagaagtagaaatgttat

3781 tttttctaacaattgaaatttcttaaataaacacagccacaaacatgttactgtcaagtttcctggattgaatgtactatgtaaagggcaataactctgtaggac

3886 ttatataacattgcatcaattcaacaacttgaaaccttcaaaataaaacatgtaaaatgaaattttcaagcaatttccctttttgggggaagggttgaaaacata

3991 ttgtgtcaaatctattgtccaatattaatttcccaaaaaactcaatatttcgtaaattcataatgacaagatttaagattgcattgtattgtctttctacctgta

4096 tttagatgtcaccaacatctatgccaaaattgaaatcttcttaagtttgaataaactgatactgtcattcttaaaatttttctaatgtatgaaaattgccaaaat

4201 gtgaaaaatactaaatgtcacttggctgtgagataaatgtgcacatgtttgtactcaatattttgttcacttgtgaaatatacaagttaaatactcacagttgtt

4306 tgtaattcaactctttgacctctttagaattaataaattttacatattcacagcagtcatggtactactgatgtttaaattaggagacattattaactttaataa

4411 gtgtagattttaatgagttcattttcattttaacttctgtgaaaattgtgcaagtaattaattcagaaacatgcatcaaaagacagaaataatcagccagctttg

4516 aatgaaaagcaaactgtgttctgtatttaacatttaaattactttgagcattcagaacaaacctaccggtattttccagtggtcaatcaggtaccgtaccattac

4621 attttttgggcttgaatgtaaagcatgacacactcttgcaatgtgtagccttaccctacaactggcatagtctaacagaaaggttagatttcatcatcaatggct

4726 tatggcagtaatggatgttgcagacacctaaacaatcaggtcagtgcccaatgattactgagttttcaaaatttcaactgagcataactacaataactaagacac

4831 tttcctaatacaaatgattctccagcttgtgtcctcctctgaagttttctgatatcttaagaactatgacaaaagtgaaaagctatagagggccttatgcactgc

4936 ggtacatatatgtgtgtactgtgtagggtattggggtatattttttttcataaatacaaacacaattctattagtcctctgataaaggacatggatttgtgatta

5041 tgactgggtcatttactaaataggtagaatgtgtatattgatttacaataatcaagtcagaattaatagattaacacttaaattgaccataatgttattgattcg

5146 taaccgagtatttcaatattatgtgatgcatgactgtgagctctacaagatacagtatcattgattgatccatgatgtaggcctacagtagctcgatttcattca

5251 agtgacagttacatggccggatgacaaggtgttgtaggctaggtcgagtgtaattgataacacatgacgatcgtcgttatcactttctaatcgcattggcagagt

5356 aaacaatctaaatactaagaaatacacgtaggcctacttaccattcaatctgccacatatcacacctgcgtctctctcgttgctgcagacgccagtcgtgtttcc

5461 tttaatgcgcgcgcaaataggaagcttttgtcggtcggaaagtttctattttgacgtatgtatctctaataaacctagaaataccccgtattgtcaccgaaaatg

5566 gcgacatttggtgtcttttaacgaccatgcagttgtagtacattcatgaaatactcttcgaatacgtaccaagttgaagcataaggatattgtttaaatttattg

5671 gtatataatgttgacgaaatttcattcaaggcatagtttgcggttaacaatgtgaaataatccacatgtaaaataacgcgagtttgacctttcttggcaagcttt

1 M S S K R D V

5776 catcatcatgagggagtaaacataacataaattcagaaaagttttcaaggcactttttcgtgcaaccaagatttaatactgcagtATGAGCAGCAAGAGGGACGT

8 R I L L V G D P

5881 CAGGATTCTCCTTGTTGGAGATCgtaagtttcagtttgccaatattacccatgtatcattgtactgttatcaatgcagtaattccttattcacttgttgatgatg

5986 aaacctttgtaacgggaccatataggcacctataaatgataaaggttgttggtttttttttttaatttttcgtatagtagtatcgtcccttattaaataaccagc

6091 acctgatgccatggtctcagcacgtacacatacacctgactagtgtagaatgtatgactaagagcaattttgtcctgagggtaatatataggggcatcccaatgg

6196 gactaaaaccactacagtagagatcaaaattattgacaggatatatagtaatgtggatatgtaccttttagactatatatcgattgacctatccgaaatagctct

6301 tttgagctattttaatgtgattttcttttttcatgaaaacaaaacagttgcatttaaaaatatattttatcagtaagttgatagcaaaagacattcagtataacc

6406 attctttttaaaaaataatgatgtgaaaaaaagaaaatttcacatttatataaaatgaaaatgaacatttaaatgtatgttttttgttgtcaacattatcataaa

6511 ccttattgttttatgctactttgctttaatttgatcaaaattgaaattcgagaaattatagctcaatacagctataatttatatactcaatatagctatgtatag

6616 gtctttatagctatatagctcagtcggcagattttgctggacctgatatatatccaccctattttctgagcattattaatcttgtgatcttaagtatttgatgaa

6721 gtattacatcaaacatggaccaacacagtacctgtccagcataccaaaagtgtttaaggaaattacctatcaaacaaacctgaatttcttagacgttagacattt

6826 tgacagaatcagtaaagggtctatctgtaagatccagatctgtcaatcattgtgtcctttttaagtgtgttcatccaaaacgccatttcaaactaggttcagtta

6931 tttacagacactgtcaccacaggaagaaccatggacattatttttgtggacttaaatgctttaacatggattgaaccagtggtaattccatcggttgttttgcca

7036 tagagatgtttcctctatttttatcttaacaaaatacattggaatttaggaaaactcaaaagtactgtaggctttatgacaaaccaacattttatgggtttctca

7141 atatatgtatctacaaataaaaaaacaaatcgcaaactatacaaatttctagcacttttacccatcttggggcttttaagagttcagacaattaatttgtgacat

7246 cataaatggatcattcaaactccatattttaattgttttttttttcaactgatttaccaattatcagttaaatgacatcatggggatggtatctgctgtaaaaac

7351 attttaagaccttgtatacatgtataaaagtattcttatctatgtaatcatgtataattgtatatttttaaatcaacaagtttaccttccagtacaatgtgttaa

7456 atacaaaatgcaaatatttttctgaaatagaattatgaaaagtgatacctgagaattaaagcaatacacatggaatttgtgatgattacaaaggtgctttaaact

7561 tgatggaaatatcagaaagcacaatgagtctatggtcatttccaacttattggggcttgagaaaatcataatatacaaagagcttatgattagcaggacttttct

7666 tggaacaattttgagcaaataatgggccccaatcaaaattaatatttttgccaaatcttggaattttcagttttattcctgaaatttcttcccaaatggtatttt

7771 gttttcaaatgaaagagacaaaaagatctcctcccaattatcaatgaaaaacaacctggttaggtactctattgcaaactaaaaatatggtagactttacattta

7876 tatcaaaattagttttgtgataccaagaaatttttccattaatattctcaccaagattgataacaacataacatgcctattttgtaacgcctgtaatacctggtt

16 G V G K

7981 ctacaaagttcttctactttgcataaaacaaaacaaagactttaaccaaaactatttaagattactgataccagtatgttttatgattacagCTGGTGTTGGGAA

20 T S L I L S L V S E E F P E E

8086 GACATCTTTGATATTGTCCCTGGTCAGTGAAGAATTTCCAGAAGAGgtaagtgtttgaactagaagtgactgtaaatattaatggatccagagttaatttgagac

8191 acaggacatgaaaaattgtttagaagctctttgccacttggaaacattagatgaggtcgtggccagtggaacttttcaaatatgagcagattaagctgtgcatgt

8296 actcgcttgaccaacaaagataaaaatggcaccaaagcgttcagatacgaacttccgtgaacttccatgtattgaattgtatgatattttaaatcaaaaatgtat

8401 cacctaccccaaataagaaaataatgaggcttttccacctaaaatgtttttgacataaattgcaaaagaggacgaagctaaagcattacagttgcaacagaccgt

8506 cagcgacatataaagtatagactgaggtgttttaattttgtaaatgaaatgtcaaggggtttgttatggacaaacaaatttacaagaatgtcattaagttgtaaa

8611 atttaggcatattgtcagtatatggcagattgtttgcccccgtattcttgcattctcttgcgtgacttgtgcattttccagcaagaactcttagcatgggagcaa

8716 ctcgtgggtggtcgctgtatcatcaattccatgagtgatttaactgacatacacaggaatgtgcaacagccaaggataccaaatatagtatcattgtatgtcatt

8821 aggtagctctctgtaatttgtccatacataacaaatgaattaagaaagaaacttattcatttggacttggtgaatctcgttttaaagatatattttccaaataag

8926 ggttaacccttcaccattgtagactaaatttgactcaaccagatcaatgcatggaagactaaagttacataagggtgaaaaggttaaatgtttctgtttatatat

35 V P S K A E E I T I P A D V T P E K V P T H I V D Y S S

9031 tatgtttttatctcccaagGTTCCATCAAAAGCGGAGGAGATAACAATACCTGCTGATGTTACCCCAGAGAAAGTACCCACACATATTGTTGACTACAGTTgtaa

9136 gttgatcaaattacatacatcatttataaacaatttttaatacacagtattgtagaacaactgttaatcatgtttgttattggttccattgtagttggtactaaa

9241 aaagggtataactacatattattgttacctcatttaatcgacccaaaattgaattacggtacaaccatatatgattttaaagaaaaaaacatatttgttaaacaa

9346 gcattagtcttcacaaaagcttatttcatatttaaaatcatatgagaaggatgaaaattgaatcagagttcaaaagtatttggtaaaggaaatatcaaattgatt

9451 acaataacattataatttgtgataatattcttatagcagaattataatgaattatgcaaaatagtataattacatgaactaccaggtgtagttgataaaatttga

63 Q E Q D E D R L Q E E L A K

9556 ctgttgctgtatttatagCGCAAGAACAGGATGAAGATAGACTTCAAGAAGAGCTTGCTAAGgtatgtgatgtattgagatatgtatacggtaattgtggggctc

9661 agtgaagggtcattggtgggattatagtttgatcaatgaacaatttattaagatttgtcagttatgaaattgatgacacttgtttagactttacagagaaaggtt

9766 aaaaagtaaacatattttacttaaaagagactaataacaaaaggtttgggaaaacattctgatgaagtgaggcatacacaaaccacttgtgtactacacatatgt

9871 agtgctaaagactactatttcaagtcttctacaactttgatgtagatttggttgaataagggtgaagtacgataaaaatatgatgtggaaagtgtgctgcctgga

9976 gacatttattgtctcatgctgaaggcagaaaccagatagtttctagattacatctttaaatatacatctagtattaagttttattaaactggttaaaagtgtgta

10081 ttttacccagaccaagtacaggtatacaatctgctgagtttagggtgcaacaatcttatttgataatattttctgatgttaccattgaagaattgctagtatttc

10186 catgtaaacctagaacctagtttgatgtcatgcatagtgccaacatattcaatgagagtgttgctatctgttggctagtggttttaagacttatgcttttgagaa

10291 atgatatttggtttgttaagttttctggcaaaaatgaaaaaaaaatagtgattttatttaagtgtacatgtacataaatagtctaataggcttaacaaaagatag

10396 ttcacttctcctattgaaaggagggacattcaagtgtaaatcagctggccttaaaatttcagttttgatgtctacttctttttgacaaatgcaagcaacaaatcc

10501 ataatcatgaattctagtatttcacctccccccccccccaaaaaaaactgttttgaacttatatggtagccaattttgtaatgttttatacccatcttctacatc

77 A N V V C I

10606 ccaccctgtagtaataaccattcagtttattacactgtttacaacgttcagaaaaaggtgtaatgtatggaatggttttatattgcagGCTAATGTTGTGTGTAT

83 V Y S V D D E D S I E R

10711 TGTATATTCTGTGGATGATGAAGATTCCATAGAACGGgtaagcttaaccttcactgctaaaacattataatttctcacatgtttataccacatacctgtgtatta

10816 catccatatatctgtctgatttgtttggccattactacacagttactgggtacttttcttttgacaaaaacaatttcattatttgaacaatgctgtcaataccat

10921 attggtttattggttggatactgatgatttttttaaagaattattttatacgtttgtgaatcaaggataatgcagaaaagtatgaatttgtattttcaataaaag

95 I T T F W L P Y L R N C L G E D H R T

11026 tcatcgcatgtattatccacaatatatattgagagataattattgacagATCACCACATTTTGGTTGCCATACCTACGGAATTGTTTGGGAGAAGACCATCGCAC

114 P V I L V G N K T D M L D F S T M E

11131 ACCAGTCATCTTGGTGGGAAACAAGACAGACATGCTTGACTTCAGTACAATGGAGgtctgtaccagacaaatttaaaccaaagttaacacagtatgatgtgtatt

11236 atagaatggaaagttacttgctggtaaatcttatcaaaagattgtgaaaatcaagaaaaaagaaaatttattgaaagatacctgagcttatgtttttcccactct

132 T M

11341 tttgataaatgtagtgggttttgtaattaatgaaaggttaattcagactgttgttagatgttactttatggtgttataaagtgatgttccactattacagACAAT

134 M P I M N D F A E V E T C V E C S A R T L K N I S E M F Y Y A Q K A V

11446 GATGCCTATCATGAACGACTTTGCAGAAGTGGAGACTTGTGTAGAGTGCTCAGCAAGAACACTGAAGAACATAAGTGAGATGTTTTACTATGCACAAAAGGCAGT

169 L H P T A A V Y N P E E K E

11551 GCTCCATCCCACCGCCGCTGTTTATAACCCGGAGGAAAAGGAGgtacagcacttaatgtcctccacactcataccaatctaaacccaaacccagttcaataccga

11656 tacttgtatcaataaaactttccgataagttcattaatttgtcaagtgggcaaacttgctgaagtattgatacaagtgaaattcatttagacagtttgcagtgtt

11761 atactttacacatttttacctatatagcatgtgagttaaatattggagtcagatattttataatctagattatatttttaatgttattaaaattttaacataatt

11866 gtgaaagaggaaaatgctggaactatttaatactgctgtgctgacagaacctttcatttacaaacagttttagtttttaaattctgttaaaatcaagaagctttc

183 L T P Q C K K A L T R I F K

11971 atgatgacgttatttttgttacagCTGACGCCACAATGCAAAAAAGCTCTTACAAGAATTTTTAAGgtatgatacagacattcacaaacaacgtcatgcttactc

12076 ttaatactttttaaatcaaggtaataattgggaaaactgttacttaaaagtaaagaaatgttttaaatattgatttgtcattgtggttatataatcgtgcatatt

12181 taatgtattttgcatatccgaatttgagtttttcgagtatttattactaagataaagagagaatttggccgggaccggcaaagtacttcaagtttagcggggtat

197 I C D L D N D S I L K D D E

12286 tcgagttttccgagttcgagttgaccaagttccactgtatatatttgtgttgtttaccttcagATATGTGACCTTGACAACGACTCAATACTCAAGGACGATGAA

211 V H L F Q

12391 GTACATTTATTTCAAgtgagtatttgtacttaattactgagtgtcattttaaggaatgccattgtatagatccattattatcaggtttgactgaagaaaacatgt

12496 gcaaatcacttactataattctataggttataaagaacgtatatctggaacagtcactcattatgatagttgaagttaaaattgaaagtatctaaagactttcat

12601 catcagattgttaaggttgagaattttgtctacacagtatatgatttccctgtgtaataatttaaaacaacaaaaattgatacccctaaaacttgctggcatatc

12706 cttattttgcaaaccatggcggtaacaaaaatcccagaaagaaaaatgagtgggaacactgattgtaaatgttttccctgtatgacctgtaaggcttagaattta

12811 actacacaataaatgatatccctgtatgacaaaaaaaaaaatttagaattttcactacacagtagatgatttccctatatgacctataagattgacttcacagta

12916 aacgattccctgtatgacctctaaagtttatatttcgacttcacagtaattgatttccctgtatgaccaataaagtttagaattttgactacacagtaaatgatt

13021 tctctatatgacctataacattcagaatgttgacttcacagtattaaaatgatttccctgtatgacaataaagtttagaatgttaactacacagtagaggatttc

13126 tctatatgacctataagatttatattttcactacacagtaaatcatttccttgtatgacctctaaagtttatatttcgactacacagtatatgatttccctgtat

13231 gacctataacgttaagaatgttgactacacggtagatgttattcctgtatgaccaagaaagttaagaattttgactatacagtaaatcatttccttgtatgacct

13336 ctaaagtttatatttcgactacacagtatatgatttccctgtatgaccaataaagtttagaattttgactacacagtaaatgatttctctatatgacctataaca

13441 ttcagaatgttgacttcacagtattaaaatgatttccctgtatgacaataaagtttagaatgttaactacacagtagaggatttctctatatgacctataagatt

13546 tatattttcactacacagtaaatcatttccttgtatgacctctaaagtttttcttctatgacctatataaagtttagaatttttataacacagtaaatgatttcc

13651 ctgtatgacctataacgttaagaattttgactatacagtagatgctattcctgtatgaccaagaaagttaagaattttgactacacagtaaatgatttccctgta

13756 tgaccaataacgttaagaattttgactacacagtagatgttattcctgtatgacctataacgttaagaatgtagactacacggtagatgttattcctgtatgacc

13861 tataacgttaagaatgtagactacacggtagatgttattcctgtatgaccaataacgttaagaattttgactacacagtagatgttattcctgtatgacctataa

13966 cgttaagaatgtagactacacggtagatgttattcctgtatgaccaataacgttaagaattttgactacacagtagatgttattcctgtatgacctatgtacttg

216 R K C F N A P L Q P Q A L E D V K S I V R R N

14071 tgagatgctatggtaactttattactgtaccattacagAGGAAATGTTTCAATGCTCCTTTACAACCTCAAGCCTTGGAAGACGTCAAATCAATAGTGAGAAGAA

239 I T D G I I D N G I T L K G

14176 ATATCACAGATGGTATCATAGACAATGGAATTACACTCAAAGgtagacaatttatgtcggtcaagacaagtttatctctaagttaattttgttttttattacctg

14281 gtttgtcgctaaattttgctgagttttagtttcttctttacttttattaaataacattttcggaaatcaatgcaattaattcacctgctaaaaatctataaacca

14386 tcttcatacagctactgtaatatcacattacataactccatttattcaggagaatttaggtgttacaaaggagtttgtttatttactcctgaaggcagatgcaag

14491 acaaatcaaagaactgttaacaaaaatagaaactggaatcttgaattgtaataaaagaaaacaattttgcctatagaagaaaaggctatatgtatatataggtgt

253 F L F L H T L F I Q R G R H E T T W T V L R

14596 gtgtgtgtgtgtctgacgatgtatgttcctgtttgtcagGGTTCCTGTTCCTCCATACACTCTTTATACAGCGAGGTCGTCACGAGACCACATGGACTGTGCTAA

275 S F G Y D D G V L L S K D F L S P R

14701 GGTCGTTTGGCTATGATGATGGTGTCCTACTTTCCAAAGATTTCCTTTCACCAAGgtaaaacatggcacacatatttatgtgacactttgataaattgactagat

14806 tcacaggcgatacttgaaaactcctaagcatcataggaatgtattcttccaaaatgaaaaatcaattgatgttatattagctgttcaagtgagccaaaaaaagta

14911 aggaatttgagagcattcaatcaaaagctgatatttgtagctaaagctttcatgtaaaacgccatacaagtatttttttcgaaaatcaaaattatttcaaattaa

15016 taacttttgcaatcaacttagtagttcatttgcaaaagtcaaacaaaaaacaaaaaaaaccacacaccacaaagtgtattaccataatccatgattttgttgtgg

293 V Q T S L G S T T E L S T Q G I Q F

15121 ttggagcttaaattttgatgttttttacagtttccttctgttgtgttttagGGTACAAACATCCTTGGGCTCTACAACAGAACTGTCTACACAAGGAATACAGTT

311 L K M I F D K Y D D

15226 CCTTAAGATGATATTTGATAAGTATGATGATgtaagtaggaccatcattactatatagctgtatatgtataaggactgtttaataatagctaacagatttgttta

15331 ttttaatgactaattgatttgttttcataaatcacaaattgcatgcattgtgaaacttaaagaataaaaatttatgatattcaaaatacatctatttttgtttta

15436 tgaaagatatttttgtgctctttatttcttgaattgcccaggaaaacatgtacactgtatttcaggtttgtgtgttaaagaattgttatctccacttagagttgc

321 D R D G C L S P T E L Q N L F S T C P V M P W

15541 tccgtgtattaatcttgtaatctgtattgattgaagGATCGAGACGGTTGCCTATCTCCCACTGAGCTCCAGAATCTCTTTAGTACATGTCCTGTAATGCCATGG

344 G S D V N N T V C T N H N N W I T L Q G Y L A Q W A

15646 GGATCAGACGTCAACAATACTGTGTGTACAAACCACAATAACTGGATCACACTGCAGGGCTACCTCGCTCAGTGGGCgtaagtctcagcagccaacggcaaacaa

15751 gtcttatattgcaagattaagtttccttctttatgtaacctttatttatcgattttgaaaatcatgactcttttttttaacataaattttaatgaaaaaatttgt

15856 gctttgttaaaatgcttgccctttaaaaagatattgaagaattttagtttgtaagatcctttttagagaaagtatcagttaccggtacaaatgaaaaaaatagta

15961 atttaaaaaaatataacaaaatattaattgattttgggtaaaaggtggtacatgtattatttaccctgcaatggctgcttatatttctcaggaaggcagaataag

16066 acatactggtaatgaaattatatatcaagtgcccctacttttactaatctatagcttcaaactagattaagtgccccactgtttagagatttaaagcttcaaatt

16171 agataaattgtttagagatttaaagcttcaaattagatgaagtacccattggtttcagaagcaaatcttcaaattttataaagtgcccatttgtttagagagtta

16276 tagctgttttgtccagagcatatctttcttgcttttcacacttatttaattaaacttgagatactaatgcaccttgacatgtaataaaaatcaaaattcaaatct

16381 cttcaaattttacagaatctgtagacagttttataaagtcaagaattaaaattcttaaaagatatgttctgagaaagcttctgacagctcaaaatattttgattt

16486 ttgtgccactcattatccaaagttttgatctatttttcacttttgaaaaatatctcaatttatgatgtcccagttaaagctagtctgattgtattttttggaaat

16591 cagtgactgctaacatgattttgtaattgcatgttgtaaaaactagaacagaaaacgtgtaatttcttacttgattgagatcctgttgtaatatttcttaatcct

16696 taagactgttatgcttcaactagccaatattaagcacattggacttccagatattcagcttggatttgttagcatacaaattggcatactagaatttggccttct

16801 agttgaaggtctaatttacaacataaatagctttgaaagtcaataataaccaccaaaaggacaaaatcaatactaaccaccaaaatgacccttcttattgtagca

16906 tcattctctttatctaggatcagcacaccctctatgtttgttttgaatagcaagattgattttcaagaggaatcaaacatttcaagacttgcaaggtcggaattt

17011 agttttgatatatcatgaatgactgctagctgtagactagtatctgggaaacaaatgtgcatctattggttccctctggtatttaaattttggtagttttttttt

370 L T T L L D V P R T V E N L A Y L G Y H Y H H

17116 tcaatgtcatgaaacttgttttggtttttatttcagATTAACAACGCTATTAGATGTACCGAGAACTGTGGAGAATTTAGCCTATTTAGGTTACCATTATCATCA

393 D S Q L S A I T V

17221 TGACAGTCAACTCTCCGCAATAACAGgtaagttgtatatcaatacagataagtgtgtctccttgaccagaaagatcagagataaaaatatgcatcattgataaag

17326 cattgtttgtaatagagttttttttttttttaagttaaattcatcccaataaccaagtacatacatcttcgataaaaagtcaaaatgaagttaattgtcttgcaa

17431 ctatagttcagctaattttatacaccatactgctgcatataattatcataatatcacttttcagggatgttcatgaaaataatgcattttatttttattttcaca

402 T R D K K I D L D K K Q T S R N V F R C Y V L G T K G V G K

17536 gTTACTAGAGACAAAAAAATAGACTTAGACAAAAAACAAACCAGCAGAAATGTGTTCAGGTGTTACGTCCTGGGAACGAAAGGGGTGGGCAAGgtaagctgtata

17641 actgaagtaacatcttactgaaggcttttacagttgttacagtccagtttaattattccaagcagccaaaaaaggataattatatttttaagaagtctagttttc

17746 tgtacaaggaaagaaaaaaaaaatctagcgttatacaaacttagatatctggtatgtttagtgctgcagaatattcttgttatgtctccccttcaccgaagggag

17851 acattctgtttttgcacgagtttcttcttcttcttcttcttcttcttcttnnnnnnnnnnnnnnnnnnnnnnnnnnnnnnnnnnnnnnnnnnnnnnnnnnnnnnn

17956 nnnnnnnnnnnnnnnnnnnnnnnnnnnnnnnnnnnnnnnnnnnnnnnnnnnnnnnnnnnnnnnnnnnnnnnnnnnnnnnnnnnnnnnnnnnnnnnnnnnnnnnnn

18061 nnnnnnnnnnnnnnnnnnnnnnnnnnnnnnnnnnnnnnnnnnnnnnnnnnnnnnnnnnnnnnnnnnnnnnnnncaaaagcttatctatgtttgatatctctttta

432 S T F L Q G H L G R N L R Y I A T L N K E H L S S F T I N T V Q V

18166 tttcagAGTACTTTCTTACAAGGACATCTCGGTAGAAATCTGAGGTATATAGCGACATTGAATAAGGAACATTTGTCCAGCTTTACCATTAATACAGTTCAAGTT

465 Y G Q E K Y L L

18271 TACGGACAAGAAAAATATTTACTGgtaagaaccgttttattgtaaaaacaaaacttatataaatgtaccttctgttaaattagtcttaatatatggaataattct

18376 aatattttagacttttattatcttattatctaaataattaaatacctgaatcaattcacctttatttactagtggatgttttgtttattatccagcactttgtag

18481 gttttttgtagtattttgctgtgtataatacgcatttatgtataatacgccccacccttttgaactactttttttgggtaaaaaagtgcgtattatacacaacaa

18586 aatacggtgcttactttatcccttcttttgtaagtattacagaaatgtatcacaaaccagtaaatatataattaatgctaatagcagtacaagtatgtgggaaac

18691 atgtacattactctatatgataaatgttggctgtgtatgtaatgttttttgtattggattacaatttactggctacatgtatatgataatgtaaatgtccttcat

18796 gcatattagtccagagtgtggtggtctgttaaaaagtcactcttgttgtcacctaaatacaggtggtgggcacttgtgtggaaggggaaatcatatagaggaaca

18901 aaactgatacatgtttgaagaattaatttgaagtatcctgctacatgagaaagcaaatttcagggatatttgtgaaattgaaggaagactatatgatttacatat

19006 agatctgatattgaaacaaattgaaaaataaattttgcttaaagtggtcaaaccaaaaaaatcttagagtttatcctttattgctgatgatgtttatgtatgttt

473 L H E V D V S L C D M L N P T E M N C D V A C L V Y D S T N P R S

19111 ttacagCTACACGAGGTGGATGTGTCGCTTTGTGACATGTTGAACCCTACTGAGATGAATTGTGATGTTGCGTGTTTAGTCTATGATTCCACTAACCCACGCAGT

506 F D F C A R M Y L

19216 TTTGATTTCTGTGCTAGAATGTACCTGgtaagtatgaaaccggatcagtttggaatcgaaaatagattgttgaacagattacctaaaacagatttttaaaatatg

19321 atgctgttagataagtctgatttcattcaatatcctttgctttcattaatcaaataaactatttgaaacaatatttaccagacctagctgttgtactatatacag

19426 ttaaaattttacttactattcaaataatctgtgtgattaaaatgaattgtcttcattgataatgttttgttatgaaaattggtgctatagatttgattatttaga

515 K H F L D S R I P T L V V A A K T E Y

19531 cctttaaaatctgtaatgactatttgaagaccctattttgactgttgtagAAACACTTCCTGGACAGCCGAATTCCTACCTTGGTGGTAGCGGCTAAGACAGAAT

534 Q A V R Q D Y E L T P A Q F C S K F K L P P P Q S F S S I D K V N R E

19636 ACCAGGCAGTGCGACAGGACTATGAACTCACACCCGCTCAGTTCTGTAGCAAGTTCAAGCTCCCACCACCTCAGAGCTTCTCTTCCATTGACAAAGTGAACCGTG

569 V Y I K L A T M A A Y P

19741 AGGTGTACATAAAGCTGGCCACCATGGCTGCTTACCCgtacgtatctgtcttactgttcctggctcaaaatcctatccttatataacactactgtcacaccacag

19846 acaactctgttccttgcactattcaataatcattataatatttctcaaagtggcatatccatgtcattttgtacttgaagtgtaattttttacaacaaataagat

19951 agatttgccaaaagcctttgacactagatcattataggtctcagcctaattggagtaatttctgagtggatatttgttcattttgaacgtgatcaatgaaaacag

20056 gttcatttacacactaacagtctttgctgttaagtataattttacaatatgtggtgacatatgttatgtcaggatacaatttgataccagaatatactatgttgt

20161 gatttactttcattttcattcatttcctttttcataatttgtcatgttcattttcattgttaagttgcttcatggcaaaaagccaatatacatatatatttcaac

20266 ccttatcactccttagaaaatggcttttgatgttttatattgatgaatattttatcatttctgtctgttgtttttttttaaagatttttttattgtgatagccac

20371 caattgttctttaagacattttgaaatatgatcatatatgtatacccaggtcattaatttatagtctgattaattgatgatgaagtgttcgttaagcttgtggag

20476 atattgagaatttcatgaatcaatgccatagtcattatccatcataaggaagcccatcatgcatcctcatccaaatttcacttcaatcacataaaatttgaactt

20581 taaagcgaaacaaatctttgtagcctgtagaaatgagtaactgtagattgctagttttgtatttgatgacagacctttacgtctgggttacatacttgtttcaga

20686 acaccctgtacatgtgttacatatgtagaaacacaatgtacatgttgtgttactctgacactcaagagtacactatgtagagtattatgttagaaatggttgtca

20791 tttcaattgattttctggactaaagctgacagtatagtatgtagtttatatattagctattggtcctccaatttcctatgaacttctggtgtatagccgatagta

20896 tattgtgtaatttataagttgtagtactattgtagctattagaccgacagtttcctaagaacttcaggacttaaggcgaaagcatagtatgtaatttaaaggttt

21001 tggtacaaatgttgctaccagaccgacaattttctaagaacttctagactacaggtgaaaggaataagtttgttgtttatagaagtaaccaatgttctgacaaat

21106 ttttgttctatagctgatggattctttaatgtgtttttagatacatctaaatctgtcctttgtagtttgtgggtgtgaaagtaaatttctgaagaggttttttgg

21211 taaagatgtggtattttgtaccaattcaccaacttattactgaaataaaaattttaaaaaattcaggtaataagatttcttcttatatcagttcatgtattgtct

581 N L K R L V H M L L V R Q N

21316 gatatataatttctttacttcagtggttaagtgtactttgactcgccccttgattattttcagCAATTTGAAGCGCCTGGTACACATGTTGTTGGTGAGACAGAA

595 P L W L E E K F S

21421 CCCACTATGGCTGGAGGAGAAATTCAGgtcccctattgttccttgtgtttatattgtacctattttgtatagcatgcatgcttgttgctgtcttactgccctctc

21526 tgtaatcaccgggtgtcttgtctgtcgtacctttgggataggataccacattactatatgtttatgtatatactcggtaaatattaacccacgctacactacagt

21631 attctcttaaagaaacaagtgactataaggcatgtataaatgtatatataaacacttccaattttctgtaaaagtggaaattcttggttttggaaatacatcttg

21736 tatttgtattatgatttcttaaactgatcacatgcacagtgtggaaatttattattgtgtagacatttttagtgttaattcacttaacacagagaatgtttacac

21841 aatgaaaattataatttttatagttctccaagatcacttataagaaaagcaaatctgaatgatttctaaagccagatttgttttgaaaaattttctaaagccaga

21946 tttgttttgaaaaattatctgtaagggacaaggatcaatttcggaattcttttgtttcaaaatacagaggaagaagcttttaaaaactgagtacattgttgtatc

22051 ttgttatttgactgttaactttgtcacttgatatcgattagagaacactgtatattaaacaagtcagcccatcgttagaaattctattttgattattttaattat

22156 ttacacctttttttataattgtagatgtagaacaatgatgtaagtcttagcaatactaactaacccatctttggtactgcactaggacattgagaaatttgagga

22261 tcctatatcttcatttcgaaacatgttcatagctgaccattagatattcacaatatgtttgattttgtcacaagagtgaagtatgatacttataacaatagaatt

22366 gtgtttaccatgttttgataacttcctgccttaaagtacagcattagtgtaagcaagacatttacagatttgtgtttattgacagtaattaaaattactctattg

22471 gaagtaaagaaatgtacagatttataatagttattatagaggatatgagacttaatgtagagttggttaagcatcattctactgaagcatatatttgtgattagg

22576 aagtaagggcagagtataaagggaccataagtaataacctgaaataattaacaataggtaagaaagaatatgtcctatagaaattgtgtaaacactgaacaggtt

22681 cacatatagtgtgctgcagccttgtttagtaaaggtgtataaagagttgtaatctggaaatctgaaatttttctggagtaaatatttttatcacaatataattat

22786 gatgcatgcttttaacattgcattgttagaacctagcatgatgctttaaacttttttttttcagttttgatagatgatttttttttcatttttcaatttctatct

22891 acataatatttttttgttcaaaggaaatattctattattactatactttggttttggattcaatcatgtaaccactatgttcaaggtttaattttacctgtaact

22996 ggtctgtagttaaggtaatgcacatacttatctagtataacacttttgatagctgcatgaagttatgataaccacaccagaatgaatgaggtcacctcagcgact

23101 gaaatacaaaacattgacaatctaatactttttggcgaaactaaatgcagacccagagctagccctgccagaaaacataccttcagcataacctcttggtatacc

23206 tttataggtaaactaaatgtaatcaggacagaaatcatgtctgctctcaataaagctgcactttaatggtgcttttagttcttgttttgctgacattttgagcta

23311 tggaaattttttgtgtaaactaaatgtttgcttaatctgacaccacaggattacaaaaacaaataacttgacacattgtgagataggttaagcagaagtactgcc

23416 attgtggaaatttccacacagtacacatggtttccatgttacagatctgctttaatttgtcacaggcaatctgattgaagtgcagacccttattctcacttcatt

23521 taattaggatctgacaacatcccataaaaggtcaggttctggcaacctttgcactcccacataaaaggtgaattatctaattgacgatgacaactgcagcaagtc

23626 attctgtcacaatatgcacaatattgtgacaaaatgactcgatgtacaaacaagattagcaagcatttggccccacaatcattaacttccatgatcctgaatgca

23731 gtaaattgattgggtagtgcaaaagttcaccagaacatgacccctattgggtgttgtcagatcctacatgtacggagagtaacgaacgactatcaggatctgtat

23836 tttaaccagattgtgacaggggtcatattcccaagctcaattgtcaaattgaatgtgtcagacaccatgatttacttacatcagagtgtatcttgctgcatggat

23941 ggaaacagcttcagggtgatatactgttcaataaactgattgaccaggccccacttgtataaaacatctcaactcttgagatttcctcaactctgagatttccca

24046 tagacaatacattgcttgagattttcctcaactttgagatgttttatacaagtggacccagacctgcttgattttgcttgtgacctccttataatacagatgtgt

24151 gatttctcctgaactgaaaatctcttataatctctataacattaatttataattactgtaagcttgttgacatatttaaaatatttaagtaccacattccatttc

24256 aagccccaaaggcccttaatataaaattatccattttgctttaattctcatattgaataaacaaaaattaccatccttctacatattacattgtttaatgtttta

24361 atggttaagcaaacatgatctctaggttactttttatattaaacagaatacagtttgaagataattgtatattcttttcacacttaagatatccattgttgatat

604 H M K D V V M K E E N G Y L R V G I G V A V L A

24466 actgagaatgtgtttttgccttgtgtatttacagTCATATGAAGGACGTTGTGATGAAGGAGGAGAATGGATATCTACGTGTGGGGATCGGTGTGGCTGTACTTG

628 G V G F V L Y R I L R R G S *

24571 CCGGCGTGGGATTTGTGCTTTACAGGATTTTACGACGGGGGTCATAGtcactgccaaactttatttatagtttatggacatatgcagatgcatgttgtaacgccc

24676 cctagtggacaaaaggaagattgttagggtgtttcacacaacaagtcatagagttcactcaccataggaataacagaatgtcaattgacttggtagacagacatg

24781 gatttcataaatagtcattgaatgtgaatgctaaatatctgcagcagtaggtgtggtatgatcaaatatcaatgtgaatgtctaatctgctgatactgttttcct

24886 atttaatgtgaatgtctgaataccctcagacatagcattgatgtttaatgtaaacatctaaatatctgtggacatatgtttaatattaaaatattgaatgtaaat

24991 gtctgagtgatgacccctgttgggtacaaaacactactgagagctccctcactagtcctgtgtgatgtcaatccctggtgtagctaataacaccagctgtcagca

25096 tgataattgtctcccttggaagctgccagtatcaagatttaaagggattatttatataagtcatatatcagcttcatgcatgctaaatttaagtctaagctcttg

25201 agatatagcacaattccataaatatttattaaggggaacaagttctggaaacttcttgtaacacaaagcatatccttttatatacttataagtgtatgtcttatt

25306 gcatatatgctgtataagatacagtcagatggaattggacaaattaaattctagacatcatcagtttccaactgtgtattggaggaaacaagttttcatagctaa

25411 gttttgtgtagaactataagttatttgtttgtgtttcagtgtgtcctttaccagtccatgtgtgtattgagaagtaatgaatgaatgtctagttgggaaacacaa

25516 aacatgtaattcattaatgaattaaacacaagattatttttttacattaacttgttcatcactgtatactttaatggacttgtccacatcaaagcatggtagagt

25621 ccattaaaattacataagggagaaagggttcatttagtcattgtctacaggtgtcggtccaagtttcaaatctttaaagacagagtaatttcatttacctttgta

25726 tttgtatcttctgttgaacacaaccagttgatgtgtatcacctaatacatgtaataagaagttagtaagacaattaagtcattctctctagtttaagaagtgtgt

25831 gacagtttgttgagattgtatgcaagtcaaccaggtttttgagtctatcacattagtcttagatttttcgaagacgatgttgcttttttcatcaatttatcaagt

25936 aagcaagaaaaaattcctcattagttttaaggtataaaattgacttatggatgaggtcattggcctttgtatgtgtcagtcatgtttgtaagtgtgcagcatgat

26041 ttgtcagatacaattaagttgaattaaccaagatgtaggacacagggcactttatatgctctctttaagtactaattgcttacagttttttaccccataaggcat

26146 gcatgtgtgtgtgtttgtgactttattggtataagatcacaggtgctagaaacacaaaatcatttaatgcatttacatgtgtgatgtataccacgtacattgtac

26251 attgtataaaagttaacgttaccatagaaattatagagtacaataaagctatatacattatgcatatttttatgtgctcatatgtatattttttatgtgctcagc

26356 accagtatatataataatacatactcatataagagcaaccttaagcatatgttcctaacaatctttttggaaattcctttatatttaattgtttaaaactgctct

26461 tacatgtttaagacctatttttacccataatcattttttcttccgtcagcacatgaattattagattatctccccttggtaatccatagttcgtactggacgctg

26566 caaattaaaaaaaaattatcagatagcataagtgtgatggcagtatggtttactagacacataaaggtgtaagctggtgtgtgtaaagtttaaacgggtcaaata

26671 cgagattatacaatgtgcaatctagtcttcagagcctaaaaagtcttacctttcattaacatcacagtgtatggttgtatgaattatggcataaattaaatggat

26776 acttcatgaaatgatcaatatacttgtaagttcaaaatgagggcattcagacatgtatctgtattcagacatgtatttgtattcagacatgtatctgttttcaga

26881 catatttctgtattcagacatgtatcttgttttcagacataaaattaatctgtattcagacatgtctgttttcagacttagacatttacatgtatctgtatatag

26986 acttgtattagattgaaatgagtaagttcagattcagagataaaaatgttcagctgattggttgatttcagactttttgagaatatcattgtaaactttattttt

27091 tcccatttaaaagatttggtaacctaattttcagccttcttgcgctaagtccattttcatctctaacacatgtaaaacttatgatgaattgagttgtattattgg

27196 agaatcatattttttcgtttatatctgtatagaactttcgacttaatggtggcagtaaaacaatgtttaactttataaaactggcatgatattttacccgatata

27301 ccatatgatcaatagaaatagtgagaggagaaatattagtatatatagaatattgtgtcctatatagaggtactacacctattatgagttttttagaagaactga

27406 agccttaatacattttcgaatcaacttttaaacttgcattacgtaagaataaataaattttgatgccctaattatgtaaacttgattttgctcaccatcaatgaa

27511 tcaaaatatgttgcataattttgatatccaattcaatcttaaactatttatgtgaagtgttcggcttggtaaagcacaatcagttttcaacatttttaagtattt

27616 tacaacatatgtgtcatgtaccgtatttatccggcaataagcccatgcctggtaataagcccacccatgtctttttcatagatttcaaattcaatatgctatgta

27721 aactataaccctgcaataagcccattccccaactattgatccaatattaaacactggccccctgggcttattgcaggataaatacggtagttacaaaacaccggt

27826 aggatgtcattgaatagaatgaataaaggtataacacattggggtataatagatggtaagttgtaccatttgtcattacctgggtgttcggaaacatttctcctt

27931 tttcagtctaggaagctctctcaaacaaatattttatgtagcaacatgtggtaatacccttttctggatacctaggattgtagctctataagccacactcagtta

28036 taaaatcccatcagttcttaacatcaatttctatgctattctaaacactgaagtgccactgtcaatggtatgttgcacaatgtaccagcatgtatacaacaatgt

28141 ttcagcatacaatgtcattacaacaatgttcctgcttaaaatgtttagcctagcatacaatcttactgctttcatacaattctggattgaagctgtaaacattat

28246 gatatcttagtcactactgtcacattctaactttttaaagggtacattgtgtatttagatataacagtacagaaccatgaatataaacatggttatataaatgtt

28351 cacagctcaatactccagtattaaaggcttgttttctctctattaagtatatactcagtgtttgttcaaatgtttgtcagtctgaaactttaggatcacaaatga

28456 aacaggtgtgtaatatttattgtggtttatacaataatgaggatgtgaatggttcaaacaaaggtgatttgttttgctcttctaaagtaaacactcttacttttg

28561 ctgtttcctcaatgtttcatggtgatattcagtgttttaatactcacagggactagacactgaagtttaatatgtgatcattgatcagaatactggtgtacagtt

28666 atagtgtcatagtaacagttacagtgtcatagtaacagttacagtgtcatagttacagttacttagtgtcatagttacagttacagtgtcgttgtaacagttaca

28771 tagcaacagttacagtgtcacagttacagtgtcatagtaacagttacagtgtcatagtaagttacttacagtgtcatagttacagttacagtgtcatagtaacag

28876 ttacagtgtcatagtaacagttagtggcatagtaacagttacttacagtgtcatagttacagttacagtgtcatagtaacagtaacagtgtcatagtaacactta

28981 cagtgtcatagtaacagttacagtgtcatagtaacacttagtgtcaatactcatagtaacagttacagtgtcatagtaacagtgtcatagttacagttacagtgt

29086 catagtaacagttacagtgtcatagtaatactaacagtgtcatagtaacagttacagtgtcatagtaagttacttacagtgtcatagtaacagttacagtgtcat

29191 agttacagttacttagtgtcatagttacagttacagtgtcgttgtaacagttacatagcaacagttacagtgtcacagttacagtgtcatagtaacagttacagt

29296 gtcatagtaacagtatcacagtaacagttagtgtcatagtaacagttacaatgtcatacatgtagtaacagtatcatagtaacagttacagtgtcgtagtaacag

29401 ttacagtgtcatagtaacaattacagtgtcatagtaacaactacagtatcatagaaacagttacagtatcatagtaagttattttgtcatagtaatattgggcca

29506 tggtatgtcatgtacgactttgattgccagaaattgattttctgtctttttgtgttatgtccaaactctaataaaaattactttgaaatggaagcaaatttatct

29611 aactttaagcacaagtcaaactaatttttattttggatataaatcctgcaatattgatagaagaaattcaacttcctgttaagtagccataacgatacagcatgt

29716 ttaataactatttaattttcatgtgtcagaactattatatctagttcctgttgaagtatttaacctagaacttttgttcagaatactggttgcctccctttcttt

29821 ttcttgctctttgtaaatgttacacctgatcctaagtgaaattgtaaatgacatgttggacaattgtaatatctgtacactttgtgtgtaagtaggcggggtttg

29926 tgttagaatcttgttttgtgtatgtgtatagtttgtccatgtaaatgtattgacaaactgtacatgtaatacctaaggtaagtgagagagggattcaaggaagtg

30031 agagatatgtgtttagattgtgtgatacagctatgggagtaaacattgattgtccagatgtgaaatacaacaacagtattcctcttttaaatacagtacatgtct

30136 taactttacactgtgagtttgttttatatgttcacatttagtttgttagtgctaactataagttatagaccgtggcaaagttattttactgtgacctatgctttg

30241 acctttgaaaaatatggcaggggcatataaattgttctatttcatctggagttctagtgaccaagtctgttcgtgtgtcatatcagttaaaaaccagtttttgtc

30346 acctgggaacgcaggagacaaatggtatcactatgtcagcattggcatctgcatagaggtatccgtgctataactcgcgttcccttgggccgagtttggaacttc

30451 atccagatgttcgtcaccaaattttatttttaaaaatgaaaaggtttgaaatattgtataatattgacattgatattgcttacaacaaaaattactggaacactt

30556 aacctgagataaatttctataagtcaccatttactgatttaaaaattaacaccatcttgattaccaggtttacatttgtcaaattctcttataaaccttcttagc

30661 tgccatacaagaagttggttcaaaaattatgtagatatgattcatagttttcattaaaactgaatgccaatcataatgtaattctgtgattatgtatggccgcaa

30766 catatagtactcaactccacaatatatagtactcaactccacaacataaactgtggttattagttcatctatcccagcagtgtcaagtctattattagttttctg

30871 tagagtgtccactatataggggaacagtaatataaatgtcaaaagtcaagcatcaaagaaacgtcaattatctatacaagctaggtttctcccttttaaccaggt

30976 ttacgccatagcttcagaatatattataaacacttcatttgattgacaactaagttaaactactacattaaatttaatcaataactaacaaaatatattatcaaa

31081 atttttatttattacaaaatgtgaatatgaaaattagattgctgctgattgcatgtaaatttacaatggaattgttctacaaaacatttacattatatacaagtc

31186 aatcatcaatatttcccttatttatatctatttgcttgttatcttatttattcaaacataaaacaattagatttgaaaatagttaaacaaatttggaaagcatca

31291 caaccaagaggggaaattacatttctgctaaaacacgttatctaccagtaattcaaccataaaacaattagactttaaaatagtttcaatttggaaaggatcaca

31396 accaagaaaggaaattacatttccactaaaacatacatagaaaaaatctgaggaatgtaataaaattatattggaaaaaaagacttgatatgttttataaataac

31501 actgagttctgtggtctaacttgacataagtacatacaaaattgaattcctggtactttaatctctaatttaaatataaacaaaatcttattgccaaatccaaac

31606 tacaagatgcattatctgagttcggcctttcagaaaaacagacaagttcaccagttcctagcacttttatattttgaatataaaaa

**CfRac**

1 agatataggacctatgcctgtcagtaccagtgagatataggatctatgactgtcagtgccagtgagatatagggcctatgactgttagtaccagtgagatatagg

106 acctttgactgtcagttccagtgagatatgggacctatgactgtcaataccagtgagatataggatctatgactgtcagtgccagtgagatatagggcctatgac

211 tgttagtaccagtgagatataggacctaatcccccacctatgactgtcagtaccagtaagatatcataactatgactataggtgatgaccattggcattaagttt

316 tgggatttggtaggtgaacacatcatattatcacaatcttactgattacaggtggttgatttgaaaaaaaatagctggttgaatgttacaggttctgtaatagag

421 aatttcctagaaattagccggctcaaagatggggaaacagcaggacaggctgtttaatcagaacactgcaggccccacaataaacaataattagtgttaactatg

526 actgtcagtaccagtgagacatgggacctatgactgtcagtaccagtaagatatcataactatgactatcataactatgactgtcagtaccagtttaatacacaa

631 ctgtttatcaccaacatatcataattgtatttggcaatgtcatctaggttactatgatcacagtagcagtgagatatgggacctatgactgtcaataccagtgag

736 atataggatctatggctgtcagtaccagtgagatatgggacctttgactgtcagaaccagtgagatataggacctatgacctcctgaaatataaggtgaatatat

841 agttaatacacaactgtttatcaccaacatatcacaattgtatttggcaatgtcatctaggttactatgatcacagtactagtgagatataggacctatgactgt

946 tagtaccagtgagatataggacctttgactgtcagtaccagtgaggtataggacctatgactgtcagtatcagtaagatataggacctatgactttcagttccag

1051 tgagatatgtgacctatgactgtcagtaccagttatatatgggacctatgactgtcagtaccagtaagatatcataactatgactataggtgatgaccattggca

1156 ttaagttttgtgatttggtaggtgaacacatcatattatcacaatcttactgattacaggtggttgatttgaaaaaaaatagctggttgaatgttacaggttctg

1261 taatagagaatttcctagaaattagccggttcaaagttggggaaacagcaggacaggctgtttaatcagaacactgcaggccccacaataaacaataattagtgt

1366 actaattaacctacagatgatgatttacacatctacataattatctgaagtgaaatagctcagtgtactgtgtagttgtaccacaacacaagtttcaatcaataa

1471 cgtaagaagttaatacccctggtcagaaaatgctggacttccgtatcgagatgtcaacagctgtgccctttggtgaagatattccttgcgaacagctggatctcg

1576 ttgctgaaacagtagagaaattatctttaatagtagatactgaagtgccttatagatactgcaatcaaatacatacatagcaaatcaattgaaattttgtattaa

1681 cctatatgatactagcattaatgtaagtatcaatgtgtctttataaaggccttaacattcacaaaaggacttaaatagtctgagatgaagttttacattggtatc

1786 atgttagaaccatatacttttcatttacaatgttctcatgtcagaaccatgtaaatgcatgtcttacaatggtatcaggtcaaaaccgtctgtgtactttccact

1891 tcgcctaaattttataatacatttgtagtatcatgtcagaatcaggcatttttcactttcatcatgttttgcaatggtatcaagttaaaaccatgtactttttat

1996 tttcaccaagtttcattaaaattgattgaaatgaactttgatagcaacttttaacaagttttcaaaaaggagcataatggatttaaaccattttagttgtataag

2101 gctttgtgttctatttctattgttcatcgacttcaatcacctaaaagctgtaaatatcagagacaaacaaatgctatgctcttttaaggctttgcatttttcata

2206 ttaattttaccctatgatatgttaggacacttgaactccatatatcaactactgaaggtactcatatgttttaattttagtctggaatacacttccataaaggct

2311 acatttgtatatgataaaaaggatggtacttacacctgatttggcattctctcttagaagttggcttcttcttcggatgtataaggttccttcaaaatgccaata

2416 attacactgaacctgttactgcagtgttacaacataagtggtatccaggaaaaataagcacaatttcatttacaattcaaatgtagcatcttattaaagaagttg

2521 tgagctatataaatgtacttatcacattcagaatcaaatgtgaaatataacacagaatatatttgtaaatgcatactaagtaaacttaatatcaaggaaagtttc

2626 ttttcattttatagtcagttataaacatatgaatacctgttgggtaagcaaagtcccatcattgtgaaactgataattgatcataatgtattattttcagcaatc

2731 aatacattctcattaatgagtcaatatgtaaagttgtttttctttccacatttttgactgctataatcaggtccagcacactctgctaattgagcaatctagcta

2836 taaagacccatttagcgctaaattcatctataaattacagctatagcttctcacatttcaattttcattaaaattgcattaaacagtattaaggtttttgataat

2941 gttgacaaaatccatacatttaaatgtttattatcgtttttaataaatgttaaattttcgattttaccacgtcattattttttttacaatgtttatactgaatgt

3046 tttttgctatcaacttactgataaaatatacttttaaaagtgactttgttttacttacatttaacaaaaaatagaattgctcaaaacacctatttcaggtagatc

3151 aattgagctatttaaggtttttacagctcaatcggcagtgtagttggacatgtcaaaatgtgcatgtgggacagtagtgtactggtgggagtgactaagacacag

3256 ctactacagtgccttttcagacgaatacaagtgtattcaatatgttattataagcaatgacgagttcttactgaactgggggttgtttggtgtcctgtaagcgtt

3361 gtattttgcatctaaggcaagtgcttgttgccagactgctgccatgtttacattttggaagaaaaggtcgcttagaccatagcagaaagaggattctaaaagtct

3466 gacgatataaatatccggataaaatatcgcattatgctacacgcatggaaacgaataccagggtgattctgaattattttgggattaagtgggaatggccaataa

3571 gtaaagagatagaattgatatattttgaaacggttattttttttttcgtagcaaataaaaaaaaatgtgaaaatcataaaattacagaaacttgaattgcacttt

3676 ctttcttctatatttttgaccattactgatgagctcgtgtaacgtaatgaattaaattttccgcgtcattcgcttattaaacaggtgcagaaatagacattccaa

3781 aaataacttgattaaaacgtctttaaatagtaacatcattgatctttaaaatgtttgtgtaaacgtagtcaaaagtcaaagcgtcaaaatgatgcaagcaccggt

3886 gtcgcgtgcatatattattactcggctaagatcgtgaataaccggaaatactataaatagttcacgtttaatcgttcctatataagaggcgacagccatattttc

1 M Q A

3991 tataggtattaaactgggagaggtcggtccatataataaggccccgatacttgacatcaggattggacgttgaataaccattgtgtacaacagaatATGCAGGCC

4 I K C V V V G D G

4096 ATCAAATGTGTTGTAGTGGGAGACGGgtgagttgaaatggactaaaaatctctctaaacggtgtaactttttctcttccgtctgtcatgagctaaaaaatgcact

4201 ggcctcaaaaatgctttggactagctcaaaacgattcagcatgtaaaacttttaagcctagttctgataaagctgaaacatttaatatgatttgaagttagtatt

4306 acaattcctcatttgaacatgggataaatgatatttgagacaaactggactaattttgtgccaatgtgaactttgccaagtttacggacaatagtcactcttagt

4411 aatcacttcctgccacttgtgtgtgacttaggactgagtttggtttaatgacacgagcatcttgtccgtgtcttagcaatgaccagagagggattacagtgatga

4516 ggggttcaccagcatcaaggaaataagttgttactagttaggacagataatcataatggggtcaatagatgactaagtcagaatgaaaagccctccccagtggaa

4621 cagacctaggctattactcatctgttatgtttactctagcgtgatgagctttagtttccacatttgaccagtacataaataaatataatgccaatcagagatgta

4726 tgaaaatgtgggtattgctaaaacttgagctcttccagttgattgtacattgaaatgtttcatggtaaacatgtccatatcttatgttttgataaagacataccg

4831 tgcaataacatcacattgtagaggtgaaactttttttaaaaccatcttatttaaataagaaatcaggctatgatgaataacgctattagcagggtatgttggcgg

4936 tgttgactttagaaattccaatatcatccataatgtttataataattcaggattgatgctttataaattattatcaataatacatcaatacaaacgttgtttatc

5041 tatttcaagctgtttctcaattttaaaaagaaatagtcttattatataatcatattttttcaagacaagtccaaaggaccagttaggtaaggtggttcaccagtc

5146 cttgttgaaatctaagtaacttgttagtcataagtctagtggactagtgttagtatttacacctgaagttttcatcacaatttcccttatcccattttatgcctt

5251 ctataatgtaaaagaggacaaaattatcatggcagtgattatgaatttattaaaatatattactacaaatttcaggtattctttatattaatataattgaagctt

13 A V G K T C L L I S Y T T N A F P G E Y

5356 aatatgaatcaagcttttgactaattataattttactttgtttcagAGCTGTTGGTAAAACATGTCTCCTGATCAGTTACACAACAAATGCCTTCCCAGGAGAGT

33 I P T V

5461 ACATCCCCACTGTgtaagtattccacacttaatctcagttcataaaaaaaaaaatatccaccaaggagactacatccccagggtgtcagtatatcatacttagtc

5566 ccagttcattaattaaaaggtaaaggtttggttaattattgtttagcatcctattaacagctatggccaatggtaatttaaggatggcctcacctgtgtgcataa

5671 tgcatggatgtttattcatgtgtatattttgggagactgaaatatgtatgtgtttgtctccttgtgatagcacagaactgctgccgattttattgtgcttaaatg

5776 agaatgaacaccttcctttgttgagatgggtttatttctaatcaatcaaaggatgactgctacctgcaataccaccaatatgtaattcgtgtggatggcacagga

5881 cgtgtcacaataagaaaccgtcagcaccttcggaaatttacgccttttcaaggtaatcccacgggcatacaggcaatagatacaccaaccccgtcgccatgggct

5986 attatacttgttgttcacgtgttttctattctcactggtgacgagactgctttgttttcctgtcagagtttcggaaatcgtaaaaatttagtcaatatcaccacg

6091 agaatagaaaacacgtgaacaacaagtataatagcccatggcgtcggctcctacggtttccccgggaccctcaggagcaggtagcagtcataaatgagaatgaac

6196 accttccatttttaccatttttacagactttggtatgtcttggccagggaacaacctgaagcctgcagtaaagtattgaatgtttaataaaaaggcaaaaagtga

6301 ggcattgtcaagggagtcattaggaagaagaaagttaagaaagaagagggaaaataatatccaaaatttagtcacccctcatgtttgaattcatgcaactggggg

6406 cagcaggcctaggtactccctacctgctgggaccggttcataaaaaaaatacttcaaggggagtgacccctttagtgacagtattccaaatttacttagtagttc

6511 aacctaatatttatgatggtgtaaactcccttttatctatttggtcatggtttgtaaaccaacctatttacaataatctttgatgcctttaaattgtaaaacaaa

6616 tggtgcagtatacatgtttatatggattaagaattaataaagacagtgcaggcaaagaattggttaaattttagagtgacctgcagatatcttttgctgcactgt

6721 acttctttatacctttacatctgtttaagtaatatacatccaaccaacattcattagttcagatccattgccaatattggatcatattgttgtcatttcatatga

6826 cgcctttatattgtttgtcagttgccatgctttaagcttacacaatatcaacataaccactatcttaatcaaagacatgcttcctacataatcagaactaattca

6931 tcaacgaaacacatttctgaatggttgtctggtacatgtataattgtgtacacaacattattataatacacaaaatagatacaacatttgtaattgaacctcttc

7036 aaagttttttctataagaataataagataaaatatactatttccctaaaatttctccttctgagagctactccctccctaaaatagaactactccctgccagaca

7141 ggattactgtcaggaggaggactactctctgatgaataatatttttaggtggagcttatataatttaaaccaatgaaaatgtttggtaggcaggacgatcgacct

7246 cctccatgaagcaagtaacattacaagtcagaaccacatgacaagtgaaaattgctagtgtactgtgacacaggctgtttgatacacgtcatgttactgaggaat

7351 tgcggcttattttagatgctgaaagtgtagcttagatacaacggctgtttagactgtagaaagttgattttgaacttatttaattgaatggaaagctatgactgt

7456 ttaatgggtttacaaaaacatatctcttgaatatttgaaataaagttattatcattgatgatgtattcagaacttccagtggtatttttcctttatgaaattgtt

7561 taaagaaataaattattttgttgaactgcctattttttcagtataataaatcagttatagaaaaggtaataaattattttgatgaactgcctatttttcagtata

7666 ataaatcagttatagaaaaggtaataaattattttgatgaactgcctatttttcagtataataaatcagttatagaaaaggtaataaattattttgatgaactgc

7771 ctatttttcagtataataaatcagttatagaaaaggtaataaattattttgatgaactgcctatttttcagtataataaatcagttatagaaaaggtaataaatt

7876 attttgatgaactgcctatttttcagtataataaatcagttatagaaaaggtaataaattattttgatgaactgcctatttttcagtataataaatcagttatag

7981 aaaaggtaataaattattttgatgaactgcctatttttcagtataataaatcagttatagaaaaggtaataaattattttgatgaactgcctatttttcagtata

8086 ataaatcagttatagaaaaggtaataaattattttgatgaactgcctatttttcagtataataaatcagttatagaaaaggtaataaattattttgatgaactgc

8191 ctatttttcagtataataaatcagttatagaaaaggtaataaattattttgatgaactgcctatttttcagtataataaatcagttatagaaaaggtaattcatg

8296 aaatagcaaatttatgtttccctttgttaaaaaaaatctatttatcttggcatctttcccacatctggaactttcagttttacacctcaggggactgattcgcta

8401 gtttcctcatactcattcaataactgataactgtataatagagtacactatatatatatatatccaaattactaacataagccaccctagaacactctacagtgc

8506 aaagatacatgtacacaattagcaatacaattcttgaggaatttacaggagctggcctgccttatcaggtgctaactgctagattagtcctatgccacctgacta

8611 atatctctgtgtttatgcctatgtgtgtggttgagaagtaacatgtaatatgtttgtagtgggaccatgtttccagcagtgtatttacaatattatttgactgtg

8716 aagagccagaggtgtttttaagaaaactcatacagttattggaaatccgagtataaagtgttttcagtaagaattgaagcaaccattcagattaacaacattttt

8821 gtgacactgggttcgtatgtcatagccagacttcctactaggatatttatttataatgtactatagttggtcacattttagaaggatgtttatcagtaatacact

8926 atacaattttgccttgtactttaccccattacctggtcactaatcacgatcctatttcagtacaggttcagtgaaccatgagttatgatatttgttgtgattgtt

9031 atttaaaggtatacgtcaggtgtaatgtaaacttcctggacattttactaatcaatacagggtcacttatctatacatgtatctgttaaccagatataggcggga

9136 ttcccaacatgtgttatatgtttatatcctgtgccagatatgcttactatactgttgtgtgtattctggttcatattaattttaatatctcagtgaaggtggagt

9241 aacaggtgtaaaatataacacatcgtacacgcatatgtagtcaatgtatacaaacttcatattcagagcaattcagtttttattctatttgaccatttatcagat

9346 catctatattaaaggaaactgaataacatcattgttctgtataaattgtgaaattttttataggaaccattttggtttgttggacgatatatatataacccatgc

9451 atatcttgaaacatacatatatacatatatgtacacaatattgtatactaaatggagctgaaatttacttcctgtttttatcagtgtgtgtcgcacatccgagat

9556 gattgtagcagtgagctctgataaagatatactttaaataccagaagtaagaccaccagattactgctttctagattgagtcaacatataggtaccataagaact

9661 atctaggaccaactacatatagctactactaggactatgtaggaccaactaaatataggtaataataggactatgtaggatcaactacactctaagactttgtat

9766 atgtaggaccaacagctttgcttctttttcaaatctagattataaataggactatatagtactatgtttttgcagagactataaagaggactacatgtatataag

9871 attatgggtggtttctaccagtaggactgtgtctgggactacatgtatataagattatgggtactttctcccagtaggactgtgtccgggactacatgtatacca

9976 ccctatgggtactttctaccagtatgactgtgtctgggacaatatgtatataagattatgggtactttctaccagtatgactgtgtctgggactccatgtatata

10081 taagattatgggtactttctcccagtaggactgtgtccgggactacatgtataccaccctatgggtactttctaccactaggactgtgtctgggacttcatgtat

10186 atataagattatgggttctttctaccagtaggtctgtgtctgggaccacatgtatatataagattacaggtgctttctaccagtaggactagactgtgtctggga

10291 cttcatgtatatataagattatgggtacttcctaccagtaggactgtctggaactacatgtatatataatattatgggtgctttctgccagtaggactgtctggg

10396 aatacatgtatataagattacgggtactttctaccagtatgactgtgtctgggactccatgtatatataagattatgggtggtttctaccagtaggactagactg

10501 tgtctgggacttcatgtatatataagattatgggtggtttctaccagtaggactagactgtgtctgggacaatatgtatataagattatgcgtgttttctaccag

10606 taggactagactgtgtctgggacaatatgtatataagattatgggaaatttctaccattaggactgtgtctggcaccaacagcatcactgtttcatacattgact

10711 gtactgtaacagttatgacaattaatgtgttccagcattgattgtgcttgaaagcctaagtttgacaattcaacgtcagttatttccgtgactagtctgactaca

10816 tctaaagacaccctagttacattgtacagtatatatatgctcattctgaggactaacttgtgtgataatagtattactcaggtgtatattgtacaacccatcagg

10921 aagtttgtttatatacatatataaataatgaaaaaggtacatattgatttaagtatttaaacaaaacaatgttgtaggtagttacaataatctgttcaaatcgtc

11026 attgtacacataaattgtggtattcatgtatttttttcatatatatgctacttgtgtatattttttaataaaaatgtttcaactgaattgttgactcttgaatag

11131 aattagtaaaataaatttaagaggtcacctatatatggttttaatcaatctggatagctaaagcatatattttaactggatgaatgtgtgtcaaaatgtaatgca

11236 gtcaggacattgtagtctctattgtaagaaacaaaaacatcttgacaagatctgccatacttgaagtgcttccagtctttcacctcttttaatgccccatggggt

11341 tcaatgtttttatttgtaaaatgtctaaacaggacaacaacacaaagtagaaaatgatgacgtcatactgtacttaaaaatgtatgatgaaataatgtactcaca

11446 gacaacaattatgtcagggtaaacaaaaagctgtaacacacctgtatatttgcatgccctatacccacaaggtaataatatcccaaatcatagttattttgataa

11551 taattattatatcgttttcaacaataacatatcgcattacagttattgaatatcagtatttaattatttttttgtcatacacaagaaatattttatacaacatgt

11656 aaaagccaaaagcatctcacattaaaggaaattcctacctgttatcagtttaaagtttattctatactaatgatctaacaacctacctatagtgatcaggattta

11761 ctgctgcaggagggtggacattagtcctggtatctatattaatagtgagaaccacctgggaaagggttaaagactgtcgggacatttcagaaaacgtcacataga

11866 tatactctctatacgaaaatcattttaatcttcaacctttattactatatctctatattaaaaatgtaaatccagccttaatgctctactagattttgagtattt

11971 ttcaaacaaaattctaaatcttttaccagccagttatatatttaccatgcaattttcaattttcctgcagcatagctgctgctttggaaaaaaatggttcaaaca

12076 acgaagcaaaataggagatagttttcaaagaaatgttcaaagaaatgtacatcaaatccccagagtgagagcagtcagaccttaaaagatgacaggctcttgtaa

12181 aaaatgcctttgtgtaaaatatgcttggcatgctcctgtaaattattcattgtttgaatttgcgtttatcctttactttttaaaatctccttcataatcagtata

12286 ccacacatacaagaaccataacattggatgataccgagagtatttgttgattaattgatgatgcttggttaatgtgttatgagaagacagtgtggtatatcatac

12391 acaggtccttactacatggccaagattatatcaccgtgccattgtctagacttcataaatataatgattggtgccaaacgtttctaggcattgaatattaatgtc

12496 atttatcacacagttctttgtggctaatatatatataaaggtcaagggttaaaaatcatagaattacagtttatcaggcatattatagggagtattatagccaat

12601 aaccttgggttttttgaaggtatcttattctgctagtagtggtaattgtttatacaataacatgtaatacatttgtaaatgtatatctgtactctatattaaatt

12706 tatgtagccttagtttatgtatatacatgtatggtattcagtgttttgtttgatgttgaagcatatttgagttcatttagactttcaagttacactccgagatgg

12811 gcataattatggaggtattttctgaaacaagtttccaatcaagttaataccgtgtggttatgttagaaacagctgggtctcctaccactacactccaacacagct

12916 gtatagtagtgcacagcagatcagcgatcacaggccagcacatatctcaatcactcaaacacagctgttttgccacctcagttttgaatagaaacaccttggaaa

13021 attaccatacacaatgaaatatcgttttaaggtactctctgtatcagcaggaaaaacgttgatatatgactgaaattcatatatacagattatttcccttacaga

13126 tttatgcagcagttaaattattgtttgtggtgtaaatacgttcaggttatttttaattagtcgtaacaaacttaattatggtgggagaatgtcttcgtgaacagt

13231 ttaatctacaagcaatatatgtgatgcacacaatactggatttattcatactcttaaaatatcatgaaaaattgaaatctattccatactagatgtatatgtaag

13336 tctatggtacagattacagtccttttaataccttaacatttctaattaatgataagtcatggcccacttagatatctgtcaggagtacttgtggtaaacagaata

37 F D N Y S A N V M V D G K

13441 tcaaaactccatggtaattttaaaatacctctgttagccattaaaagtattgcatgtttatttacagATTTGACAACTATTCCGCCAATGTAATGGTAGATGGTA

50 P I N L G L W D T A G Q E D Y D R L R P L S Y P Q T

13546 AACCAATTAACTTGGGACTCTGGGATACAGCGGGACAAGAGGACTACGACCGACTACGACCATTGTCCTACCCCCAAACAgtaagtcaacttttacaataaattt

13651 ccgtatcaatttattaatagcctttttttagcatttaattgaattgattgtctcccataattattgttaagtaactgttacttaactacagttacataaattcaa

13756 tcttgattcatttctatccagttgcatttgattgtaatccatatttgtaattatttgcttgaaattcaaatcacattcagaatatttttcacctttatttttgca

76 D V F L I C F S L I S P A S F E N V R A K

13861 gGATGTATTTTTGATCTGTTTCTCCCTTATAAGTCCAGCAAGTTTTGAAAATGTCAGAGCAAAGgtaagttacatcctatttatcattactattgaaactagtaa

13966 ctctctaaaatagtattggaaattagaatgtgttccttggtgatcatttaagatagacagtatgttatgggagtttatgtgtccttggtgattgtttatatgggg

14071 agtatatatatgtgttccatagtgattttccttgttttctcagataaagaaaacttattgtctttaactcatatattaaactaaggtacatgtgtaattaatgtt

14176 taaacattttgaaaggatgctatgaaacaattatccatctttaaaacctgctttaatgaaaggttatcgttcatcttttggttttgacacattttgaaacaaagg

97 W Y P E V

14281 cattttattgacattccctattacagtcaaagtgtacattgtcagaactggaaagatatctaattgacttacaatatgtcttttgtttagTGGTACCCAGAAGTC

102 S H H C P N T P I I L V G T K L D L R E D K E T I E K L K E K K L S P

14386 AGTCATCACTGTCCTAACACACCCATCATCCTGGTCGGAACAAAGCTGGATTTGAGGGAAGATAAGGAGACCATTGAGAAACTGAAAGAGAAAAAGTTGTCGCCA

137 I T Y P Q G L A M A K E I S A V K Y L E C S A L T Q K G L K T V F D E

14491 ATTACCTACCCTCAGGGCCTGGCCATGGCCAAGGAAATCAGCGCTGTCAAGTACCTCGAATGCTCAGCTCTCACCCAGAAAGGCCTCAAAACAGTGTTCGACGAG

172 A I R A V L C P K P K P K N K K K C I V L *

14596 GCAATCCGAGCTGTCTTATGTCCAAAGCCAAAGCCGAAAAACAAAAAGAAATGTATAGTTTTATAAttacataaacatttattcaaagaattataagggagggat

14701 atcaaaacataaactggaaaacaaaatgagtttgaagaagagtatggaactccattgtttcataccttgttattgaataggatacaaagttcaatgaaaccagga

14806 gatatattatggttacatggatgagcagaatttgaaattaattaatgaatttgcatcaacaacatgcatatatagctgcttgatcagcagcctatggtaaaatct

14911 gtataaatcctgtatcagttttatatataaaatgtaatcacaagtatgagacatataatatgcagcattgtatgcaatgaaagttaacttgccttggtaggagtt

15016 gaaatgtaggacttgtatgatgacgagtactctccgggattaacataacaccgttaattgtcgtacatgttgtggatctgcaaacaatgtttaagtgtccttctg

15121 tcctggctgtaagctcagctttcatataaatgtgctgctaatctgtgttagatcataggcttgctcacagtgtttctttgaaatcactatctatcataagtgtta

15226 tttctgtttagaaaactcattaatgtttcattcatagttctttatttttattaatctgcttatttcatagatgatgttcgtgctaagcacaacttaggctatgat

15331 gtgtaacatcacgaaaaataatttttattggaagtttaacaacagtttttattttagtctaaattgtgttcttttggtggccatatgtacaatctaaagatgtat

15436 caattataacatgcatcaattaggaacacatttaccaaaaacaaatcctagctttttagaagcaaaattaatgtctttttatgaaatttatgttgactgtggcat

15541 tacatttgtatgtggtcaatagaattaatttatgtgttagtcttttagcacagcttcaatcgttcacctgtttgtgtttgggcagtaggtggccatacaatatag

15646 tgtggtaaagttactcactttctacacatcatttgtttctttaacttctccctttgaagtgatgttctaaacctgcttagcatataatatttctgcttcattgtt

15751 gcaaacacaaacatgtttgaagaaaggggattttgacatatattcatacttattttgattccatttaatcgtctgactgatgttttagatatatagatagactct

15856 gtacttgttatgtttcactttgtaaataaaattttactttttaaatttaattttttgtcctataaatctcttgcatctcttacattatatatatgaataaacttt

15961 tattagattataaaagtacatggcgtgtaccaatatatttacattgtgcatgttaaattgtggtgaagatgttagagcgaacagcatacatatatatgaatgtgg

16066 tcagaatatacatgtacttatattttcaaattacttgttcatacattaaaggtcaacaatgctcattttatctttaaaaaaacccaacaactgatgatcctccac

16171 agcattgtctatatctcgccaaaggaactgcaaacaaaataaaaaatacgacaaaaaattaacacactatttgttattgcatgtgatatttatgttttatttaaa

16276 aacactatattttgtttgagtaatgttctttggcagtgagatattatgaaaaacacctacactttttagtatgttgaattattttgggtaacttttaataagatg

16381 aattgctgtagccatggaagacctattacataatctatatacagatttcggacaaatgcagcagtcaaagttataaaccttttcgtagagacacttgaaatattt

16486 tgaaaattctcaccaaaaaaaaatgttttgcaaacattttaattttttttcatttaaattgtatgaaatgttaaatatatgtgttttccatgaagtgagttggca

16591 ttagttaacagctgctgtgtaagacccatttcagttgtttcatgtgtgctgttccgcaattgttttattaaagtgcttggttttacactagctatataccacact

16696 aaaggtgtataacatgtttgtatagtttgtactattgtgtattataatactgtatagctttataaggtttaagtaaaagcaagccatgtattgtcttctttttca

16801 tcctggagatgagttttctttccatcgttttgatcccatagtaaacatttctctcactgttcttccattttttctcatgactgcgtagtggtattcagagatatg

16906 tatctcttacaaaaccactctaaggttgacagtatgaaaatatattaaggtcaaagttcaaggcatcagaacctgacgtcatttgtacttataaatgacaaactg

17011 gttttctatcttatgctcaatgtatgttactagaatattcaggtgttaagagtaaatgatatacttcagttttataacataaatagtaagaacctgtaaacggct

17116 aatgttcattaacaggggagactaagcaatgataatgtaaatgggatatattgggacatatacactgatatacagagaagttgtaactaattatcattaatattc

17221 tgtataacagccaagtctgctacaatttcaaaggcttcttcccagacttgttgccagaatggtaacagattttaatcatttatagttggtatatagatttctaat

17326 ccatctctatgttaatgaaaccatagcttaactaagtattaatttaatgagagctacatcagtgttttagttgtacattttattggtgctttctatgtttaaggt

17431 cagagatctgcctctactcctcagatgttgcaaacttaacaaagcaaacaccctgagattgtataactgctgcgtacaattacttcaatatgaacaaatcgttat

17536 cagcatccttgtattgaaccatatgaggtggcatcattgtaacttactaacaagtcagcagcagttttaatgtcgactgtaaactgtgaaaataacttctatttt

17641 atctttcgctgtaaagggagtaaatttctcttcatgtaaaggggtaaattcttttctttatgtaaaggggagataatttctcttcctgtaaaggggataaaattc

17746 tctccataaaaatgggataaaattctgtccttgtaatgggggttaatatctatccaaacattaaatataagctaacaacatacttgatgtaattatttcaaatga

17851 agagtgaaattaacttcaggcgaattgacaaattaatctttgaaaagaagaaaattaaaggcaaacgttttcaagcatacaaagacatagatcatttagactgcc

17956 agtttttagcttaacatgtggcactgttttaaataaattgaactgaatgctttatttttcttcaaaatgaaataattttatgttctgatgactaataagattgtt

18061 ttccttttgataatgttttaattgggaacagtttggcgacacattgacgtggtagtaggttatgatttagctggcctacattaagtgattgtctctcccatggaa

18166 tgcagtgttagcacaatgccagacatattagtattgctttcaacaatcattggagagattgtgtagataaataatatcatgtgtaacatacaaatgttaaaattt

18271 cagctctttatgtaataaaacgataatacttgctaaaaatgtagttggtgtttttattagctcacctgggctgaagggccaagtgagcttatggtatgatccggc

18376 atcaacttttccattcaatcaacacagggggtcaaatgtctttaaaacttcaaacaacttcttctcagtaaccaaaagccccaagatgctgatattgcgcctgta

18481 gcatgctgggatagagggctaccatgtttgttcatatgaatgactttgacccactttcaaggtcacaggttaaattcttttaatcttcaaacgacttcttctcaa

18586 taaccaaaaggccagggtggtgatattaggtcagattgctgggatgaagggctaccaagtttgttcaaatgaatgaacttgactcactttcaaggtcaagggatt

18691 aaatgtctttaaaacttcaaatgacttctgaatatcgaaaaggctcaaggttctgatacagggttcaaatattttaacaggtcattttaaatgacttaaattata

18796 ctgatacagcgtttgaagctttatttaagatggccttttaccttacaaatatgaaattaataccaggtgagcgatgcaggcccattgggcttcttgtttcttatt

18901 agctatgatcccttctcatgtacataagacatgtatttcattaaccacttgatgaattatgttatccacttgatgaatttgtaaaatttgcaaaaaatataacgt

19006 tggttataatgccatgttagagtttatcctataggtgtgtcacaacaaactgcagtatagatggaatgtattgtgatacaccaggacataacaccagttctatat

19111 acagactctttattatgtactgtgaacaaaatttgggcagaccaatactctggatttaagagatcccagagagctcttggctcccacaattggatgatctttatc

19216 agccctaagaacgactgatcttttctccactttcccttctaccgcttaatactttgataacaatgtgaatctatatgtgaagtctaagaaaaagctaagaagaat

19321 taatgtcagaaatctcaataaatttccactatcaaattttaaaataagcacatgtcggcatttttgtttccttgatcaaacataaaactaaataggcacaactag

19426 aaaccaaggtgaacctacatatgaaatttgagaaagagctgcatgtctagtactaagaaatagcaacaacagatattcattatcaaaatccaagatggctgcctg

19531 tcagcaatcttgtttattggattagtgtcaaaagttgtcctgcacatctaaggaccaagaagaacctacatataaaatttaagaaagatccctccagtatttctg

19636 agaaatagtgatgaacattcactatcaaaaaccaagatggctgcctgtcatccatcttgatgatcaaatcagtttcaaaagttttcctgcatagcttaggaccaa

19741 agggaacctacatataaagtttgagaaagatcccttcagtacttcttgagaaatagcaataacaaacattcactatcaaaatccaagatggctgcctgtcggaca

19846 tcttgtttatcggatcagtctcaaaagttgtcctgcacatctaaggaccaaggggaacattcctacaaaatttgagaaagattcctccagtactttttgagaaat

19951 agcaataacaagatttttttacggacggaccaaggaggcagggtgatttgaatgaaatcaagcaaagacaataaaaacaagcatactgagtattgctgaagcaat

20056 acatgtcccctaccggcccccacaaacaattaagatgcaacagtgacctcaacctaaatgtgaggaccttgaaactcaaacttgtccaatatattgaaattcttt

20161 acaaatgtgtgaagtttgataaaaatccatgagagaatgtggcactagagtgctgacaaagatatttttttaaaatagtaacagtgaccatgcccttgccccgat

20266 taccttgaaacttgaacttgtccaatatattgatttttttttacaatagtgtgaagtttgattaaaatccatgaaagaatgaggcactagactgctgacaaagat

20371 tttctaaaaatagtaacagtgaccttgtattgccccaatgaccttgaaactgaaacttgtcaaagatattttaattctttatgagtgtgtgaagtttgattaaaa

20476 tccaagaaagaatgaagacgctagagtgctgacaaagattttctatttatagtaatagtgaccttgaccttgatccgatgaccttgaaactcgaacttgtcaaag

20581 gtattttaattctttatgagtgtgtgaagtttgattaaaatccaagaaagaatgaagacgctagagtgctgacaaagattttctatttatagtaatagtgacctt

20686 gaccttgatccgatgaccttgaaactcgaacttgatctgtatttccccaaaacaaattcataccccaaatttcaagtcaatatctcaaaggacaacagagaaaag

20791 tccggaaaactaaattggacggattttctatttatagtaacagtgaccttgaccttgatccgatgaccttgaaactcgaacttgatctgtatttccccaaaacaa

20896 attcataccctaaatttcaagtcaatatctcaaaggacaacagagaaaagtccggaaaactaaagtggacggacggactgacagacagacaccgaggaaacctat

21001 agtccccccccggtttcactggtaggggactaataaaataggaacagtgaagtaaatgtgagataacaacttgaagacatatcaagaacccaatcctttttatta

21106 atggacaaagttgtttgtatcacattcactcatattattccatgcagttgaagttaattgatgcttatatatatgcttgttaattttgccatacaaaataaaaag

21211 gggagttttcggacaagcccaatttaataaaaataaacctaagacttcatttaatcatgataaattttagcaagtgcccagtatgagatttatggaaactgaaaa

21316 cttgtgagctcttcataaaacaaaacacaaaacatacttgctactcttaacagatttaattcataattatacttatgcatacataataaacactcgtgaaaagtt

21421 ctttagccccacgtttatttcgtcactcatccatttcagaggcagattcaactttcaatatcaatttaagagttttactgtgatcccaataaaattcacacaaat

21526 ccttcacaaacagaaggagaaacactgtggtaagtctgttatatcaagtctattgtcttaattgttgtgatggtactctacacagcgtcacacttctctcatcta

21631 gtgtctgaaacagaaacaaagcaagcagttgaaaattaatctttaaacaacagaaaaagattcttaagtcttcaggcacttgtgatcataaaactgaagatacca

21736 tgtaataaaattaagacaaattataaattatacagaaggctaacagaaaccccctccctccttaattccagtctagaaggcctccaagactctccccagcattgt

21841 gtttaagctaattagacttttttcaagataacttcaataacaatttttatgtccccgagattgaagatcgggggacatattgtttttggtcagtctgtttgtctg

21946 ctttagggtttccgtcaaattactaaagttttaatccatggatttcaaccagatttggtttactgcttcatattaacaagatcttggacgagttccataatggta

22051 aaaatttgtcaatatttacacaagttatgggactttgaaattctaaaaatcgtttatttctttgtttccggcaaataactcaagttgtaatcaatgaaattcaac

22156 caaatttgggatactgctttatatcaagaagatcttggacgagtgcgataatggcaaaaatctgtcaatatttacaggagttttgaaaatcgtctatttcttcgt

22261 ttccgtcaaattactaaaattttaatcaatggatttcaaacagatttggtatactgcttcatattaaaaagatctttgaattcgataatggtaaaaatacgtcaa

22366 tatttacaggagttatcaaatgccctaatttatgtatgcacatatgaatggctatcccttgggggcatagtgtttcacaaacacatcttgtttaaatgtagattt

22471 gttgttttgaatttatccacggttaaaatacattaaaaatgattttttgaaagctggtatatgtaaatgatacataaaaattaggtaaaataattaatgaaaata

22576 atactgtatatggataggtattcagctttgtatgtaagttacgaaatcttatttgattaccttttccaactgaacacacacagctttagttatgtataatgcaat

22681 tgtgtgtaaacattttcacaat

**CfRho**

1 gtttctggcgtcgcttgaacgtttgattccgtcagatatttgcattaagcactaaaacaacaccgtcaaatacttcccatgatgcttcagacaaacatccgactc

106 ccacaattcctgagcaattgatttacccctctaaggacaattgaggtgtggtcggtgcttgaatgaatcagtagatgaacgtttttacttagttgaaaccggtaa

211 attacactcggtataaggtaagaaattatcatcaacacttttgtaatgtaaactgaaagaaagatgatcgataaaaatccttgaaacaacgaagattattagaat

316 tgtggatataattattcacttccctatttggcaatgacctcagcatgctcattgtcattacaatttgacggcgaattcggttataaaatgcattacgcgttgcca

421 gatcttgtcatgtaatcattaagctgtcctaatgagtaacgatcaaatgtgtatgtgcgatagaatacgtacattgcgtatgtttttgctgtggatttgatgttt

526 gaattccatattcgccatcttgatgacatcatcgttgcatctgctgtctactctagaatgtctggaaaaccctcttttctgatcttattcagtgttgtacggtca

631 ttttattgatctttcgttttggtatacattgtttggaactataaaaactgatgcagcctattaaaatgttttggatagtgcaaaagatgtgttcggcttcactga

736 aagaggaaacctggtcgacagtaccctattaaataaactagagtgctacatccagtactagacgtagaatcagaccagatgatgtttttgtttggtagccctaga

841 tgaatattcgataaaagtagctaactgtgattttataaggtcaatttgttggggccataatagatagaagtatttgtctggtacatgccatggtacatataatca

946 tcccaaaatctggacaaaactttttttatttccaaacatttattttagctggatattgtattttctcccgctaacagacacattccccaaaaggaaaacaaattg

1051 tttttgtgtagtcatgatttactaattggataaccagctcagagaaatcagcccttgtgaaagtgattagtacatgagctttgaaccagcatatgcaccaaccag

1156 aaaaggttgtttctgatgacaagataattaactagtgtcaataaaatatttgtttaatctttattattaaagatttgaatactgattattcataagattggtttc

1261 attaaacagactgggaataagcaatcatttgtacatggtgtcccgcaccactaagatcgttcttaaataaggtgtgttcccatttaaaaaaataagtgcataatg

1366 tgagcagagaatgttttgaacacaagttttattattgttcgaccatattgtattgatagttatttttaccacccagagaatgtgttttaagacgagttaaatatt

1 M A A I

1471 cctttgtgcgaccatattgtatttttaaaatagttttaccactctgcatcacatgctatttcttattctgtatttcagattttgatgttacagacATGGCTGCTA

5 R K K L V I V G D G A C G K T C L L I V F S K D Q F P E V Y V P T V F

1576 TAAGAAAGAAGCTGGTCATTGTAGGAGATGGTGCATGTGGTAAAACATGTCTCCTGATTGTGTTCAGCAAGGACCAGTTCCCCGAAGTCTATGTGCCAACAGTAT

40 E N Y V A D I E V D G K Q

1681 TTGAAAATTATGTAGCAGATATAGAAGTAGATGGAAAACAGgtttgtattttaagtgctgcttaataggtctgttgggtaaatttttgacaaggggaacattgtg

1786 ctggttacagatatgattgcttcagattggtattttagaaatgtagggctctgctttcttgtaaatcgttattctaagatcaaattctcatatttctacaggggt

1891 aatatttccagtatgtattcttatgaagttgtgtaggatttgcaattgcatgcagatttcaatagtaaaatagtatggtactctcagatgataggtagaagtgtt

1996 tgctttcattttgctccaattgatttattgttatgtattttttcatattggtgcttcagtgtctagactgtgtgcagtgtaaaacaattgtattctttcacatgg

2101 cttccaattttatttttggaatatttgcacatttgctctcacctcatcttctatgtacattttgtagtggttgtcaattttactcaaggcaataatggtaaaata

2206 tttgaactgagataatcatgttatcttaaactgtcaaatcactctttttcgcgaatttttcgctcgacttgtttcctacaacatgaagcagaccgacatctgaag

2311 gataaattataattgaaacggtgagttcttttcaatgtctgcatgcagtaaggatttgtaccagctgttgaggattgttgtccaaatgagatcttttaatttttt

2416 ttttctatttttttatcaagttgaaactcgaaaaaagaacgtggacctttcatttcatttttctgtcattttactttcaacttttatctcatatattttaaaagt

2521 ttcatttcacataaaaaattatttggctatgctacaacctaagtattccagcacttcacttaagttgcattatcagttttctttggttcacacttaatataaaaa

2626 accagattaatgatactacttattgaggatcaactgaagcgtttttaacaatgttgttaagagaacaatttaagttcagaataacttttcatgttttgtaattga

2731 aacattaactgtaaatataatatttaggtagttttaaaagacatccaatcttatataaaaaaaaaaactgtgaaccattagaaaaataaatgttcgttcaggtaa

2836 actattgcatgattgttgttgtaacattacagttggggcatgtttttaattttgctgcttatgtttcaaatggctttgagcttgtgcttcagttctgacaaaatt

53 V E L A L W D T A G Q E D Y D R L R P L S Y P D T D V I

2941 tgctcaccttgtgtcattgcagGTAGAGCTGGCCCTATGGGATACAGCAGGTCAGGAAGATTATGATCGACTACGACCACTGTCCTACCCTGATACAGACGTCAT

81 L M C F S I D S P D S L E N I P E K W T P E V K H F C P N V P I I L V

3046 CCTAATGTGTTTCTCAATTGACAGCCCAGACAGTTTAGAGAATATTCCAGAAAAATGGACCCCAGAAGTGAAACACTTTTGCCCCAATGTTCCTATTATTCTTGT

116 G N K K D T R N D E N T K R E L R K M K Q E P V K A S E G Q S M A E N

3151 TGGGAACAAAAAAGACACTAGAAATGATGAAAACACAAAAAGGGAGCTACGTAAAATGAAACAAGAACCAGTCAAGGCATCAGAAGGGCAATCAATGGCAGAAAA

151 I H A K A Y M E C S A K T K E G V R E V F E T A T A A A L S T K K R R

3256 TATTCATGCCAAGGCCTACATGGAGTGTTCAGCCAAAACAAAAGAAGGGGTACGAGAAGTGTTTGAGACAGCCACAGCTGCTGCCCTGTCAACCAAGAAAAGGAG

186 R R R F C K I L *

3361 AAGGAGGCGTTTTTGCAAGATTCTGTGAacaatataagattcagaacttttcaaatgcaaattctagaacttttcaacgcttgtcttttcaaaaatgaaaaggtt

3466 taacatcctttttgaatagtgcagtttgcatttgattaaaaatcacacctatataatagtatacacataattgatgagtgtgataagattgcaagtactatggag

3571 atgtacaatttcaagtacaaggtgtatggtgcaagcagtgaagttgttaatttaagctaaaagcaagatgatagagttttttgatattttcagtcattttgtatt

3676 gtattgtgaaaaatgcagatagaagctttttgtttttggtacatgttgtcttggatttacaaaaccatcctgagatatccctgatttttatgcattttatatttg

3781 taaaacattaagatgattttaattccaagggagagattttattaggcaaagagtgtagaagattgatcaaaaagtactgttaaagttttaacattacaccagttg

3886 caaatgtgatttctttcttcaccataaagagtctttctctttaataatacgtttacaataacattctaatagtaattctacacattccagtttttgacttgtaaa

3991 tacaattgttgttttgtgattatatttatttcaatcttgtataaactaatgaaatttagttttaattttgtgataagttgataaccagataagtgagattaaata

4096 catgttctgtcctgaaataatttagaggatattgaaatatatattatgtaagatctgattttatttgtctcagactctaaacttgtagtttcatgttaattttca

4201 ttttgtggtatttctgtaaaaaaaattgtaaccatgctcagtaaagcaatttgtgtagtaataaatttgtatttcgtccgaacatactgt

**CfRhoU**

1 atttgaaaatttacaaaacattacttaacaaaattatctttatctggtcaccgacgatcgagccagtccagttccgatacattcctcactctttttctaagtctt

106 cgtctccgaagacgacaactatttacaacccaggtccgggtatccccccttaccaaccatacaccaaatgcccgaaacaagggttggtctcggcatcactttacc

211 aggagatgaaatgccaaaccagggttcgaacccagtaccttccgttcgccgacgatcgaccctatccagttccgctacgtcttttaactgtatgtttttcatcga

316 aactaaaacccggatcacatcacgcgtacaggtccattatgacttttaacctcagatttttagaaaatggtgatactgagccagatcgatcagaccaaaccaagt

421 gtccacaaggaccaaaggttaccctggcaagtgtcacttcagacgaaataatgaatcatatttcgtggtaatttatttcaaagccagattctggggtaaaatact

526 tcctgttgttgataaaaataaaatgcagtgtatttcatttgaaataaaacatagcatttttcgaattcttaaatacacccaatcgtcaaaatgcagttgtttatg

631 gagaagaatatagtttgttttgctgttgcacaaacaggtggcgttcttatggaaatagtatacatttctttgaaagagaaactgcggttcgtattgagttacgag

736 gcagatgtaaacattgggcttaccacatctgatatttgataatttttgtgcttgtgtaagagctaaacccactcggtgttatcagggaagatttttatcaacatt

841 tgaaaagttaagccggtttggacgtgtacagcgataggtgtgcagatcgaactatttatataacaagtcgaaagtgaaccgactacataacaaatgaattaatta

946 ctactgctcaatgtctgtctagtttctcacgtgttctagaatacgtcaccaggtatatgctgtgcacaggtaagatatatatacgctatgtaaataaagaaaact

1051 tgaatgactgttcaggtgagtgttagaagggcgttgagaaaacgttttcaaagaactcggtcatccgttcttatagaccaggacacttgtacaagacttctgtaa

1156 gaccgacagcgcacaggtatatatagatatatatatataatatagggtttgaatgaacgacaatgttagtcatagcgttcagccaatcagaaaccgcgacacagt

1261 gattcatagattgacggtctcgatggacaaggccattcgacgctcagcgctaacacgcagaggatgtgaattaggattttaccaagcagagatgatttaaaacac

1366 gagtgcttttgacagatgaaaacgttttacataaactttggcatttacctgaagacaaacgaaaacagtcattgtgaatcgctacaactgttccacggacttttt

1 M P P Q S M M D H N T N L L P E P D E C

1471 ttagtagataaactaaaatagtttctttttttaccaactttttgacaATGCCTCCTCAATCCATGATGGACCATAATACGAATTTATTACCGGAGCCGGATGAAT

21 G Q T K I K C V L V G D G A V G K T S L V V S Y T T N G Y P T E Y T P

1576 GTGGTCAGACAAAAATCAAATGTGTTTTAGTTGGGGACGGAGCGGTGGGTAAGACCAGTCTAGTGGTCAGCTACACGACCAACGGCTATCCCACCGAATACACCC

56 T A F D N Y A V

1681 CTACAGCATTCGACAACTATGCAGgtaagaattttacaggtgtgagaaaacatttatctgtaactttgatatgtgttttaagcatatacagtatacaagttttgt

1786 gtaaaaaaatacaggtacttagcaaacagctggatgatttgtttatatatgtaattgtacaatatatatgacaacttaaaaaacgtatattctgcatttatgtaa

1891 tttttaggttctaaaaaccagagtgtttttaatttgtttaaaagtttaactatgcccatgagttgtttaatatattagtcaactatttaaaacagtcattattca

1996 actgctcttaggcgtgaatggaacattatgataattaactttttggaaatgttaattatgtttaaaacgtggaaatgtttataaaagcttgtctgggtcacattt

2101 gtttaaaagtggttccagattaagcaatatataagtgtaaccatgctaggaggttcggatagtttgaggggagccctgtgtgtattgggtgtcactccagctaac

2206 attagctgccatagagctagctcacatagttacagctgctggctggagattctattaataaaacatcttcataaataaataaagactgaagggtcttgtatccag

2311 ggcaaaatctgggctgattcgattcatcagcgttatcccaaaggggtcagaatgtatgtagaaaggaaacaaccttttattaaactttatatataaatattcatg

2416 ctcttatcagataagtaattgaagtatctagttttaatttcctgtatagaagataaagattttttaactaaactgtaaacaaaattgtatacttaatgtttaatt

2521 gatttaatgtaaaataaaaattttgattatcacattttacagtgtaatgtttcctccctgctaaattaaatgagaacatgtgttatcagaatcttggcaaggtcc

2626 tattctatacagttctttgtgtagatatgaaaattttatattcagctttgtcagtctgaaacctgttgattccgaacaaacaaatgttcctgttttctcttggac

2731 aaattttcagaacaaacatttttcaatattcagtttagacaaggttttttgagtaaaacaaaaaatccaaaggttttacttgtgatttgactgtgaacttacgaa

2836 attgaaaaattgtgaatttctctttgtagaactttgatgttttttagagtggatattttatattatcaaaattaaattacttaagggaaataaaatggagggtgt

2941 ggatatatatgcgtgtacttctggttaattttctttataaaacaagtgtttctgatatcttggggaattgatagctcactaaatacacttataacaaagcagtct

3046 tatgtgtactatagatattagtccacgatatcatgtctccatctccgacatatactccccattgtttctccgatcaatctccttcagtgtccattagttggggga

3151 ggggcaccaattagccagttatcagaaacacatgctgccctaatacactaacaggaaatatacctgtccttttacctgtgtagctcgtcagactgataacttggc

3256 caagttagaacatttttattaaccctgtcaggtaaaagccatgacctgttaagttgacctaaatagtcactttctagtcggctaatatatcacctgtatcatccg

3361 atccatttaataaaaattaaaatttgggtcaaattatattttgataccagacgttttttaatcatattttgaagtgttgtcatgaaataaaaattacttatttat

3466 atatatgtgtattttggtttgtaaacagttatgaactgtgactgaagtttattttgccccaaatgtgtaaacgataaaattttccactacctcattcaaactttt

3571 tattcattcagaaaacggctgaatatatcttgaatctaggtcatttttttatatacgtagacttttgtatgggtaagtaaacaagccctgtggttttatagatcg

3676 taaccgctttaaatatgaatgatttcacctccacagaagacccgaccaagtctgtatttacactgttcatgaaatcattgataccaagcagccatttaaaaatct

3781 gcccatgtagtctacaaacacttcaaatgaactcccctcgaaaaacgaacattgaacaccgatcaaacttgtgatgggagatttataggagcatatctccttaac

3886 acaaatttatgatgattttattggtgcgagtttatttgttaaagacaaatttggcgaagtgtcgatgagtagccaaacacaagcaagatttttctttaaggcaga

3991 ttttaggcgtcaggcttataaattcttcggacagtcataaatatcttgagacactggacaaactgaccactgtttcgacgtatttatcacaatggaggggacttg

4096 acatcacatttattaatacctatcaggtagattataaacgcaggactaataaagtcatctacctgtacaaatatagcgtaaacaaactgataatagaggatattg

4201 atttctttgttacctcaggtagttcgtaaacaatacctgttgtttcagatatgttcatgtgcgttaactagtgacatcacgatttcatgaattaagtattaggag

4306 tcatattacgtaacttaggtcgtaaaatagaatggtttaatctcttagctcctgttgtaatgaagggaatataaaactagaaatgaagggttacctttagcagag

4411 gaacataaaatattgggtatattatcgttaccttgatctatttttgacagcattttagattaattgggaagaagtgaaatatagaagctccctcagaattgtctt

4516 tcgacaagcctgtcaagtgcttttccgggagtaggggagcttgattttaatgctgtactggtaaaaattactattatatgtaaagaaatacagttctttgttcat

4621 ttcaatatcttgttccgattcagaaaacaaaattattcttacaactttctgtagcacatctgttcgtaccatttatgattcatttgaattttttctctaattttg

64 V V T V D G S P V R L Q L C D T A G Q

4726 acagTTGTTGTCACAGTGGATGGTTCACCTGTACGCCTGCAGCTTTGTGATACGGCCGGCCAGgtgagttgattagcactttatacacatatagacacacaccat

4831 cagctgacattgtgtaatcggatgattcacacttgttcgggacaggtgccctgccctcgtatcaaaccgtgaccaaaattagcaaacaacattggtaatcacgcc

4936 tcataaatggtctttgtctctcgaagtttataacccttaatgtatatatgggccgaaaaactactaatacattcatgtcacaagtttaagctactctgagcctat

5041 tcatggattttgtcggtcagttatattgtccatctgctgctattgtataatcatggattatggttggacacccattcataacgggtagggtaacagattgcgagt

5146 tttggggacatttctaaaagattaataataaaaaattgggaatatttatcttaattgtctagagacatgatgttatgtaaattatgtaaaggaaacttttattgt

5251 gaactgaacaaacaaatttcctgtatgcattacatggtctctatagcataatgaattaatgagacggaaaattgtcatcgattttaagacaatataattaaacaa

5356 agctataaaaacagtcgttattgattatccacataaacggaaaaatttgaaaaattaaacgttgctaattgtctgcggttattgtggggagtacaggtgttgatc

5461 ggaagctgttcgagatccgtatgactcatttagctacggtgaccgagtgtttacctttcaaactacacaattccaccgtacacccctaattatgtctcggagctt

5566 gacaaatggttgttaccatggtatcaataaaagtcacaagaaaacctcagtgaatttgaaaattgacttgtctgtaaaattcttccacttgatgattcactgacg

5671 aggaaaaaaagtcccataaagattttttcaaccgttgcacattctggttcaccgagaagaaatcacttcagggaaatcttttgtgaagttcatataaactcattg

5776 tagtgttaatgtgatttgaagataaaaaagacattccgaacataacagataatttgtacattatgcttttgatatcaaattcagattgctctattaaactatagc

5881 gtttgataatagagtacatttaattaaattaacatacgaaatgtcactaggtctgaataataggtatatatatatatatatctcaagtctggaatgttctacata

5986 aaaacatgtaagccgagagtcgtaaagaaatttgtttggcgcatactagtatgtcgtgttcatgaatgaagtctcaacaagttacgatttgacataccaacacat

6091 cgtacaaacttgttgttgtcatttgtccacaaacaaggacgtttactggttcggaatcaaagtggctaactaaatatttctgtgtctagttaggtatggagagcc

6196 ctttaattaagcatcattacgggtctctttccttgttggacgtttcatgttaatgtcaaatcgtccgatttaatgatggcgtgtttgtaaccaatggtcttttat

6301 ctgaaaattttcaaacaaaacacacacaaaaattactgataactatcttattgtttactttgaaaagtcaacttgacataaaatatgggtcaggaaggaagagac

6406 aatggggagagagaaagaattggaaagggaaggagagagaaaaataaaatccttccaaactgtgttttcccatacacttttataaattcaaacttgacacagatc

6511 ttggactttaaatacaaaggagaaatttgtcctttaaaataggtacaattttaaacatcatgcaatgaaatagttgacaattcagtaagcaaggtgtatttatat

6616 atacaagtgtatagaaacgagctagggaaaaaaagctttttaacagactgtttggctgatttgggcgcgagccaatagtttcattagccgtgtacaatgggttac

6721 tgagataatatatggctttgtaatattggcaaactttctcggtgaccatatatacacacgtttataatctacccttagaccaaatccagcctcagacacgcctag

6826 cttagtcggaaatttttatacaatctcgttacaggtatcttagctcgttacctatgattcaggtacgctcaacaggtgtataattggtaaactttctctcataca

6931 caactaatagggtaaaaagtggccttaccgatacattagagttaatttgaatgaatatcgggtatatttaggtaagtttgtaatttaatatttgaagtgtttgag

7036 agtataacacagttcttgttcaataaatacaatgaccttcatatccggttaaactgattcaaaagatagttttcatctatgtcagaattttgaaaagtcggttat

7141 tcggaaagtttgccaaagaaattccttcatatttaggtcaagttcttgttacatattgaaaaatagtaacagtctcgaaatatttaaagattatcagacctggaa

7246 tctgcttaaatggaaaaaaaacttgtctgttgattttgtattcctatgtttaattacgttaaaattacggtttcgtattttcatactcattttccccaagggtaa

7351 taattcacaatttcaagattgactggtttttctgaaatagacattcagatacacatttacgatacatctcatttggccgtaaaggaatgtccttttaaatacatt

7456 ctacattcacattaatagataataaatatacataggtaataggcggatggagttttattttgatagatacaatattagtctgtttgtggtatccggtacgtacca

7561 ggatatcgaaatcgatatcaacggacaatgtccagttgatgtccatgtgattcaaggcgtgataaacatgctgaccacttgaagctgtttacatatcttgttaaa

7666 atttctattttaacttttgaccagtactttaccaggtactaacattcccattgcgtgtccagtaccttagattgacctttaaatatacttgacaatatcggtcac

7771 ttcggtcaccgattatgtggttgtagccctcggcttttgtgttcgctatttaaccaattatagacacttgacaagtgacaaatacccgcatgtaaattgacagtg

7876 aaaacatatttttcgggcgtaaacttttgaactttttcataagtgcttaagtttacatttaaaaaagtgttaataaatacttatggttacgttctacaaaataca

7981 tgtttacgttctacaaaacgctttctcaggcaattagtgattttgatttatgaatttattttgtacatcaaattaataaacagacatttctttgcaaaagtacac

8086 aagtactgttaatttttgtcattgaatgacctgagagagaatccatttcatgacagctctcctgtatatttgtgcctcaaaatagaactgattagttatcgaaag

8191 atattcatgaagaagtgacacatcttgaaatagattggcttggacataccacttagtaacagctataggtgatggccggagtagtggacagcttgaccggtgact

8296 aattaggtcctgtgtccgagtgtttacctggtgagacaggtaattcttgtcatgggtcacttatagaccagttgtaagtgagtcgctggactgtgtcgcttgacc

8401 ttagttaccttagataagcagtggtcatctctcacctatgctgacaaaccttaagtgacagtgccgtaaggtcaagtcttgtccagtcagaatgtgttggtcagt

8506 tgtagatggaggtattgtaagaaatgtaattttgattcctaacctttatgaactccatataagcaaggacttagattgactaattctgtgatataagtagttatc

8611 ttggatggaaatgatgcaaaaagaaaaaaatgtgaacagtttcactatagaactctatctgaaaatcgtgtagctattcagaacagatttcggtacttgtgtact

8716 tattatcttgttgtttattgacaaaatgaggttaagtatcgtatttgtatgaaagatcattgattatctgacggaaagaatacgacatgcttcagtgtaagtgga

8821 aatacagctcctaagaatcattgaataaatgtcatttttatatccgagctctctcgggacggctaattaccttgacaggatcccatagtctatgcagaaattgat

8926 tttgtaataaaaatactatttgctgattcaggtcttttattgagaattgcatattttcttcctgttctggagttttctgtttcgatgtaaaaacctgactcttaa

9031 atttatcttgacggacgatccgtcaccttgtgtcagtcctgttcatttgtatattgataccacagatgcctgcttagtttcatctctgactcagcttttctttat

9136 catgaatattcttgtagcttaaaaatactcccaagctaactgtctgggaaacccgaagaaccattagtctctgctaaatgcatgcaaaatgcaaattggatcagt

9241 ctggaatagaaatgcagaaatcctgttagttgtattgtaatgttaaggacctgctgttcgcctgcagtttgaattgtaccaaacgtcctttgagcgctgtacgac

9346 ccgccataatgtcgtagagtatgttccgtctctctaacctgccatatgcttaaatttaaaataaggagttttcgttgagtcagtttcttctgctatgttgcctcg

9451 ctccatgtctgggaatttcatttgatacactagactgtctattttttctgttattattttgaggaaattggttccaagtcatagatttcaacagtttcagcagtc

9556 tgacagcacagagattgctaaggaggtttggaaattaaatcttcaagatttttaacaacttgtcaggttcattctggccccttggaaaattctatcaacaggaaa

9661 atttagcattacataatttgtatagaaagaatatattttgttatgtatttaaagtaattgactgtccttcagctgctctggcaggagtatttgtgaccctatgaa

9766 agccgaagaacagttcaactattcggccacccgagttcaccagttcacagggccctttacctgattacaagccctattgatgttaaaggcttggtcaaggagaga

9871 gttgacctggtcactcttgtccacttttatttttatcctcgatatccgaggtatattaaaacaactcaagtgtcataagtatgaaattcgcccgcagtggtacca

9976 agctagaagtggtaccgcaggacaatcccatacaatgttgctttttgttttcttagacgcttataggagattaaactctgtatacacctttgtaagtcctggtca

10081 agcctgggcggtcagccactcaaaacaaaccggctatagaaagcataatttcatgttacagactcaaaacttttcggatatttcattttggaaataaagcggaac

10186 tattcatggagattatagttttaaggttgttgtcacagttacataattttgttcaaaaggttcggagattagtttttatgtggggtcttgtgatatgatttaggt

10291 tttcctcgggcacagctttcggcagccattatttctacaaggtacatcctttgctttgccaattggtgttctgaaattgataaattgtttaagattaggtaaata

10396 tttgtatcatggattgaaagggctcagctcttaactaactattctgcagacttaattcttaccaaataattgaattataaaagtaaagaatttttaatgataatc

10501 gtatggcaagaatttagtctgtagcccaatttcctcaaaaaatccatatattcttcagttttctggatttgaaaaaatcattaatatcactgttggcgatcttgc

10606 tagtgtatctttaaccaccgattcaggcatttcttgaaattatttctctctggatggtaaattttaacttttatgatgtgttcacgaatcttcaggttattctaa

10711 ctttgacccagtttaccacatcttgttttaagtttagaaatgtttacgataatgatctagataccattctgactgtaccgtgaactgttgatgtctatactagga

10816 tgttgcgatgcgatgctgatatcggcacaatcttgtaatgtcttatgattcagaggctgtagcaagcatctttaaagatgaaacagcattttatttttagtagct

10921 ttgggttttacatgtcttcgactggaactccatgatgatgtatagatcaggttgctgtttttctccaagtgtgattaaactgtacagtactctgttagcttgaaa

11026 gttttcgaacaaatttcaatgaaattttgatgtcgggttaaattgtgttgaaatattcatgaagtgggattatgataatgttgttttttttctgtaaaaacataa

11131 tttatagattacttttttttttataaattttaacacaattgtaatttatttgttattgaaaacaacaaattgcaaacaaattgtctgttctggtttaccatgcaa

11236 tttcctcattctgtctttcacaccgttctcatgagtacgctcgctatttctctgctctgcctaagttgtgctggtgaccatccatcacatctgtgattgactcat

11341 tccatgatgagatctagtgatgtccgccattaaaaactgatacccctagggcccccgagtccatccatgtataaatctaagggtcaattaaggtcatgcagatct

11446 gtactgagggctttaggctaccgttcctgtcaattagctgacatcaggcacaaggcaataaacacctttctgaagcacacctgtccaagtcgctaattaccttgt

11551 agctgtcaataaatgtacatttcattatttttttcccaaaaagttattacaaagggtttttttcctattgaggaattatcattattacagtaatcaaatttttga

11656 ttttgttcatttgcttttgtcttcaaatatgaaataacttgatcacaaggaattagacaattgttgaacattttcctttttctctcgaactcaattgttgaaccg

11761 tttgctttttcttccgaaatttaacgagaacattttttaccttcccacacaataaaactcatttgcgcttgtcaatgaagcattacatatgctattttcggctaa

11866 aaactaatgacaatgtgtaaatgaggaatttccaaaataggctgtaagatttattgtctagccacacattcctccatacaaagtatgtcacttgtctttatgaca

11971 tttcaattggtgtttgaaatctttacgaccacaagaatccattatgcgatctggccattgcttcaatacttattaatcaatacatctgcagtgcctgaatgcctc

12076 gttggagctacagcgagtacttcctctgtatttatatggtttatatggaaatggtggggatttcgccacaattaagtccattgtcgtgttgtgactcatcgcgcc

12181 ccaaaacttatggcaatcaccaagctatagatgttaattactggaataccgcacacgcaatttttatctataatcacatagatatatatatatacatggaaagta

12286 ggtaataaaatgttttagatcttttcataaacaaattaccaaaaatgtataaatatataaacctcttttgtatacagatattgtgtctgaggaatttcatttagg

12391 ttttataaaagacataatgtatattaaaaaaacatttgtgtacaaaggagagagagagaaaaatgggtagacattgtacacggtttggggggggggaaacatagc

12496 attgaaacagtatttaaacagatttaataggtttggtatttcagtcgaatggtcatatagcatgtatccatctattcaagcgctgaaagaccaggaatagatccg

12601 ctgttggcggtacgtgtattgtggtggggtactggaccacacaatcgagcacccaacaccactattgtgaacaggtcaatttcccagggggaatttaaacataaa

12706 caaatcatatgaaatgttacacggtgcagctcttggttgtgttaagtggaatgggtaccctttaattgtgtgacctttctaccctccccgccttcctctttttat

12811 tataatttgcactgtgtgaaagaaaagacacttatgaacgagcacttgtatttaactcacgagaaaagaacttgatacttttcttcctcagtaaaaatgcacatt

12916 tttcgcttattacattattttttgaagtggagggttgggagagaatttgtaggaatctttactgtttcaaagaatgagggaatgaaaactttggtgtcttaaaag

13021 tacacattctttcatttgcattaattagcttataaaattggtcagataaatagtttaaatgttaaattagtccataatcaactgaggaaacgacaaattaaaaaa

13126 gtcacctgcttcaggccttatcaccagaggtcaaacattttggctcagccctctctttctctggtcctcttacagtgataaggcatcttgtaatttgtaagaatt

13231 cataatgataagctcaataattatgtaatgtatagcctgcaaagaaccactttctatgtgataaaggctttttatgttgaaatttatcaacctgtcgtttttcct

13336 ctaccctgcacccaacccccccttcaccccctgaggggacacggagtcagggaactctgtgtagtcaacaatatcgtggtatatgtctagaacttgtaatcattt

13441 tctgtctgcctagagacaagagaatgtctgttattattgcagcatagttctttttgttgttttctctctcctgtgatgccgacatttacattttgtacggcaagt

13546 ttggctcgcgacagctgatcttggctgtagatgtcgagttagtcatgcagagaatttcccctgtgcacaagtaacaaactggacccattttgttctgagcatcat

13651 cttaaactcaatataaactaaaaacttcaaatttgactctttttcattcttttcttttctgttcttttaattatggttgatatttttgtcatgatttgtgaattt

13756 tttgtagtttttgtggttcatgtatagaaccccagagggaattctttgggatgttaaagtaacaaggctttgaggattttgtcctgtggtttgggaggcagaatg

13861 gcacaaaatctgcagataagggaagctctcagcagttaatgcacacagtagcgtttttgtactccagtaattaacattcgaagaagaccgtgatttatatataaa

13966 tgagcagagtcacataaataggatttaacataactacgcacccacacacttaatcaagaatatttacaagtaaattatgtaaaattggatttgttggatttaatg

14071 atttcggagttcaataagtataaggttacggacctcccctctgagtcatctctccggggttttgttttttctagtgtatgtatgtatataaatatactacccatc

14176 tgttcagcgatgttgtatcttgataaatcttctgccggagtagttagctggcacagatattacttgtagtgtatcttgtttatggtgaagtgcagataggagttc

14281 acactccagctctatgggactaaatacaagcagatctggcaagattcgaatatcagaacatgaataataatatgctattacatctatctttagtgattaaccctg

14386 gtcattgctacagcagctggcgcgtaccttgccagcagagatcccctttcatctgataatcggtacaaacttaaaaaacacgttatataaaaacacagatataag

14491 tcagcacaaaatactgccacaaatcgcaacggagaaaatcctgctaacaccgcagggcaactccgaaggatggccaaaattaaagtgattataagttggcacagt

14596 ttttaaggtttgttcagcgagacttatcaaaataggtcggattattacacacttagtgtcagataatccagttgaaaattaccgtgtgacatatcttttgcgtta

14701 tcagaaccagatgatagctacatagaagctgtcaaaagttctgaaatgcctcggcactttgtttttcttaacattgtataatagtcaattaaaaagtattttaga

14806 ctcggctgccaactttgtgcgagctgcatatttcaggcaatgtatcaactttaaataaccttgaactttttcaataattctcaactgacattgggcaatgaaaaa

14911 cttatcggccaatttaaaacggccttgacatttcaagttaagatagaaagggaacacctgtctttgtggatataatgctgaaacatgtaaaccattaaatagtct

15016 tcaattnnnnnnnnnnnnnnnnnnnnnnnnnnnnnnnnnnnnnnnnnnnnnnnnnnnnnnnnnnnnnnnnnnnnnnnnnnnnnnnnnnnnnnnaaatagtcttca

15121 attcttctacagatgttgtctggtcatctatacctgatatttgtagatatatatatatatcaaacacgactacatatcttttgtgtacatgcaaattttgtttta

15226 cgaactttggcaaatgtagacatgatgagtttattaatatgtgttaatctttgattgaacttcatgttttaatgtcaaaattaagagtagaaacaagattgactg

83 D D F D S L R P L C Y P N T

15331 tttaagtataagttgtgaaatgtcttaggtacctgattttaaactaaatattttatttattgcagGATGACTTTGATTCTTTGCGGCCGTTGTGCTACCCAAACA

97 D V F L L C F S V V T P T S F H N I S E K W V P E V R K H C P K A P I

15436 CCGATGTCTTCCTCCTGTGCTTCAGCGTTGTGACACCGACATCATTTCACAATATTTCTGAAAAATGGGTGCCAGAAGTTAGAAAACATTGCCCCAAGGCTCCGA

132 I L V G T Q C D L R N D V K V L I E L A H Y K E Q P I P E S E A Q A L

15541 TAATTCTGGTGGGAACTCAATGTGATCTTCGTAATGATGTCAAAGTTTTGATCGAGCTTGCCCATTACAAGGAACAACCCATCCCGGAATCGGAAGCGCAGGCAC

167 A D R I G A S Y I E C S S L T Q R N L K E V F D T A L L S A L K L S G

15646 TTGCTGACCGAATCGGTGCAAGCTATATCGAGTGTTCGTCCCTCACACAGAGAAACCTTAAGGAAGTTTTCGACACAGCATTACTTTCGGCCCTCAAACTTTCAG

202 P L K R T K S S R R S K K G K K Q H E Q S P Q P T M S G K P C A K K T

15751 GGCCACTCAAAAGGACAAAGAGTTCACGAAGATCAAAAAAGGGCAAAAAGCAACATGAACAATCACCACAACCAACGATGTCAGGCAAGCCTTGTGCTAAGAAAA

237 G W K R F C C F L *

15856 CGGGATGGAAACGATTTTGTTGTTTCTTGTGAagttattcttatgcctaaaaaacacaaaaaatcaactttcattcgttaagaataaattcgcacagacttgcaa

15961 caacaaatgcctagacgttcaatctgcaatacagcactgcaatatagaactcggccaatcgcatcatgtgacacatgacggatgggctagacacccaccacaggt

16066 gatatgtgtgtattggtcatttagctgttatgtatagacacacaccgacagagattgtgatgagatagtgacgagttctcagtctgtcagctcaggctgaagtgt

16171 acatgattgcaccgacgaactgtcaaccatggttgagtcatagaactagagacggatgacgtcgcttttaagacaggttgattgtggattgtgtattacaaatta

16276 atttatcaatgagagagagtgcacatgttcgtataaatgcatgttttaatgagaagtgtctgagtcattggatgtaaacgactgcgacagcgatcgagtgcgagc

16381 gggagagtggtctgggtgatataaaacattagctatcgtttgtcacagatgtagttgtctgttgcactttgaggtgcgcttacatattggctcaagtgatatttt

16486 gagctgtgtgtttaacagatcaaattatgccatttctgtgagttatgcatccatcttaattgtttccatgtagatttttagataattttctgtttcataatagaa

16591 acaattatgcagcgcttttcaattgagaaccgagaaaattcattacagagatttattgccgtattacctttcttgttttagaaaatcgtaatggagtggaagaaa

16696 atcttttcaaattctgtcaaaaaggaaaatcatgtaattgtgattgttcatagctattgaatctgaagggtcatctggcatgggaaatatgtgtgaaaatgtgca

16801 gcatgcaatgcttttatagcaatagtcattataggcccacaatttcgggagtccccatgtctagcgtagagtaccattttacaagtaagaaatgtgaaggggctt

16906 tgtttctccctacctgaaacccgcctatcagatcaaagtttctggtataaattgtggccgtattttttttatccgacttttatcagattttagcattcttttaac

17011 ctgtgggactacttatgctatctggtgtagtgatacacatctttacattgtcacctacccttattcccacacacgagagccttatcacgcctacagacctctcca

17116 ccattgtcacagggccgtatttcctgatctgtcagggtggctttgtattagctggtatgttggtaccatgcccaatcttaggtccagttgatgaactgttcccga

17221 tgacaagtctgacaggtcgtaaattaattaatagaggtgttgaaaacccaaatgtggcatgctgccttaacagaaaaaaattgtgacacaattatttcgcaacaa

17326 agagaagatcaaatcgtgacgatgatctttagtgttatagtttttcctttattttaacaattacaaggcccggttcagaattactgacatagtcctcaaacattt

17431 ttaccagatcattcctcatgccaagtaatgtcctcagatcagattgcaataaatcagtatttcgtcaagtttctagctcatcaaaaatttgcataatacctgcca

17536 aacttactgtacattgctgtattttcgcaaatctttcaataactttgagaattttgattgacagttggaatggaaaaaaaagtttaaataagttataatatttgt

17641 gattatttgaatgaaatgaatgatttaacttcaggtatttcagtttttttcttcatatcttgtctttttccacactagattattaccatttccttcccttacctg

17746 tatctagataatgtctcccaagagtttttgtttcgacctcatccctttcatagaatgtgagggtgaaaagagaaactacaagcattctcctgcttcccttcaatc

17851 caacctgaccccctgaccccctgacccctgacccccgacctctgacccctgaactcctctttttacctagtcatctccctttgtcctacgtagccacacatgtga

17956 ttccaatctacgacttctttcctctctctggaatttgacaagtatatgtttatttaaccatttttaataattattgtaattaaactggtgctgacaacatcattg

18061 tcttttaaatcatactaattagttttttaatcatgctaattaattttcaagtatttcttttttaaatcaaatcttttatttgaaacacaatcttgacaaaggttc

18166 tcattagaacttagaaacctacatgtactgtcacaaattcaatgttttaactgttatataagggaacaattttaattcagcaaatgttatacttacagttcagca

18271 taatgtaatgtttagtttgtctgtctgtcgaagctgcttatctcaaacctgtaatttttcgaaggataatttccttaaaatgtttaatttatggaattagaactt

18376 cttgtaaataagttttattgatttttatcataaaatgcccttgcttataaattttcactttcaaaaaccaatcaaagagatcaggtgactagtgtttcattatcg

18481 atgaaaactgaagtgtctgcaatatcaaggttccgctttgtacaaaagctaaactattcagacttttcagattccacttttatctatatagctgtcatgaaaaaa

18586 cacttcaaggctggtacctgacaggcacacctgcccaggtaggtatatacctgtacaggtaagcacaggtagatttgatgataagcggccctgacagacttgacc

18691 tcaacagtacaattctgtgtttgttatatggaaatgagatgctcacgtgtttcttgaagcatcaactgttatatgctctcatgtctttcattcatgttttgtgtg

18796 tagattaattgtcctgcaggtatatatgtggaactagtcccatcgaattgcttgcatttgttaaggtcaaggccaaattgtttgcttgaaaggtcaaggccacat

18901 tttttgcttgagctgagcaataagaaaaatatcatctttttttttttaaacaatattcctgaatgtagttaagtgtaaattactttatattgaaaataatgttac

19006 ttaatacacagcttttgttgataaaaaatagtctgtgattcttcaaattcaaaaagaagctagatttgtaattgacattgttgtgagaaaaaaaaaaaacttttg

19111 aaattacatatgttcagaattgagaaatattatttcgtgtaactgactaaattatatcaattttgttattggtagccctgagcattacgacaaatggccaccatt

19216 attaaaatcaagtgaggctatatatcaaattcactagacctcccctatctctcaagccaaaaatgattttgtagatatgttatttcgtaatgaaaagcttgtggg

19321 taattcacaagcagttctacacattttaaacttacaaaatgagattttttatcttgaataaattaattgatgatatgtatatttatatattttgtacaagaaaaa

19426 gtgccttttaaataagtagaagttttgacgctggaagtgtgggcgttttcgttgagacaggcctcataacttacataaaagtccagctaagaggtactacggcat

19531 ggtctgtgaaggaatttgggttaggtcgaaacggaaatatttcccagcaaagtttgtaaacaactttaaattatctgaatttgtatgaaaatggaaacaatataa

19636 ggaccaaattgacaataaggtgtcagagtgtcaaaatggtattgtcctataattgtctcgcgacaaatccaaaccattttaaatcggtaacaagacaatatggtt

19741 tagatattaacgcatttcattgggtcatgttcctattgagaagtagaaggagtcggtatccattacaaaaatgtgtgtcgtccttttgagcagagctacgtaagt

19846 aataagataactattgttggctggtgtgtttatgggctagtcaagaatgcaacagaaaatggaggcgttttaatgttgatcagattaagtcaagaaagcatgctc

19951 gcaatgcttctgttctctgccaaattttacccacaaatgtacatgatgttacgtccggtgtcgtctgctgtgaagtgttatgcgattaatggcttagcggtgccg

20056 gaattaaaagttttatccaaattccttatcacagaccatggtgttttaaggcctctggacatttttttacgtctctctgacgtcattgtgttaagactactgtca

20161 ggtaaaaaaacagcgaacgttcaccgcctattgcgtcatatgtacgtcagatggagacacgccgatcgtctcgaatttttatatgaatgatttattgcctgttga

20266 ataaagatttctgaaagaaacaattccgaacttgtatgttatttttatgttgtgttgtacattttgttacaccagaacaggaatcgatggtacgtcaagtttagt

20371 ttatttgagtttgacgtcctattaacagctatggtcatttaaagacgtgtcgggtctttgtggtggaggaaagccggagatccaggagaaaaaccactgactgac

20476 ggccagtaccttgcaactgccccacgtaggtcttgaaccagcgacctagcggtgaagggctagatgtagcagaatgttaagacggcttagccacttgcttggaaa

20581 ctgtaaatcccgggattttacgacaatatcagcgataaataccatacaaaacatccagatttttcgccaggttattaaaccaaatgttcatgaaaatgattccaa

20686 agtaaaacagaccaaagcggaatacatcagctataccgacatttctgtaatggcgggagaacgccgaccgctacattgataaaacgccacccattgagttgtgtt

20791 gtttgtatttaactggatcgctattcacagttaggaaggcgagagaaactattacgttcttagtgcctttaccgacacgctctgtctaatgtgatggagaatcca

20896 gaatctcgccaactttcaactaggaatcttcgacgtgtgatgtgttagaaattctgctgttatcagagcaatctaatctctaaaactagtgacttgtacggctct

21001 gaacacagacactgacggaatggtttatttttagatttttttttattaagatgtactttgaaataatttcctaatacttcgccatgtttttaaagactttttgga

21106 atgtaaaaattgtccgacattggtttaacatatactaaatctatatatgaaatgtttgtttgttgtaaaatattatattatgatataataatgacgtaataataa

21211 aatgtgccgctcagtgatagcatggtgcagaagtcgggcttaatgtaatattaattttgggaatcgtctatggcacattctagcaaataaaaagacgtaaccgca

21316 tcaagaagtgctggaggaatgtgaaatcccgcattcccacgggatggggaccgtcaatgttactggcactggggttattatggaaaccatatgacgcatgaaaat

21421 gagaccaaaagattcctctgaactctcttcattatgttggcaggcgagtaggacaaagcagatggccgttgccagaatatttataacgtacatataatatccaca

21526 ttagtcttgtaaatagatcggagtttatatatcattatatatacgttagatccaaactcgacccagattggagctattttggtcagtatatatatgtactgttaa

21631 acgtatggtgcttagttaaagggacaccctcgctataattctatttgagagctactttataactttggtttaaaactaccacttgtttaaaataatcatttgtac

21736 acgttttatggttttaaaaagccctacccagattcgagatagatttattgatagtcaaagtgtttttaacgcttttcgtgtcatcgcccagaaacgggagtttga

21841 tcatattcccttcggtcagcaaaaataagaaggtaatattttatataagttattgatgaaattaaaagagaggaaaaaagcaactcttaaatcttcttatttaat

21946 gctttttattgtaaattgtaaaaaagttttaagtgatttgtcgacaagtcctgcgtgaatttccaattttcacagacttaatgtatataatttgtacatgtcacg

22051 aaacagactcgtgtaatactagtgaatatgtgtttaaatctgaaaaaaaatcttacaaaaattgcatttgtcgtaattacatcaaataaaatacgtgaccgttaa

22156 cagttttctgaaaaatatgtcgcataaatttaggcagtgcttattacattgtaaaatttccattacaagtagttatagcgctccaactggatcaaaactcaaaga

22261 aatccgattatgtaaaaaatgatcgaagtatttgtgtaagggagaatgcgtgcacgcagccgttggaacacaaaaccgacctgcatgcgtgtcacaaaggatgaa

22366 tcgactagagttcattatttaaggattcggctgaatattaaagcgttggtgcttgacatcagaaacgacatgtttccaaaacaattaaatgttacatatgtctga

22471 aaatcgttcttaattcctttgacatgatagtagggtcaagtgtggtcatttttttattgatacatatattcaatcctgtctttaccgcacatcttaggaacagta

22576 tttaggttcagataagccaagttttcgaaatatacatgttcaatttacataaacaaaaaaataacatcgagaaaacagaaaacataccgtgaaactgacaatact

22681 tgacaacaatagttagtgttggacacggcagtttatactctctacctgaccgcattttggacaggtcagttgtcacagaaggtaatacacattccgatatataat

22786 ataaaactcctattggtcaagtgtgaaatcgacattccatgcaaaggaaaaaaagtatggttagtatgaatatcatcaaaatctgttcgtattttgaaaacccag

22891 tacaactgaaatcgtatttcatttatccataaaatgacattgaggacaagatgccttaacatcaaacaagcgtaaagaagaaaatgtttacattttgacgatttt

22996 tatgacagttctcacgcgctgtccgtatctatatgatatacggtcacgactcgagagataaaagtctggcgaatcaataaatgggccgtatggctggcatattta

23101 cagcaactttacagcaactttacagcaacaaaaacgaatgttaataccagacactgtggtggcgtgctcagaataagcttaattagcagacttgacatgtcaagc

23206 atgcagatatactcgtaattgaataaatataaatgtttttgtaacaaataaaagtgattgtttttctatggggaaacaatacacgatgtttttctatgtggaaac

23311 aatacgcgattcaaatgtctctttatagattagctatccaaaatacttcagttctcgacttatttttatcttaagtgcccatacatttcagaaaatgtctggttt

23416 cacgttttgtatttccctgtatctagacgtgaaataaaacggggctttgtttatctcatatcttaagatagtgtcgaaaggatctcactttctaaatttctaaat

23521 ccactttagcacaaacgatatttcaatttatcttgatttgtaaacatttccataagtttcctaaaagtcgtgtgaccgactttaactctgacagctgttgaacgt

23626 gttaaacaaaaatattgaaacacaaattggataagttgagtgcaaaacgaatatagggatttatcaacagagaaatttagtattgccttataggaagtttggtgc

23731 acattccggattaaaggattc

**CfRnd**

1 gattgaattagtaaaacagaaaaatatgcacaattttatttacaaaatgttcccttttcatccgttcatgaaaaataaaataaaaaatatagagatgattttctt

106 ccggttgaatttgtatgggtgattataagcgttttataagttataaataatatacgttgtaaaactcctgagagtaatataggacgctgtgtaaatgatgttatt

211 ataattattatttaaaaaatccaagtgaacattccagaattatcagtgaaaatcttcttcaaaagtcgaatgacctttttattgcaattgtgtccatttgataat

316 ggttctagtcgtggacttgtctgtagacagccgatagtaacatacggttattgtcacactgttgacagccgatagtaacatacggttattgtcacactgttcaca

421 gtttcattcagaattcccctctggcagccaacatgtgttctcacacttcaccgactccgatcgtatttatgatgaataacccattgtaaaaatagttatgttgta

526 attctactataaaatgtcggaagcctgccttacctgcggctactaatcaatggataggtaagatctacgggagtgccgtgacaggtaaagaccttttttactgtt

631 ccggtgtcaggttgacgtgtctgggcaacaggctatagcttaatatcaactaataaataaataaatgaatgacatcaaatgtctttatcttgttaatagaagtag

736 gtagagtttaaaaaaaataataaacaaataaactgatattagaaacaacttctactttttctttgtcaaagatttccacaaacattttttttacattcgttatat

841 acatctcctgatgaaataattagcaaaatttccttgcaacagtctggatcactcgaagacgacctctgagggaaaatagcgtattgaatatatatgctggaattt

946 gcaatcgtcttcatatctcagaatgaactatggggacaatgatcatgctatgtattatattgcttgccgataaggagacgccattaatatagtataagctatcac

1051 gttacgcccgcattcatggtcacaaagaaatagtatatgacacgtaacttgctattatttgtatgactttggcatacaatgaaggtcgtacgggtgaagataaaa

1156 ttttcaaacgacattgtaatattgatcgactttccaatgatactatggcgggtgaagcatcatacgtaggtcaaatgttgtcatagatttgtttatgtagcttca

1261 tcgagagaaccaaaaagccgatggtcatcacaacttgaaaacgctggtgacacgggtgtaatacgtacaaatgaatgaccttgatcggtattcataatcactggg

1366 gtcaaactgcgctcaacacgtagaaaaatgttgatcggtcgactgcagttttggtcaaatatacaatggtgattttttaacattcgacacttcgtgtagattagt

1471 ataatatcttcatcagatgaagtgattaaagggaaggagcatggctgctctagtgatttgatttacctatttgataactttgtcatcaataaaaacagagaagcg

1576 ctatacacgtacattatatatcttgtcaggaaataacgcgatcattcaacgattttatttctgtactttccacaaggaatgattctagttgaaaataatgacaac

1681 gataaaagtagtgatgccgaatacagctgtgagggtgacgccaacgacgagtctgggtgaaaattttaatattcgaagggaagtaactcgtacagtaatttgcct

1786 gtcgtccgaaaatctcgcaatacgacttgtgcaagtcgcagggacctgacaactagttacagctatatttgggcctaaactaaggtgctgagagtaattttacaa

1891 gttatggggctctagatcgattttcgataaaaatgccaccccgatggtcatactcctttttatgaccaccgggggtggcatttttatcgaaaatcgatctagagc

1996 cccataacttgtaaaattactctaagcaccttagtttaggcccaaatatagctgtaactagttgtcaggtccctgcgacttgcacaagtcgtattgcgagatttt

2101 cggacgacaggcaaattactgtacgagttacttcccttcgaatattaaaattttcacccagactcgtcgttggcgtcaccctcacagctgtattcggcatcacta

2206 cttttatcgttgtcattattttcaactagaatcattccttgtggaaagtacagaaataaaatcgttgaatgatcgcgttatttcctgacaagatatataatgtac

2311 gtgtatagcgcttctctgtttttattgatgacaaagttatcaaataggtaaatcaaatcactagagcagccatgctccttccctttaatcacttcatctgatgaa

2416 gatattatactaatctacacgaagtgtcgaatgttaaaaaatcaccattgtatatttgaccaaaactgcagtcgaccgatcaacatttttctacgtgttgagcgc

2521 agtttgaccccagtgattatgaataccgatcaaggtcattcatttgtacgtattacacccgtgtcaccagcgttttcaagttgtgatgaccatcggctttttggt

2626 tctctcgatgaagctacataaacaaatctatgacaacatttgacctacgtatgatgcttcacccgccatagtatcattggaaagtcgatcaatattacaatgtcg

2731 tttgaaaattttatcttcacccgtacgaccttcattgtatgccaaagtcatacaaataatagcaagttacgtgtcatatactatttctttgtgaccatgaatgcg

2836 ggcgtaacgtgatagcttatactatattaatggcgtctccttatcggcaagcaatataatacatagcatgatcattgtccccatagttcattctgagatatgaag

2941 acgattgcaaattccagcatatatattcaatacgctattttccctcagaagtcgtcttcgagtgatccagactgttgcaaggaaattttgctaattatttcatca

3046 ggagatgtatataacgaatgtaaaaaaaatgtttgtggaaatctttgacaaagaaaaagtagaagttgtttctaatatcagtttatttgtttattatttttttta

3151 aactctacctacttctattaacaagataaagacatttgatgtcattcatttatttatttattagttgatattaagctatagcctgttgcccagacacgtcaacct

3256 gacaccggaacagtaaaaaaggtctttacctgtcacggcactcccgtagatcttacctatccattgattagtagccgcaggtaaggcaggcttccgacattttat

3361 agtagaattacaacataactatttttacaatgggttattcatcataaatacgatcggagtcggtgaagtgtgagaacacatgttggctgccagaggggaattctg

3466 aatgaaactgtgaacagtgtgacaataaccgtatgttactatcggctgtctacagacaagtccacgactagaaccattatcaaatggacacaattgcaataaaaa

3571 ggtcattcgacttttgaagaagattttcactgataattctggaatgttcacttggattttttaaataataattataataacatcatttacacagcgtcctatata

3676 actggtaaaattactctcaggagttttacaacgtatattatttataacttataaaacgcttataatcacccatacaaattcaaccggaagaaaatcatctctata

3781 ttttttcttttatttttcatgaacggattaaaagggaacattttgtaaataaaattgtgcatatttttctgttttactaattcaatcacacgattttaataaaag

3886 cggtatgtaataaagtagattgatttctatccacgtgtgtatagctgcttaccgtattatgctctggggacactgacaaagcctaccccacaacacctaggtatt

3991 cgccctaacaaagtctttccaatatcaccaaggtattactaaggtattcgccccattcacaccttgtcaaacacttataatgcatagaaaattgcgtgtccgttt

4096 taaggtgtcatattgtaagaccggatgaggccctttttatattatgaagcgctaacattaaacatccttttaattgaactattataattaacctgtgaataatct

4201 caggtttcgttgaatgtcatcttaagtagatgattttccgttgagtaattgttaaagtgcgtgttggtagccaaagtcagaaaacccttccttgggcctttcaac

4306 aattcagctcaattgacccggtatgacctggaatctgattggtcgagccagaatgacggatcacatgactgtagtcattcacatttttctcactagctagcatta

4411 ctattttgagcgagcagtgtgctgtgttgatacggacggttgactggtcgttttgtgaatgtgacgggcgtgtgtgattataagactatctaattacacaggtgc

4516 agccctgactaccgagtaaaaaccactaattatctgtagctatgtaaagttgtggtcagtgcgtgaaatgtgggggaataagtttcctgacagcgccccgacacc

4621 acgtggaggatagtcgtgaacagctcgaaagtttataaacacactgctacttgaagaaagtggtgtgaattcgtagttttgacatctgtccctggaacgtagtat

1 M G Q N G G E T E A A A S T V T V V K S K L V

4726 ttgtgaatatatttctcattggaattaactgaccagctATGGGTCAAAACGGAGGAGAAACGGAGGCGGCAGCCTCTACCGTGACTGTGGTGAAATCTAAACTTG

24 V V G D C G C G K T S L I K R Y V Q G E H K D

4831 TGGTAGTAGGAGACTGCGGGTGTGGTAAAACGTCCCTCATCAAACGATACGTACAGGGGGAACACAAAGATgtaagtatacataaccactaatgccatgtaatat

4936 ataggcatatacatatatatatatataatagcttaacatgtacggttccgtaaggccgtttatatatatatatatatatactgatattcgcgatagaaaaagttc

5041 ctggaatatgtttcttttagaatttatggagagaatttgtaatctaagtcaaaaatgactaaaacaagtcggaaattttgataacgtcattttcatattataatt

5146 ccaaaacttatattgctcgtacgagagtcattttgggaggaggatagtatcccgggtgtggcctgtgagggggaggagggagaggttgtatcacacgtgccgtct

5251 tcagttataatagatacttttgatacttttccttttatgtatttacaaaatgtacaagataaacattaaacaataatccaagtaaagtagtcgattttatgtcgg

5356 tcaacatattacaatatatacaccagggatccaacaattacctacttattgtcggggattctcaatggttcctttatctcagaaaaagaatgtaagacaaagata

5461 ccattaatgtctaaagtacaacactcaagtatcttcagctgatttacgaggttcacgatacataaatcgaatacacaactgtgtcatgacgatatatattggact

5566 tttggagaatacaaattttaccacaagtctatcccatccaagtatacctcccccgaccccccaaacacaaccactaattactatattttacttctccaaccttcc

5671 cttcttcctgtctaatgaccggattcgttccgtcggcaaacaggggtcgaaaagtcgagaattcaattcagtagcacaaaaaaaatcaaccattacaatacaatt

5776 agtaattaagacgactttgatttactacaatggactattggtgtactgtggccgactatctttactgcaccagctaggcacaatcaacgagtcatcgagtccctg

5881 tgaactgtacgtatgccaccaagacaatagtacaatatacatagacctatctattgtagtataaacctcaatgatcgttcctattcatttatagatcatatatac

5986 cagtcctatcgggacaaaaaaaaataggacgaacggacagctacttaaggtcttctttatattgacaataaatgtcacagtcggtgtatatattcgaaggtttga

6091 tacataaaaaagacttgagaacacgtacaagttgtcgttgagtggaatatcaatgatagaaaatagactgtacgcttaaagcaagtcacagtgttaaaatttaca

6196 agcacttcttcaagtataatgacatcaatctttatttccaatctgccattccagtaccgtatttctaacacgacattttagtaatgttccaggtttatacatata

6301 ctatgtgaacgatgtctatatatgacatcgttaaaatagaataatatttatacagtgtgtacattaacctggtggatttcgttgaactgtttggtatagggatta

6406 atctagatttagttaagcagtatttgataaagcaaactacctatgtcctgttatatatacacaataatgtctctttttccgagtgaacgtcgaatgaatttcatt

6511 attttgtgaatgcaatgtagaatcgtagtccggagcctagaaattccggtaaacgacaagtatcggggatgttcgcttccggtttctgagatatgaatggaagaa

6616 aagatttttgtgtgatgtttaaagccattttgcggttgttatcgtcctacctataagtacctgtgtcggtatagacacatgtatgttacctgtacagtacaggta

6721 tgcgccctcacctggtgacctatatataatcgcctaggagtggcgaacttgcatatttgtcggacataaatacccaataaacgttatgtaacttacaaaagtgac

6826 attgtaagtctgtagtatattaacggaagtccataagacgtgtagacacatatatatcctattatattcctgtcaacagtgaattcgttatgtgttcttgcgtac

6931 ttttatatcccgctggtcagtgtcgtgtctgactctaaaccatagttgattttatcgtcagcccctagtttttatgtaacccttgggtggctttgttaacttccc

7036 tgaatattgagagactacaatttcaggtatgaactcgatgacatttttaatatgtaagacttctatcgagaagtttaatgactcaatctttactgtaatcgtgtt

7141 gtgcaacagcagtcagacggaaacgagccgtactggtaaggtacagtgtacgagagattgactacacaagtcgtgcgagggtataacgatataattagcaccaat

7246 gacaccctaaatttcactagttaataagattggaataaacattcatggaatgtatataatcacaaaacatgaacagatgaagggctttatgaaatccacaaatcc

7351 taactaaatagtgcgggttaaacgacgaaaaccattttttgtaaaaatgtaaaaaaggaacacatgtacaggtctgttttctttcgtaggagtccagacccatta

7456 acctctttcaggttgtgcaatttaccaaactttcgtattttcatctttaatgtttttcttctggcgctgatgctgctcattctccacctgttatgtcacagacgt

7561 aaccccgatagggggtcattatttcattattagacaaagactaacccctatcaggggttcaattattttatctacacaactacagtatatgcgtgccaagttaag

7666 ctaagcaactccccccttccccacacccgaatctcctcagtcggactgtccatgtgaatcaacacttttgacacttttcgttatcagtaaatgcagaattattgc

7771 gataatttaattatccaattagttgagctaaatatacatccacatactatagtattatcacgtgatgcgtcattagctatcgcggtctgcacgtgctcgttagtg

7876 acttcggatagcggtaattagtattactttgtgtaagacgttcttagaagtaacaacggtataactgagtctgtacaaggctcaatgtgggaacatggctttaaa

7981 atctcaaatgaacttaatgagacgtagattttactctaagtaattaaaatgttggaggggagtgggttaatcgttgcaatttatgaacattagaactcgtatcaa

8086 aagaaatgaaaacagtatgtcctacttttttcctgccttgttattccgaagttatgtctcctcgggtttactgggattatgagttggtttcttttaactttagtt

8191 aagctttcaataattaaagcttgtttatgaatttcgctgctaaattgaatattaaagaaggaatgttggaactaagtcacacttgaattgttaatttatatatgt

8296 taatgaatttgaagttcgtatgtgtgtggtgtggatacgttccccgccagctcagggacgacggggatgggttgggtagggtagtgtggaatggggataacagtg

8401 tacaggaatgtatgtaacagattagacccactaagagcatcactttaaagtgctattacgttacagaataaactgactcagaacatcttcacgatagtacattgt

8506 acttggacgtgtgtaattatcggaagctcttgaccacagtcacgtgggtttccccttccagctcctccactcccaaaaacgagtaggaaaggatagagaaagaga

8611 aaacaaatcacggaaaattataattattagggaataagtataatagtatacaatatataccgattatggtcatgataaatgataatttaaccatgataaatagtt

8716 ctagtgttagataatagtttccgtctgttgtcgggtcgacagtgatgtttgataaatgtaccgttcatatctctgccttttcgacatagaaaagttcaggttact

8821 tcattatataacagcgtagcgagcttataattacccccctttatgaccgctggttatataatgcgtctcgattccaggcgagatgaaagacgcgagtgtatacga

8926 aatacaacgctgtcaaaggtatattttggcatctgtatatgtatgtttgtttcattgtagcattagttgcacccattcccctgtaccgattgcgaggtcttgcat

9031 tcttggttctaaagtccgtgtatgctcaaagcacatatttgtcttggcctattctatatcaattttggtgcttctgaccaggataactaaccttattaaaatata

9136 actcgtgctttcataaaaacttaataaagaattgaattgaaaatttaaactttaattgaaattttgattaaattctttattaaggtgtaatcctagccgacgctg

9241 ctttggcagtattctgtaattcagacaatatgtactctgtaaataacgttataatctcctaagtatggcattcaaatatcacgacatcgttctgcgcgttttttg

9346 gaccatatttttatactttgcgggggagaggttttgtagtggcatgaattttaaatttgacattttatcagtgacagcctttaagaggaatgtggaccacagaat

9451 ttcaatttactgtccagacttcaatgtgtagtgttccagtatcttgaatggtttgggctgtatttgattttcagacttacgttttacaatgtgtacagagactgg

9556 gtttagaatttaaagggtaccgtataccaatatatgagtattaaatatttgcacgcaaatcttgtaaacgcgagtcgggatgtttgtggcattttagctttcttt

9661 acttttattgtctgaaaaaaaactacctatttagacggtaaggttttcatgttgttgcttattgttcagacagtctcttttccttacgttttgtcagtttaaagt

9766 tatatttatcttaagttgggggtagagatttggttaaaaaccctatttgtactagactttctgtataaaacacgttagaaacaattgcaaacttgatcttagata

9871 aagctatacagagtacatgcgagtcggagaagaacttgtttacgaaggcagtgcgcacgacgctgaaatattgatttcttcacatcttaaaagtctaccaaattc

9976 ttaagcaaataaaaactattgttttgtgtagataaacaactcatcaaatagtctttgtcttggaaggttttgcttgtgatgtatattgagagaaacatcaaacct

10081 gtttagttgtgagttgaacactagaccaagtctggcggcggctggtttctgattctcctgtcggatcgctatcttctatacaacagcggcaccgtttggagattt

10186 tgcaagtttttgtcgttaaaaaaatttgcagactttttgctaagctaaagaattctctaaatagttttgtcaataaaaatcctgagcatacaattaccaacgatt

10291 tgatgtgattttggtcacaatataaagcgattgaggtaattctctgcaggatatttgatggaaggaccgtccatgattaagaacttgtattccaaacctgcatta

10396 tactgatatcatactgcgtcttatagaacacaaagatgtacataagttcgaaaaagaagtagtagcaatgcgtgtgtaagaaatcttgtaccgtgattcctgaac

10501 gaagcaccgcatggatggtaaaacaaatctgagtattcttgaagattatgcgagacgacatgacccatattttcctctctatcgctgaaatcggatcacaaaacc

10606 aactgttaatgggttagattgtatcgttcacaggtcggagtccgatttcctcgacaaaagagattactgaaaagttccggacttttccccctccctcttgttaga

10711 tctggcatcaaatctgatagagagtgatgtggtattgtgtttagtgtaggggttttatcgtaacgtgtcggcttaatcaactggtgtgttgtattaatgacttct

10816 accgtgaactttatttcaccaaacccttatcgatagatattacgaccttacgaagctaactatgcatttacagtatatatctctctctccgaaattctatattta

10921 cacaaattgctttctggacgtggaagattaaaagttcgatagcaacctctgagcaaatctcacgagatctcgtcagagtggagaccgattgttgataaaatccgg

11026 aattctcaactatccgaatacgaatggtgtttttttcttgtcacagtgaccttctacacgtcacgaaagctgataaaacaaacacgtgtagtcagaacttttaat

11131 aaaaaaaaaaaaatccatttttctacatatgtcgatggtttataagcacatttctattatggtactgtatataggggttcagttacctgtatatgtgtcacctgt

11236 gtataaaggttacgtgtgaattcaatttgctgtacagtgattgcccctcctcctctcaccaattacacaagtgttcaattgctttttacggcttaatgtatacat

11341 atatgcaatagtacccagctaaacacatgcttacaagtctataaattacggaacttaatgttaaaggataccagtattacctgtacacacatgataaaggattgg

11446 ccgacgtaatatttagttatttacaatggagaacttaaacaaaaaatgtcgttttttatttggtcgaattatgaatgagtcctgtatgtaaccacaaggcatgtg

11551 atacgcctacacaattatacgcctgactgacgtgtcccgacatgccccgtacaggtggaaatctccaaccaactaaaaatagacatcacgcaacttacagtgaga

11656 atttcgtacgtctgtgtttgggtaatgatgcgatatagcctgactccgtttatgcatggtggctgacccattttatactgatgacacctcagggaaaacctgttt

11761 aaatgtttgccctgcttttaaatattattaaagttaccgtagactacttgggtaatgaaagtaatgcaccagttaacgctggggaacagggaatgaaaaaagtct

11866 cactataagtaataaggacagctcaaatgattttattcctataatatattatttgctgttcttaaatactttatacttctaattatcggatatgacaagtattat

11971 ttcctcaaccgtaagaagttataatatatgggatttattcgcgtagtaatgagatgattcaacaaggaaacaaacttgtgtcttcatttctttagtaatgggggt

12076 attcaacaaggaagcgacatttgtcctttattaccgatttatcattgtgttgaacaaggagatacacaatttgcgttgctctaacacaattggcgctgctcttac

12181 acatttggcgttgctctcacaattagcgttgccgtcacaattggcgttgctcttacagtacgagaaaacagggcttttttgttggttaattatgaggccagggac

12286 ttaaaccattgtcattagttcaaaactgggttaacgttggtattggtgatatgagataagaaagacggaattttcacttctggatatgaggtactgagacaaggc

12391 gccgaactgttacctccccttttctgtatacctataaaactaatcatgttcactgcaagttttctgacaaccaacagttgctgaaaattgcctgtttttttttag

12496 tccaattgaactgaaaactgcaggcttgcagaatgcataatgatgtatcatactcgaccactttatcgttaatgtgttttgttttacttgtgattcgatcctttt

12601 aatgtctggataaaaaaaaaacacctcacacattagattgattattatacagagtttcatagctctgtccattaacagacagtgtcgtgtgttttgatagagtta

12706 ttgtgagagtacagtaacgctatggctcggctgcaatattaattagttttggatcacatggactttttatttgatctcggtcatatacacgagggtgaaagaatg

12811 ttgactgttacgtcataggcaagaactcaatattgatctccagctatgaaagcattcggagtatctctagacattaactcaaaagtgatatggaaaccggaaaaa

12916 aacctcggccattccttcgagcatcgcttttatagctctgtaatcatatacataattatatatataactggaatttaagtgcaatatccgtttaactttttttat

13021 aaatatctaatgaatgtgaaagtatctgatttatttcttttctgtcaagattaaaaccataaaggcgagctagaagaaagttttactgcccttgtcgaggttaga

13126 ctgtgttcaacatcgcctgcactttacatttgaaagtaaagacgaaagtttatcagcagtaatgatgtttttatcagcagaaagcgaaacctgagagtatacagg

13231 tcatgtccacattatttactacacaatatcggaggattttatctgttaaccttgttgtaacaaggtcatatacaggtcatacatattaacatgtattatgtcccg

13336 gactaggactaggggaggggctttttctgttagctgagcaatcgaggtgtttaatgttttcttaaaagcttacgctgacagtaaatgtctgtaaaatatacctcc

13441 aaaatgtgaagagtacatgtttatttttttgtgaacacttgctacaaacagctacactaaacagtatatacaatgtatacagtatacactttacagtatggtctt

13546 tgttaagtctgttctggatttctcagcagacttttcaagatatctgagggttgtgtctagaacagattgtttcagatttctgagcgctgtgtctagagcagactg

13651 ttgaagtatgcttccaggttttcatttttatgagatttgaaatcagattatgtaatccatcttacacaatcacgcttccgtcattttccttatcgcaagttgaaa

13756 cgctcaggtgatcctgatttaatttggttacgtcacgtctcatcatgatgcttcagacatttattccacaagtttgataaatttatcacaatttaattatccttg

13861 atgccaaaggtagaatagatattccctgaatttcaattccgtcgaagctattcaattaccttatcgtgataatccagaagtcgcgcaatcttctcgcccttcaga

13966 gggatatttctaagacgcgctggattggtacttccatatatatctgtcaagtccatctcctgtgaaatagtttcttggtcacattaagatgttaaatctgattta

14071 aatgtcattcttaatactacgtgttgaaactcgtgttaatatatttaattttttataaaattaatttccctgaattttatggaaatatattataacgatctacaa

14176 ccatttctttggtataacataagcatttgaagctaacataagccactcttagagcgttctacaatacataataacatggacatataacttaatacagttagcgta

14281 tacatacaaatgtaaatcaattcaattttgaggaattcgcaggggatgcctactattagtcatattagtgtttgcccgtgtgtatgctgggatgttttgtgttct

14386 gtttgtagtgggaccgttcttccaatgtatcttttatagtgccacctgactttaatgctacaacacagataccgcaacatccaccccagaacgctatgtattata

14491 ccagataacactatgtattttacttagtaacaccatcataacggatgatttggctctgtgttacgagccagcaaatgtggttattttgtactttcatactctgac

14596 attgaatccaactgaatacatacaatacttgtttctctatggctactctcactaagtggagggacgcgataaagagactgcacctagaatcagctggcaattatc

14701 ggcaaattaaaggtccacttcccctctaccgtgggaagaaggtttgggggtcagtgtttccgtagtcggcactctttttgtgtgcttttctacagccaatagatt

14806 tacacaatcggcagattatttgaggctttcagaaataaggaaaaatttgtctgacaattatttcagccggatcaaaagtatctaggcctgtctggggttcttgtc

14911 tttttacaagcatttccgactcggttatgtgtgcgcactaggtagtttgagctcctctgtttagtttagtttaaatcaattttattgcaaaaaatatacaacagg

15016 attggacaaaagttataacaacttacttacgtcctttccataacaaataaaatcatacaaattaagacagtcacgcaatagccttagaacttttcatattattat

15121 ttagttaaagataggcttagaaaatgaggaaaaggaaataaagttggggggagggggagggggccccgacgagtaattctgcggatttaagatgacttatttcct

15226 aatctagtcggcaaagaactggctctttactgtgcacacattgcgttcaacttatctaaaatgttcagtcacatcccgtgctttcattgtcgttctcccgggtca

15331 ctccttgtccttctacgatgctgtactctacctactttgccgacaagattaggaaacctctgtcacatgtcatcacattattgtttgatctgtacacaaccagac

15436 acttttgtcctagctaaatagtcactgactcaccatgtttattacaataagatcgcaaggcgtttaaaagaaccgaatactgtttctatattcattcaaattttt

15541 ttttaagaaacaccatacgtggtgtagtcaggtgtatatttcagtaagttgatatattgtctggtttgtaagtagtatttgtgcgtcaggcgttttgtttcgtta

15646 atttcaccgtgttttactttgagttgcaaattactgaagtggtctcaattaaagaacgaaatcatagggcaatattcgggtaagcagaacattcgtatgtcctct

15751 ccctgtcgacagacacttcatagcgaacatacatgtataatgactgtcggttaatgtcatttggccgtggttttgtttataaagtgacaacatattacgtcatac

15856 tgatgacgtcaagatataaactcatggctagtcatgtacatataaacacgtgctatgcttgacagaccaatacggtcatttgtcagtatttgtatttaacacttg

15961 aattgcgctcaatggcacttcggcgttttggttatcaggttatctccccttgtagtgtcgagcacaagccccgggatatcaaatggtggtgaaaagtttgttgaa

16066 tagcaagaatgtaaaacgtcgtcacaagtcctcgggtcaaagcaaacatttacatggcgcaacgtttcatgtccttcaaaagactagctaattgtttgagattac

16171 gcaatgacccaggaggtgattaaaagtggtccttacaatatttttgagatcatttattacttccaaaaaatgtgcacccaaacatagtacgtcgtgtcgcatggt

16276 taccgtgtcagctccgatatgggcgatgtacgcgtaatctattatccattacacgtgcacgaaagaaaactgttggatagattgcttcgtctgataaactattgg

16381 gcatggattccagtcacagcgggacaatacaccagacgagcagggttttagaacgcaatatgcgtgcacttacttcgtagggatttttttacaagtgcgtgtaaa

16486 tatatcataaatttatctaaattgcaacgaacgaaaccatggtaatcttaatgaccaagtgatacaggtgaataacacgacagtacatacaggagataaacacga

16591 ccgtatgacactgttcagtacagtgtcggctaaactgtatactagtgcacaccggccacactgtattactggctgtaagacccattactggacaattgttgaact

16696 gctacatatctgccttattgcacatcaaatgcacactggctacactgcatactttgcgtactgcacatgtatctaaaaatatatactttatttttcaacaattat

16801 gaaacatatatacttcatttttgaacatttatgaaacaagttatatgagatcttgttggcccggatcttagcgtgcgagggtcttaatttgagaggaaaccgatc

16906 ggcgaaatagtcggagaaaacccacattaccccaaaaccttttacgtccgatcgaggaatcgaacccctgtcgtcttcgtgaaaggcaagtgcgctatccactgc

17011 ggcacccgaccacgcgttcactaataatattcacatataatgcttgtatactgaagtaggaatttctggggctgatttcttgagacacgagtgaaacattaactt

17116 gttatctttacacctactgtatctcctacgttacgtaagacgtgaacgaaacacgtggttttgttgtttgcatgcagcgaaataaagttaaagttgtttataaca

17221 tttactgtttatctgaaatgtgcatcccaatatgtatacgtggaaaagcacttatcctcgcccagaatctaaaatatacctgtaacccattttgtgttctataac

17326 agctgtgaatacagtggtaaactcgcatcaaggtgaaaatcgtctgcaccttatcaccagactcatcgacatattggaaatttataaattcgtttttatctcgga

17431 atcccccgaaagaagtctgatgtatgatcagttgttactatgcaccctgataaaaataaacagtaggaacgccaccggcctgtaccgtctcgagtcgtgttagca

17536 tgggctacatacactgccgctgtttaatgtgaggatttcacaaaaaaggacgctcattattttcgtaaacatttttaattttgcaaaccaaaatgtagagataaa

17641 aaggagagtataaaaaaagaaaatctttttcccgaagaggattagagatgtttaagctgtcagtgctaagagtgccgagtttatttgaacagtgattcatcatgc

17746 cccccacctccccctcctcgaggcgataacgggcgcagaaataacgcggagaaaagcctgaagcatgtacaggagcacattcctgcaagttgttggcaaaatgtc

17851 acgtccttctgaataataaaaaatatccttgtaatcttataattatatgataaaaattattatcgtctatatgacatttacaccccgtacatgagagtagcgtct

17956 agacgtggactccggggcacactgcactccaaacatcatcctagtgcaagcagcacgcgcgattaacttgtatatatttacagtttggaggaaataaaactgata

18061 taataacaagggcttaatatacaaatccttcgatttcggatacttatgagtcatcaatacgcattaaaatataacgtacatgtatatttgtctggatttacgtat

18166 cccttcttacatattttgacctcgcaatttataaatgtcttacgtgcatacaacaaggggtggcgtaaaaaaacacaaagtggattcgtgatggcgagtcttcca

18271 aactacatgcaatgaagtgcttttaaataaataaaaaaaaacgtgaattgaaccctgcgacaagtaaatgtttagtggtaattgttcattaacgcaatgtttcaa

18376 cgggtattcggttgtcgatctcaaaatagtgcacgcttcagccccggaacgagtaaccggaagtacatcgctaactgacgtacaaaacatgtttcttggaaaaaa

18481 aacatcacttacaagacaagtcaccgaacgtacttcaccaaattacaaacatgacagctgatctccgtggacacatagcttaattgaaatcccccatttaggtat

18586 atgcttcatttaacaatcatgtcgcaatgcaggtcatgaaatgcacggtctcgaccaaatcagacccgtcttcaagatcatgtcaaatttgtttatttcgtatat

18691 aaaccaaaatgagacttaagcgactcagaaattggtgatggcttcattgcggaacgatctgaataaatgccaaggtttggaacatttttttcccggaaagatgct

18796 tatttccgattggcaagctccagaatcaatttccatgctataccacgtcttcagggacacttctccggatatttaaatctgggattcatttccaggaatggtgtg

18901 tgtacccatcatccctgatttgtacagttgactgttcctcgctatggaaactgagagtctactattcatatctagggtatgaccctatcgggcatattgacattt

19006 tctccaaaatattacaaattctaatcagaacatggagtgtagtaaatttgataatgtaaatttgaacattcgttttttgctagttttaattgtaatttattttga

19111 acacgtttccataggaacctttaccaaggaaatctccaaatttgtcctgttttacaatttgattatgcaaatgagtagaacaaggctattcgattttccggtatg

19216 aatttgatttgaaagaaagctgcaaacgattgcagaatacttaagtttgacaaaattgcatagaaatttgaaggtttcgtgagtttatttgaagtaaaatgacat

19321 gttttatggttgacagacaaagtttagatgtgctgttcatgtcgttgctagggcaatcattttgaagtggaaggtggtttacattaatatcatgacacgattgta

19426 gagcgtgctttatgggaaggcgattatgatgcttttatgggccgattttcaatgatttgacattcgtgtggtgatggtgtataatgaacgtcagactcgtggaaa

19531 gctggtatataacggataatgttatgtattaacttgtcagataatcgctatggtatgacccgtatattcatgttggtgaatactggtctagaaccaggtttgatt

19636 ttctccccaaatcgataatgttctatttattcagacaacctggaaccgaaatggttggtcttacatctcacgacaatgccagagtctgctataaaccaggctaaa

19741 ttaatagtgacagtcttcctcagtgaaaattaacatgtcagaatagattactgtgatactttctatacaaactgtactggctgggactcctcatcactactttac

19846 tacactgtatctatattacagactatacatacatacgatatcgttctctgatcacatcatcagagcttttaaacaacttgttaatctttgccaaagtggagagtt

19951 cggtggtgcaccttgatgacgtcacggcccaattctgctccctatctgtgtgtaaggcagtaggctaataataagtgggaaatgtggagatgtttgttgatataa

20056 ccactttctgttatcatatggcgctgacatttagacgcatcccaaacccgcgaggttgagttgtggcgagttgactaatagcgttcgtcggtaaaatcaaaacca

20161 gctgtcgatcttcgtgagtaacgctggacattcacgggaaatgatcgaatcaattttgaattcagataacgatttacttgcaagttacattatctaattatagcc

20266 ctcatcattaaaaaacataaacaaatagcgtgggcattgtcaaattttacttcaggaatattatttgcatttagtgagtttctattttatgaatttttgaaacga

20371 gatgacgattcgtcaagtttaatacaaaagcaacgctacaaaattaacctttatctaaaaaaatccgtaatgatttattcgcgcaaaagactaagttttcgcaaa

20476 ccattttgacttcatttctataagggggatgacgtggtattactatggtcagacaatgtcgatgttcattgtacagacggcatacgccgcggtgtaatgttctca

20581 caaaataacaatggtttctcttggttactttgttgttgagtgttgacctgtgtccagtcttggttgaccattacgggcattgatccaaaggtataaagttacgtg

20686 acttttcgactctgtaatacccctgtaatcccgtatccagatctgtctagaaaataaaacatgcattgtcctctctgtgtcaggtgtcacgtgggtcgttccaat

20791 cttcacggattaggacgacggatggccaggtgatatagtataggtcacttataagtcaattggtcactcggcttaatacggatgaccagtgcagcgttaattgcc

20896 tttatcgatgtgtaatcaacgcagtgctgattaaaatgtcaaatggtcaagagtatgcaaatgtcgccattctttgtcaagggcatatactgtatacagctatac

21001 ggttccaactttgacgtaacacgtcatttgcatgtcgctttcgtaacggatagagtgacacgtgatcagaaattttatctgtcctgcgtgccatactgcatggtg

21106 tgtgagacagtttatggtcaatgacattgtatgttggatgataagtctctgtcttatccgactgtctgaaaccatcaaactggactcccgggactaaatctgggc

21211 gacaaagcctccctgttactatgtgatgtaaagtgtaaatcctatggacgaccagtggtcttcatgatttgtcacataacttaaactatagggattggtacggct

21316 gcgacactgaccttgaggttgcacttccggcgcattataagaaatcatcgagacttttaaaaaaaaagtttaataataaataaatagtgtcacatcaattaagta

21421 ttttacgcagttctgacattactccgagtgatggttggcggggcataacacactaatggtagggtgatcgttctcgcggtatttacacgagatctgaagcgcagt

21526 atactctcttacacagcacattacgctcacaaaataaaggtaaacacacgatttaaagttgatacttgcacgctgatataaatatgtattatattttatggatcc

21631 gtctcggcagtaagataaatgtttacgcctgtctgatgtatataatagatatatcgatcatataatgagtctgaatatcatattcctaatatctacttggctttc

21736 tgtaaagcagcaccccgatggcttacaatggccgtcccccgtcacacattccatattcgtcatacaatggctgtttgataatgcataatccggccaaggatgtct

21841 gataaaaaaaattacaaacttcaacaagcttcaacctttacacttcacgacaattgccaaaggcattcgtgcaaaattaaaccaaatgacattcattaaacaccg

21946 tcccacctcagagattggcacacaaagattgttatgtgttcccctgtttaataccacacagagctcgcccatacctcgtaccgaaggtccttctaaatacatttt

22051 accgccgtcagctttgtgtaatcacgatctaaaactacgagtagaacatggattcatacaaatgtcaaatatattattaaaaccagtacgtgtttttatttcaat

22156 ggtcgtataatattaggaatttaaataaacgttaatatatttggatgtctttgggttttgtgtatgttaaagagtacagaaccgagactaagctatataggttta

22261 cctgtgtcacctgtaaaactcccccgccgagaactcttcggtagctaatcgttcctgacatgaaaggcgacaattgtagtttacacgcatataaaaagggtcgag

22366 gctgttatatgggttgggaataaaatgcaacgtcatgggaatgaaaagttacatgtatcgctgttaacccgtttcatgtggctctgtacatggctacaacgatta

22471 tatatataaatatattaaagcatgcattctttttttcatacatacataagctgttggtctaatactttaaactttgatttcacagctgtctcttatcaagaggca

22576 aatattccaaatgtccccttgcaacaattttctgttgacgcatctttataataaaagtattcgatggctttctttccatctatacatattacaagccaatgtaca

47 T Y T P T G F D T Y T A T Y H V

22681 ttttgtaatatgttttgggacactttaataacgatgccttttatgttgtatttttcagACATATACACCGACAGGATTCGATACCTACACGGCGACATATCACGT

63 S D T Y K I Q M S I W D T S G

22786 CTCTGACACATACAAAATACAGATGTCAATTTGGGACACATCTGgtaagtaaaaaaaaaaaacaattgtttcgggtattccgacctactctagatttccctgata

22891 cataagactgcttagcctatgttaatttcaaatagttcgtcggttttaattgaaaattgcttgttttccggtagctttatactggcgttcaaggtacacagtatc

22996 tcaagtcatagacctaaaggttcaagtcgtacaaaaaaaaccggaagtcagcaaaacctagactggaattgtaatcaagatactgactctttaaagaccgcacga

23101 tcaaaggccgtacgtaggtggtgagacgaggtctggagaccgaacaaattttccacggccatgcatgtttcatgaattcttctcgtagatagtttactagtatgc

23206 gggttcgattagatttcgggaaaggtgtgtgtgctgttattacggcagaagcgaacaagatttacgttgtaattatggatatgaatgggtgtatttcctcttgct

23311 atattaaggcgtccgaaatccgacattttgagctaatggtttcatgtattacttgcagaatccatatcgattagtgacagttgcagtacactggcaacaatgttc

23416 acaccaagctggaacacaatatttttatttcctttaatagtttgttataaacgctatgggcagatgcaaaattacggcaaagctcctagaatccgagtgctcatt

23521 tgtcgtttattttttatataacatttaaatattgctcgttagtagagcatccttaaaactcttcacactgttggtaagggagagctgtcgaggagagagacgcgc

23626 tggtttacggggagagagattcaccccaaccaagcaattatgttggtgctctttaatataatgcctgtatacttaatatttcacgctaccctggcagcgatatac

23731 tcgtggtaacgctgttcaagttagtacacagaccacaatttcaattctatacatgtcaatatggaaaatgactcgatatgaacatcttttgtaaacaaaacataa

23836 aatgtgtctcatgttagtggtatcggacagtgagatacaactcttgaacatatccttgaaatttgacttccccacttaagattgcatacaacaagccatagaggt

23941 ctatgataacctcttcaaacttgcagtaatacgaaactacggctagtgcaccggaagtctttttcgtgttgtcacataacgccgacgtcgatttatttccggtgt

24046 ctttgtaaggctctatcagttcaagtgattccgtttcctatttcaagtgttctgtgtttacatgtgacaaacttagtacccaagatgttttctcaagggctcagc

24151 tgcaaaagattcccattatcactgaaacgtgcatcgtatgcacatgatgctgtctggaatgaaacgtttccaggggatatttttagcttgtatgttttgagagga

24256 gacctaacatgtacaacatatgaatggtgtctagatataacggtcattaaaatataattttactgtagtatgagagacataaattggcgtgttaagaattcattt

24361 tagctatataaatatcgtattcactttcaaggcaatgttggttgcaaacctcccatccggttaacaagtcaaaacttatcctgctccgaacatggttaaaatcga

24466 ttatattgccaacatttatactgaattagaaagatcctggttattgaagcattgacaaagtattatttaaagtaaatttttctttggttataaagttgtgattgt

24571 tgcttcatttgctgtaatgaaccgcagttgtagctatggcgcagcccgactctagctgtgttgtactttattgctgctattaataagtgtcacaactgaaatgat

24676 taggttttaggggcggaaatcttgcttacattgttcataacaaaggaaaggcgagaatatttttattcgatttagcgtgatagtgagcggaatccttacaactta

24781 gtatcagcctgcgtcagtctgtatcggatagtatttatgcctcccttccatttaagataagataagcaggcgtgtttgctagtgctaagccctagaaattcctca

24886 ccaaacggaaaacgttaaatttgacattgtgttctaatttgtcacttcacatatatacagaactctgatttgctttgtatggccgtccgtcagtggcccagaaac

24991 ctccgatactgtatccagcaccgtaaagtaatatcgggttgggtgggcagcccgaaaatacgtccaaacctgctactctaggtagcagtatatatgcactagccc

25096 gtcacgatgatccagggtaggagtaacacatgaacatttttgtgttaactaaactggccatcgtctctataatattgaatactaaagaacacgattctatctcta

25201 aaaatcttgataagttggatgtctgatttcaagatctaatctagggggagataatctagtagcaaaatgcagcatagatattcttcataaaaattgtgggggggg

25306 ggtctggtagcaagttgtgttgaaagattgtgtgtaatcacttttcatgtcgtaaacagtgattggacaatggacagacaatttgctagctcatttctcaacaga

25411 gaaactatcataatgtgtagttgtgttatactgcagatttcatttcgtgttgctgcactttaaggcggtattagtcttcccgaaccaacagagcgttgtgccaaa

25516 taagagtattacttactgattatagattagtactacagtaccctagagcgggagtagaacgacgcagtatcttaaagtccagcagcgtctttgtactttgtgaag

25621 aacacttatccccagcagtttccgataattcatatggtgcggaacaatctttgggggttagaaaagaggtcaccatatgaaacagatctgaatgtccacatctcg

25726 gtttgtacttcttggcacaaagcatataaatgtgtatggtttctttgtgcgcgaaacatattttcttcttagttttgctttcaaccgaaaccacatacgtcatcg

25831 acaaccagacgatgccagacagttataaacggaaaagcacgaccttgtgcggaagcagacggcttagatcgggaacaaacgatcgctgtcggaaattgttaaaag

25936 aatatcgcaacattttccagaataggctcacatttaatatattgaaagaagtgtgcaatcttcaacgctatcaattttcgaagttatacagtaagttatataaag

26041 gtttactcacaaagcacgtgaaaagttacgcgtcagtaaaatagggttctagtaataagcacgtgggtaatgtgtaaacatgtgtgaccgaatttgatacatata

26146 gcggcattcctccgtgccttttcccacgatgggaagatgttttggggatggggagggggtagtgaagctcgcagtagagagataaggtttgttttggtggggaac

26251 ccaacggaggtgttgacataagccggtaagaaatggggacgatttggtcgaatttctgtatactgttactacatacatacagaactggtaatgtgaatgaagaaa

26356 cgagctatatttctcactataattataggagaaatttaccccactatatggacgattatgttacaatattaaccaactactgtaatttgatagcaatacaatttg

26461 ttaatagaatactactagaaagttgttctacattaatcaaagtttttgtacaaaaaatgttgagagaaggacggttattgctttatccattcgagtcgaaagtct

26566 actacattgtcgggtcatcgtggttgcaaagttcgaatgattctctttttaatgtaagcttgtcagtatgacaaagtttgcccgttaaagaacaataagttaatt

26671 atcaccggtcaatgttaactgtatgtatgctaaccagactttacacagctttgattaaagtagatgacacccactcaatgctgagtgtgtcgtgggagcatttat

26776 agactttgcggtttctggaaatatttgggttggtcgtatttctacagacttccttgtgtggttataggataggtcagtagctcacgatattaatgtatattatca

26881 taattgaaatgatttaaaaggtaatcttctgtaaggagatatagcagacggtagggtagacctataggcatgtttttgacatttaaacagttggttgctagtcta

26986 ctgacacgcccatgctacatgtttaacaatatacataatatctcgggtgtggacagatggtgcatgtttattcatatacatgtacgaggtgctaaaacaagaaat

27091 cgtgcaaaaaaaaaagagagaaatcgtgattttatacgttaacgccatctttggcttgtgtattcaccataatacgtgcacgtgtatatagctgacaaaccagaa

27196 gtacatctactacctaacgtcagtttataatttcagaaaaccatctctgacagctgactattggtttacccgtgttgaagttttctttccgtatctttatgtcag

27301 aaacaagtacttttgtaagcgagtagcccaataaacaatctaccattcatcacattgtgtaacaaaccgccccgcgtgaagggaagcgtttccggaacgctttgt

27406 ccgagtccgtctcgggttttgttgacggctgacccatgactaaaagttcacgtgtgctctcagagggggtgagatggctaaggagcgcatggtcggatcggactg

27511 acttggttttgtccgttggtgataagatcgtgacaaggtctgccgattgcctggcctttttgtctatacgtgtttaggtgtgacatctgagccgctgatcggaac

27616 gattaggaaacgtctacccgagcaaaaataatatataaaacaccaacctattcataaaattctgactgaactttggacggctaaaatcacagctggcctataccg

27721 gctatacacaaaagtatctgaactacacgcaatattcgtcaacatttaaggccagcaatttccttcctccattgttaaatgtgacggcaacttaaataaaatcgt

27826 ccgatgatttctggatcgaaattgagagttaaaataatttcatgataattttaaaagaatgctttttttcggcgcgtttttcgactctccagttgtttgaggggt

27931 gggtaactccataaattaatagtatgccaaagtcaaccgagtttattcaaaaagtaaatattagagttattaaaataaaatacaaaaccacattttagttcggag

28036 aattatcataaaaaaataacaaggagagtcccacccacctacttggtatatgtaacgtaagacggtattactacgttataaatcggacgggatgaccttttaatt

28141 ctgtactcggtgcagagctattgaagtcacgggtggatgtccctggtaagatagtggggaggacaaaaagtaaacaacggtgttgttgctacagtaaacacccgc

28246 cgaaccgtgtgggaacggattatagggttcaaaaaggccggagctgggtatttcacaatttaaaagcattgtcaactgtcaatgtatatatacataacgctacta

28351 gtagtaatccatatataagaacgcattttaaaattaattggttttattttcttaaataattccgaaacgtcacttctgaatattgatactttttgtgtcgccttc

28456 acgtttggagtatcaagttcgggattttcgtggtttcgttttaaacgattgaatgtaacacctgtataaatccacttgaatgaaagtgagggggagtgaattata

28561 catacatattctataggtgtatgaacggtactgaataaatcacaaacgaggttagtcgggttcaaacaaccgacgtcctggtaatctctaggggcgttcaactgc

28666 aatgcccttgccattccttaaatacgtgtgtattttatgtagatgtgcgtcgtgcattggaaagtatgaagaaatagtttaaatctgaccttttagtatgtatat

28771 ttacattacaatttgtttaattgatcagctgttgagacaatatatatgggccgcggtcaccaattggttaaggcgtctgacattctgctaccacgagccctccac

28876 cgctgggtcgcgggttcgagaccaacgtggggcagtcgtcaggtactggcattcggtagggtttttttcctctgggcactccggctttcctcaaccaccaaaatc

28981 cggcacgtccttaaaagaccatagatgttaaaatgacgtaaaagacaaacaaaacatactttcacttcattaaaggagttgcacagggcgcgtagtttgtgtcat

29086 tttctgtgaatctgtcgttataattgacagacttagtaacattaagagagggcatgtgatgttagaatgcactttacgacttgctacaaaaccacatatggttga

29191 catgcaccaatatgcgccctcttcaccgcgtctttgtcaatggcgacaagtcccccctaacaagcgcaggcattccggcataatgggacgcatgcctctcagggg

29296 aatattgtgactgtatttgatgagctgcacttgcaattctgagtatgcgtggcattctggcgtacatgccgtcaggcccgccatgtctggcctgaacgcgacata

29401 taggtagatttaacacagcagttacaggtatacatgttttcgtatatgtaactttgatcgctctggcgcgtgcaggcgcccctttgataaattcctgattcttgt

29506 tctgtgtaagcaattaaatgtttgaagatgaactacacacaatttnnnnnnnnnnnnnnnnnnnnnnnnnnnnnnnnnnnnnnnnnnnnnnnnnnnnnnnnnnnn

29611 nnnnnnnnnnnnnnnnnnnnnnnnnnnnnnnnnnnnnnnnnnnnnnnnnnnnnnnnnnnnnnnnnnnnnnnnnnnnnnnnnnnggagcgtcagagtgaatgacaa

29716 tccgatggaaactcgctctacattgttgagaggcagtctgccagatgtacggttgaattcagtacttgcacttgggaaggccagaatcaaatacacagcatgtgt

29821 acataatacctaactgttacaaacgagcgacgtctcagatagcgacatttttcgtaattgacagcatttacatgatgcactccatcacaccattatacgacttct

29926 cagcatatgtgtctcgcggtgaaaaatggcgggggatgtctcgatgtcgacttcgtacacgcaacagaacattagagcgcataataaattccaaatccaaaaact

30031 acgtcaacggaatcgtggaacttacgatgcagaatctatgtactttgacttctctataatgaagaaaactgttttgacataagtgtgtttaggaaagaaacgccg

30136 gcttacatcaattccacacgtcgccaagaacaaccaaattctcagaacagataaagattgccagctagaaaccgtccctcgttgttatcatatttcagtttactt

30241 tatgttttgcaattcttttgttttagtttctatatatattagcttctgacataatcctaatccagacccagccgtttttatgagaagattggtacatctgctcta

30346 cacagcctccagatactacatctaatcagaaataattacggttgacgtgtgctatcgtctaacccagcatgttaccggaaacgtagactggggcaatggaggagt

30451 agggtgggcgtttagggaggcaaggacagaaagagccacaggtgcgtgctcatcaaaccggaaagggatgccgacacaggggcagggagaaacggggcagggcag

30556 acagggcatcatcaaaaggcctcatctgtcacgtatgcgatgtgaaacttttgagcagacgatacgtatatttcaacagtgcctgcaattgtcattctgacagta

30661 cataaaaaaggatgatgtatcgggaggccttccacctacaagttgaatccgtacagacaagtggatcacgggtcacgtagcggctgaacctcgcgctgaaggaaa

30766 ggggagagggatagacatgcacacttatatatagacaaagctttgtgttggttaatttgaagaacgtcaggaaaaaatcttaacactctgcaagaaaataaaatg

30871 aaagtttacagcggttagatggtcgaacatatgattatttgaacattttgatcttattcgacacttttgtttacaagtgatttaaaaaaaccccgcaaaaatgag

30976 caataatttaaggaaaaaatcatatcgaatatctagccagttaatcagctgtatttgcctacacttaattaacggtagacaacatcaaacatacatattttaaaa

31081 aaatctttatttgggctacatatatcccaacctgttatttaacagtaatggaaacctagctatgttctgatcagtacatactggcccgtctggtatatatgtact

31186 tgtaagctcgccagttacagcagccaagcgtgacagccatgttggaggtgtggcactgttctcactcgatcgtaaagcattatgaatggaatatttgacactaat

31291 ctcagcgttttaactcttataacctccacatccggattatcttaatctttaagattaaaatgagatttgtataatgtttaaaaggtgttaaaattattcatatgt

31396 atatagttgttttaaaccatgtgaaactcgggtactttgtacttgaatttaaatcccaaatatgtatgcgtcattcaaccacattcgaaaaataaagaaaaaaat

31501 aggaataagacaaaatacacccattcacaatcaaattacttttttaccgacacgaaaattcccccttgataccacagtcctaaaaacaccatcagaccccctccc

31606 ccttggtaccccattcctgagtacaccattagcccctcccccctcctcccttttcctaagtacatgtacaccatcagacactctttggtacccaatatgtacacc

31711 ctcagaccccattggtaccccattcataagtacaccattagaccccccttagtaccccattcctaagtacaccatcagacccccttcatattcccggtctctaga

31816 gtcgcaactgatccacatgaaatacttatagaggatgcatcacctctgttttcctgttctcatctccttctcggatttcaattgttttgattactgcgcatttaa

31921 tatagactcatcatgtaaagagaagtataggagaaaccgtcagataacgcctcactcccgtcgcgctcttggaccatagggagccttcaaatgtctcccgtctcc

32026 gattttacatttcaaatttaattatcggtaaaagctcttcagtgccgggcagttacgataatttagtctgacgcaagttgaagtcataatcagcattaaacacga

32131 aacagaaaactgagctgtaactgttctatggtcattactgtaacctgacgcaagttaattacgtacgctaatctattcgatatttatattttataatgacggaag

32236 aaacctttgactctgagctttgatgatacaattatacgtaatcatgtaccgttccgtaatttccttaagtgagattgatttcggtatatctttactgcatttaaa

32341 aacaacaacttaggttttaacagtcaaaggcgaccggaagtcgaaacaaagtacagaaagaactgagaattgtcagttttgaaaggtcaaggatatgaggtgttg

32446 agagacccaatttaagctcaatggtgtccgttaaaaaggagaaaatctttgttagaactaattgaaagacgagagtagcgtgatccaagccttttgaattgattt

32551 gaattgaattcaaaaaaaatatttagatatttttaactactaaagactgtgcaatgaataaggaacagtctagtatttaatacagtggcggatccagagtatatt

32656 ttgggcgtcaggaccaccccccaccgagaatttgctttaaaaataatgtattttcactattttcttaaggtttttacccacatggaacacccaccccacccccac

32761 ccccaggaaatatttggaaacccctcaagataggtccctggatccgtcactgttaatattcattttctttaacgaaacactttaattgaacaagaaaaacaatcc

78 D S G Y D R V

32866 tttacatgttacataatattttgtattatgatccctgaacgatctcgaactgttgccctgaccgaacctgtttgtatttttcagGAGACAGTGGCTATGATCGAG

85 R P L S Y S D A D L I L V C F S V A D P E T M D A V V S K

32971 TGCGCCCCCTGTCGTACAGTGATGCAGATCTCATCCTTGTGTGTTTTTCCGTGGCTGATCCTGAAACCATGGATGCTGTCGTTTCTAAGgtaagctaacacttag

33076 atggatcttaaacgaactcgcgcttcttgaatattggcccaatgacgttttggagttattgcagaaaattactttaattcaagctgtgataaggttatatgtaat

33181 ttatggataaagttaacaatgacagcgtcgtattgtggttagatgttcatgttgaccaaatgcataaatctgaagtattacggagggtattttgtaacgttttat

114 W Y G E V R E H C P T Q

33286 ctctatgttgttaacgtaactacgattttaatacttaatacctaacatgcgatgtgttttgtttgactagTGGTACGGAGAGGTACGAGAACATTGCCCTACCCA

126 P I I L V G C K T D L R S D T D F T A R L A R K K Q S M V T Y D Q

33391 ACCTATAATCCTAGTGGGCTGTAAAACTGACCTAAGGTCAGACACGGACTTCACAGCCAGACTCGCCCGAAAGAAGCAGTCCATGGTTACTTATGACCAGgtatg

33496 tcggcctcagaagtattcataagaagtatcgccggtaatacgtcataattgaatattttttcatattgtagacccaaaagttaaatgcaagtaattcattttcat

33601 tgtgacgtacctctggcatcgtagttttgcccaataatgatatataaatttgataaataaaatatcaacttctagttggtgaatggttattaagtaacgtatact

159 G L

33706 gtaatgttaaacttttttgaaaactactttagttatgataattttgtttaagtatacatgtatatagtctttttaatatttgaacggtttctaattgcagGGTCT

161 K T A K H I G A L V Y S E T S S K T S H R S V C D V V E V A A L S S A

33811 GAAGACAGCCAAACATATCGGCGCCCTAGTGTATTCAGAAACGTCATCCAAGACGTCACACAGGAGTGTATGTGATGTTGTCGAAGTAGCTGCTCTTTCTTCAGC

196 G N K S T A E T T N F R R Q R S F I K R K R F S G M G E A K V H L R K

33916 CGGAAATAAAAGTACCGCAGAAACGACGAACTTTAGACGACAGAGGTCATTTATCAAGCGTAAACGCTTCAGCGGTATGGGTGAAGCAAAGGTCCACCTACGAAA

231 E A A K S C V V M *

34021 GGAAGCGGCAAAGAGCTGTGTCGTTATGTGAactttgaactatgacctttttgtctcatatcaagatcatagatcatattcttacaagtgttgtgatacttttta

34126 tttatctttcattatattgataacgctagacttagccaaagcaatatttgctcaatgttgagatataagaggagagaaagcttcaccactgaatgagtgcaattc

34231 gttataggatggaccatacacaatgcaagaaacagcaaagtagaccggcgatgtggttggcgatgtcatccatcagtctacaatccacaggacagattcagataa

34336 aagcaagattatacactcatgtgttatgtacaatgataagaagagtgtaaggaatgtatgttctggtaaagatttcccaagtttgaacgaagctttccttggacg

34441 gtatgcttgtatgtttcgatattgacaccctagtgacgtcttattcgaatttcggttagtgagttatgtatgaaccacagcaatggtgcccaaaagctatctagt

34546 cattaaagagacggttgtactgctcattgtatattggattgcgcgtgcgttatttgtgtctgttttgttcgcttccggttcatcataaatcccgtttaatatggt

34651 gacagacgagcttaatcagacatctacattcagaatgactatataccacttgtgtttttaattgtaatttatttatatttattgaattataaaaggaagtgagaa

34756 atgtataaataatacagattgtaacattcaccttaatagaactgattgtgaccacgtgggccacaggttcattaaataaccaagaacgaagagaatttattgtta

34861 aacaggacacattgatgatgtatgagagagtgaatgaggagaacgaagctagacaggaagacgacctgcatcaaagggagataacgctagtaatatagataatag

34966 actggattacttgccttagtatcattttctcggcgctctgtgatacggagcgaaggctgctggatagcagggagaaaactaaggcaggcaaaacattctttgtag

35071 ctaacaacatggactgtaacaacatatcccagagttccttgaaatacatgattacagaatatttcaaggaacctgggattgattaaagctgaatgcagttcacat

35176 gatagtctattactagatcgaaaaatagagatatataatattttgtcgtaggcactttagtatcaagacttgggtaactgtctctagttgtttatccataagatg

35281 aagttttattatagcataatactttgtatattgttaaaaagtaagagttgtttatatatagacggatggacagacaatgtctgtgtgtatacatatagaaggaaa

35386 acacccgtctgtttatttaatatgatgtttgatatacgggtaattctgcagtaagatcgttgtgtaagattattttactttcaaataataagtatttacagaagt

35491 cccaaacataagataaaaaatatggtcggttaagccgtacgacttgacttatatttcttgggtaaaacaatggatagaaataaaggttatgattgagtgtaccac

35596 atttttggaactctcctgttaatttggttatatgatattatagattagtttgacgctggttgtatgtgacgttgttggacgttgtttggcgcttggtaacaattt

35701 tgttataatacaaatgtctttcatcccttgttgttgtctcagttggaagagttatatatataacgtacacttctgccgttgtcagttagaggaatcgtatagagt

35806 acattcatgcctaaacgcgcttccctgctttgcgaagtattaattaaaacaaattgcagtatggcatcataaaccttggagaagttttttgttaaaatattttct

35911 ttaaattaataaaatgatagaaaaggattccgccactggtggtggttagttatcctgtcttaccctacgaaatctcgtataggacaaatacattgtgtgttaact

36016 aaccaacacacgtggagaacttatcaactcacaactttgccatatttcttctggttttccagcatgttacatatctttgatgttcagtttctcattttcccatgt

36121 attggcacaagtcgatatttttaaaccagagaacggttaccgtcgaaaaatatagttttaaagaatacaacatgttgctaggcaactgaaatttttcgtactttt

36226 cctttactacatgtattctggtcttggagagaaaaccaagctatggtctgaataatttattacaataaactatttccgaatgaaaattttcccttgcgggctgta

36331 cagtctccttcacgtcatggaaaacaattatcttcaatgttagtaaaattgtgtgaaatgaagtgggctttcagagggtaaagagagagaatgagaggatacaat

36436 gagaatgagaggtatccgttggatacaatgagaacgaggggtatcccttggatacaacgagacgagatgtatcccttggatacaatgagaatgagaggtatccgt

36541 tggatacaatgagaacgagaggtatcccttggatacaatgagaacgaggggtatcccttggatacaatgagacgagaggtatcccttggatacaatgagaatgag

36646 aggtatccgttggatacaatgagaacgaggggtatcccttggatacaatgagacgagaggtatcccttggatacaatgagaacgaggggtatcccttggatacaa

36751 tgagacgagaggtatcccttggatacaatgagaatgagaggtatccgttggatacaatgagaacgaggggtatcccttggatacaatgagacgagaggtatccat

36856 tggatacaatgagaatgagatgtatcccttggatacaatgagaatgagaggtatcccttggatacaatgagaacgaggggtatcccttggatacaatgagacgag

36961 aggtatcccttggatacaatgagaatgagaggtatcccttggatacaatgagacgagaggtatcccttggatacaatgagattgagaggtatccgttggatacaa

37066 tgagaacgaggggtatcccttggatacaatgagacgagaggtatcccttggatacaatgagaacgagaggtatcccttggatacaatgagaacgaggggtatccc

37171 ttggatacaatgagacgagaggtatcccttggatacaatgagaatgagaggtatcccttggatacaatgagacgagaggtatcccttggatacaatgagattgag

37276 aggtatccgttggatacaatgagaacgaggggtatcccttggatacaatgagacgagaggtatcccttggatacaatgagaacgagaggtatcccttggatacaa

37381 tgagaacgaggggtatcccttggatacaatgagacgagaggtatcccttggatacaatgagaatgagaggtatccgttggatacaatgagaacgaggggtatccc

37486 ttggatacaatgagacgagaggtatcccttggatacaatgagaacgaggggtatcccttggatacaatgagacgagaggtatcccttggatacaatgagaatgag

37591 aggtatccgttggatacaatgagaacgaggggtatcccttggatacaatgagacgagaggtatcccttggatacaatgagaacgaggggtatcccttggatacaa

37696 tgagatgagaggtatcccttggatacaatgagaatgagaggtatcccttggatacaatgagacgagaggtatcccttggatacaatgagaacgagaggtatccgt

37801 tggatacaatgagaacgaggggtatcccttggatacaatgagacgagaggtatccattggatacaatgagaatgagatgtatcccttggatacaatgagaatgag

37906 aggtatcccttggatacaatgagacgagagggtttccattggatacaatgagacgagagggtatccattggatacggtgagaacgaggggtattcgttggaaaca

38011 tatgccattgttattccagaattataaacgagaggattttgacgagcaatggacataaataaatttatgaacaaaactatacgtgtttttgtctattgttatgaa

38116 acacacaaccgaaatacactgttttgtcaattattaagatacacagctccaattccatacagaagactctaccatgtattccattgggtgaacatgtctatttgt

38221 acttctgtacacgccatgcaattaaagtaattgtgtcaactcaatttgtgaaattgacattttccccatgggaagggatacggttacacaatatagctgttgaat

38326 agaacctaatatggaataaaaagtaataataaaattccgtgttgtatttgtcacgcaagtttcctcgctagttgatacaggaatggaaccatgcaacaacaaata

38431 caaaatgtccccaattaatgtatttatagtaaagagacatgctatctgctgacggtggaggctaaaacatgtactcaaatatacgaaagctctactttatacgac

38536 ccctggtacaatactatattagacgatagacagaagaaaagatctttgaacacttatgaggacgaaacaaaagagtagagtcgggcaacttacaaggtcaaacaa

38641 cggaatgctgaccacttgggtgatacatacataggctgaaacattgaacaacgtgttgatagacggagacaaacaacggtatgttgatacacattgacagataca

38746 aacaacggtatgttgatacacattcacagagacaaacaacggtatgctgatacacattgatgtcatacacagacaaataacggtatgttgatacacaatgaggtc

38851 aaacagagacaaacatcagtatgtttttgtgtacattgtatatagtaccgacaaacaacattagattgatatgtgtattgtcatacagaaacaaacaacgttata

38956 ttgatacacattgatgtcatacacagacaaataatggtatgttgatacacaatgatgtcatacagacaaacaacggaatgttgatacacattgacagagtcaaac

39061 aacggaatgtgtatcaacataacgctgtttgtctctgtcaatgtgtatcagcataccgttgtttgtctctgtatcacctcaatgtgtatcaacataccgctgttt

39166 gtctctgtatgacatcactgtgtatcaacataccgttgtctctgtatgacatcaatgtatatcaacataccgttgtttgtctttgtatgacatcaatgtatatca

39271 acatactgttgtttgtctctgtatgacctcaatgtgtatcaacataacgttgtttgtctgtgtttgacatcaatgggtatcaacataccgttgtttgtgtctgtc

39376 aatgtgtatcaacatgccgttatttgtctctgtatgacctcaatgtgcatcaacataacgttgtttgtctctgtatgacgtcaatgtgtataaacataccgttgt

39481 ttgtctctgtgaatgtgtatcaacataccgttatttgtctctgtatgacctcaatgtgcatcaacataacgttgtttgtctgtgtttgacatcaatgggtatcaa

39586 cataccgttgtttgtctccgtatgacatcaatgtgcatcaacataccgttgtttgtctttgtatgacatcaatgtatatcaacataccgttgtttgtctccgtct

39691 atcaacacgttgttcgatgtttcagcctatgtatgtatcacccaagtggtcagcattccgttgtttgaccttgtaagttgcccgactctactcttttgtttcgtc

39796 ctcataagtgtctctgtatgacatcaatgtatatcaacataccgttgtttgtgtctgtcaatgtgtatcaacatcccgttgtttgtctctgtatgatatcaatgt

39901 gtatcaacatactgttgtgtgtctctgtatgacatcaatgtgtatgttgatccacattgacagagacaaacaacggtatgctgatacacattgatgtcatacaca

40006 gacaaataacggtatgttgatacacaatgaggtcaaacagagacaaacatcagtatgtttttgtgtacattgtatatagtaccgaaaaacaacattagattgata

40111 tgtgtattgtcatacagaaacaaacaacggtatgttgatacacatttacagagacaaacaccagtatgttgatacacattgatatcatacagagacaaacaacgg

40216 catgttgatacacattgacagagccaaacaacagtatgttgatacatattgatgtcatacagaaacaaacaacgttatattgatatacattgatgtcatacagag

40321 acaacggtatgttgatacacagtgatgtcatacagagacaaacaatggtatgttgatacacattgactgagacaaacaacggtatgttgatacgcattgacgtca

40426 tacagacaaacaacagtatgttgatatacattgatgtcatacagagacacacaacagtatgttgatacacaatgatgtcatacaaacaacagaatgttgatacac

40531 agtgaggtcaaacagagacaaacatcagtatgtttttgtgtacattgtatatagtaccgacaaacaacattagattgatatgtgtattgtcatacagaaacaaac

40636 aacgttatattgatacacattgatgtcatacacaaacaaacaacagtatgttgatatacattgatgtcatacagagacaacggtatgttgatacacattcacaga

40741 gacaaacaacggtatgtggatacacattgacagagacaaacagcgttatgttgatacacattgatgtaatacagacaaacaacagtatgttgatactcattgaca

40846 gagacaaacaacgggatgttgatacacattgacagagccaaacaacagtatgttgatacatattgatgtcatacagaaacaaacaacgttatattgatacacatt

40951 gatgtcatacacagacaaataatggtatgttgatacacaatgatgtcatacaaacaacagaatgttgatacacagtgaggtcaaacagagacaaacatcagtatg

41056 tttttgtgtacattgtatatagtaccgacaaacaacattagattgatatgtgtatattgaggtcacaaggtcaaacagaattattgcatgtacatttcccttgta

41161 tggtcgaattaattttacatgttgattgacatgcatagaatatcgtttaaagaaacaattcggaaatctatacataagtaagtagggtaacaatatatagatcta

41266 ttaccaagtgcgtcatattcccaccagtgcaattccttaaaagggagggattcttcgctataatctgaattcatatttgataatttcagcattaaagtaatattt

41371 tatcggattttaatcaaagttaaaacagagcacaaaaacattgaatgtagaaaccactttctaaatacttcgtgatcgtgattacacaattattaaattagagtg

41476 ttagttgtttgccatttcaatattgaaaaccgccctttaatctagattaaaataattatcaactattttttaaaatcaactggaaaaatgtcagaattggtcggg

41581 attacaaatttattccataaagaagtattccatgattcagatgccgcggaggccaagtggttaaggcgtctgacattcctgctaccactagccccccaccactgg

41686 gtcgcagtgaccaagtggataaggcgtctgacatctgctaccactagccgtccacccctgggtcgcgggttcgagacccactttgggcaattgccagatactggc

41791 cgttggttagtggtttttctccgggatctgaaatcgtaaatttgacacatgtattttgtttgctaagcttagtcataaaacatcagtacagtcatcttaattacg

41896 gcttctttagatataatcttgtttaaagtgcagtttgtagttatggacatccttgtgataattactatttgaaatgtcaaagttgtgcaaacgaaagacgcattc

42001 tggttcaaaatttaatcaatttaaaattcaaaataagattcgtcagtttggtatggataatacaggcttggtttatattacaccttaaaacaaacttagccttga

42106 aataatttgatgtagtgttcaattgagaattaatttgcttggcaagttttccacagacccacatagttgttgtcacttttgaaatggattgaaaatgtataccgg

42211 tactggcagaatgctgtggcaggttgtaataacgtgtgcctaatgcctttgtccgttttttggcaataaaatatgttttaaactagtgtagtttattgattaatt

42316 actgccctcataatgcagtcacacgtggtcaaacaacgttttccttttgaccatgaaagcgatccaaataaaggttaaatgtttaaccactctatggcacaggta

42421 cctgggaatttaatgttaaaatgttagttattatatatatttaaatcctcaggtacctgtgctctatggcattgtcactctgtaatcctacctaattctggcgat

42526 tttagctcgaaatgcattttaaacctttgagattgtatattttcgacattggaggcacggtagtctagtggtagaacgacggtctcgtgacacggcacggctctg

42631 tggtgtatctttatactttattctacttgtttcagttgactcagctgtaaatgagtatgtggttgggtgctagaatattgttgaatgctagctgtcagcaacgat

42736 atggcacctccgggagtttgctccccagtcgcccagggtgtgttatcaggcaactttattgtgtcgtgataaagcgatgtacaccataagaacttcgattttgtt

42841 attatttacatgtatattaaaaataattttgttttaataataatattgtaattttagcacaaaatgtcataatttacatggaagttgtccaggtatggtgacgtt

42946 aatgaaaatatctcgatctggattcaccttctgcataccaattaaaaaggggaataatgcaatggtgataagttcggcagattggtctcccttgatgaattacaa

43051 tcaaccctcatatttgaggtgagctttagaagaatattatttttttctttacgttcaataaaaggtttactactaaaacttgttatatttttatcatttaatcta

43156 ttttcatgtatatgatttatatttgatcgatataaatcaaaaatatatctaaaaatgcattttaagtgaaaagtatacaacaaaatggtttaagatatccggatg

43261 aattttaagtattatttactagtaattcacttttcttgtcaattaaattgtatattatagtatcaaaattgaaaatgtaaaattcataatttagaattatttgca

43366 ttaacttacataatggaatcaggagttttgttttctttctgaaattcaaaccttagttcgttttggttattagaaaatccccaataccaatatggcggacgcgcg

43471 gtagaaagacaatttaatattgaaaaatgctaaatatggagttaatactgtgttagatagacatctttggtaagtaacttacttcgttataaaatcaagaaggtg

43576 tgtatgtataaagataagttttgtgcatgctttattacaaattttgaactcattttgaatgcacatggggggggggggggggggggggggggtataactgtactg

43681 ttattgtttctttctctttgagttttgttaagatttttaaacgatataatgcaaggcatgtaaattcaatgatgaagttttactttttatcataccacatttagc

43786 aaacttcaatacatttgcacagtttgattattcgtgtctctttctttcataatttcccttttcacgacttgcccacaggggcacggttcgtattattattatatg

43891 tggtcatgctatgcccctgtgacttgccctagaacaggtccagcacaatctgccgattgagctatatagctataaagagctatatatagctgtattgagctataa

43996 tttcttgaatttcaattttcattaaattaaagcaaagtcgcataaaacaataaggtttatggtaatgttgacaacaaaaaacatacatttaaatgttcattttca

44101 tttttcataaatgtaaaatttgctggtcgaccatgaccaaacagcaagatacaagaagatttatatattaattatttagtgtcttatttgttaaacaatgaacat

44206 tagctcagtagtgcttttattacgataaagcgttttcccaactattaaaatatttaaatgcgttattttttggagttaaactgttaaatgcggcctatgctttca

44311 aactgttttatggacacagcagtctgagattaaaggtccattattattacattaaataatggtgatagtgattatgtgaggaacaaatcaataatagaacgacca

44416 aaattcctacgattctaatttggtgtttacatttcatgttgtgtgtattttttttacttgaa

**CfTc10**

1 atattgtatactatataattagtatatataatgaaagagtatttagcgcattcataatttatcgcaatgcgttcaacgcgaaaagcgctaaaataagattaccac

106 tgaaatatataggtttacacaaaaattcctttctgtaccttaaagacattcaaaggcaaaaatgatttacggtactggagaacaggtgcaaaacggcaaaacaat

211 atactcccatttcttcgaagcggggcataataaattgtaaattgaaaaaaacccaaactcctttggaataatgtcggctcagggacaaactctatccccgattta

316 gatagtgagttgcggtgagaaatcgtgccgggtgagaaataggaatgagaaattgtgttgcgaacgcattgattttagcaggttaataaatggtgtcggggtgat

421 aaatcatgtggtaacacctctccaaagtacatttcaacaaaacccatgtattcggataatatacgataagatataagagctaaattgtttttaaaacatcatagc

526 caaggatatataataatgtttaatactaaaatacatgataatgtagaacgatgatctatgggaaagggcagacaactcgtacaacaccacgataaactcaggcat

631 atattatccgtactcatagattaaaaaaaaaataatatcaattttttactggaaatgtaccagcccagaaatgatttcagaattatgctaaaaagggttttgaca

736 aattctttttttataccaaaaggtacaaattcattaacgttgaattttctgaaagaaaaaaaataaagacatttatatcagacaacacatgttcatgagtaaagg

841 gagactactcgtataagttaaacgaatgcacccgtaacatatgtagaaaacgcttcaagtaaacatgaatatttaacgagacgaaagaaacaatatgattatagg

946 tattatcaattcctttagcggacaaagccctgctctttaatgtcgattagaaatattgagtagttttaagctgtacatgatacgatatgaagagaatattcgaag

1051 tttcgtagacaggctgttttttttttacacttatttaatgatcacataacacgaaatgtgtttctctttttgctaaataaattgttcacgctaacaataagcatt

1156 ttctaaaaaaatatagggtagcgtgctctctttgaataactcccactataaatgtcccatatatgtgatgaaatcggcttctccagacttgtgagagggtagtgg

1261 ctttcttaaggaaatgggatgacctccatcaaaattctgagtgatgttaaagactgttgatgtgtggagcatttgatcattctcattaattgtttaaaacaaacg

1366 aaactgtattttccattttcttatatacaatatggttgtaattggttttgataataacagtttttaaaaaaaaacaaaaaagaaaataccaacatttcatccgaa

1471 tcataatctaaactcaaagtcgataaaatcaaggcaaaataggaacaaaagaaagatatacaaggagactcctgaaaaagtacaaaagaataagaccaggtgttc

1576 cggaagagtaagcgtcttctgcttcatcgacggcacccgccatgtaaatcttagtcaatccggtaaagtattgtcacgttcccaaattagaacacggtgaacaaa

1681 ctacatatgtatattgctgtaatataattcaaaatttccattcagatctcggggtagcttcctttgaatgacaaagtccactgaaaagaagtcttggaggccaga

1786 tgtatccaattaaactaaaactaaaaaataactgtgatgcatacgtgtgaatgtccccgctaccacttcatccctgattttctatattaagcaacagtgaccttg

1891 acttttgaactctgaaacacacccatgccagaggtatagtcatacgtcatacttgacctatgaataaagtttgatggaaatctgtcaaggaatgaagtttttatg

1996 tgtaaaaagttgaatagggacgggtagcgacgcgacgggacgggacgggacgggacgggacggacatgggcaaaacggaaaactatatatgcnnnnnnnnnnnnn

2101 nnnnnnnnnnnnnnnnnnnnnnnnnnnnnnnnnnnnnnnnnnnnnnnnnnnnnnnnnnnnnnnnnnnnnnnnnnnnnnnnnnnnnnnnnnnnnnnnnnnnnnnnn

2206 nnnnnnnnnnnnnnnnnnnnnnnnnnnnnnnnnnnnnnnnnnnnnnnnnactgtgacacttaaaattgacccctgtttcgcttttgcaagctttattgtggttta

2311 tttcagactttgcgctatgacaacagtcttctttaatcggagacactcagtctaaaacaaatcacacgtgtgtgtataacgtaaaagttaaaagatgataaactt

2416 ggctgctagatttcttagcttacgaagtttcaaagtctcaaagctgttcacatattattatccgtgatcatagagcctttgcaaccagccaataactttctcaac

2521 tcattgaggaacatacagccggagctgccatttcggcgctaacagctagcattcaacaattctaccaccgcccaaccatgtattcatttacagctgaatagactg

2626 aaacaagcggagtaaagtatcttgttcaaacgactcgtacctacaacctttcgactacgagaccggcgtcctaccaaaagacaaccacgcctcaaaatgtaaaca

2731 ccaatttgaaatcaatctgcactcctccattttttttgtccctgcaagtaggaggtaagaaatgtacctgctatcccaggcatgatcgtaaaaggcgactcagta

2836 tgggatcttctttttaaacacttttctacttaatatataaagtctcccctgacaatgccttacttttgcccctgtgaggaaggctttgggttctacaaatgtatc

2941 tcctggccgagacattccaaagtctataaaagtggcatttcctactcttgcttagcgctttgcaattaaggagtgggactactggttcgcccattgccagtataa

3046 tgtgacagggtggtatgtgctgctgggtgtcttcggcaggatgtttcagtgaggtagcactatacattcagcatcagagacaatacacgaacatatcgctacctc

3151 ccaatacaaacacacatacactacacgcatgtatcttgtatccttaaatgtccttagctgttgatagtacgtttaacaaacaaaaaagaaaataccaacatttca

3256 accgaatcataatctaaactcaaagtcgataaaatgaaggcaaaataggaacaaaagaaagatatacacggagactcctgaaaaagtacaagagaataagaccag

3361 gtgttccggaagagtaagcgtcttctgcttcatcgacgacacccgccatgtaaatcttagtcaatccggtaaagtattgtcacgttcccaaatgagaacacggtt

3466 aacaacggtatcaacaggtatactaacatgcatcaaacacgtccgacttcatattacaaaacgaaacagaattaaaattattaaacttaatcccaaacctagtca

3571 tatgcatttaaatctaggtgttcatgcaactaatcgatccgaaaaaaaaataccggtaggatgatgttcagtttgaagtgcagtatgttgcaattagcgaaaaca

3676 tcccctaatacttgtgtgtgctttatttaaaaaacaatgtgaggcgagcattgttacaacctgaattgtaataacaacatgaattgtaataaaattgtacgttgg

3781 cggtactattgttttacattgtcttacgcaagtcttttatcctaaactacatcatatttcattttagtttaaaatatgcatttagtatatctttaacttatttat

3886 caagaaacagaatatagcggaacattttactttcgttgagactttgaggtttggaagcttcatcatttaattagtatatgtattctacacataaagcacaaacgc

3991 actttgcataatccctaaatcctataaaaaagacaactgtatttctgggaagccaactattggaatgtgtgagcatactgcttgtatattatactctcgtatacg

4096 ttctctatttaaactaaactatcttacatatatactcaaattatgtatcatttaaactgatagcatcattgtaatcaaaatttaaaagcatttttttatatacaa

4201 aaataaagtaaaagaaattttagggcatctcgaaaggatacaacatgtattcatttcacaaaatgcatgtcaggccctgtgggccttttcatcttgcatgatttc

4306 aaataaaacgtattgtttttgttcagctattgtatgattgaagaacttatgaagtttcatgaaaatttgtgcgtgggcagggtctatgggaatttttacataggt

4411 accctacctttaaaaaaagtcaattcaacacgcgagttataattacaactatttcttaggtaaatcaatattgttatttaataagtgaattgaatttattacgat

4516 agcggtatgcaatatagatatattttacatatatgtgtgtgtatatataccctctcggtgtttcgtggaaactcccatgtaagctgagacatatctgatgtaagt

4621 ggtattttccggtaacggcccgggatcctcgccatcgacaaaaaggttaaatctcggctataaatagaagtcgtttgattggtccgataaagaaggcgataaatg

1 M S D D G P

4726 atgtcatacttacaagctgtggagcgagagttgtacacatatgtattatacgtatatgtatacatgaacgatatagtgtggcaaacaccATGTCGGATGACGGGC

7 K F I K C V V C G D G A I G K T C M L M S Y A T N K F P T E Y V P T V

4831 CGAAATTCATCAAATGTGTTGTTTGTGGGGATGGCGCCATAGGAAAGACTTGTATGTTGATGAGTTACGCCACAAACAAGTTCCCTACGGAATATGTACCGACAG

42 F D N Y A V

4936 TATTTGACAATTACGCAGgtaaggcaaacccgtcccatggccaaacggaggcacaaacaggtgtgtggccaagatactcaggtgtgatattggggtacacgatgg

5041 ggaaactgacaatagtctgtaatacacgtgaatgtttctctcttctactaattatgatgtcatgattaaattactttttggtggcgggttaattataatttttga

5146 ttgacaggtgtgtgtatatctcgccttacccggaagtgttgtatacatttcacatccaaatttaacgagtgaaatgagcagttgattgattgtatatataaactg

5251 tctggcttgaggtgattttgtagtggtcattagttacatttgtactggtcagtgatgtccgatgaactttatctgaccatctacacacctgtacggcgactgaaa

5356 ttgcctgaaggacaaacagccattaaacttgaggtctttataaaagtatgtaaatttcttgatatttcttgaaatggaatttgaaatgtgagtcagtgttgttca

5461 acgcatagaaaatacagaagtaaaaacagcacggctgtatagtgacatacagtttgatgaaatacacagaggtattgattgatggcaagggacactgtcaagtcg

5566 gggtcacacggggtcaaacatcttaaatcctttacaccatagacatacagatgctctaaggtgttcgctgtaatttcccttccgctgtttcattgatatataatg

5671 aacaaagacacctttattccactttattaatttttctattcagccacacaggttgtaaattacaggtaaaatgcattgacgtttttgaaagataatatcatacct

5776 aagctgattaaacctaaaggaattctttattccttttattaaagctaaggaattataattacgttttctgtccggggacacaacgggtttaattatgattgcgtg

5881 cttgactggtattgttgtgaatcaatgcatattttaaccagatatcaggactattatgtaaaattaggacatcactataactaatgtttcctgtaactttgaaaa

5986 aaaagagtatgatttgtggtaatagaaatatttcatcttgattataagtaaacaaataataccaaaataattatcatattgatatttgacaaaatataataaagt

6091 ctaatgtcagtttcatttttacatgtatgtatatatactatcaattttaattaaaaacacatatatatttactttggccttggtagatccgggtctaattttccg

6196 gtgtcggattacgtttaggatttatgagtttcctttttgttgttattgacattttttttctgtattgtaatgttgtattattgttgtgttcctgtgcactcctag

6301 ctagagtttgctgtcactaattaacacttgtcaggagtggatgggtcttagtcagaccggaagttgaggacaatgacatacatcgtgggaatatggcgacatgtg

6406 aatggcctcgcgctgtcctcagctcatgatctgttgccacttacgttttaatttgacacgacaattacaagctacaaccgagtatatatatatatatagcatcgc

6511 ccttaggtcgacaaagcattttagacaactaaattgttttagaacgacagagaaatttagggtcaattgaggtcaagagtgacgagcaattgcacgtgcttgtcc

6616 actttggctgaatatttcacactaaatacaagtacgaccatttcataggaaatatttacatatgaaaactatatttaagtaaactttttataaggcatgacatat

6721 tttcaataatcgaaataagtcaaatcagagacgtctctggtcaaatattcaccatattttataatcagtgtctcattggcaaaaaatccatcaggaaatagataa

6826 agatcgttttatgagtgcatacaaagagtttgggtgtaacacagaaccaacattacgtattgttgacaattttgtttacgtggccatcgatattgtttacgaaca

6931 cgccaatgacctgtggtttacgtaaacaaactatttgtacaaatgtacaggtgggacgtcgcgatcggtgtctttattcacgtcttttcccttttattcaactgg

7036 ctgtgattgttaaaaacttagtgttaaagtccttgtggtatattctgttctgtttattaaatctttatccgcgtctgataatttaaaaatatttttgatcacctg

7141 aggctaaaatgtcaatttatgactatatctgacaaaacataacgtattcattgtaggtgaagtcgtgtgaataatcgaccttaatccacatatacaagacacatt

7246 tcgtggctgttagttttaataattaacttttctttataatcaaatctactgtaaatttttgtgttgtccctgtgtccacagggacctgggatcagttacaatatg

7351 catgtttagaatatcttcagaaataccgggaatattattttaagtttagcatgaggatatttaaatatttagttattggttctcgggtccctgtgcccgtgctcg

7456 gggctattttcacaacacgtgacatgatagagacggtgagtgtagaagttgacctgtcaaatcaatttcagctcatagttagatactcataacggctccaaaaca

7561 cgaagttacaaatgtttatttatacttaaaagcattttctgtcatataattgaaaagagatttgccacaagttgtacagcattattataggcaggattgataata

7666 aacaattagggatcgtttagaactgaaattaactaatgaaatggacatgtaattgaattgtggatatctgttttcaagtctattgcaatattagttattcctttc

7771 agtgtgggaggttttctaacttacaaatacgatattgtatgtctagcaaactagtgtattatgcacgcgttataattatgtgtttttattacaatgctggtctca

7876 cagccataattaaaaacctcgtgtagtttagataatatactatcaagcagagccaagcatcccctccatcacacgccagacaatggctgtttctgtcagatgaaa

7981 gcgaaagatacagaaggaaatagctgatagctactgtctacatagtatacaagtcggctcaaatgttatcatttttacaaggcaaatgtcgtgaaccctttgtag

8086 agcaatctgatgatttactagcaaacttggaacaaacaaacccacggaatctttaagtgtcttagacaaatccttcaagcttctgtttgataccaaaggcattaa

8191 aaaatgtgcacgcattccgatgttttggatagcaatatgcattgctcaaagttataaaaaaagttaggaagaatgatagggcagatgtataaaaagcaacgccaa

8296 ggttttttttgacagttataatgaagacatatttctgttgtaatattccctgatatatcaatgaggtgtgtatttcccatgatgctacaggtcaaaggtcagcgt

8401 gcatagctaacaagtgagttaatacagtgcattgggcgtgtgttattatgtgtaacgtatccgatacgaggaataacggatgtttattggtataaaacgccgaac

8506 aatacacacctatatctgcattgtgtaccacagggacttgaaatatcgtcacgtattacaaaatatcctgattgcataatacattacagttgtttcatgtgcata

8611 ctgcataactaaacacacgagcccgtagaagatccatccacttatatttgataaattgccagaaaaaacgctaataattatgtataattttttacgagttctata

8716 cgcccctgtgcactgtacgtcctccccctcccgctctgcattaacccccgactcacacgcgcgcttgcatctgtttactgttgcagccaaattggtaacttatgt

8821 tgaggatacgagctttcaggatgtggcgatatttccttagaagttaactcgttaggcagtcggtctgttcacgggaccatggagcgaggtgatataattagggaa

8926 aacaatggccgattatcaggttgccatggacgcgttgaagtgtaacatgatacgtacgtgtcctgtagctgcatatctctacaaataaaatcgattttgaacagt

9031 gtccctgcttgtaattacgtatgatttataaattacccaggtgacactgataacattagtctggcggttaataacacaggggcctgggaattaatcacatctttt

9136 atatttttacaccaatcggtgttttgatcatgatcagcaactaggttaactgttcaagaatgacagaaatgcagatccaatgctcaaaagcagtcggctttcatt

9241 tgtaaaattttgtaaaatcacaattagatatgttacccccctccctcgaacaaaatattcgtataagtaaaatctgttttgtagcaccttatttaatctcaagac

9346 gttcatggtattttattaccactatatgcataaaggtgtttgaagatgtgtacgtgttctacttgttatagaaccgtttttcccaaaggctcaatactaccagac

9451 ttcccaactctcacggctaccagtattacacttgaaaactacatcatagtaaccgtttgaattcacaccatataatactataagcttggcgagtaaaacttagca

9556 acaaagtttattgtaatactcaaagatctcacaactgtctttcctctgcgtgctccgaacctacgaccttacagtcaataggccggcgcaccaccggctttgatt

9661 tgttaccctttacaaattcactttaaaacaacagttatctttgttttacatgtcatgtttgatcgatacattcataattatctatatttacctgcgttgcgagac

9766 agaacgtttgaaccctacaaattatatccggtgtgccgaggccagaaggtcatatgtaatttaaatatatgaacatttcacctcctaaacacaagaataaaataa

9871 agtaaagttgaatgcaaatgaacgtaaatcaaatgtcgggcttgagcaaggctactagtagtaccagactgcttcgttttttatttatgagaacttcgaatcgat

9976 tgaagcagctgtgaatataaaatcaatcttaagctggcgaatcgaacttcggaggtctacgtgaaataccatcgatcctatactaaatgcaaaagtcaacgccgc

10081 taccaaaaatgtcaatagtatttgttgtgaaacgataatcgttaacagtattttttgtgtttgacataatggcggaacattagacaagacagattctctcataaa

10186 ccaactgttgcctacagggcttggttttccatcaacattgtagtcacgtgtttcgcatcgcttacacgtgccgcacacacgaccacctgaagcgccttcctttcc

10291 cccatttcggagggcacgtgccaacagctggtaggtgggttaaaataacattcttaaatgaatgtttattttgaagataggtgagctgatgttgccgcttgaagc

10396 atatagcgtacacaagaagctgaattcgatcacatattttatttttatttaaggaagttagcgataatatattttagagacttgttacgtcacaagagaatttca

10501 tatcgatatttacatcgccaatctcaacaatccgtgaaggcgactgataaaagaagaaacattccgaacactgataacatgtctggctgttaggcaatctgcagg

10606 atctcagctatactcggtaaataagaacgaattgtagcctagggcttagtaacaaatatcgctttaaaatcttgtagtacacaattctagattaaagttaacagg

10711 aactttttagctatgaaatttgtttcctgtaattcgtcaatgcacgaggatatccgtagcggtcttatgtagtcaccaaaatattataattcccggattttatcc

10816 tcttccgaatttatatatatcagaaaagtcgtaaatgcactgtagtgtaaattaaatagacaaacaacatttaagtgccggatgtaagtcccaaccgctcaatcg

10921 ctatacttgtgcgaaaatacagtcaccgccgtacaattttacaactttgcatccaatttcacagccgcataaatactaatttaggtcacctctttaaaatgcata

11026 tatgacagtttgcaagcagtttagattattattaatcgatgggtccataaatactgtcaaaattattttgaactcatttcacttaagtcgttgtcggctgcgcgt

11131 cttgcgattgccggcttcgtcaccttgcaaacaatgtactttgatatagatgttttctggagtgaaagccctttttaaacctcggatttattgtcttttgtaacg

11236 attcatttgttgatatcgaaaaagatgtcaagcgtaaaaataaaatgtgttccgagaactcacctttggctgtttccggctgccgccatctttgaaacagtccca

11341 actttaatcagcgtgcagacgacgctatagcgatatttgtgtttataattagtttcgttcaagcgtatttgtgaaggaagatgtttttatttttaaacaaaaggc

11446 tccacactgtaacaattagccagcaaaattagttgtttgaaactttctcataattgttttgcagctacatgaatgtagagttaatttgaagataattagcattcc

11551 gataatttggggacattttgggaaaatgtactgaacttgttatcaagaaaaatactttttttggaggatattaaatgtattaaatatcaacaattgttgacgatt

11656 atgttgtttggctgggctcaacaattatcattaatttgatacttgatagcgcacatgtaattaccacattagttttatagtcaatacgtatattactctaacaca

11761 attgaaagctagccatgtttattatgttgaaaatgtgccgggttaactttgtaacataaacataaaacttaacatatagggaagtatttctcttgccgccatctt

11866 tcagtttcattgataagtaaaccactgtttctaatagtttaaccaaacataaacatgaaaatgattctcaaaatgtcctcatcactattccagcttgtcaactgt

11971 aagccgaaacgttcttcatcaaggaattacttctagaggacgactcttttgattgtaccacgataaaaatcagaacacagcaacactccaaataatttattaccc

12076 agaaactagttcagcgtttagatttaaaaataatctggtacatatagattgttttgattttgtaaagtttaaagttcaattaacagctacggtcatttaaggacg

12181 gaatccggcatatagctattgattaattcgtatgcgtgaaattgttttagataggaggaaatcagtcattgttcaagttagaaaggatgtcaaactcaaaacacc

12286 gctatcttgaacatacatgtagtatcgcaaaaggccacagtgggattaagcacagcttaagtgccaaaacatcctggagctcatgaatgtctggaccgtgggaaa

12391 tgggatctgttatttttgttacgatacttcatccatttcttgatgatgtgttgtaaagtgcattttgtgtaatcctgttaattaatcagtgattttaacattaga

48 T V A I K D Q

12496 ggctgaaaaataaatcgagtgcatttttttttattatgaaacacgcatttcatgtactgtatgtataatttgattgtttcagTAACGGTCGCCATAAAAGATCAA

55 Q Y Q L G L F D T A G Q

12601 CAATACCAACTGGGACTTTTCGATACAGCAGGACAGgtcagtatatcccgaactggtgttgtgtttccttatcttacagtattgatgaggcaaaacaaccccngt

12706 taaataatagattggttggttgcatgaaatatttattcctccacttgttagcaataaggatgaaactaattaagtctcccataatcacagagtcgtttgcctttg

12811 tacaatgtatgtatgtacttctaaagtgttaaatatccttcctgaatatcttataaatatatagctctgtgatataaagagacgatctagcacgagtattcatgt

12916 aatattgaattcatagaacgagcttattaattgcatatgaaataaacaatattcgcagcatggttaggaaatatacagcatgaatacgacaaaaaagcatcaaag

13021 attaatatgattatatcaaggagtacaaataatgatgataatgtcgcagatttaaccagattaggaatgacacacgtatacattatattattgttttaaatgttt

67 E

13126 tgcaaacataaattttcaagttgcatttggaatattaattatacacaccaatgtgtgtctaatgatcgcgcattgtttagttattctttcgttttgtattacagG

68 E Y E G L R R L A Y P G T H V F L L A F S V V M P E S Q Q N I E L K W

13231 AGGAATATGAAGGTCTCCGTCGGCTGGCTTACCCGGGAACACACGTATTCCTTCTGGCATTTTCTGTGGTCATGCCAGAATCGCAACAGAATATAGAACTTAAGT

103 L P E I L Q T L P P P A Y L I V G T Q V D L R E N E E I R Q K L Q K R

13336 GGCTTCCAGAAATACTACAGACACTGCCTCCGCCCGCTTACCTTATAGTGGGCACGCAAGTAGATTTAAGAGAGAATGAAGAAATCAGACAAAAACTCCAAAAAC

138 R Q K P V T I E E G Q R F A K R L D A D C Y V E C S A L T R Q G L K D

13441 GGAGACAAAAACCGGTAACAATAGAGGAAGGACAACGATTTGCCAAGCGCTTGGACGCTGACTGTTATGTGGAGTGCTCTGCCTTAACAAGACAAGGCTTAAAAG

173 V F D E A M I A V L D P K K R R R P P K K R R L K C V I L *

13546 ACGTGTTCGATGAGGCTATGATAGCTGTACTGGATCCGAAAAAACGGAGACGACCACCAAAGAAACGGCGCCTCAAATGTGTTATATTGTGAagtggacgtccag

13651 ctcttgtcgttaaaatattatgatccgttgatcgcttgattagtgtaaaccggatattcatctagccctctgggtcacgactggctaagcgtgtaagtaattcta

13756 atgaccaggttttgaccatttaactgttaaattctggtcgctgaaggacagccgttagagatctagacatccggtttaagcgaacatctttgtggtccgctgtga

13861 gaaagaggtgattaaatctcaagcaggaagcaaatgtgatgtaagcataatatataatacagagtgatataaatttatgtttcttgcgttctaaatagtaaaagt

13966 attaaactacagattattggttcaagtaatgtatttcttggacacttggatggcgactcgaaaaacaaacagcattgttctgataaccgccctcgcttgacctga

14071 ttacacacctagatgacgtttacaatatggataattatctatcaatccacaaattattagctcttatctcgcattaagtgaataagccagtatgttctttcatct

14176 tttaatataaaccattcctccgtttttcatgtgtgcaatttgaatcattccttccgtttgttcatgaacagtcctggtcacaatggtgcttcggtgatgccgaga

14281 tgtgatgtgtgtattgatttcaacagcccatctttctctggtgctaaaaaaactaattcgtttctattcttgcgacagtggcaaaagcactgttcaaccttctcg

14386 aagttgaaacaaaaaacatgtctccgactcaagattatgtctgcatagacgattgcattaaaattgcattactaacggcaaaacaacggttttctttgaaggaac

14491 agcgagattagtagttaccaaatgggaaaacatacgttcttgttgtcttactgactgtggtgtcagctcatcaatgaggtgatcacagactcgtaacagagcatg

14596 tctaatgggaatgttgtcataattaattaacattgttgttcagtaaatagggtacagtctatggtgtgcattaatgtatgtattcctcgaaagtaaaaatctttc

14701 tgtagcaatgctatatttctggtcgcgaattggaatagggacaaaaacgacagaaattcgagcgatatacacgacagctttctttatcaaacttaaaatgacaat

14806 tcagaaataacattctgtagataaagattaagtatgtctctagattacaatacgaattttgaaatgaactgtttttcttcgtatgttgtttcggagagtttcttc

14911 atcaatatatatcaaaggtgctatgtgtttggatattttcactacggtgatatacttggtgttcagggatagatctggctgcaaaaataactctttaactacttt

15016 aagcttacgtaatcctaaattcaaaccggcaagagttaatctttctgtgcaaatacttaaacgaaatatgtcaaaaggtggacatttatcgcatttcaagggaat

15121 tttacaaattttgatttgcaatatatttgcaaacgatatttaaagtatttttacaaaacataagtgcttggaccagtttttaaaaatgctattagcttaattagt

15226 aattagcacaagttttcatcagtgtgtaacgttaaaacaaaaccattgaattgaacacaatttagcagatatcatataaacagcagagtaaatgcagatgcaaaa

15331 ttcggtgatctcaccttgaatattgacaaagttatctcagattgaaaaatattgttaaaggatgatttagctaacctccttttgaacaactgaaacttgatgcat

15436 atttcagtgattatccacaaagtttaatagaaatgaaaggcacatatttatatatatttttaaaattcatatgaaaatctaaagttgaatcagatgagtattttg

15541 ttgtgcattatttatgaagtgttatatcataactgtatatacattttataactgatcaatatttggtatatgatttgtctgaagacagaaaacacatcaaatatt

15646 aaaactgtcttagaattgacaagtaatctaatacattgcttcttttttctcgaaacaccttatatctattttttttggtatgatttcaagggtcgctttgttatt

15751 ctttcctttgggtatatccctccatttgatatatttttagttctatttatggttttgttccatttctgtattcccttttgttgttttctggtattttgttcagct

15856 gtttggcgttgtcctcaattaaaaagaatatattgaatttaaacgacaaattccttgcaaataatgtgttcaaaacaatttgctcaaagaaaataaaacaagata

15961 ataatgaccgaagaagagcttactacattttcggtgcaatatctggcttatttatatctaatctatactcttatagtgcagatgttcgcgtggtttgctttgagt

16066 aacatccacaagagcatgatttatttattactctagataaataaaatataaatatttaagaaccactaggctttgtttaaatagtgacaaaacaagaaatatatc

16171 tttgtattaaagatgatataacatatacatttcagaaccctttttgtaaagattgatttatgcgctatcgataaataaaatatagatatttcagaatcacttggc

16276 cttgtttaaaaagtaacagaagtgaacatctatttttgtattaaagatatttaaatatatagagttcttccatcagtatgtaaaatttatgtcggcatgtcttac

16381 tggtagttatatgtaattaaaagagtatttggaaaattcaagtcttttgcagaaaatatatcatatgctagtcagtgaagacttttttacacggatgtttttttt

16486 aaaaatatattgcctcgactattatgattttcatacaaatgtaatgaaattctaaatagaataattaattttaatgccctttataatattgtctgtatggtattt

16591 cactaagatccccaggtcccagtcttcagtaatttattgacgaagcactgcttgaatttgtaacctcggcagttttgcagcagttagactttcgcctttgtcact

16696 tgctgttctaaggacgaatttagcacaagaacaaatgttaatatacaacacatcataacataataatttgtgaagggcgaaatgtttcacattttaacagttaag

16801 gctgaaagtttaactgagcgaaaattctttttagttcattcactattcttggtttctcaggggttacgctcaccttcgttctctgaaccgcatggatatgtctta

16906 tatgtcttagaaatatggaaggctagatgtgcacataatttgactaatttgatttcttgataaagatcaaaagaatctcgcggtcacgtaacggtacgtattgat

17011 agactttcaaagtagacctctctctattgtaatcttcaaaggatacgtatcacccttgtgattcgttagtttgtttacacccaataccaactctttgtgccttca

17116 attttgcaatgacatatttcaggggtgtagagatcgaaagcaagcgtaactttcggatatcgaagattttaatttctatgcaaacctgtctgttttctattttta

17221 tgaacgatgttgtatgttactaatttgtcgtatttgaatgatttgataacaattttcacatggattcattttacttttaattcattatttttgttatggattgaa

17326 ttaaagacgagcgcatactaatcaattcattttaagtacgtttaaaacatttagagtgtgtaattaaggctgaaaacaataaccttcaatttgaatcattttcag

17431 atggaacacgaaattgtttaataaaaaatataaatatatattaaattgaaaatgttcatctcatggaatgcaaaaacagatataaattttatcatattatccatt

17536 tccattaaatagtaattttgatatttttataatgataagataaacaccaacatttagttactattgtcaaaaatcaaaggtcaacattaagagtcattttctata

17641 tgcgttatgacgtcaaatgctgacagaaaaatcataaagtacatgttgtcatgtctatcaaaatatcatttatgcaacagtaccatgttatatacagtaaaacct

17746 cattacagtgtataatgtcatcattattcagagctacatatatattgacacattaaagggattcgacaatgagttgttaaacgagagataaaggaaaggtatata

17851 ggatttttttttggaaaatatgaaaaagacattgcaatgtattggcatgaaatgagatcgaggttttgatatctaacaaacaaatcgatgtaaaagtaaggtaat

17956 atattgatatatacatatcagtatttataggaaggatatttcagttacagtatgttatctttttatacttaagacaggaaacaaatatatttgtaactataagat

18061 aaactcttgtatattgtgtacgtcggcgttaggtggtccagactgcctcatttcccaattttcttacattgtacatattgtgctctacctttcaagataatggcg

18166 catcactatttccattaatcggacatgcagtaccattataaaacgaaagtaatatgcgggttttgatcataaatctgacggtgctatttacattgatgaatttac

18271 aaattaaaactcgtatcatttgtctacataagtgtaaatacgagatattttagtgaattcttttacaagtgttaagtgaatgacagaaatcaggctgggaaatat

18376 gatgatacatgtaagatattcagttgaaataaaacactaagaaacgcacattgcgagataatttttatactgctcatcgaataggaatttcgtccatatgtactc

18481 ctcattgaatacttaacacattagccgataccgtttaattttatggttatgtatgactagtctaatgcttattcgttatgtgctatgtacatgtaccctcaagta

18586 gctatcctgggcgtatttttattattaattgaagaatgatggaaaaccttcctgaaatgatggaagagcctagggcaatgacgaaatatctttatatatagaaat

18691 taatccagtctataaatagaatgagtcagtatctttgtaaaaattggtttcatactttgagtaataaggatctcaggaagcacacctccctgaaaaaggtgacac

18796 attagatacaaatcgtcgtcttgatgaggggaagtagtagtcattcagccaagtatgatttagtttttgcaaatctttcaaaataatgttgagcggaatgtgact

18901 taattgtaataaaagactaatgcgattgttttactaaccttattcttctgtttataagggtagactcagtgctaataataagaatatctgcagtaaaatataaag

19006 atagattctttcctgttgttactatacttacatttcaaatgtgttcaagcttcagtatttgtattgtcgtcttgaaattgttacaaatatattgattgtaaatat

19111 tttgatttgtaaattttgattttgtcattattttgaatgtgtattgagcacatcatacatcgaaatgtttaagcattttcattattttatcattttcccctttaa

19216 aaaactataaatgcatttttgatttgtcactttacataagaaaccaccgcgttcaataaagggagcacgctacctttttggacatattttagggtagatgttgtt

19321 tttctatgacactaaggagttcattctacctttctgagacaaacgtaaaaggttattgctaactttctagaacttacttcaatataaatcttgaaataatttatt

19426 gtttttcctatttagaaacaaagaccgctgatgaatgtaattattacgcaataaaatcattgatgacgtaaaggaagaaaagtacaaaattattgacgtagtcag

19531 gggataatcctgaatattcaataaatgcaatgatcgaaaaatcacaatcacacgtgtaatgatcacggtgtcatgtaaccttatcgcatttacaaacaacaatat

19636 tcaaatctgaaatttatacattatcgtatttaaaagagatatacatatgcatgttcattggtaaccggtgcgttagcggaaatattacagtcactcctatcaaca

19741 ttgtacaaggaatgccagagattgtagataaatatattgacattattatgtaagatgaattcacagacggtgtataccagagaaatgactatatgataatgtgtt

19846 aagtatcaaattgttagtttgatataacacatggagttttattttacgttgtgttacttttaaaagaagattcagaaacaatctaataaataaaaagaaattaaa

19951 tacttttatgttctttgttttttgtttatttaacgataaatatatccaacaaatgttatcttgaacaggttctgcattaaagggactaaatcgttaaaatttcat

20056 ttcgtaattgttaaaaaaagttaaacgaattaaaactatgtaaaaagttatgtactttgtgttatttataaaatttaaaagtgtgctcaaccaataaaaaaatta

20161 ataataaatgaataaaataagataaaactaacctagtattgttgttaaaattattcatcggttcgtcgatgtaaacaatgatttgcgcaggcgcgatgtgggaaa

20266 tttaagtcggtccataacagaaatggcggagtcaacactgaagcggagaggtcctaaatcattccttaccgatcgcgagagacacgaaagaaacgataacatgat

20371 tggaattatgcaaagacgggggtttatctagaagacagcacagtcagatgacatcgattaatatagtgacagaggggattaaatgaggccctcagtcggtcaagc

20476 tatcacttcactgaagttcacaggtaagttctgcacttgacttcgatgtttcgtaaaataactgctgtgtcataccatgtttgagatgtaatgtggtcaatacga

20581 gtataatatgtaattttatccggtaacgttaccaaattaacaatgatgagtgacagttgatgctcacacagaccaattgcaaaataagcacgtgttttaactcca

20686 cgtggcaaatgtttacacatgcctcggggtctatttcgtcgggtgaattgaggtcacgcgatgtactcggctcacatcgatgtaaacattgaatggcggtagaca

20791 cgccttcaagtgttgttcagcgatatattgaagctgataatcattgcaaatatttgacaagaagtgccataattttgtaaacagtctaatttcatgtattacaac

20896 gtataccagaaatctcgtgcatgaagaaaattgacgaaatctaacgatttagcccctttaatgaatcacatgtgacatgaaaacagtatacgaaggaaaagattg

21001 cgtgttactatagagtaaatgaaagctaataatcataggaatgtataaacacacacttcactaaccaaaaccagtatcggtctcagatttattcttctgagtaaa

21106 gtgatctatatgaaaataaaactctcgttaaaatataacaaaggtgtttaattatactacacggtcacattaaaactgtatcagcaattgtgatttttacatata

21211 caaatgtacattctattcaactttggtcgatcctcaatgcgttcctgattgtagcattgtttcaaaacaatgcatgtgaacaatttgttaatagcagttatagtt

21316 tcttatgccttcatttgcattttgttgttagtaagctgga

**PyRhoBTB**

1 aaaattaaagttccgagttttgacaccatttctgcttgctagtctacaggaccaagtcaatagattatatgtaattaagtatacattaatgtaactagttttaaa

106 gagattttctgaaggaaaaatccccagcaggaaatacaaaagtgatttgcaagagaactatggtgtagtccatgtaattagacaatgatttacacgttgatgtga

211 gatgtaatgtgtctgtaatttaagctgtttatataccttgtatttggacaggaatattattatttaacaatctatcagtcaaataagatcacaataccttataag

316 gaacatcatttttacacatccaaatgacaagtttcaatctaagttaactgattcaccagttcaacctgtatcaaaaacgttagtatcaatgttgttttgatgatt

421 gatgacagagccgagttcttggctgataacaaaataatctgttgaatctcgaaaatggagatacactttacacttttcctgtaaaaacataatcataggaaataa

526 ttgtcctggtttttttgggactcttttttttttggggggggggggggtcatcatttttagacacagaccatacttactttaatgaacatccgggccaacaacagt

631 tattaataaaaatgtgtttgaaaatggtgtattgatttccaaaagaaatatcagaaggaatactagatatgctatcgacataacagctgcatgtggtccaacatg

736 gctcacattcagaccttttctgtctggtcaattaatcccctattgcaaaatttctctacattaaaatctggagacactatttcatcttattgaaatcatttcaac

841 taagggcaccagtaaatgtttgtaaatattgagtaatgtgtgaaatatttagaatgtctcaagctaatgactgtatactatttaaaaagaatatagtatgacgaa

946 agaaggaaaatgacactactgccattcaaaacatacatgtatagaccttcaggtctcgaaacaataccaagttacttcatcaaccagattgtagaatattctgat

1051 tacctacccatgatgcaccaaaacttaaagatccatgtcccactgcactgtagtttttcgtaatgtatatacagtaacctccccttagtttggttttgtctatac

1156 cggtaactacagaacgcttggacaaggacacttctgttgagtagctggcagctccgtcagttagagcgacagcataacattctgaagtcgtgagttcaaatccct

1261 gccctgcccgctgaatttttactttcaaaacgctgttgttgattttggtttttttcaaatttatttttaatacttttttcataaaagtcccacaaaatattgttt

1366 tcttttcagaaacgatttatgaaagcaacatttatgccgaagaaatctgtttctgatgcgtagttactgtttctggcagtggtacggagatccttaacttcctgt

1471 gtacatttgtgtcccaaggttaacgctttatgtctacaccagcttggcgggacataaaaagacaatgatcaactgtcatggttatatatatacctatcactcagt

1576 ctggcaatgtcaaaaactatactggtatagccacatcaaagtcagatcagctatgaataccatggcctgatggcaaggttggtgtcataatatacgacattaata

1681 ttgtatggctttgccattacatattaactcattcacccctgaagacacatttgaacccctccaattcaatggttggtatagttcattataaaatttcaggggtga

1786 atgagttaaaccaaaattagaaggtatccatatcagctataaatgctttatatataacactattgctgatgtcttcattatatatatatatatatatataattat

1891 tgttacagtgaacattatatacaactgacccttacttataaatgtttttctttttattagtattatttaaacaagcatacgtacaggatacgtacactaatttga

1996 acttttaaaaaaatgctgaattttgataaattgatttagttttgactgacatttctcttggcactcagagcaaatgacaagttctttttttttccatttcggaat

2101 atcttattgtaccaatctcaagcacaaacagaattgggctacaggcatatagcctatatataattgttaagccctctccttatgacaagttctatatgaaaaccc

2206 acttaaacgttaactgaaagtagttcaatgtttattgacactggataccaattaatgacccttcctggaagatagccgttacatttattgaaaaagcataccggc

2311 acatgcacacagtacactagcaaatatttaatttaattaaatgctgaattttgatgacttaattttatatctcagatcaaaagtttaaaagaatgataaaaacaa

2416 tttaattgctttcagtttgtatacttattccataaatgtggattccaataggaaactttcaaattcaaaatgaagtaaaagataagtgcacttcatggaattcac

2521 tatttcaacttggttttcacagtacaatttaaatacaacttcagttttgcttgattatcctctattacatcaaatcaaaatggaaaaaaatctgtctttttattc

2626 atttttctggtcactgttattaaaaaagtacatgtgtagcggaaactgattaggactgcaggtgttaagatagctgcgacaatgtagcttgtgacgagcagacaa

2731 tttttttatattgcctcgtcagagctacgactcagtcctatgtcaactaatggagcaaaatcaaaagtgtcaaatctgttagatgactgtctagtgatcaagtaa

2836 agcggatgtaaaaccaactaatcaatcaatcaatcaatcaatcagatagtacagttcaacttatttaaatgtctgtctagagttcagagggtcagggtttgaatc

2941 ctggtctgtctattgcatttcccctgtccatgcttacaccagctaatacagatatttcttacaccagctaatacagacttcttacaccagctaatacagatattt

3046 cttacaccagctaattaaaagatttcttacacaagctaatacagagatttattacaccagctaatacagtcttcttacaccagctactacagatatttcttacac

3151 cagctaatacaaacttcttacaccagctaaataaaagatttcttacactagctaatacagagatttcttacaccagctaatacagagacttctaacaccagccca

3256 ttaagagatttcttacaccagctcattaagagatttcttatgccagctcattaagagatttcttacaccatttcttacaccagctagtacaaagataccttacac

3361 cagctaatgaaaagattttttacaccacctaatacagagatttcttacaccagctaatacagatatttcttacaccagctaacttggggtccaatcaaatataac

3466 aattgaagaccttagtaaagaatctcatgtgtctacagcagggtcgaagactttattgaaggagaggaaagtaatagaagtgtacttggatcaagtgacatggtt

3571 aagctcagttggtttgaacgtctgtctagagttcatgtgatatatatatatatgtaaggttctgcatgggttcctacctagtatatatataactttatttgtatg

3676 ctttttccagtgttgcagttgaagtctctgtatttggagaaaaccccatatataattgatggccaatgcaactaagataaaaactatttctaaatttgtacaatg

3781 aattagtaacaaaagttggacttaggaaaattattataatttgaaggtattgatgaacacgatataggataaataaagcatatcagaaaattaaatgcaactaaa

3886 atttgattccaatgtctaattgataaaaggaatatgtgtgcttgcagaagaaaacaagtaagattattttcaacgtatatttataatcattgtcatatatgccaa

3991 ccgtctaggagggaactccagattttcaacctgttaatttgcttaattgttcttatattctaagatctattaatttcatctaaaagaaaaacaagggataatttt

4096 gtcgagctggggggggggggggggggggggtgttatacacacatattatttatgttttaattttgagcattcatgatcactcactgaatgaatctccatcaaaaa

4201 gttggcaagtatgttatgtcaagtacctattgattagttacaatattgattagttacaatatatgaatgtctaatcaatgtgtaacattttgttccatcttgaaa

4306 aataagtggataacaatttaaaattaattttcatcaagggcgatgtatcaaaataatatacataaatatatttcaacaaataatcccaagtggcagttctaacag

4411 atcatggaagtgcagaaaaagactatcagcctgtacacagtattaaccccagggtacagtgacaccgagttaagctgtgatgattcataacatgagtcaagtgtt

4516 caacaggtgaaatcaaccctaggcctgatacggttgcacagaagtcgatagaaaccgatacgattccttgggtcattgacacctgacaacatgcagaatgttttc

4621 aatcgtaaggtttgcttacgacctaaacatctaagtatatttggatgtgatatcggtcccgattaataaataaataaacagaaacaataaccggaagtatatata

4726 gctgggagtgacactgacaagagaaaatgggcgggttggcgataacaaacaggttatttcagttttgtgcacgattggagtgtgcttaacattaatccaaattaa

4831 ttattggttaagaaaagaccagtctcaccaatccgtgtccgatttttcatccacaacatcccggtaactcttgataagagcatcttgatttttcctcatatcaag

4936 cgccatcctgctttctgcatgtgcttgtgtactacgtcaccaattcatgcgtagtgtcacggtttgcactcaccctcgggcataatatacactgcgatctcttca

5041 gaagggaaagtcttgcatactggtatgaccatcgggcattctctggtcaagaacaagacacgcttgaccgactgcggctaagcctggtaattatctagttacgtt

5146 atgttgtgtggtcattttacggtctgttatctcataatgcgctcccgctgtcagtcagctgtcatgcgaggccacggtttgtttacaaacatcgtcttgaaatat

5251 gaatggcgctgcataaataggccactgtgaacgtaaaccacctaacggactcgacatcgttttattagctattgatacggaattgatcatgtttatatcataaag

5356 attgatcataactgtgtaagaacatgtcctagaatttggaagatatagattcaataggcctagtgtgaaaattgaaatgtctgcataatattttgaagcagtttc

5461 taaaaatagaatttatgcaactaatataggtaagattataatttgattaaaagaaaatctaacaaataagttgcaccacttttagttgataactttatatatttt

5566 tttataaagggtactgtatgtgttaatttatacctttagtatagtatttatataataacagataatgataattttgaattttctaaataatccaaacttcaatgt

5671 caaacttatcgcacttaacaaattgtttggatgatctacaacttgcacaaattataccgtgcatcatgtgcattattgcctgtctgtcttaatcctggaaattat

5776 cattaaacttttattttcagacttgtaatggtaattgaattatttaaactgaactacaaatgtaacatacatattaatattaattgtcatatctgaacaagttat

5881 gttagcaccagtatgcataacaagtattcagttcgcttcaaagatttcattgtaggagtttacgctagaatcaagatgacggatcattaactttagggtccacgt

5986 ataaaatctgaatcaatggatttagtttgtgcttttattaccagctgtcactgtcagtgaattaaattgttgtaaagtatgtgtattactgttacaagtttcaac

6091 ctggtgcccttttcaatgtacacacaaaaaagtgtcatctcaaatttaaaaccgtcaatacacaaagggaacaattggcagcatgtcaccatttattgtatatat

6196 gtacacctgaccatttattgtatatatatgtagtacacctgaccatttattgacttttaaatctgataccaatgccaatgttcagtttgtaaatgtttgcttata

6301 tagctgttgagacgtacatttgattgctaatctgcagtaaatgttgtcattaacttatattacatctgatatcaaagagacgtaactaaactattctaaagagca

6406 aacattacctgataattgtgtttatatattaattgcatgcttttaataaatcgaaaatcgaaaatcagccattaatgttttcactagattggtccaggcactgta

6511 attggactgatatatcatatatacttatgtttttgtcatctatgtataccctagccttcagccgattttttcaagaatatttgtaccaaggtggctgtcctgggc

6616 actaattacagtaaatatgatagctaggcttgtacataattaaatttcctgttttcaatatatagtttaatttccagctttcaatacttttcattagaattctca

6721 tatttctggtcactacatgtatataccttgcatgcactagtaggccggagtttgattccccaattggatgtgaatatgtatagggtcacccgattctggccatgt

6826 gccagattgcaggttttttccaggttctccagtgccctgcatgatccctgcatcatgcatggtgaccggtgtccaaggagacaatgtatctgattctggccttat

6931 tggttactttatattgtataaaatatcgttaaatttatcacaggaaaacttacatgaaacattgaaaacaatgtaggcttcatattgacttacataacattgaat

7036 attgatgaggaatagatgattggttgcttgtataactgttatttgggacttgtgagatttaatagctaccgtacataaaattacattgagaagtatctgcatgag

7141 agactaccttaaccttgtctagagctttctctgaaaagatcaccactttctctatcatccttataaagcatttaactttatactcaactgttccatctttgttaa

7246 gagaccagcttagagtctaaaatgttcttgggtagtctcctaatacagatttaactgtagatatgttgaagcaatatgatactctaaaagagaaccgcccaattg

7351 gttaattgatgctggtgaaatagatttggcagttctaggggaaaactatactaatttgaagtgcatactaatggacaattaagctcacaagaacctgatactgtt

7456 gtttgtgttagcaggtgattgaaatagtataaaagcaaattaaaagaagttttcagaatcaacagattgctttgttttcaagcaaaaatatatgaaaagaaagcc

7561 tagctgcttcacagtgggcaacagttggtttcattggcctgtgctgtacatgatcactgcattagtattcctggggaaacagttggttcacgtacaggctgatat

7666 agggtttactgctgcccactaaattatttattgctattggaaaacggtcggttctaaagagtaaaaggatgacttctgtccattgcataataattttgtgaatga

7771 aaatttcatagatactttattttaaaattgaaaaaaaatatctggtacgtgtgttccaaaccattttcaatacaaagcatatcacatgtgtaatacttttcgttg

7876 tttataagagctggccttcatttcgttatcattaattaaatcattgaagttttgagatggactatatatatatagaacttttattagctaggttgaattatgcaa

7981 attattacattatccttgtcgcagagttagccacttaattgggtaggtgggtggcaggggatgtatttttacagagtcggagtaaactttatttattttacaaca

8086 attatgaaacaaattatttcaacttatattaataactcttattagctcggatgttagcatgcaaagggtctaaatgtgggaggaaaccagagtaccccggagaaa

8191 actcacatggccgggcaggtgaccccataccttttcatgttcgataggggaattgaacctcggccgccttggtgaaaggcaagttcgctttgagttgacgccacc

8296 tgggtgggagatcagtcacaggtatcgtagagcaatctttgagtgggggaggggagaggtggcttcagtcacagagccgaagatacctcagggaattagtcacag

8401 atatggagcaaccttaggatggggtgaacatcagttacagacttggagcaacagggcagtactgcatttgaacaagaaagatcgatgctctagctagccaattga

8506 ggtgattgaaataggagtttgtcacaggcaccgctgtatcaggaaaggaaaaatgtaatcattctacactgggattagaacacaggacctcaaaactctagatag

8611 actcactgacagagatccagtccagactcaaaactctagatagactccctgacagagatccagtccagactcaaacctcgagatagactcacggacagagatcca

8716 gtccagactcaaaactctagatagactcactgacatagatccagtccagactcaaaactctagacagactcactgacagagatccagtccacactcaaaacccta

8821 gatagactcactgacagagatccaatccagactcaaaactctagatagactcttaccaattaagctacctggtcacttagatagactctaaccttaggatggggt

8926 gaacatcagttacagacttggagcaacagggcagtactgcatttgaacaagaaagatcgatgctctagctagccaattgaggtgattgaaataggagtttgtcaa

9031 aggcaccgctgtatcaggaaaggaaaaatgtaatcattctacactgggattagaacacaggacctcaaacctctagatagactcactgacagagatacagtccag

9136 actcaaaactctagatagactctctgacagagatccagtccagactcaaacctcgagatagactcacggacagagatccagtccagactcaaaactctagattga

9241 ctcactgacagagatccagtccacactcaaaaccctagatagactcactgacagagatccaatccagactcaaaactctagatagactctaaccaattaagctac

9346 ctagtcacttagatagactctaaccaattaagctacctggtcacttagatagactctaaccttaggatggggtgaacatcagttacagacttggagcaacagggc

9451 agtactgcatttgaacaagaaagatcgatgctctagctagccaattgaggtgattgaaataggagtttgtcaaaggcaccgctgtatcaggaaaggaaaaatgta

9556 atcattctacactgggattagatcacaggacctcaaaactctagatagactcactgacatagatccagtccagactcaaaactctagctagactcactgacagag

9661 atccagtcgagactcaaaactctagatagactcactgacagagatccaatccagactcaaaactctagatagactcactgacatagatccagtccagactcaaaa

9766 ctctagctagactcactgacagagatccagtcgagactcaaaactctagatagactcactgacagagatccaatccagactcaaaactctagatagactctaacc

9871 aattaagctacctggtcacttagatatactctaaccaattaagctacctggtcactgacagagacccagtccagactcaaaactctagatagactctaacccatt

9976 aagctacctggtcacttagatatactctaaccaattaagctacctggtcactgacagagatccagtccagacctcactactatactcctgattataaacacagct

10081 gggaatttatttttagtattttcttggttagaatagttattagataatgggcaaatcactacatacattcagctttatatatcttcctgacaacattttgcacaa

10186 acttgtcattgacaaatgtatgttaattatgtaaaaaaaatcattgtttgtatattatgtaaattcaactcatgttagcctaaattcttctcgcatgactgtatt

10291 taaaagaatttcattattcttatgagacagtttaatttgtacaagtgatgtatatttcacctgccttgtatagatgtgtatatatatggatgtacttatatacat

10396 gtttgtgtatatatgtggacaaaacgttaacactgcaagttttcctggaaaattgcctgttttttattttttttctatttatattacttgcaaatttatagccat

10501 atatgaaataaatttatgtaaatggttgtccttttcaccataatgtaagacttggcaggtgtattgtactttgtactcgatactcgtgctttacttaaggaggta

10606 caacacactagcttgctttccattgcatgatgtttccatatccccagtaatccttaattaataatgttgaatgtctgcagtaaaagttagtgcctagatctaaat

10711 cagcagttttggatcaaaagatacaattgtaagtacaaaaaagcctcttgacaatcatatgcgcgtgacattttacatacatgtatatgtaccaggtgcatgtaa

10816 ttacattatgcatgtatgtcggcttatgtaaatttgaggatgttacttttattaaaccgaccacacgtaattaatatgttttgaaaacaatagttgtgttcaatg

10921 atcagacttaaatgaataaaattgtgattaacatttacagaaagatttaatcgtaaatattataagaaatagttttctatcttggtcaggttatgtatgaagtga

11026 ttaaactgcggttgagcatgagtaaatttatcatagatggcgaagtagtttattcaatttatacatgctcaactgtaatcacttaataacctgaccaaagtagaa

11131 aatgattccttaaaaatatatatatgatacctgattttttttatggatgccataattatacatccaaaaaaaacaaaaatcgtgatacacagcagtctctcgtga

11236 atgatctactgataaccagtataacctcgctacaaacattctaaaatgtctgatctcctgtatcaatttttgtttaaaattgttgacgtgataatcatgtaaatg

11341 tgatgggatatatatgtagatcaacaaagctgttcactgtcttgttgtacactgtaccgttttttcccctgtaacggccgttaattttcatattgtcatcacaat

11446 ctgacgtacgtgtgcagctctcatgatcacatagcttgacttcagccaatcacagacaggcaaatgatatagagccgtttatggccagttaatccgcatatgtga

11551 catatttttatgcatttttgtgattgtatataatgtaatttgatacatataattgtataaaatcaatgttatacgtattggataaatattgtaattttacagtca

11656 tctgtatcagatttcactttttaatacaaaattgtatcataatgtaatacagaattactagcttccctgttacgtatatatgtatcccaaaaaacaggagcaatg

11761 tataagtaagaatcttatatcttaattagattggcaacattgaagacttgacttgattggtgatctggaaaaaaaaagtgtttttcatgaatattcataaaagtt

11866 ttacgtaaataaaaggattctgaatttgtgtcgatttcatatgagatggagatttatgaacttctagactttttttgcctcctaatgtataggatatgcacacat

11971 tatgtgtactgactattaacgtccctcaggccgggccttgttgattatctcgcataaaaatgtgctaaattacacgtttgttctcaaactggtatggaatctcca

12076 ttttagacctagttacagagctctatatttgtggcacctgtacgtaagtaatgaagtacctcgttagtttgacggtacatatatattgtagtcagtcggtctgca

12181 gctggcttacaactctaattcattataaattaaaacactgtacacaaagtgtttgtaacagtatactgttgttagaattgaattcattttgcattgacgtttttt

12286 tatacaaacttgttgtaacaagttcttcatttttgagagactttctgtatacactcggcagggcgttacttgtctagctaacttgttaatttacatatgatatca

12391 tgttctgctgagcaagcaatatattttgatttattcttttattttagacaaatataatttcaaatcaggttcaacaagtgtatttgaaaatgataatcaattcat

12496 tctttctttagacagatttttaaatcaggtttaataagtgttttttaaaatgattaaaatatatataaaggtaaagtaaggatcatatggtaattctgacttcag

12601 attaagcttaccagtaaatgctgatttagtaaaaatttatatattttttttgttggctaggattatatgggtgagtgaataacctcaatccaagcattcaattct

12706 tcttcgaccaattagacgtccacctttaacttatatgatattgtatgtcacttttacgtacattctgtgtgtatgggtgactgtttatgcgctggagagtacttc

12811 tgtgttctgttttggctatagaactgtgtttctgcttgaaaatcacagcacagatagtgttatacctaccctgaccatatgttatccgccatccagggttgccgg

12916 agttggcagtaagggtaaatatcttactctaagattaacaatgaaaatttatagaaattttgaaacaatgaaacaacatgcatgcattaatatgtcagcgtagtc

13021 tttacttttgtctttcgagtatttcttttttatggatatacccgtcttctgaggggtttacaatggtatcatcttgtacgtctgtcaacgactttgtctctgcgc

13126 atgctctgtcaatcgacaatttattcctgatcatcttccacaaagttttgaggtggttagttttggtaagaggcacagaatttaatttccgtatttcttcaatgc

13231 attataaccttgcagacttatattggttgataatgtatacatgtatggagtcagtgtaatgtatctatgaacgtaacttatatcaccagccgcttttaggtaaac

13336 cagcgggaacaactattcagaaaaaaatctatttagataaacttgtatgcatgggggacatgttcatggcatggttgttaagtgtgttgccttaagcctattgac

13441 tgccgattgcaagtcacagggcaagaaccagagttattaacgcaatggttctttcagccattgtggaattgagaaattgactttcctcaggtatcgggaaggtaa

13546 aggtatatgtagtacaatgtacagggtacagtactatagtgcacacatggatgcttcagagacaaaacagagatgtggaggtactgaggtacacaaaacagagat

13651 gtggaggtactgaggtacacaatacagagatgtggaggtactgagggacacaaaacagagatgtggaggtcctgaggttcacaaaacagagatgtggaggtacgg

13756 aggtacacaaaacagagaggttgaggtactgaggtacacaaaaaggggatttggaggtactgaggtacacaaaaaagagatgtggaggtactgaggtatacaaaa

13861 aggggatgtggaggtactgaggaacacaaaacagagatgtggagggactgaggaacacaaaacagagatgtggaggagtactgaggaacacaaaacagagatgtg

13966 gaggagtactgaggaacacaaaacagagatgtggagggactgaggtacacaaaacaaagatgaggaggtactgaggtacacaaaacatagatgtggaggtatact

14071 gaagtacataaaacagagatgtggagatactgaggtacacaaaacagagtcttggaggaactgaggtacacaaaacaaagatgaggaggtactgaggtacacaaa

14176 acaaagatgtggtggtactgaggtacacaaaacagaggtgtggaggtactgaggtacacaagacagagatgtggaggtactgaggaacataaaacagagatgtgg

14281 aggtactgcggtacacaaaacagagatgtggagttactgaggtacacaaaacagagacgtggagggactgaggtacacaaaacaaagatgaggaggtactgaggt

14386 acacaaaacagaggtgtggaggtactgaggtacacaagacagagatgtggaggtactgaggcacacaaaacagagatgtggaggtgctgaggtacacaaaacaga

14491 gatggggaggtactgaggtacacaaaacatagatgtggaggtactgaggtacaaaaaacagagatgtggaggtactgcggtacacaaaacagagatgtggaggta

14596 ctgaggtacacaaaaaggggatgtggaggtactgaggtacacaaaacagagatgtggaggtactgaggtactctaaacagtttatcatgtatactagcagtagta

14701 gcagaaggtgtcactttgcttgccaccagaatatatatacatatgacaagatgaagtttggtttatatggcagtattaaatgtcgtgttaaaatgttaaatggtt

14806 aattaagtaattaaaacataaattttcacttctcaaacatctacatgttaagggacagcataacagctgttgaccagctgtagctaaattagaacacccaactag

14911 agcttggaagttccaagttcgaatataagtttggccccatacaatgtagcatttttcctcctggtatataagtctaatgcttctgtttgttagtaggtgtggcct

15016 gtactatgtaccagcccttctatatgaatggtatatcagtgtcggatttattgcattcccatcacgtttacaagtacaggtctgtttattagtaaaattacagta

15121 attataacatgtacgtacctgagagttaccagctactagattatttgtttacgtggttccagcttggtatgaaggttacagtgtgccaggagcaatgggctgtga

15226 aaattaattcccagtgtctgtaaaatatgttacaagaatgcacagtgcacacttatgattaattacaggctgcaaccagatggtagctcttgactttcatttcac

15331 cacttaacaattaaaagtctcacgaaacattagcagctttatccacagacatggtttataatatgtatggtatcaaacaatttatttgacataaaaattgagtga

15436 tgattgtgaattactcaaacgcacgttttcactgaaaatatctatattcctaatgtatttttagcctacctgtccaaagtgcaggtcaattcatgcactagcgca

15541 atgtccattgtctgtggtcagattttcctttgaaaaactactattccagagcagttgaggagattttggctaaatttgattcggaacatccttggacaaaggaga

15646 cattgtttaaatgatataaacatcatataattaacaagtgttttctgagcatgaatacaaagatgagtaagatcatgctaccatgagaagcctagggttctggcc

15751 tcaccgctggggacttaagttccatggacatatcatggaaatgattgagtttcaaacccgtaaccatatacataatatgtacagatatataaatgaatatggatc

15856 tcaacaaaatatgctttatgaaacatctttggtctttgtttatagatgaaaggactaaacggaaccctggatcatatgaacagtgtacattccaagatgtttata

15961 gaaatgttttatgttgtagaaatttttgatgttgtacaaaggcaaaatacagctaggaacaattccgaaatggtttctatgtttactgtatagttagtattttac

16066 gctggaaatttaatttcgctaattatttgcagtcaccaatttaaagatagcacttactaaataccaagcgaatattaataccacaatattttcccatgaaaacat

16171 agttatatatacagaaataaatctgttacctgttcactgccaaataatgaagataaaacaacaattcaatgtcatattttactttattttcacaaaacatatgta

16276 aaataaatctcatcacccaagtgcaaaagtacactcctctgccaacgcgatattttcacctggccatattgcacgaaatttaacccctgcaaactggttctgatc

16381 gggacaaccatgaaaatatagccccacgaaatttaacagtatactgtatcttaaaatctctcatgtttatattcatatctttacagatatgcatacaacccaagc

1 M V N K A P S D I S T G S S Q Y E Y I K C V

16486 tgacattgaaaatccctgtcctgacattgccacctatagtgATGGTAAACAAGGCGCCGAGCGACATCAGCACCGGGAGCAGTCAGTACGAGTACATCAAGTGTG

23 V V G D S G V G K T C L V R A W A C D T K Y R L E Q L V K T H V S T V

16591 TAGTCGTCGGCGACTCGGGCGTGGGCAAGACATGCTTGGTGCGGGCCTGGGCCTGCGACACTAAGTACAGATTAGAACAGCTGGTCAAGACCCATGTATCAACGG

58 W A T D H Y R N D K E

16696 TTTGGGCTACTGACCACTACAGGAATGACAAGGAGgtcagttattagctcacctgccctaagggctagtgaacttaatatgctgtggtgtggcgtctgtcgtccg

16801 tcgtccgtcaacttttcctttaaacaacttcttttgaagaatggacaggcctaaagtcatgataatttggtgatagcttcgttggttagcgcccaataaagttta

16906 agaacaaaaatgaccttgacctactatgaaggtcacagggatcaaaagtttcaaatacttaaaacgacttcttctgtagaactgataggtatagaatcttaatat

17011 gtggctcgtagctttttggtagagagcccaacaaagtttgagaatgaaaatgaccacaacctactttcaaggtcacaggggtcaaaagtatcaaatactctaagt

17116 gacctcttctgaataatttaaaggcatagaattataatatttggctggtaactgtcacagatgtagcctgacaaagtttgctattgaaaattacctcaacctact

17221 ttcaaggtcacagtgtggtcaaatatgtcaaacacttagaccaacttctcctgaggaactgaaacgtatagaatctttatattttgctggtagcttttttggata

17326 gttcccgacaaagtttgaggatgaaaatgaccttgacttactatcaaggtcacaggggtcaaatatgtcaaacttatcaaacgacttttcctgaataattagaat

17431 gcatagaatcatattatttggatggttgcttcttggaattgagcccaacaatgtttaaatatccaggtgagcgacacaggccctctgggcctcttgttataagtg

17536 catcggtcggtcagtatctaaagttttcaaatttgatcactgtatgttttcaattgatcagtttattatacgctgtttgtaaaaaaaaaatcattacattaatgt

17641 tcaaaatcaaggataattagttattattttagtgcctaaactgtttgtgacctgaaatactcaaatttacaatgaacatgtatcataatgatttttcggattttt

69 V L D R S W C H V D D C R V S L R L W D T F G Y

17746 cgttctgtcattttattccattgtttttttcgcagGTTCTTGATAGATCCTGGTGCCATGTAGATGATTGCCGAGTCTCCCTACGATTATGGGATACCTTTGGTT

93 H D K D R G F A Y K G

17851 ACCATGACAAAGACAGAGGATTTGCCTACAAAGGgtaaacagtcataaaaaaaaatgatgaaactctgattgcctgaaactttttcaacttcacaatttcctgta

104 A D V V L L V F S V

17956 gaccattttatacttcaacgaatgtgaaagttttatttgtaatttagctagttgcatgtattatgtattttcagGGCTGATGTTGTGCTCCTGGTATTCAGTGTT

114 V K P N S L R N I L S K W Q T E I K N E C S T T P V V V A G T H A D M

18061 GTAAAGCCAAATTCTCTACGCAATATTCTTTCAAAATGGCAGACAGAAATAAAGAATGAATGTTCCACGACCCCCGTTGTCGTTGCTGGGACTCATGCAGACATG

149 R F L Y K D Q H Y R G M E K G L L Y K

18166 CGCTTCCTGTACAAGGACCAGCATTACCGGGGCATGGAGAAGGGTTTGTTGTACAAgtaagttgtcagttcatcatcaaactagggaacattttcagctctgacc

18271 tgatttggaagcaaagtggaaattatgagtgaaaaaaattcgagttcaatgtagcctcattttcacattttaactcaatggagttggaatatttgataagaggtt

18376 ggttggtttacggatttaatgcctacatgacaccaccgatcagttttgtttcactgacggaggtcggtgttctgtcagtgtgcgataaggggtacaaacccattc

18481 tctttacagcatatagtccttcaatctgattggttgtgtttgcactttaacattgatatttaagtcattttcaccgaacaaaggttgcagatgtaagtaagtaag

18586 aggtattcacctagaatttctgcccattctttcacagtttgaagaaatgtctgcttaatttctttcgtaaaagatacgtcaatttattattagcagttcctgata

18691 ttatttttaaaaataggctacgcgctggtcatacgagtaataaacgtgatgtaaagagtgacaaaagcgtttgaaggtccgaattctgtgtggccagagaatatt

18796 actccaccatgtgctcttgtttgaaataaactacacttcatgtattactatctgcatcaaataaagcttatttacaccaggaagctgaatggtactgtaacttcc

18901 ctgttgtttcgtccaatatcaaaaatctgtgatgaacaggaatgtctgggtgatgtgtgattgcatgctctgaaacttccgttgatgcattgaacttccctgtgc

19006 agtaataacaccggaattgtctaagttcagccatgtatcctctgaaaatgaatatacagtagataggtcactcagattgtggatgatacgtttttcatcaatcat

19111 agcctagtaatagtaagcgtttcccaaaatgaaatattctccgaattccttattctcataacagggtaccatgggaggagcagaacctacttaccccaacagtaa

19216 ttctgatttcatttccaacttttgtaggggttaatgtttatcctctcatgtatacttgttacagtaaagtgctttaatttcgctgtgtcaaaattttgtgttttc

19321 atacctagagcaaatttgcactacatgattcccatgtgtcagaattcatacacgacacagctgtcacagacaactactggtgatgttttcctaatttcatgtgtt

19426 tggtgttgcccgtttgacatcgattttaattaggtattttttttactgctaagatgttaatagagcgataattccttttaactcaaacctaacatgttacctgtc

19531 cccctgactgcctgcagtattagcatgcaattgggtgaaatgtctctatgattattgacagcttacttaatttacttgattgagcccactgtgccttagtgagct

19636 gttttcagcggttaagagtcgagacaccagtcgtttacactctttgcttgacaaattaaagaaaacagacgttttctaaatctcacaaaataaggttacaattcc

19741 tgtttgtgttcaaaaccaacttggcctttataaatcaatactaaactgatgaatcgtattcctaacacaagtataatacttgtacgacccgcgtgtgatagtcga

19846 ccattgtgagcatattctgtacagttgtaaagataaacttgctactttgtagaacagtcttattggcatagacatatacattagatttttaaataagaagctgtg

168 A I D K G D I I

19951 tgtttgtatgaagaaacattaaagaattttacactaaaatgggaactgtacgttaccatggaaacaatgtgtgttttcagGGCGATAGATAAAGGTGACATCATT

176 T P D Q G R E V A R M I G A P Y Y E T S I L F N Y G I E D V F F N V V

20056 ACGCCCGACCAAGGACGTGAGGTGGCACGGATGATTGGTGCTCCGTACTACGAGACCAGCATCCTTTTCAACTACGGCATCGAGGATGTCTTCTTCAATGTAGTG

211 R A A M V E R R K I K F W N T Q L R R I Q Y P L I Q S P L Q V P Q S S

20161 AGAGCAGCCATGGTCGAGAGACGTAAAATAAAATTTTGGAACACACAGCTACGACGTATACAGTATCCTCTGATCCAATCACCACTGCAGGTTCCTCAGTCTTCA

246 F P T V T V A A S T F D L N L A Q L F K D Q N D G D I I F N V R G V R

20266 TTTCCAACAGTCACCGTGGCAGCATCAACTTTTGACCTTAACCTTGCACAGTTGTTTAAAGATCAAAATGATGGTGATATCATCTTTAATGTCCGGGGCGTACGT

281 I R A H K I C L V I A S E L F R E I L L M D I K E Q E G G S V P Q T N

20371 ATCCGCGCACACAAGATCTGTTTGGTTATTGCTTCTGAACTTTTCCGTGAAATTCTCTTGATGGACATAAAGGAGCAAGAAGGCGGTAGTGTTCCTCAGACTAAT

316 G R T E K R T N K E D E Q V L L D N E D I L E E S P I I D A N S N C S

20476 GGAAGGACGGAGAAAAGAACTAACAAAGAGGACGAACAGGTGCTTTTGGATAATGAGGATATTCTAGAAGAATCTCCCATCATTGATGCAAATTCTAATTGTTCG

351 D D R M V R H G R R D I I S T R Y L N H A A F E K V E T V R S K N S T

20581 GATGACCGTATGGTACGGCATGGCAGACGAGACATTATCAGCACAAGATATTTGAACCATGCAGCATTTGAAAAAGTTGAAACAGTACGTAGCAAGAACTCAACA

386 G E G V Q Q T V V T V T Q E I T P Q A L Q C V L E Y L Y T G R V R E E

20686 GGTGAAGGTGTTCAGCAGACAGTCGTCACGGTTACGCAGGAAATCACGCCCCAGGCATTACAGTGCGTCCTGGAGTACTTGTATACTGGTCGGGTGCGAGAGGAA

421 Y S Q L L E V Q Q A A E L L K L F P M L V A L S N L Q T Q E T Y L N L

20791 TACAGTCAGTTGTTGGAGGTACAACAAGCTGCCGAGCTTCTCAAGTTGTTCCCTATGCTGGTGGCTCTGTCTAACTTACAAACCCAGGAGACTTACCTCAACCTG

456 G L E K R F H T D R V D K L Q E L I F E Q G L L K D

20896 GGTCTGGAGAAACGATTCCACACCGACAGAGTGGATAAGCTTCAGGAGTTGATTTTTGAACAGGGGCTCCTAAAGGgtaagttgaattctccttggggcttctaa

21001 agggtaagttgaattctcctaggggctcctaaagggtaagttgaattttaataggggcttcaaaagggtaagtgatattgtaaaagtgagaaaaaaacctttctt

21106 ataggggcatctaaaggagaacttacaactagagattaacaaattaacttgcagtatgtcaaaccatttttgagcagcctctaaatggtcacattattatactat

21211 aggagtttccagaggctaatactattatgataggggcatctgttaagtgtgataatttctttgatggttagcatatatagccaatatcataagtaaataacatgt

21316 tcattaagagaagtagagaaaaaatccaataatgaatgtctagttgcaatcacaatgttaaaagttgtgctgataacaacagttccattacacctgtgttttgca

482 I H F E V D D G V V G A H K P L L I A R C E M M C A M F T D N F L E

21421 gACATCCATTTTGAGGTAGACGATGGAGTGGTAGGTGCCCATAAGCCCCTGCTCATCGCGCGCTGTGAGATGATGTGTGCCATGTTTACTGACAACTTCCTGGAG

516 A S A H V

21526 GCCTCCGCTCATGTGgtttgttttaattccttcctccaaccttcaaaatccttaatcttcacagcccttaaccctttaccataacttccaaccagccaggggctg

21631 tggtggccgagtggttagggcatctgacattctgctaccactagccctccaccactgggtcgcgggttcgaggcctaagtgggtcagttgccaggtaatgaccgc

21736 aggtcagtggtttttactccaggtactctggctttcctccacctccaaagcctggtaccaaatttgtaagccctcaaattctccaacccttaaccccaaagtcca

21841 taccacctagcatgtaaatcttaaaattttaaaaacacaactcctaaaccctcaaattcctgaaccccatatctagcttccatatcaccaaatgcaaagtcacac

21946 ttcaaaaactcaaactgcccaactgccaaacccctgaaatataaggatggacactcaagggtgattttgtggttccattgatttctagattttttcctattcaaa

22051 tggttcatatctttatacacaaagcccacatagtgtttgaacagctattgtttagcctaaaatggtatgcttatctcaaaaagtgagaaagttaaacaaatacgg

22156 aattacatataagggaggactactacttttgggaaacactatttagacttgaaaatcctttctatttatagagacatttacacttggggaacactgtatatcttt

521 I P L P D V T C E V F G V L Q E Y L Y T D K I Q S

22261 acttagactgggaattccctgtcaatttatagATTCCTTTACCTGATGTGACCTGTGAGGTATTTGGTGTCCTACAAGAGTACCTATACACAGACAAGATACAGA

546 L D S V D Q L A L I A V A N R L C L P H L I S L V E D Y V V T E L S R

22366 GTCTGGACTCTGTAGACCAGCTGGCTCTCATTGCTGTGGCCAACCGCCTCTGTCTACCCCACCTAATCTCCTTGGTAGAGGACTATGTTGTGACGGAGCTGAGCC

581 A A R C D E D I L E E V L M L I E P S Q

22471 GAGCCGCACGATGTGACGAGGACATTTTGGAGGAAGTGCTCATGTTGATAGAGCCATCTCAGgtaattacatatataaggatgaatagaatcttcccctaccttg

22576 agggtgtgacagagaaatctccaacccgagggatgattttcttgattgtcttacccgaggctttgccgagacattcgagaatatcatcccgagggagatttctgt

22681 cacaccctcaaggtagggaatgattgtttttctctcatcctgtttcactcacaaatccactgacaagtgtgattgcatgaaatataagcggtaattgcatgtttt

22786 tccagcaaatctagagttgtctcccatggtctgctactattgatgtgacgtcatactattgttatgacttagatcgcacgaatctgcaggaggcttttcccccta

22891 gtaggtgacaagctgatcttttccggattaaaagggagacacacctgtgattcagggtgggagaagtcatcaggaacgatcaaagctgaacaactgctcataccc

22996 aaagttcttttaataaccagttatagttctgaggcccctaaaatcgcagatttgttattttgatacaattcattggatctgttttacacagatttcaggtctagt

23101 ggatgtgtcaaatagtactatggtaaggatagatgtagtttgcaggatttgtttttctcagaaaattgttgcaacaaaaatctcgaaagtgaaaccttcataaaa

23206 accaatgaaatttaatttttgaccatctgagcattagtcatattgctcaatgtttgatgttctatgttcattcaaggttcatttaatgttcatcacccctcagat

23311 gactggaaccagtttactgtatgttataatttagtggaatctcacgtatgtatgctgggttatgaataattgatcgaaggtgtgtcaacttcttccttgaaacat

23416 cttcattagtaacaaattaggccagggtaataatctgtttgcctgtagcatgctaggttaaattgggctatcaagtttgttcaagtgaatgaccttgacccagat

23521 gtttcagtatttattagcatgtcaatgttcattcaaatacaattttcttttaactgttcagaaatattagtacattgttgtgttatctgagggtgattaagctag

23626 atatcgctgagccttgtgaataatttatgagaggaagatgtgaaattgaatgtagtaaatggacaaaattgtctagatatagcacttggaaaaatggattgatta

23731 aagcactttgtgtacctttgttaagccattacacaaacctaccaaagctagctgtgttttatacgaccttaaagctagagtgaggtccttggtgtctttacaatt

23836 gtgatgtcatagtcataacgtttgataatgatgaccaatactggctggatgctatttcacttaagagctcatgggccatgtgaacttataccataccacagttta

23941 gcatccctcatctctccctctgtgcagcaacatttcctttaaaacactaatggttctgaaaggctgagtggattttaaccaaatttaatctgactattgttaggc

24046 caagaggactcaacattacataaacagagggtgtggcatctccttggggcagaggaacagtgcccagatagggagatacaaataaatctttcatagacgcgttta

24151 gtgcaggtaattaaaccatgacaacatcacaacagtggttaacagtggtcaacatatcaagtctactgtttccttataaaatgtttaaaaatcattgccaaatca

24256 ttgctgtactgggcaagttcccttaagacaggtatctagttctgcttgtttaacagttaaatcaagaatgatattgaatgcttcaatgaatatgattggttacat

601 F H N A L Q L A A W C Q H Y V C I H Y R E A S K R F C R E L R

24361 tttttaccgatagTTCCACAATGCACTCCAGTTAGCTGCCTGGTGTCAGCATTACGTGTGTATCCATTACCGTGAGGCCAGTAAAAGGTTTTGCCGGGAGTTGCG

632 S L Q K E N L A L I E E N Q W P P V W

24466 TAGCCTACAGAAGGAGAACTTGGCATTAATAGAGGAGAACCAATGGCCTCCAGTGTGgtgagtatctgtaacttagggtcgacaatggtaagccgattacttaaa

24571 ttccctcgcaacaaaacaggggatataggattccacccgtacgtatgtacgtgtgttacactctttttcatcactctagcgacttcattcctcaacggaatttca

24676 tgaaacttcatacaagtgttaagtatgacaataccttgaataagttctcgtttcgggtttctatgttgaaggtcaaggtcactgttacttttttttagaaaaaac

24781 tttgccatcgctcttgcgacttcatttcctcttacatatgtcacaatgtgtgaatgcgaagggatttgtatcacttgtgatgccctgttggttataaaatttaag

24886 aactgaatagttagatatctaactgtcccataggtagccccatgtttacctgggttagtatccatctatgagctatgttaatgttgttttgcatttatgcagtag

24991 gaaagagatgctagctgacagggaaatgttaaatctggacaggttttaaaaaggggccaaaaggcaaacttgtgaaattccatttttgcaatgacccaggcgggt

25096 ctctcctttgttaatttgataaaactgtatacgggtacaaacgtcacatatatctctgctaacaagtttgacttatgtcctacagatctgcaaatcaaattttga

25201 aaataaacttttgtatattcatgtgtcattggcagtacaaactgccctgtttaaaaattactcaagtcttaaacaggatatttctttgaaatggttgtcgtttaa

651 Y V K E K E R Y D Q L M G Q K T P S L H I Q R K A Q F S

25306 taatggtctttatcatacagGTATGTTAAGGAGAAAGAACGATATGACCAGCTGATGGGACAAAAGACTCCCAGTCTTCACATCCAGCGGAAGGCCCAGTTCTCA

679 R W Q Q C K G S C F C F C R R S R V L V E E D N Y D F P M *

25411 CGCTGGCAGCAGTGCAAGGGCTCCTGTTTCTGTTTCTGTCGTCGAAGCAGGGTGTTAGTGGAAGAAGATAATTATGACTTCCCAATGTGAaaactgctgaaaggg

25516 gagataattcagggctttcagtttactttacccttaaaccttatcactagttattaaaaacacttgtcaccatcacaaaatgtttgaatactcaaatttatccac

25621 ttcgatgatattatattccgaggaaatgcaacgagccaaactgaaaccactgaaaagtcggcacttctccaggtgagctgcctgcatcattcaagagtgtcacta

25726 tcatcacttcttcgtagtatgacaaagcctgttcctcaaagacatgctgttacctaagtgagtttgattgtctcgttgttagaaaatgtctgtgatagtgctata

25831 ggtacccatctgtaatagaagtggtcaaatactaagggggttcaaaccctgtaaagacctatgtaattccagacacggaaaaaaagtccaggtgttatatttacc

25936 tggtcgattaacagaagaaatctccagaaaatagaattatagcgtgaaacctaattctgcgatattgttgcgatgtatgttaaaggagacgtataaaagcatgta

26041 atataaagtctttgatgtgtttcatgctgaagttgtgataaaacctgtctagatataccagtatcgtcgtcatcaaaattgtgaccctttttgtgatctaatcaa

26146 ttgtttgcctccccatacttgatgtcgccttttttgtattaccatgacaacagttatatcctcttgtctgtcccctgtgatatcatcaacacgcacaattttcaa

26251 gaaacatgtccccaagatcatgaaaatattaaggcttcagtccaaattaggatgttgttttattcttcaattcctcgttaaattgtcaagataaatatttattgc

26356 attgacgtttaaaatttgaacataaatctttgactgtttcatgatcctggggcgaggtcttgttaactcactcacccctgaagacacatttgaactcttccaatt

26461 caaaggttggaatagttcatcatgaaacttcaggggtgaatgagttaatgataactacaagtaaattcacatcataagtaccaggtagatattttgttactatta

26566 gttggattaatgtaatcaaactttcaatcatgtttggttcagttttcatacacgtgaaaatgttcaccacataatttgataaatcacatgaagatatgtatgaat

26671 atattttaaaatgttatttgataaatcatgttagctaatgaatcaaaggtggatctaggcccagtgccccatttaagatattttggttgtttttgggaatatgtg

26776 catttccctgtttgaaaaaatcctagatctgtgcctgtgaatcataaaatatctatagagaattaagtcatgcttgcacgacaactacagtaaacttcgttatac

26881 tgcctacagtaaaccttgttatactgcctacagtaaaccttgttatactacctacagtaaactttgttatactgcctacagtaaaccttgttatactgcctacag

26986 taaaccttgtaatactgcctacagcaaaccttgttatactgcctacagtaaaccttgttatactgcctccctttgtccagagaaaaaggaaccttgaaatatgtt

27091 ggcgaggtggcacatggattaaagggcattataacaaggttttactgtagttttaacatgataaattctagatattatattcgtcctgtagaaatatttatccac

27196 tatatatttaaaaaaaaaagaacctgtgtggacaacagatgtcataatgtatataaatgtttgtgattgctggtagtatagctggtgtggtcattatact

**PyCdc42**

1 catctattagaatctgcttcatgtgtgtgtggttatatacaggagacaagttatcattacttctttgactgtgccagatacaacaatcacagacaagttatcatt

106 acttctttgactgtgctagatacaacaatcacagacgacaagttatcattacttctttgactgtgccagatacaacaatcacagacaagttatcatcacttcttt

211 gactgtgccagatacaacaagcacagacaagttatcattacttctttaactgtgccagatacaacaatcacagacgagttatcattacttctttgactgtgcaag

316 atacaacaatcacagacaagttatgattacttctttgactgtgccagatacaacaagcacagacaagttatcattacttcttcgactgtgccagacacaacaatc

421 acagacaagttatcatcacttctttgactgtgtcagatacaacaatcacagacaagttgtcattacttctttgactgtgccagattcaacaatcacagacaggtt

526 atcattacttcttcgactgtgccagatacaacaatcacagacaggttatcattacttcttcgactgtgccagatacaacaatcacagacaagttgtcattacttc

631 tttgactgtgccagattcaacaatcacagacaggttatcattacttcttcgactgtgccagatacaacaatcacagacatgttatcattacttctttgactgtgc

736 cagatacaacaatcacagacaagttctctctgctactctactcaatgataatcattcgcattatactttacatactttactaaatggttgttaccattgtgacaa

841 tgaaaccaataaaaatatcctagagagtgtctcaaccattatcttgaactcaaaaatatttatataactagaatgatctatattaacttatattataatgtacta

946 tcaataatctaatattgtaaacatgaacagcaaacatgcatatggttataaatatcattaaacgctttctataaacacagtgtgaatatgatatactcacccatt

1051 aaatcaacatgtacttgtattttgtaatcaataacaagtagttgaataattatatcctcaatgtatacgtattataatgtgaatattaatataccatacaatatt

1156 gacatagattattcacgatttttaattatataagcctaatattcaactacatgtatctatgagtcactctgtgtggattttaaactctagtcaaggaatatgtca

1261 aaaacccacttaatactctggtgaggtcaagaagtagcacagcttgacttgttacctttcataagcagttagagttatctcccctataccatatgacttgcacat

1366 tgataaatatctgttttgtgtataagttgtatctcttttcaatttttcatttcactgataaattataatatatttatatgctgatttatacaaatgcatgtagga

1471 gagaactgatataggcatagcctgttgttcgatccaattcatacaacatcatgaataaaatttgttggacggaaagacggacggacggagtgcaaacctatagtc

1576 aaatacgttacgagatgaaataacatcttgttgtacgtacaaaaataagttgccgtcggctaagaccattacgcagctttgacgaaaagaggttaaaacgtataa

1681 aacaagaaccaaacccaaataattcaatcgttgtattgaaaattatctaaaaaaaacattaatgaaaacaatatacattgtacatgaacagaacttaaaagaaag

1786 aaaaaacgttaggctgttctgtatccgttatgcaagttctcgtatgtttttattcaatcaatagtgacctaaattccaaagtttagatcagcacaagcgaaaagc

1891 agagcaacttttctgttgaatattttggattgtgtcgagctttatttggcttcaccttcgccatgttctggaaaggagagagttgaggaatgtggtgttattttt

1996 cgtttttcattttggacaaaataaaatgacatacaataaatacataaaagaacaattaattttagattttagaaaagtattcccatactacatgaacgattaata

2101 agcccctcaatcggtctcaattttaagtatgtctgttcagatttaccagcaatggtaaggtaagatcagatcagatcagaattattttaagtcggtgggcatttt

2206 aaaaacaaatctcttaagctctatagagcttttgactgacatacatgttacatataaagacattcacacatgtacaagttagactagtccgaattattctgacgt

2311 tttatcaacttttttcatatcggtctgtcgtcgattaaccgacgacagaccaatatgaaaaaagtcgataaaatgtaagaataattcggactaaaattagacata

2416 ttgacacttagacaatgcaaatattatacaggatacaacacttcagatttcgagcttttgtcagtatagacacatttgacgcattgtagttagaacaatacaact

2521 agcatattttaaaacctatcagatctcactacgataaagttaataaatttcaaaattcatatcgggataggatggcaacctgctccgtacaccatctggatcaat

2626 acttttctgtagttctgaaccttattacctacagaaagcttacagggaaagaaagaatatctgattcaaatcacgtagttatccaacatttcttgaagttataat

2731 tcttatgtaacaagtgcgaatctgttgactagtgtcaaacctatcctgagctgcggtttaaaggataccacgtgcaatctggggggaattgcacaaaaggtttaa

2836 cgtaatataaattctttttttcagagctcatgtacatgtggtataagtacatgtaaccttaaggactgatataaaaacattgtctttaagataaagctttatttt

2941 aacttgatctgtacggacacggaattagattacggacttaaaaaaacacctgaggtgatcacaataaacatgtagactatgttaggtaatattgtaactggtatc

3046 acggaatatggaaaaaaataataggatataacatttttatccgctcgcccaaatcaatacagaagaagaaaaatattctatatatcaccacaagctactagctag

3151 cttacaaagatcagagacgtataatacataataatatgtagtatacgtctctgcaacgatgtcatctgggcgaaactcacgtttaggtctacatgcagtaacaca

3256 tataaatatagataagagaaagacaaaactaaatcctttagtttgtatataaacaactgttaacgggcctattttactgcgagaatgtacagtagagcctaagtt

3361 ataaaatcactaacaaataacgtaacgttagttttatttatagaataaaaatatatcatttcagattatagatcgaatgccccttaaaataaacggaccaagtgt

3466 atccaaaaatagaaaaccagagattactataaaatggccccgctcgtacactctcctctcattctgactaaggaatgagtgtagtgttgcttccggagcgattta

1 M R S Q Q Y P L E V R A Y L I K H P G N R E

3571tatacgactactaaaaataacttattacatcagttcgtgcATGCGCAGTCAACAATATCCGTTGGAGGTTCGAGCCTACTTGATAAAACACCCCGGAAATCGAGA

23 P E R

3676 ACCAGAAAGgtacaaataggcgaaaatgtgtcatatttaggttgcatttcactttatatgattattttggaaaccattgaagacgtttgtaataatgaacggctt

3781 ataagtgatgtaaatgcggcactgttctcaaacaaacatgagccaagccccttatcaactgacatcgagaaaatggcggacatgaaaataccgtccgtctactga

3886 cattacgttcagaatcatgttattgtgatggttcctgtgcacatatgcaggctatattgttttaattgctgtggatgtttttaaagttatataaaaataatctaa

3991 ttgaccatttctttggaagataaagtggacagtaacatgataatatcgaacttaatttatttggtgaaggcctcacccattcattttgagggtaaatgtgtacaa

4096 taggcccctcaacttcaattgataagtcggcttcataaatttgttgttagtttttagttttacattaggcaaaaaaggatccacgaaatgtaagtctctgtgaat

4201 gttcagatgaattatttactagagtgagttagctctccagacattacagtaggcctaacaaggaagactaccgaattaactgctgtggtctaaatttagcctaaa

26 P D

4306 ctgtatgaaacctaagctttacattgaatgtaatgtattccatgttattgtaatgaagtaaacaaaatctgaagttgattacattatttgtctttcagACCTGAT

28 I K G P G Q V I M Q T I K C V V V G D G A V G K T C L L I S Y T T N K

4411 ATCAAGGGCCCCGGACAAGTCATCATGCAGACAATCAAGTGTGTGGTTGTTGGTGATGGAGCAGTGGGTAAAACATGTCTGCTCATCTCCTACACAACAAACAAG

63 F P S E Y V P T

4516 TTCCCCTCCGAATATGTTCCAACTgtaagtatactgatttttctattaaattaaccatcagaaaaccatagcataaaagctgagacccccccctcctcctctaac

4621 taaacctaactcgcatttcttatcaatactcaatagcagcagaaatagtaatgaaaaaagtatttgtaatctgaagtagtaaaccaatcaatgaataattacata

4726 atcagcctctaattttatgagactgaataccaaatatgacaacactatttccacattaccatcaatcatataataaaaacttaatatttactttcaatgcataca

4831 ttgtgtacatttattatttgacacttgagttcagtaataacgcaattacgtcatatatcaatttgtgatcaaaatcgaaaagggcaaaccaataaattattgatt

4936 attgttgatcatcagcccttcactaaatgtactgtacacgtgtaagtttcgagtgtacttgctgcagagttgattgtaggatttagtgtgattgatgttatgatg

5041 tatatcactgtgcatatcagattcacatcactatcagcttatcatgattatgttccctccttcctcttgaatctgtcttgaaagttaaaaatgagatgtgtcttg

5146 tatgttgcattgtgtattgtatccagttgtggatccagagtgggttttgggcttcaggaaaccccccaccatgagaattttctttaaaaatcatgtattttcaca

5251 attttccccacttggaaacccccctggatatttttgagagacaaaaacccagatataggtctctggatctgccactggtatgtaagactgtcatctaaagaatct

5356 gaacgtgcattctttgaggtgtttgggcaagatgttgaataaacaatgcaaggagaaggaccatagcgaccaaacaggttccgtgagctactggaattatgttgt

5461 tttcattcatctgatatcgatatgcaggatcctcgatatgcaggagttacgctcactttcgctctctgcacccctggaatatgtcatagcaaaattgaaggctca

5566 atgtgcgccaccctgttgatgagacaagaagactagtttcatcctttgatgtacatcaaaaagaatctcgtggtcacgtaacagtaaaaattgacaggctttgaa

5671 gtcaggcgtctcttctttgtaatcgtcaaaggacaggtatgggcctagcaattggtcaggtgtttttttacccgagaccaatcaaacagctatctttgtgttttc

5776 aatttttctatgacatatatatatatatatatattccaggggtgtagagagcgaaagtgaacggaacccctggatatcgaggatgctgaatcgagtacttctctt

5881 tagatgcactgtgataagaaacctgttatctatgttagcatgttccctgttaaatcaagagtatttgacctttcaatgtagtttttgtggggtgtgaatttggag

71 V F D N Y A V

5986 cactgcatagaatcaaagaatttctaatggaattcttttcaaatcttgttttctcttcctttcaatgtgttttcccctttacagGTTTTTGATAACTATGCAGTG

78 T V M I G G E P Y T L G L F D T A G Q E D Y D R L R P L S Y P Q T D V

6091 ACAGTAATGATTGGTGGGGAACCCTACACCCTGGGTCTCTTTGATACAGCTGGACAAGAGGATTACGACAGACTAAGACCCCTGAGTTACCCACAAACAGATGTG

113 F L V C F S V V S P S S F E N V K E K

6196 TTTCTAGTGTGCTTTTCTGTAGTTTCTCCTTCATCATTTGAAAATGTGAAAGAAAAGgtaatgatatgtcatagtgcatgtatttgacacgtacagtatattata

6301 tttaattttgtggacaactcaacgtagtagaagaacagacttcaattgttatgtcaatctttttcattaagaaaagcaccgactacacaataaaatgtattatga

6406 ttagaaactgcatacacagttaaattgccctagactggataatggttttgataatttcaaaacattttaaatgcttgtacattatacctgatattgagaaataat

6511 acacatttattatatgttttgattctgaatattaatatgaattgaaagatatattggtcagattagtgaatggtttatatgacacacaggtctttggaaataaag

6616 ttatggaatatgagcaagaaatatatgtatatattcaaataacagaacatcatttttttacatctttgaaattttaagattctaccttagctgtgaagtatcact

6721 gtttaaactttatgaagtttgcttttatactgatatatatatatatatgtactctattggaatataagtacttgttgatattgaacatgtataatatatttattg

6826 gtaaattgagtttgtatgcctgaaaaagttatggtaactatttactatacaaatgaagataactcttagctgtaaattgacatgaggggaagcaaagaatgttgt

6931 ttttctcctcaaacaaaaatatgaataaatgtaggatattacaacctttcatgcattgtagtgaaaattaagtccgtaatctgttatatattacttatctctttc

132 W V P E I T H H C Q K T P F L L V G T Q L D L R D D A T T I E K L

7036 attcacagTGGGTTCCCGAAATAACACATCATTGTCAGAAAACTCCATTCTTATTGGTTGGAACACAATTAGATTTAAGAGATGATGCCACAACTATAGAAAAAT

165 A K N K Q K P I T I E Q G E K L S R E L K A V K Y V E C S A L T Q

7141 TAGCCAAGAATAAACAGAAGCCTATTACAATTGAACAAGGAGAGAAACTGTCCCGAGAACTAAAAGCTGTGAAATACGTGGAGTGTTCTGCCTTAACACAGgtga

7246 gaaatagtttagctgacaatatgaaaagaatctgatagatgtgtcaaattcttctgcgtgttcaacacttatgtcagacttgaggagtgcaagttttcgacagaa

7351 attacgaatctaagtatcggacagtcacattgatatatttatattaataatgagtattattttattaaacttttgtcacctttgaatcaactgaagtaccaacta

7456 agagagatctatgtcaatttggacgtttgggacctagggggtttattatgtcccaactctcaaactagaatgttagacataaattgtttgtgcaacaccatttaa

7561 tttgaataaagaaatgtaggatggcaacattaaaatcttcagtaataacttaaaagaagtgtgtagtaaagatatcctactgggggtttgatgacttgatgttag

7666 cctagttattaggaacttttaatttagacaacaacataattgatctcagtttcaagtatgtaatgaggtgttaaaggttgtaatccttgccatatgttggtaagt

7771 acacatttggtttgtatcgcatcatgatcaacctttgagagggttttatttaatcatatagatagttgcttaagttatgtcagagtaagcttttattcaaattat

7876 attttcaattgatgtgaaatatagtaaaaacgaagagcagatagatataaaataatacactgaggtcaagaatatctctggttttaaaacaaacattcatgtttg

198 K G L K N V F D E A I L A A L E P P E P P K K K K C V L L *

7981 actattacagAAAGGACTAAAGAATGTGTTTGATGAGGCCATTCTAGCAGCACTGGAGCCCCCAGAGCCACCCAAGAAGAAGAAGTGTGTGCTGTTATAAatata

8086 atgtatggcaaagtaatgaattacactcaaaactttgaactgacaaaagcaactgcaagagctgggagcgacaggaaaagcggacagaacaagcaacatgtctaa

8191 gcatagttataaaacagtcaaattgtttaacggctcaatctgtggccaaagaccagctgttcttcacatgtgatacccctatgttctttgcaatcactactggta

8296 gaacattgaaatttaataattaaagaaaaaaatgtaagtttctatttgatatttgtttttgaaaatacgaaaaatatgcgaaaagatattaatataaacaccagt

8401 gccccaggattaaagttctctatataccataagtcattaaaaacatcatcacataaaacacagatttcactcatctgaaccgttagctctatatgtgtgaaccct

8506 ttggttgaaatctgtcgctgctgtgttactatatactaaaagaatttacattcttattaatagaatgccctatatataattatattctcataaagaaaatcctgt

8611 ttgtaaacacagcaggccattaattataagatgggtaaagagttatgtggaaatgtatttttaaacaagataactattaaggtttcatttaatatcagaatttac

8716 ctggttttgatttttgtctggaaatcactatatatctatatatgcatgttgaatacaggtatcgcatattaaaagtgtagaaatgcatgtgtgttgaaaaactgt

8821 gctgaaaatatttagaaaaagtattcagtgaccttttacctctgaacattttcctttagtctctgcaaatgctgtctctttgttgtcgtgacagtacatgtatac

8926 aatcttcccattgtttgtatgattttacgtatatgattcagatttgcctgcagataaaacaagtgctattacatgaggacagacacattagtcagtatatctcca

9031 cattgtctaggatttgtctaggggcctgtttgtttatgcagaacaactggtttgtctgcttttaaatgtaacttcttctggaatatgcaggccttgcaaggcttt

9136 gtcgaggctcgcctgagagcagcatcagattactaggtccgaccttatttcatgaataacaccccatgtccaagctgttttaccgtttgcggtaaacgaaagcgg

9241 catagtattcttaggatctgattatagtctaaatatgaaaaaagttttcccattcaaatttctttgaaatgttactattcaaaatgcaacactttaccgtaattt

9346 aaatactgaataattccatttttctttttcttttttgaagtagtatgataattattcttgtggcgatttttgtgaatggaaggagccacactatcaaaacaaaat

9451 aagtgtgttgccaaaatgataggaaaaagtctgtctttgaccatatcatgttatttgtaagactttcctggtcatgtggagacgagtcttgttaaactgccatat

9556 gctgcatgtgtgtcgaccaaagcttcctgtatgtgatgtgttgtgatcgtatgttgaaagaagtgtgcaaggagaaagtgtgtccttaatatcattatatattga

9661 ggatgtattactatgttaaaaatataacccacataacaacgtcaagtttgcatttaactttacactaaaataggaaacaattttgtgcccacatacaaaaccaaa

9766 aggtaaattgtatttcatctctcaaattttccgtgagcatttattttaaatgcttggtaaagatgggttttcctcatccacaccatcggcaattttcattaagtc

9871 aaaatcttttgaatttaaaatctgttgcaccagaagtaagatgaagcatacttgttgtgtatgaaaggattacgattgtgtttttttttactagcttgaatatga

9976 tattgtcaaaaagttttgatcatacttaattgtattaccatttataaggaaacaatcacttctttttcataggagaaaacaatattttacatattttttatgaat

10081 acttttgaccgtaaacatagagggaacatgaaagttcatgtatcataattagaagaagaaaaagaaaatatacatgtatatacctatctatctttgtttggtgtt

10186 ttctgacatttctctgccgcagttattgttttattaacaggctttgggtgcaataatttaaaaaaaataacccgtttgaattaataataccagtaagtgctaaac

10291 ttatgttaaacaatactttcatttagaaataacaaattgtgcatatgatacagctagagaaattctgtgtgtttatattaattattttagtagtacacttattca

10396 aaaataaatatcaagcatataattatgccatgttataaaattgttaagtgatctgtaaaaagtaagtatgagatatatgtatcacaaaatattgcgagttgttaa

10501 atacaaaatggaattttggacatgagttttaaatctgaatattctgaaatatcaaagaatttgtcaaaaaacaccccaatataagtacatatgtaggactaaaag

10606 ctgtataattatatgcctttgtaaatatatcattgtatttgtacacacttcatttgtatagacttataatagtattctactagaggttaaaattagatttatttt

10711 tctttctgaaaactttcaccttctgttgaaattaccactctttcactcttaatttcaacatttggataatgtcatatactttgaaaagttaattttggattgtga

10816 aagactctagaattgttacctgtaaattgttataacatgaaagaacagtggtttggtctgcacctgaacaggactgaagtctacttggaacatacacagaatatt

10921 ctttctgttgttgcttaaaagtttaggaaatttatttctactgcccaatatacactgtactattgtgttaagtgctttctttatagatctgctacattggacaaa

11026 atcgttcaccaaataatgatccttgttttggtgtgcaagaaatgagtaaacaaaacgcagtaaaacttttaaaaaattggcttggtccaattatccatcacttat

11131 ttggtcatcttcattttgttcccaaaacggtattatatacatacatatattgttcacttagcttggagtctattgtatatatttcatggttagataatattatta

11236 tgtgtatttagaaagcgacatattgtatatacttttctctgtcagttgctgaattgtaactttaatttaatgttactgcaataccgtttcgtctttgtatacaac

11341 agatgatgcagaaataaagacttggaagagagttctgacaaactctcccagggaaaacatgcacacatagcatgtatctcaaaatcaatatcagcttgttcatga

11446 tttgacaacttgcttatttatgttcaatggaaagttaattttaattgtgccaaacttgtccagtgtttctgaatttttctttctcaatcttttgctttaagaaac

11551 agttcatgtttctgttagatgcacttgcacctctttttctgttaaatatttctgccaagtcattgtttggttccaagtttttagtgacgtttgggtaaatatttt

11656 ccaatttgtctccagtaatcctttatcttttaccgtgattgactttcaaatgcttaagaaaaataagatttcttggaagctggcgtttaaattatttttcatggc

11761 agccgaaggaatctaagtactagcgttgttttcctctcttttccatttgaaagaaataagaatacaccaagaacgtgaagcattgtgaacattgtactcctacaa

11866 aaatgtatagaaaaaagtctggttatctgacaattaaaattgacattattaatggaaacaattgtcttttgtattttaaactgaccggagaccgagattaatgtg

11971 aactacgacatcatcctttgtccgtcgctgtgtgtggtttgcctttcaatcttcatattaaggttgccagagcctcacttgcgaatctttttgtaaaattttgtc

12076 cattgtataaaatcatccttgtgaaaaataatttatcatggagaaacaaggtttctctgcaacaacaagtgtattaacatccttatgaagtggtatcgttgggat

12181 taatatcaagtgcccaatttttttattagctcacctgcccgaagggcaagtgagcttatgttatgatgcgtcgtccgtctgtccggcgtcaactttttgctttaa

12286 acaacttcttctcaataaccaaatggcccggggtactgatattgggcctgtagcatgctgggatgaaggactaccatgttagttcaaatgaatgaccttggccca

12391 ctttcaaggtcactggtgtcagatggctttaaaatcttcaaacaacttcttctcaataaccaaatggcccggggtactgatattgggcctgtcgcatactgggat

12496 gaagggttaccaagtttgttcaatcaataaccttaacccactttcaaggtcaaaggggtcaaatatgttaaaatcttcaaacaacttgttctcaataaccaaaag

12601 tcctaggaagatgatatttggccagtagcatgctgggatgaaggactaccatgttagttcaaatgaatgaccttggccccctttcaaggtcactggtgtcagatg

12706 tctataaaatcttcaaacaacttgttctcaataaccaaaaggcccagggtactgatattaggcctgtagcatgctgggttgaagggctaccaagtttggtcaatg

12811 aataaccttaaccctctttcaaggtcagaggggtcagatgtcttacatcttcaaacatcttcttctcaataaccaaaatgcccagggtactgatattgggcctgt

12916 cgcatactgggatgaagggctaccaagtttgttcaatgaataaccttaacccactttcaaggtcataggggtcagatgtcttacatcttcaaacaacttcttctc

13021 aataaccaaaaggcccagtgtactgatattgggccagtagcatgctgggatcaaaggctaccaagttggttaaaatgaatgaccttgtccaactttcaaggtcat

13126 tggggtcagatgtctttaaaatcttcaaacaacttgttctcaataaccaaaaggtccagggtactgatattaggcctgtagcatgctggg

**PyMig**

1 ctgatgaaaatggtatatgatatggctaaaaaggatgaaaatagactatatacaatcgatatatatatacatgttacagataatgtgggagaacacaggtaaaaa

106 acaggtaaaatatacatgaaacagactataactgctatcacaacattactgcctttatgtgttttttaaaaagtgggagaaaggagaacacaccatttaattgta

211 ctttgctagatatgacaatgtatttaattgtactttgctagatattactatgtatttaattgtactttgttagatataagaatgtatttaattgtgctttgctag

316 atattacaatgtatttaatttactttgctagatattacaatgtatttaattgtactttgttagatattacaatgtatttaattgtgctttgctagatattacaat

421 gtatttaattgtaatacggtatgttgtaatggtatatgttgaccggataagttgaccggtactgtgttttgatttagtcgtgattaaagggtacaatggcgggag

526 cgtagtggaagttgtcgctgagttgacttacttcaagttacttgcgtaaagaagtcatgtctatctttaagtataactgaataatataattgttttattattttt

631 aatatttattaatattcatatctaaatataaatcaaattgtcactatggccctaagacactatgtacatgatgacttggaaaatacttacatcatacaacccgga

736 gtaataatcagacattgtatatatatacgaacagtaacaattatcatgtaataatctcataactacagtgtataatttcatgtttttcggcaatggaatgttgag

841 acatatcattgccttatctatataacagcatatctgattgcttatattgatcacgtgacggaaataaaacatcatgtgatctcacgcgctcgcgtttccctagtc

946 tcgttcaaccagacgcttgatcacgcgtaattcaagagactctctgttcggcgtagcttgcgcagacaacaagagtctggttgaacgagactagcgtttcccggt

1051 catattttacttagaaactctctagaaaaattgccaatggccttacatctgagctccgaatgactgaggcagaaatggcatgatcatcttcactgttatagtatc

1156 gttttctttatttttgtattaagaatatttgtcgcgacagaggtatttaacacgactctttgtttctgctagattatatgagtacttatagttaccagagacgta

1261 tactttttttttttacaattctttgttattacatggaattagatgctatctatataagcccgtggttattatgacatcacagctgtaagatacccagacccaccc

1366 gcattttattcaatatttaattgttgtggactagaattaaaacaagatacaagaaactttatttaacgtcgcatacaggtaaacaaggaacattagctcttgaga

1471 gctattattgcggcgaacatatgtaaaaataacaataacatattatattacaagtaaatggagataatcacatattatttataataaaacactaataatcatcac

1576 agaaaaagaaagtatacatatcccaggtttctagtatttactaattaacattgtttgaaattctgtgatgcattgtctataatatcaaatgatacaataacagct

1681 gactgaatcagtgttgttttatttttattgttatgcagcatttcaaaggagaaaggctaaagatgtattcagactttgggctatggggcccattgtgtagtatat

1786 gatatacataaaacaatctatataaagaatgtacagtgatgatgtacattatttacaatataattaactaagggtctgatcgcttcaaagaaaagtcccctaacg

1891 taggctggttactattttcgacacgtaacaatgaaactgaaaagccttaagaaatgttatcaattcacggtaaatccaacgttaaaaagatctataacttacata

1996 taactccatatagctaaacctgcttctgttaattttgatccgcttctgttaatgtttatgacagtgtttggaaataactgatttttttttttttttgcaaggaac

2101 cggccttgtaaatgaaaatagtatttgcttactaatgaggtcacacttttttatatgagatatctattcctcgcctttgagattcttagttttaaaaaatgcatc

2206 cttagtcgtatacgtaggcctagatagatattttatctggacgcatcagcagccgcaacaccggaagttactttgggatccgcagaagccggcgaagcaaataga

2311 tcaatttgattggacgctaaaaatagctagcattgatgaaataaaatggcagctggaaggtttggcttctgacacagtggaatcttatgaaaatcaaccatgtgc

2416 tattccatgatgctttaatatgaatttgaaagtgaccaacattcattgttttaatcaggtaagtgaaaatcctttcgtatatgtggggaggaagataagtaacca

2521 ccccgaagggttaacaaggaggcgatggtttggtggaccgacttggatgagctggctgatacagtgtgcgagtcatcatgtcggaatggagtttaagaagcaaag

2626 gaaatgacatcagtgtcaatatacgtatcaagcattaggccagactcgataatgagtcatgtccctcttaatttttacgatagaacgtgtagcctcttttgtctc

2731 cgaggcggtgttgataagtctacacaaagaaacgcttgttgaccagactgtttttacgttggagaccagtgtcacatatcacaatttacatgttcagaatatttg

2836 atgaatttgtgttgtttccggtcgtttaagtgccggtaaacaccattagcttcttgtttatgtggcgacaatgtctgacacatgcgaaacgcacacacgagtgtt

2941 gtacatgtgtacgatacactgtatgcgtgactcatatggtattcaccttgaccagggtctccgtttcctcacatgttgattaatgaacatgtaactcgacccatg

3046 gtctgtgatgatttcgggtagctgggtatcactattactggtgtcctaacataacaatcaatatgtgatgtacactagtagtaaatatacagttactgttaccgt

3151 tgtaacttgtagtttatacatttggttttgtccaagtttttaaatctcttcaatgatacaagaacctttattttttttgtcgtatatatgtaacatgaaacatta

3256 gctctgaagagctattcttacgatagaaaattatagggagaattttaaacatttaaatgatagaattcattaaatatatatatgggcggattggtcgagtggtta

3361 acacttcatccttcccgaggttgtcgtgcgcatggattattcagagaaagtatttttctcctgcccacacaactgtcctgtagcattcacttgcaatgatgtggc

3466 actaaaaatgattcccttggaatcacagtcccactacaaacagaacacaatacatctccagcatacacacatagggagatattagccaggtggcataggattagt

3571 agttagcgcctgataatataaggcagctcatgcgaattcctcataaattaattgtattgattaatgtatatatgctaattgtattaattatatatctatgtgctg

3676 taaagcgctttagagtggcttattctagctagatagggcgcttactgatatgaatatctattatatgctaccatcattctaacttagatcaacgactgatgaaat

3781 ttacaagttaatgtaatgctgggaataggtctgattttgaagatcgattatcggacattcaagatcgatcagtaatcgattatcggttgaaataaatgccaaata

3886 gaatacataactacacgtacatgttattttccaaaaaacttgatctaagagacgttcatatgatgagaaaaggtacttcagaaattgacaatgcccagtagatta

3991 ccgtttcagaagtgaacatagggtaccttttccaatctcctgcagacgagtcatgatgatagcgatcgtaacacaaacaaatcaggttatgcattttgggttaag

4096 tacttgttgcgacatttatttttgttaatcaagttaacccatttgaaatggagtcaaattgagttggtattgtgaagtttatgtcccaccatgcatcccttcact

4201 aacaaaaaaagtgttttcattccgatggccgcaatttgccgtacttccggatttcgacagtaataaatttagctgacctacaaaacactgcactgaccttgttaa

4306 cgtaatgacgctatagctaccacgcttgaatgctggcatgattcaagcaagaatcaaccagaacgggtgaaagggacattttattttggatttaacttgaaaagc

4411 aactgtatgtatattaaatataagaatttcatgataaacttacaaaaaacgatcttcttggtgactaagtttcaaaatcgatctttcatgatataatgtgctaaa

4516 tcgattatcgaatcgacctcatgtcaaactcgatttaatctatgtttccggttattggtacagatactagtgctgggaatcggtcaaatattcaagatcgataat

4621 cggaacaccatataatcgatcgataatcgattttcggtttaaatgacctactggtgtaacatagcggccggctgatttattgtcttatgtaattgtgcggactga

4726 tgagaatatactcgttttcggaaatctagacggaagaaaggattcaattactgtacatccctgttttgactcaaaacttgatgacaactagatgtacacacagtt

4831 taggtattaattacccccatttgaatgctccaatcactctaacagagcatattaataataatttatcacgaaaaacactctgttagcttcattttactagctttc

4936 aaatatggcagtttcggttgatacaaaatgcagatgtttatattataggaaacgaatacaagtgacacaacgaaaagcaattccgctgttttaccattaccattc

5041 catttttagctcacctggcacaaagtgccaagtgagcttatgccatggtgcgccgtccggtgtcaatattttactttaaacgacttcttcttaatacccaaaagg

5146 cctagggtgtcgatatttggtcagaagcaaactgggataaagggctacaaagattgttaaaattaatgaccttcacccattttcaaggtcacaggggtcaaatgt

5251 gttaaaaactttaaacatcttcttcttgataaccaaactgtccagggatatgatatttggccagtagcatgcttgggtgaagggctaccaagtttgttcaaatga

5356 atgaccttcaattattagctcacctgcccgaagggctagtgagcttatgtcgtggtgcggcgtccgtccgtctgtcctcgtccgtcaaattttccttaaaacaac

5461 ttcttctgaagaatggataggcctacagtcatgatatttggctgaaagctttgttggtaggagccctacaaagtttgagaacgaaaatgaccttgacctacttta

5566 aaggtcacaggggtcaaatatgtcaaaaacttcaaacgacttcttctggagaactaaaaggtaaagaatcttcatatttggctgatggcttcctgggatggagcg

5671 taacaaagtttgagaatgaaaatgactttgacctgctttcaaggtcacaggggtcaaatgtgtcaaacacttcaaacaacttcttctgaagaactgaaaggtata

5776 gaatctttatatttggctggtggcttcctgggatagagcctaacaaagtttgcgaatgaaaattaccttgttctactttcaaggtcacaggggtcaaatgtttca

5881 aaagtttcaaattaattcttctgaagaactgagaggtatagaatctctatatttaattgatggcttcctgggatagagcccgacaaagtttgaggaagaaaatga

5986 ccctgaccttctttcaaggtcacagaggtcaaatgtgttagacacttcaaacgtatagaatctttatatttggcttgtagcctcctaggaaagagcccaacaaag

6091 tttgaggatgaaaatgaccttacctacttttaaggtcacatgaggcagatatgtcaaacacttcaaatgacttcttctgaagaactggaagtaatagaaccttaa

6196 tattcggctggttatttcctgggaaagagcccaacaaagtttaatggatttgttcttaagaactaaaatatttttagtgcaatataatatttaatgagaatattt

6301 tgctatgactgcttaaatattcaggtgagtgatacaggccctcttgggctgggcctcttgtattcaaggtcacaggggtcaattttcttaaaaaaccttcaaaca

6406 actttttctgaataaccaaaatgcccagggtcatgatatttggccagtagcatgctgagatgaagggctacaaagtttgttcaaatgaatgaccttcacccatat

6511 tcaaggtcacaggggtcaaatatgtttaaaactttaaacaacttcttctcaataaccaaaatgcccagggtgatgatatttcgccagtagcatgctgagatgaag

6616 ggctacaaagtttgttcaaatgaatgactttcacccatattcaaggtcacaggggtcaaatatgttaaaaactttaaacaacttcttcaaaataaccaaaatgcc

6721 cagggtgatgatatttggccagtagcatgctgagataaagggctacaaagtttgttcaaatgaatgaccttcacccatattcaaggtcacagtggtcaaatatgt

6826 taaaactttaaacaaattcttctgaatacccaagaggcctagggtgatgatatttggccagaaacatgctgggatgaagggctaccaagtttgttcaaacgaatg

6931 accttcatctattttcaaggtcacaagggttcaaataagttaaaaacctttaaacaacttctactcaataaccaataggccttgtatgatgatatttggcatcta

7036 ttttcaaggtcacaggggtcaaatatgtgaaatacctttcaacagcgtcttctcaataaccaaaaggcctatagctaggatgatgatatttggccagtagcatgc

7141 tggaatcaaggtctatcaagtttgttaaaatgaatgaccttcacccattttcaaggtcacatgggtcaaatatgtttaaaacattttaacatcttcttctgaaca

7246 cccaagaggggaaaggataatgatatttggccagtaacttgctgggatgaagggctaccaagtttgttcaaatgaatgaccttcatctatttgcaaggtcacagg

7351 ggtcaaatatgttaaatacctctcaacaacttcctctcaataaccaaaaggccttggatgatgatatttggccagttacatgctgggatgaagtgctaccaagtt

7456 tgttcaaacaaatgaccttcagccattttcaaggtcatcgtggtcaaatatgtttaaaacctttaaacaacctcttctgaatacccaagaggcataggataatga

7561 tatttggccagtaacatgctggaaagattggctactaatattgttcaacaaatgaccttcatctattttcaaggttacaggggtcaaatatgttttaaaccttta

7666 aacaattttttctccataaacaaaaggctttggatgatgaaatttggccagtatcatgcttggataaagggctaccaagttagttcaatcaaatgatcttcgcca

7771 attttcaaggtcgcaggggtcaaatatatttaaaacctttaaatgacttctactgaattaccaagagccctagggttatgatgtttcgtcatttagatgctggga

7876 tgaagggctaccaaatgtttttaaatggatgaacttttgttcatattcaaggtcacaggtgtcaattatgttaaaatctttgacagacttctgaataacaaagag

7981 gcctagggtgatgatatttggcctgtagcatgctgggatgaagggctaccaagtttgttcaaacaaatgaccttcacccattttaaaggtcacaggggtgaaata

8086 tgtataaaacctttaaaactacttcttctcaataaccaagaggctgagggttataatatttggcctgtagcatgctgggatgaagcgttaccaagtttgttaaaa

8191 tgaatgaccttgactataattcaaggtcaaaatggtcaaaaatgtttaaaacctttaaacaacatcttttcaataaccaaaagtcctaggatgatgctgtttggc

8296 cagtagcatgctaggatgaaggactaccaagtttgttcaaacgaatgaccttgacatatattaaaggtcacaggggtcaaatatgtttaaaacctttaaacgact

8401 tctcaataagcaaatggctaagggtgatgatatttggccattaacattctgggattaagaactaccagtttttttcaaatgaatgaccttcgcccatattcaagg

8506 tcacaatggtcaatatatgtttaaatctttaaatgacttgttctgtataaccgaatggtccagggtaatgatattttgctagaagcatgctgggttggacgacta

8611 taaattctgttcaacctaatatccttgttcgacattcaaggtcacgggtgtcatatgtttaaatattttaataggttcttattaatcaaacaccaatattctgcc

8716 tgtagttgtattttagtccacttttaagataacaagtgtgaaattaataccaggtgagcgattcaggcccattgggtaggtcacctgagctttgacaaagtttgg

8821 tcatcttcagcagtttgtcactatttccaagtcggatttaattgagatataactatataagagttcatcacttacctggaggcaatataatgcttcccatcgtgt

8926 gtacatgtactgtacaatgtactaaaaagtcaagtggcttgcttctccaccttaacatggtgaatgcctttcctatcagtgcttacttttcatctattagctttg

9031 atttcgtagtacacttttacaaaaatctaaaaaatgttgatgccaagcttatatttacttaaggaatgcttatctagtgatgatatttgtgtataccatttgaaa

9136 tgtgaaaataaaaaaatagagaataaagtctagccagatatttcctgcctgacttgaaattccctatggtaatctctaatttcattgcacggacatgtacagtat

9241 acacacatgtatgcaaatcgtctgaattgccactttacggccaccatcaagaaattaatatgcttccgtttgttttttcagaagttgtaaattaagtggtgacaa

9346 atgatacatgtatcgtatttgtgggaatcggcgcccagagcatctgatgaagtgagataacaaagcatacatgtggaagtaaaatgctgttaaggaaacaaaaat

9451 atcaatcacattaccagggaaaatccccgaagggcaagtgagcttatgtcactgtgcggcgtatgtcgtccgtcaacttttccttttaacgacttctgaagaaga

9556 actaaatggtatagaatcttcatatttggctggtggcttcctcggatggagccagacaaagtttgagaatgaaaattgcattcccagggaaaattcccgaagggc

9661 aagttagcttatatgtcacggtgcggcgtccgtcgtccttccgtccgtctgtcgtccatcaacttttccttttaacgacttcttctaaacaatggacaggcctag

9766 agtcatgatatttgggtgacagcttcgttggttggagcctgacaaagtttgagaatgaaaatgaccttgatgtactttcaaggtcacagctgtcaattatgtcaa

9871 aaactttaaatgacttctgatgaacgaaaaggtatagaactttcatatttggctgttggcttcatgagatagcgccggacacagttggagaatgaaaatttcctt

9976 gacctactttcgaggtcacaggggtcaaatatgtcaaaaatcaaacgtcttcttctgaatacctaaatggtatagaatcttcatattgggctggtggcttccttg

10081 gatggagccggataaagtttgagaatgaaaattaccttttcgttcttcagaagtcgtttaaagtgtttgtcataaatgacagctgtgaccttgaaagtaagtcaa

10186 ggtcaatttcattctcaaactttggtgggctccatccaaggaagccaccagccaaatatgaaggttctataccttttcgtttttcagaagtcatttaaagtgttt

10291 gacataaatgacagctgtgaccttgaaagttcatcaaggtcattttcattctcaaactttgtcaggctctatctcatgaagccggacaaagttggagaatgaaaa

10396 tgaccttgatgtactttcaaggtcacagctgtcaattatgtcaaacactttaaatgacttctgaagaacgaaaaggtatagaaccttcatatttgactgttggct

10501 tcatgagatagagccggacaaagttggagaatgaaaattaccttgacctactttcaaggtcacaggggtcaaatgtgtcaaactcttcaaatgacttcttctgaa

10606 gaactaaaagggatagaatcttcatatttggctgatggcttcctgggatagagcccaacaaagtttgagaatgaaaatgaccttgatgtactttcaaggtcacag

10711 cggtcaaatatgtcaaacgcttcaaatgacttcttctgaagaattaaagggtatagaatcttcatattttgctgtaggcttccttggatggagccggataaagtt

10816 tgagaatgaaattgaccttgacttactttcaaggtcacagcggtcaaatatgtaaaaactctttaaacaaattcttctgaagaactaaaaggtatagaatcttta

10921 tatttggttgttggcttcctaagaaagatcccgacaaagtttgagaatgaaaatgaccttgaccttctttcaaggtaacaggtgacaaatatgtcaaaaacatca

11026 aacggcttcttctgaataatcgaaagcataaaattataatatttggctggtggttttctaggatagagctcacctaagttagcgaatgaaaatgacctcaaccta

11131 ccttaaaggacagcgatcaacagcattttaaaggatttgttcttaagaaccaaatggaatgaattcataagatttggctggtagcttttttggatagagctataa

11236 caaagtatgagaaggaaaatgacattgtaaggtcacagggaaaaatgtgtcaattaacttctgaagaagaaccaaaaagcatagaatcttataataatatttggc

11341 tggtagtttcaacagatggagccaacaaagtttgcgagggaaatatatttagtgcaatataatatttaatgagaaaattttgctatgactgcttaattattcagg

11446 tgagcgatacaggccctcttgggcctcttgtttttatctgaatttcaacataaagtatttatggagaagtagaatcagttgacaacttcaaaatataaagttttt

11551 aggtcacctgagctttagctcaagtgacctattgctatccgttttcgtccgtcgtcgtccgtcgtccgtcgtccgtcgtccgtcgtccgttaacattttacattt

11656 caaacttaaagtcaagttctgctgggccgattgaactgaaagttgataggaatgatcgcctcatggtcctgacaaagtgttgttatttttcgggtcgatcagaaa

11761 tccaatatggccgccacggccgccattttgaaaacctatttcaaacttaaagtcaagttctgctggaccgattgaactgaaagttggtaggaatgatcgcctcat

11866 ggtcctgacaaagtgttgttatttttcgggtcgatcagaaatccaatatggccgccacggccgccattttgaaaacctatttcaaacttaaagtcaagttctgct

11971 ggaccgattgaactgaaagttggtaggaatgatcgcctcatggtcctgacaaagtgttgttatttttcgggtcaatcagaaatacaatttggctgccatggccgc

12076 tattttgctactaagttctaataggccgatctcacttaaaattggtctaggaatgatcagctcatggtcctgacaaagttaagttaacattatactattttttac

12181 tcaaattggtacatatttcaacaacaaaaataaatactatagactcaggtgaccgttaaggcccatgggcctcttgtttacacaaatcataataacaaaactgaa

12286 ctaacatccgagttacttttgagtttcgtattttacgccaaagtttagaacatctccgacggacagccgctgccaacttccaaggcctgcccgccatagctcgct

12391 gtaacagcgtattgggcgttcttagaaacccatccgtggtgattatggaacaaatcctaaatgagatcatttgtcttgacgcatgtcatcatttttagcagcgtt

12496 tcccccctttttaggtgagcttatgccataccgcagcgtccttcatccatcgtttgttcgtccgtctgtccgtcaactttttccttaaaacgctactagtcatta

12601 acgcctgagcagattttgaccaaatttggtcagaagcatccttgggggcttaggggcggggcccaatagggaaaccttagcaaattctttaaaatccttcttctt

12706 ctgtaggaatgaaagtatttggtccatatttggtctgaagcatccttaggtaaaggggaaccaatttgtataaacagtgggtctgccccctccccccccctcggg

12811 gctgaggggcagggcccagtaggggaaacatagcaaattctttaaaatccttcttcttcttagatgaagatgaagggctaacaagtttgttcaaataaatgacct

12916 tcatccatttataaggtatcacagtggtcaatagatatttaaatctttaaatgatttgttctgtataaccaaaaggtccagggtaatgatattttgccagaagca

13021 tgctgggttggaagactaaaaactctgttcaacctaatgtccttgttcgacattcaaggtcacaggcatcatatatttaaatcttttaataggttcttatgaatc

13126 aaacactgatattctgcctgtagctgtattttagtcaacttttaagattacaagtgtgagattaataccaggtgagcgatgcaggcccattgggcctcttgtttc

13231 aatgtatgaccttgaatcatattcaaggtcacaaggagcaaatatgttcaaaatatttaaacaacctgttctgaataaccaaaaggctcaggatgttgatattta

13336 atcagtagcatgctgggatcaaggactactatgtttgttaaagtgaatgactttgacttatattcaaggtcacaggggtcaaatttgtttaaaatctttaaacga

13441 cttcttctgaataaccatgggtcccagaatgatgatactttgcaagttacatcctgagatgaaaggctataaaatgtgttcaacataatgactttgttcgacttt

13546 caatcacaggcgtgaaatgtttttaaaaaaaataacaggatcttttgaatgaatttaatcataaatactgatattttgcctttagctttattttagatcactttt

13651 caggttacaagtgtgagatttataccaggtgagcgatgcaggcccattgggccacttgttacatttggtctagcaatttcattttgcaaggtgactcttttcatg

13756 gatattttttctacatatcaattaattgatgcatcacagctgtattcgaatgaattatgaactccttttaacattcaatattaatttataataataatgatcata

13861 ataatatacaacatttgatatagcgacctatttagctaacataagccactttaaagcgctttttttacagtacatagatatacaattaatataattagcatatac

13966 attaattaattcaattttgagaaattcgtaggagctgccttatcaggcgctaactactaatcctatgccacctggctaatatcgccctatttgtgtatgcctgcg

14071 tgtgtactggaaatgtattgtgttctgttttgtaatgggaccgtgcttccaacggcatcttttatagtgccacctcacttatatgatattacctctggtatttca

14176 gaataaattaaagaagcttcaaaacatgtttagcttacctgtacctgtcctgtaaagtcacagtcgcctcttacaatcatgcagtgggatacagcaggcctatat

14281 tctttaatttccccaaaactgcacagaattaaagaaattgtgggcatcaaaatcaattggtcagtatatatatatatatacgtatgatttttaaagctgaaacat

14386 agtatatagctttaatgtatttacactaaaatcaacataccttgcaagtgccccccccccccccccccccccccccaaaaaaagaacaaaaaaacataataaaat

14491 aataaaaattaatttgactttaaaaacatattttttcctaatgaggcttgagaaaatatggtctgatcatgtgtgtgtttgagtacaacctatatagctattaga

14596 tggtggagatggcacaatctgaggttgtattgtcagattacatggtatttgcaattgtcatgtagtaagtggtaggaaaccatctgtgacccatgtctattgtta

14701 gcgagtctggtgaaaatgggttcccctttgaagagtttgagatgatgattcacagcagcctggtagagttttgcctcctcctcctccaatgactccaatacagca

14806 ccagtccttatcaaagctgtgtatttacacaggatagcatgactgtgtcagattacaaggggaagggctgtagctcagtggtaccttatggcatgtcagctgtcc

14911 ttcatacaagtttaagacaattttaatttgttaacatatatccacagtaatcatatatatgctgcagggggacagtgaacttaaaagtctccaactaaaatttat

15016 cacaatctatattattctagtgctttgtataccgagtgttttttatttaactgaaatttgaacatctacatataatgctaggctgcatcccggccgcatcccagc

15121 tgcagtaaatataaatatttacatttgcatggcattgccactatatagcctctttgaaagctgtgtcgttgatgggactttaattaccatgcatcacgagacaca

15226 attcttagcgtattaatacaacactgactggaactacaatatccaatataaataaacatgagacacaattcttagcatatttatccaataaaaattaacctgacg

15331 gcagctgaaatatcgctgggcattgtgagatttgacaatgtcattgtaaatttatgtgcaaaataaaaaataaaataaaaaacacttttattacaacgaaaacaa

15436 gaccaaggtctgcaatttcttacaaaatgcacattggttgaaatcaccaatgtccgcaatggaaacaattttaactgaatgcaatttatcagaaatattctatag

15541 acctactgacacatatgttacatacctactgatatgagtcacagcacacataaacatttcagacatttgtaacgaacctgtgctccgtcgcaagatcaatcactt

15646 caaacgctatcagtttcacgcgtcttgcatccacggttccgcatgatcagttttagacaacatgtcaaataacgagtgttgtcaggttcccatacaatcaacaaa

15751 tacattcacccctggattcttcacaaaatgagcaaagagaaaagtttgaattaacttccaaaaataactgccccgtggatggcaaggtcggatttacggtgatca

15856 ctgtatttgtagatgcgcaaaatttgttccgggggaaaatcgaaacaatttcaaatctaactttcagctcagagttctattgataaacttaaatcaccctataga

15961 catatcatatgcaattgtattgatactaaattgtggaataaaattgaaatgtttgttaaaatttcaaagtactatgtacttcgtttgccaatctgcacgcgacaa

16066 tacgctgttcaaactattgaacgaaattgtgtggactgtcgataaggcgggcatttttaatttttaaatatgaattataagttaaaaaagaacataagaggcaga

16171 gaacacgtaaatattagtactgaactgtttttatgtggtatttatgatctatttgaatgacacaggtttgcaaagctgtgtcattcaaatagatcataaatacca

16276 tacaaaaacagttcaatgcttaagtatcacatgcactggtaaagtccggtggtggtgtttgggaagttgggtgagatcagacaatgtgcagatgagccgaagcac

16381 acagataaactgaccaagttaaccgtaggtgtgaaattaaagtacattgtaggtggtcaagctttcacacaacatgatcataattaattggcagttataacgaga

16486 agcacctctagttaacatgaagcatgcatatattgtagtgatagaagtaatcacatttgcctgtcaattatatacgtgccatgatgtcatatatttctctgcact

16591 tgttaagtttgtcagattaaagaaatttcaagttgaatctgacttttgtacatgttcttcagaagtaaaaaccggtaatattatgactatatgtcagatttatgt

16696 ttccatttttagctcacctggcaccaagtgccaagtgagcttatgctgtggcgtccgtcgtccgtccgtccgtcctcgtccatcaacttttcctttaaacaactt

16801 cttttgaagaactaaaaggtttagagtcatgatatttggctgaaagcttcgttggtaggagccctacaaagtttgagaacgaaaatgaccttgacctgttttcaa

16906 ggtcacaggggtcaaatatgtcaaaaacttcaaacgacttcttctgaagaattaaaaggtatagaatcttcatatttggctgatggcttccttggatggagccca

17011 acaaagtttgagaatgaaaattaccttgaccttctttcaaggtcacaggggtcaaatgtgtcaaacacttcaaacgacttcttctgaagaactgaaaggtatata

17116 atctttatatttggctggtggcttcctgggatagagcccaacaaagtttgagaatgaaaactagtttgacctactttcaaggtcacaggggtcatatatgtcaaa

17221 agcttcaaatgacttcttctgaagaactgaaaggtatagaatctttatttttgattgatggcttccttggatagagcccgacaaagtttgagaatgaaaatgacc

17326 ttgaccttctttcaaggtcacaggagtcaaatgtgtcagacacttcaaacgacttcttctgaagaactgaaaggtatataatctctatatttggcttgtagcttc

17431 ctaggaaagagccgaacaaagtttgagaatgaaaatgaccttgaccttcttttaaggtcacatgaggcagatatgtcaaacactttaaacgacttcttctgaaga

17536 actggtagttataaaatcttaatatttggcaggtggcttcctgggaaagagccaaacaaagtttgcgaatgaaaatgaccttgacctactttcaaggtcacagcg

17641 ttcaaatatgtcaaacagttcaaacagcttcttctgaagaattaaaaggcataaaattataatatttggctggtgacttcccgggatagagctcaccaaagtttg

17746 caaatgaaaatgacctcaacctactttagaggtcacagaggtcaacaacattttaatggatttgttcttgagaactaaaaggaatggattcataagatttggctg

17851 gtagctttttggaatagagctaaccaatttgagaaggaaaatgacattgacctacttacaagatcacagggaaaaatatgtcagcaacttctgaagaagaatcaa

17956 aaagcatagaatcttataataatattttgctggtagtttcaacagatggagccaacaaagtttgcgagggaaatatttttagtgcaatataatatttaatgagaa

18061 tattttgctatgactgcttatatattcaggtgagcgatacaggccctcttgggcctcttgtcttgacacaggaattgtgaaaaaatctagcttgaaaagttcaaa

18166 taagatattatacgcctttaattctgtgccttcaaacaaaaaataaaactaataaaatccaaatcaaaagtagcctcagtgatgcaaaattgataattatttttc

18271 ctttgtttacattcaccgtaattcatgttttatgtaaaaaaaatatataaatatatttataaagatcacacatttaaaatgtaaatggcagtagaacacatttga

18376 aaggaaaaggacgaatttataacggtattgaccaacccttcaataaatcgggagcacatcacgtcgcgcaacttggccattcccgtgcacgtgtcgtcgattggc

18481 acctaatacaagccaacgtcgaagtaattctgaacggtttgctgttctaaaaactgcataactccgatgctaaataacttttctcaatcacttgaaattctaaaa

18586 cgtctaaccacgaaagtacacagtcagatacgatatagggcctatcgtttgaagttatctgaaaggccgaaaatctgcacttcaacatggtgtttcaaggcgata

18691 cttatacgttctaatcaagcatatcggacgtatttgtgcttactacggtactggaaacggtaaatgaataccatgtacaataatttagatatactgaacccgtaa

18796 gtttgttggatgcggtgtttgaacgagtttatcagattggcttacggcgccgtcaaatgtatgtgtgtctcgcttatcagactgttttctcaacgtcaaagggtc

18901 atccaattaacttgaacccactgtccattattgtacaagaattataacaccttttgccatctgaagctcgataatcgactttacaacttagacaaaagcattaac

19006 taattcgcagtcgtacagcgcgcgctgatctgtgacggccctgattatgtttacgcaatcagtgtcagccacaatacacacgtgagctcgagtagacggatcgtt

19111 ttctgcgacaaagaaacagagtttccccatttttaaaaacttataagtcaatttagtagccacatcgaatttttagtaaccaataaaaaataaaagtagccatac

19216 tgacagcgtgagccctgggtataggggtctgatgtcagataacatagggactgagaccaagtcaaacttcaatatatctacaatatgtacatgtatttctccgtt

19321 tgtacatgtacgtttgtacctaaacagcgtggatcaaggagggtgtgggcctagggcgatagcttcctgatcagggtcaaggggcaattaatcatggtagagtag

19426 agcaaagtaaaagggtgtgggtagggggaggggtttatttaatagatacatttatcactcatttataacatttttcatttgggggggggggggggggatacatgc

19531 atatctatgtgaggatgctcacaatctcttatctctagttccacatgaaaacgttatggctttgagggaagatgatgacattttcctgaaatttcaagttaacaa

19636 gtctccataatgaagtaatttttcttgaagctgctgatggggaatggtaccgcaaacaaaatcaagagaaaatgtttgctttaaagtatacgtctctgaccgttg

19741 tcagactttttatagacaacgccacaatagatgagtggcaaaactacataacattttgtttgtctgtgctcaatacatgtaagtgtaagtgctacaatatttcat

19846 attttgtcatacatgtacgtagcctggttcaagttctttattactttattactttattactttgagtcgttcgactcaccacggcagttacaaagtaacaaaatt

19951 tgtgatattaactgtgcaaaatacaaaataacattacacaagattacacacacccaacacaattacaagtgcaactatacctatttataataattaggttcctct

20056 cgaatttgcaaattttatatatatttccctaagttcgttatttcttttgtatttttcacattcaataatcctttcaattcagatatgcttggctttttaatataa

20161 taagttttcaaaacaattgctcgcaaatcagaatgacatgatcttcaacatcatcttaattacaaacattacacactcgacgattcaatgttttgatatcgccct

20266 ttttctaattttgagttatgaggagatatttttttttttatttctttcagatattctttttgtatgtgtttacgcaggtagttttgtagatcaaaattatcgtaa

20371 acatatctataaaaatttgctttcgagaatattctatttttttctgcccattcttttctaaaaatatcaataaacctttctttaattaattaataaatcttgttg

20476 ttgttaatataatatttccaaatataccctaggcaaggttaatcccagcctctgatatatactgtcccctgtgtaacttgctaaaagcgctaagtggtactaatt

20581 ggtcagctgtaaaaggtcagttatctcttttattgaaccatgtaagttcataggtacactagcccggaacggcttgactgatttttgacacctttataattacca

20686 cacacagagagggcactccgttaacaaacttactatagacagagacgtagtgtaatatatacgtctctgctatagacaacaaggaatcactctctattttcacag

20791 gaactccgtcacatgatgacaataaagttatcttcatattattttttttaatgattactgaaaaatatcatcattttcaataatctgatatgaaaagtttattac

20896 gtattagggttagtattcaaccttgctcgctgataacagaactcatgatagattatttaaaataaaatagatcagttttaaaacaaatcttctacatgtacgtga

21001 gaagttaaatatcctaataagggacattttcttttattaaattaggaattccaagttaaatattagtttaccttggttaaggcactgttgtgaaatgaaattgtg

21106 cctggggctgtcctttcctgatcgagggacagccaaagtatcttacctgtaggtttttatcttgaactgatctacctgtatacagctgtacgtgattgatagaca

21211 atgaactgtccatacaagatggcgccgaacagaggcatacacggtagtgagagctccgttgataaccgatgagtttgaacgtgcaagtgactttttaggctttga

21316 aaccttgcaatagactatcgaaagtatcaagttacagaggtattttgtgaattttgaattttatcgtggttttgaatgattgatagattgcaatcgtatcgctat

21421 atgtattgtaacgtgatcatatttagtatgtgaactttgaactttggtgaagtattgtggcctgttcttgtccgaattatttcgtcatgctctagtggttcagga

21526 acttacctgtgttaccggtgtgttacctgagcgcgtaagcccgctcgcgacgacaacgatgactagcactacttccagtatattagaaagtgtgtgtggggtcct

21631 acacggggacaccgaaacagatatagccagttctatgcggcaacttatagaggtgatgaaaaacataaatgatcgattggggcgaatagaaacacaagtacacaa

21736 gattgatcgtatagatgctgcattaacttccctaaatgcgaaggttattactattgaacatcaagttaaggaaatagtacagaaaaacattgacatagaaaaaag

21841 tacggaagctatcggggaattccatgaacagatgaaaagaaagactgaaaaaaatgagacaacatcatcaaaaacaccgggagaactccaaggtgtaataatgga

21946 aaccagagacatttcaacaaatcaatgcattatccaaaacgaaatgatgaatttaagaaaggactgtgaagaaatcaaactagaggtggaagaccaaaagtgtag

22051 gaacatgaaatacaatcttattctaacgggtttaatggaacaaaggtacgaagcaatcggagacaaagtgcatgacttttttcagcgtcaacatggaatcacgcg

22156 acatatggaattcgcgaacgtgcaccgattccagaaatacgttcatggtaaagtccgcccagttgttgtccgctttttgtattacgaggacaaacaagaggtcaa

22261 agatcatggttatatgctacgaggtacccgattcggcatcaacgaacaattcccggatgcagtcaaggaaagacggaaaaagttatatccggtcatgaggcactc

22366 acaagcgcgctggtcaccatgccaaacttgtgagagatcgtctgtacatagacggggaactttacttgccaggcagtgacacggataatgtacagaggtcggata

22471 ctcaacaaaaagtgcagcaacatcagccttctgttgatatgagtacgccagctagcggacaaatagcaaaagaggacgtaacccgtctacccccgatgggcgtga

22576 acgactttagccgcccgagtcccgcgcgcgcaccagctcgccgcagcagcccatagccagcttccatggtattgggtcgctaatagcaccagaatggcactacac

22681 gccagcgcgacaagaacgcatcaccgaattccaaccgcgaccctcgcgaggacaacatacggctactggccagaggtggaggggttcatcttcgcgaaaggacca

22786 cagtgcgcgcttcgatcacgagccacctagagggaatcacgacttgtcgtattagggttgtgtggagggactttccattatttcatggaatatatgtagatcaat

22891 cacagacaaattagatacagaagagtttgtaaatattattaccaaatatgatgttgtatgttggtatgaaacgtgggttgagaaagaggatgaagtggaattgga

22996 aggttatgacagctgtgtgtttcctagaatgcatggtaaaggcagtggaatttttattaaatcaactatcttcgattattgtagggtaatagataacattaatga

23101 caacattgtattgttaaaaatatccaataacattagaaataatgtcagtgatatatatgcatttgcttgttattttccacctgtaaatagtgttttttatcacaa

23206 atgtgaaattgatatttttgccagacttgaggaaatggtgtgtgaatataaaggattaggtaaagtgattgtactaggagatttcaatagtagaacgggtattta

23311 cgacgattatataagtaatgatattgttaatcaagggcctgcgcatattatttcaaatattgtaaattatgttaatgattctgtaccatccaaacgttccaattc

23416 tgacctgcatattaaccaatttggaagacatcttctttcactgtgtaaatctacttccttgagaattgtaaatggacgacataaagatgatcctaatggtagtat

23521 aacttttttgaatagtaggggatctagtctaatagattatgtattgactgttgatgatttctnnnnnnnnnnnnnnnnnnnnnnnnnnnnnnnnnnnnnnnnnnn

23626 nnnnnnnnnnnnnnnnnnnnnnnnnnnnnnnnnnnnnnnnnnnnnnnnnnnnnnnnnnnnnnnnnnnnnnnnnnnnnnnnnnnnnnnnnnnnnnnnnnnnnnnnn

23731 nnnnnnnnnnnnnnnnnnnnnnnnnnnnnnnnnnnnnnnnnnnnnnnnnnnnnnnnnnnnnnnnnnnnnnnnnnnnnnnnnnnnnnnnnnnnnnnnnnnnnnnnn

23836 nnnnnnnnnnnnnnnnnnnnnnnnnnnnnnnnnnnnnnnnnnnnnnnnnnnnnnnnnnnnnnnnnnnnnnnnnnnnnnnnnnnnnnnnnnnnnnnnnnnnnnnnn

23941 nnnnnnnnnnnnnnnnnnnnnnnnnnnnnnnnnnnnnnnnnnnnnnnnnnnnnnnnnnnnnnnnnnnnnnnnnnnnnnnnnnnnnnnnnnnnnnnnnnnnnnnnn

24046 nnnnnnnnnnnnnnnnnnnnnnnnnnnnnnnnnnnnnnnnnnnnnnnnnnnnnnnnnnnnnnnnnnnnnnnnnnnnnnnnnnnnnnnnnnnnnnnnnnnnnnnnn

24151 nnnnnnnnnnnnnnnnnnnnnnnnnnnnnnnnnnnnnnnnnnnnnnnnnnnnnnnnnnnnnnnnnnnnnnnnnnnnnnnnnnnnnnnnnnnnnnnnnnnnnnnnn

24256 nnnnnnnnnnnnnnnnnnnnnnnnnnnnnnnnnnnnnnnnnnnnnnnnnnnnnnnnnnnnnnnnnnnnnnnnnnnnnnnnnnnnnnnnnnnnnnnnnnnnnnnnn

24361 nnnnnnnnnnnnnnnnnnnnnnnnnnnnnnnnnnnnnnnnnnnnnnnnnnnnnnnnnnnnnnnnnnnnnnnnnnnnnnnnnnnnnnnnnnnnnnnnnnnnnnnnn

24466 nnnnnnnnnnnnnnnnnnnnnnnnnnnnnnnnnnnnnnnnnnnnnnnnnnnnnnnnnnnnnnnnnnnnnnnnnnnnnnnnnnnnnnnnnnnnnnnnnnnnnnnnn

24571 nnnnnnnnnnnnnnnnnnnnnnnnnnnnnnnnnnnnnnnnnnnnnnnnnnnnnnnnnnnnnnnnnnnnnnnnnnnnnnnnnnnnnnnnnnnnnnnnnnnnnnnnn

24676 nnnnnnnnnnnnnnnnnnnnnnnnnnnnnnnnnnnnnnnnnnnnnnnnnnnnnnnnnnnnnnnnnnnnnnnnnnnnnnnnnnnnnnnnnnnnnnnnnnnnnnnnn

24781 nnnnnnnnnnnnnnnnnnnnnnnnnnnnnnnnnnnnnnnnnnnnnnnnnnnnnnnnnnnnnnnnnnnnnnnnnnnnnnnnnnnnnnnnnnnnnnnnnnnnnnnnn

24886 nnnnnnnnnnnnnnnnnnnnnnnnnnnnnnnnnnnnnnnnnnnnnnnnnnnnnnnnnnnnnnnnnnnnnnnnnnnnnnnnnnnnnnnnnnnnnnnnnnnnnnnnn

24991 nnnnnnnnnnnnnnnnnnnnnnnnnnnnnnnnnnnnnnnnnnnnnnnnnnnnnnnnnnnnnnnnnnnnnnnnnnnnnnnnnnnnnnnnnnnnnnnnnnnnnnnnn

25096 nnnnnnnnnnnnnnnnnnnnnnnnnnnnnnnnnnnnnnnnnnnnnnnnnnnnnnnnnnnnnnnnnnnnnnnnnnnnnnnnnnnnnnnnnnnnnnnnnnnnnnnnn

25201 nnnnnnnnnnnnnnnnnnnnnnnnnnnnnnnnnnnnnnnnnnnnnnnnnnnnnnnnnnnnnnnnnnnnnnnnnnnnnnnnnnnnnnnnnnnnnnnnnnnnnnnnn

25306 nnnnnnnnnnnnnnnnnnnnnnnnnnnnnnnnnnnnnnnnnnnnnnnnnnnnnnnnnnnnnnnnnnnnnnnnnnnnnnnnnnnnnnnnnnnnnnnnnnnnnnnnn

25411 nnnnnnnnnnnnnnnnnnnnnnnnnnnnnnnnnnnnnnnnnnnnnnnnnnnnnnnnnnnnnnnnnnnnnnnnnnnnnnnnnnnnnnnnnnnnnnnnnnnnnnnnn

25516 nnnnnnnnnnnnnnnnnnnnnnnnnnnnnnnnnnnnnnnnnnnnnnnnnnnnnnnnnnnnnnnnnnnnnnnnnnnnnnnnnnnnnnnnnnnnnnnnnnnnnnnnn

25621 nnnnnnnnnnnnnnnnnnnnnnnnnnnnnnnnnnnnnnnnnnnnnnnnnnnnnnnnnnnnnnnnnnnnnnnnnnnnnnnnnnnnnnnnnnnnnnnnnnnnnnnnn

25726 nnnnnnnnnnnnnnnnnnnnnnnnnnnnnnnnnnnnnnnnnnnnnnnnnnnnnnnnnnnnnnnnnnnnnnnnnnnnnnnnnnnnnnnnnnnnnnnnnnnnnnnnn

25831 nnnnnnnnnnnnnnnnnnnnnnnnnnnnnnnnnnnnnnnnnnnnnnnnnnnnnnnnnnnnnnnnnnnnnnnnnnnnnnnnnnnnnnnnnnnnnnnnnnnnnnnnn

25936 nnnnnnnnnnnnnnnnnnnnnnnnnnnnnnnnnnnnnnnnnnnnntcatggtcctgacaaagtgttgttatttttcgggttaatcagaaatacaatttggctgcc

26041 atggccgctattttgctactaaattctaataggccgatctcacttaaaattggtctaggaatgatcagctcatggtcctgacaaagttaagttaacattatacta

26146 ttttttactcaaattggtacatatttcaacaaaaaaaaaaaatactgtagactcaggtgaccgttaaggcccatgggcctcttgtttttaagtattttagcgaat

26251 tgtcttcaacggaacctgttgagaatgaaatggttaatgagtttttaaataattatgatgttaatgtaccaaatactatgtttcaagaattagatgatcagatat

26356 ctgatagtgacattttcattttataattagcaaattattacggtaattttttctttcatggtaagctagcaagaagtaacgataacagttcatcagaaaacaaac

26461 aaggataatcttttccctaagaaaattgaaataataaattattcattcccctcgggcatttaactaataaacaaaagaagagaacttcaagaagtttcaagtttt

26566 atttttacaaataaaacttgaaacttcttgagtaaagtaaagggccaggacaggtatctaacttaattttgtgaatattatatatggttcagactgcatttgttg

26671 aaaaaaaacctgaaaattttgaatggtgattgacacccccttgaggaaatttcgaatcagaatccgctcctgggtctagatcttagaccctttctagaaccagta

26776 taattaacagttagacgcaggactaacaaattaaacttgactgtttataacacgtaactgtcgatccattcaacaggcccactctactatctcagggagctagag

26881 catcagcctctcatttaggatccagtaggaggtatagatctatagcagcaccataacatccaatttactcctgccattatagaggtcaaaggtcaaggttataat

26986 ttgggaaatgttggtgggtctctgtcaggaaagcaattactgtgttacatttatgtcaaaaacaataatgatgtctatattgtacaggacagcataatgaaactg

27091 tatggtaatatacaagtcatgatcatgaataatgatatgcaaatgagggcagcacatgaacgacctacttagttatattattgagtggaagaaaccttatagact

27196 gacaaagtcagactcatgaacttgacctacagatggatatggatacattttattgatgggtataaatatatcagaaattacttccgtttatgcatatagtatcag

27301 tcttcctgaaatatctggttgatccccagatatctgcagacaatttttgttttactctacccaccctatggagcttatgccaaagtggcacagaatcattcatcc

27406 atcttatatccccaccattaaattcttaagcattaaaaacattaattactaggcatcaaagaaacaaatacaggtcatgctacctggatttttccctggttcagt

27511 cttaccccaggtgttaaagtcccagctgggtcattacataagaacagttgtatttcaacgaaatttgaataatgtatgcttgggatcaggaaatacaatttttgt

27616 ataaaagaaatatggagagttcgagcccaaaatggggaaatatttgtaatattgtattactgttaattggaatattaaatttggccatgattgcatttgcttaaa

27721 tatcaagatgattgtgcattacaggccctgtttgcctcttgcttctttaaacaatatctttcaaaaataactaaaatactttttatatacattgtagctagggtc

27826 atgatatttggccagtacacataattataaccttgacccactttaaagttatcaaatgttgaaaattcaagatgacaatggtcatggtatatatcagctataggc

27931 aagtaacattcttgggcaaagtctgttccaatagactgacttcatccactttcaaggtcacaggggtcaaatgtattaatatctttattaaatttcttctgttta

28036 gtttcaatgtacaatgattttattgtcgaatatagacaacgtattaagcacaaatcataaaaagttatcagcattttcttccaagaacaattgtcagaaattgtg

28141 gtaaatttattggaaaagacctttgcatatttgagtatttgagcttccttatttaattatggcatgctgggaccactgtgggtcaacagttgatgtattgatttt

28246 tcctcaaaccctcataagctcctgtcatttttatactcccgtcaaagacagggacatattatgttatcacgtgagcgggtgggagggcatgcgaacgatgtccgc

28351 ttgatatctcaagtaattattaacaattaaattaacttcaaacttcacaacaagattaaataccatgaaagctcggacaagttcgataaccgccattatccgacc

28456 ataatttgtggagttatgcccctttacttgccgaaacagtgtccgctcgatatctggagtaattattaacggattaacttcaaactacacaacaagattaattac

28561 catgaaagctctgacaagttcgataaaagccattatccgaccttaatttgtggagttatgcccctttacttgccgaaacagtgtccgctcgatatctcgagtaat

28666 tattaacggattaacttcaaacttcacaacaaggttaattaccataaaagctctgacaagttcgataacagccattatccgaccttaatttgtggagttatgccc

28771 ctttacttgccgaaacagtgtccgctcgatatctcgagtaattattaacggattaacttcaaacttcacaacaaggttaattaccatgaaatctcgattaagttc

28876 aataactgccattatccgaccattatttgtggagttatgcccctttatggacttgccgaaacagtgtccgctcgatatctcgagtaattattaacggattaactt

28981 caaannnnnnnnnnnnnnnnnnnnnnnnnnnnnnnnnnnnnnnnnnnnnnnnnnnnnnnnnnnnnnnnnnnnnnnnnnnnnnnnnnnnnnnnnnnnnnnnnnnnn

29086 nnnnnnnnnnnnnnnnnnnnnnnnnnnnnnnnnnnnnnnnnnnnnnnnnnnnnnnnnnnnnnnnnnnnnnnnnnnnnnnnnnnnnnnnnnnnnnnnnnnnnnnnn

29191 nnnnnnnnnnnnnnnnnnnnnnnnnnnnnnnnnnnnnnnnnnnnnnnnnnnnnnnnnnnnnnnnnnnnnnnnnnnnnnnnnnnnnnnnnnnnnnnnnnnnnnnnn

29296 nnnnnnnnnnnnnnnnnnnnnnnntaaacaacaattattcctaaatatttttaatgaattaacttcaaaatttatgacgaggttaatttccgtagaagcttggac

29401 aagtttgataaccattattatccgaccgtaattattggagttattccccttggtttcattaatgcgatgcatgcgttccatgatgttgtattgcactcattgatc

29506 taccagttatgttgaatgatttctcttttaatatcaattactcacaaacacttcatatatttcaatcatacaaacaactgctcattcaatgtgtgatgggcatat

29611 tatataccgctcgcggtaaccttgttgatttgtctattaatatattactcccttatacagattgttttcaacagaaatatgacagaggactttattgttgtatgc

29716 ataaattattaaatttcctctttgcaagtatttgtcataggactagatatttgcaatttagttttaaataaggtaaaattaaaacttttataggataaaatatag

29821 tgttcttattgtaacaattgtatgatttagagtccggtgaagggcattgttcaaatcctgtaccggaataagcttaacttcactattatgctaagaaaactaagg

29926 aatgcagcatgaattatgcgaacaattactagcactaatattattaatcttaaaagtgttgagacaggccaaaaatcctaatctacatatatatgattaattgat

30031 ctcctttcttttacagtaatgttcaaattattgggggcgggaggggggaaacattttatttacaagctttaacttggtatgtttgttgccctagagaagaaaact

30136 ctgttgattttaaacagtcagccaaaggtcaaattaaagttgctttaactaataataacagaatttcacataaaaaatcatttgtctggatgctaaattcagaat

30241 gaatgatcacataaaactcacacttggtgtaggttgcaagaaccctgttgaatttggacacaattgttcaaaggtcaaggtcactgttaattaaaatacataaat

30346 tttcttatctgtatatacctttagaacaactgataacccttaatagctaagacttgtgtgtggttgacccatatacccagtaggaaagaggaaatacaacaaaat

30451 gtacactgttgattgtgggcatggttcatcaaatgccaaggtcactcttttgagcttaattactgaaaatggaattttgtcaaagcatattttcagaacaaatgg

30556 ttctgcaaacttcaatgttgactgccctagaggggaagaagtattctattattgttggttgtgggttgtcaaaggtcaaggtcaatactactaaaaatagaactg

30661 cctagaacctcactttgagatagaaactcatctacaattgcaataaaaacaatttcacaagcacagccccatttaatgaaaatcattattatgtttcaacaaaaa

1 M

30766 atgttcctctggggtttttcctgaggatatttaaatttgcatgtgaccacatgttaattgatgctttgcttgattccttgcagatctgacagaaatttgacggAT

2 S V I I S N H L S D S M A Q G R P I K C V V V G D G T V G K T C M L I

30871 GTCTGTGATTATTAGTAACCATTTGTCGGACAGCATGGCACAGGGCCGGCCTATCAAATGTGTAGTAGTTGGCGATGGTACTGTTGGCAAAACCTGTATGCTCAT

37 S Y T T D S F P G E Y V P T V

30976 CTCCTACACAACGGACAGCTTCCCTGGGGAATACGTCCCTACTGTgtaagtgtatcttgttgctgctccaggcacagtatgcagtatgtatgtatatacatttct

31081 aatttgtttttgacaagctttcaaaaaaaaagaaaaatggaaactacattgctgcctcataaatttaaacaatctcggaaaacttgacagaatctctgaatattg

31186 ccctagcatttcttcttgtaatgaaggcggaagtttattttaatgtgtacaattttgatagtgcagcattattttactggaattatgtagaaaaattggctagct

31291 agacatcaacaaacagtgcattcaaattgtctaagctcctaggcttgatttgccaagaaaatattaccagatgtttttggtaactgcagctattacatgaatgga

31396 tatctatgtaaacacttgcaaattgtttttccagagtaggtattttatgcagttaaaagataaaaaatctgaaaattttgctttgttattaaggcatctgatcta

31501 tgatgagtcacttgggtcactgtgattgtcttttgtctgtcaccagtaaacaaacatattttggaattctcagaaaattaatgtctgattttgatgaaaaatttg

31606 ggtaatcatcagtctgaggtggggatcagaatctagactcaagaggtgcctaacctaagagaggggtggggtgggggttgaaggtcgggccataattatataagg

31711 ggtcagagtgcaaaacctacaaatgatatctcctctgaaactaacaatttggatatatataatatatataacatgctgggatgaagggcttccaattaagtctgt

31816 tcaaatgaaagaccttgaattatattaaaggtcacagggatcaaacaggttgaaaatgtttaaacaacttcttctgaataaccaaaatggccaggatgttgatat

31921 ttagccagtagcatgcatagatgaagggttatcaagcttttttgtttggaatgcccttgatcatgacccattttcaaggtcacatttggtcaaatatgtataaac

32026 atttaaatgacctaatctcaataaacaaaaggatatagggttatgatattttgcaagttgcatcctgagatgaagggctataaaatgtgttcaacgtaatgacct

32131 tgttcgactttcaaggtcacagggctgaaatgttttaaaatcttttaacaggttcttttgaaggacttgtagtcataaatactgatattttgcctttaggttaca

32236 agtgtgagattaatactaggtgagcgatgcaggccaattgggcctcttgattaaactaggtcttgaaattgcagtcctgtgaaatcagatatttttgtggggtcc

32341 aaatgaatttgttcatggtatacatcgttcataggtacatgaatttgtggattacgagtatccaatcattaggagaattgctttatataaaattacttcatggac

32446 ctatgaatttgttaatgaagattacctaagaaatcaataaaattaaaaacctcacaaaagttagtgatttcacagtatatagatggatatttggggtcaattttc

32551 tgcctttcatttgaatactcgatattttcccaaaaccactgtttgcctccaaactgatagtgaaataagtaagccatcatcacaatttaaaagaattattgaaac

32656 tgtaaagtatgcataagtttcaggcaaacaggccctgaggaaaggtagaaaaatcagtgtttactacatatactgtgctagttcaaaccattttacatgtagaag

32761 tattgcagtaatgttatttaaatgttgttgcattattcctgatcatgcaaagatatttacatcaacacatctaataccaatctaattaaaattatatcttcacat

32866 cgctcccgtttactgatatgtcgcgttgaagatctaacaacatcccgtagagggtcacgtcagaggtcaacatgaatgcattatcgcatttataacaagctaatc

32971 aaagcgcgacataactctttcattcaaccaatcagaaagcaggacctgtagcatttaatgcagacacctcgtagcccaaaatccatttttcgactggtccagtga

33076 tagtttgaatattgcgaagcctgtcataggttaattttagtttcaggttgttaaactttttaacgacaacatcaccagaagaggaagtctctcttatttcaaagc

33181 aaaatccacaaattgtacaaacgttgtcttgtagatcgacacggcgtggtgtcatgggcaccgccattttcgatgctcatttgacacctctcggtcttttatcat

33286 agcaagtgtgttccgaaatcattacatcatcacttacagaaatgcgtaagctctcatgtgattggttaatgcaaaggtcgcctgaacatgaccctctatgggatg

33391 ttgttagatcttcaacgcgacatatattagtaaatgggagcgatgtgaagatataattttaattagattgatctaatacataacaaatttgctgtaataagatcc

33496 tctcaagttaacaaaataaagtactatatactttatattaaaaactactgttttgcaatctaacaagtctgtttcagagtggtttagaatagttttgaaactctc

33601 ccttcaaatgtgtctgataattttgttgcctcttcagcaactgccataacctatgttctgttatcttaataagcgccctcttcagcccttaaatgacttaagctg

33706 ttcatctgatgttaaacctttaacaacccagccaatatttcaagcaccctggacacttattgcggttactttatgatcatttgcaaggtaccaatacacaaatca

52 F D N

33811 tacttctatagtctatacttctaatacactaatactaaagtcactgagaaaaatctctaggtctgttatcaaataaaatctgattgtatttacagGTTTGATAAC

55 Y T A N M M V D G V P V S L G L W D T A G Q E D Y D R L R P L S Y P Q

33916 TACACAGCTAATATGATGGTGGACGGGGTCCCGGTCAGTCTCGGGCTGTGGGATACTGCTGGTCAGGAAGATTATGATAGACTTCGGCCACTTTCCTACCCACAG

34021 gtgtgtaattttatgtgtcgcccaccattgtatacctcatactgaaatagacatacacatatgctgtatataaagtttggtagagctacatgtgcagttgggtat

90 T D V F L I C F S V V S P S S Y E N V T T K W N

34126 aaaagccgcttattttctttcttcctattttccagACGGATGTATTCCTGATTTGCTTCAGTGTTGTGAGTCCCTCCTCGTACGAAAACGTGACGACGAAGTGGA

114 P E V K H H C P D A P V L L V G

34231 ACCCGGAGGTCAAACATCACTGTCCTGATGCTCCTGTACTTTTAGTCGgtaatgtgttgcagggaagacagaatggtgggaagggatataactggcaggggtgca

34336 tgtggaagcaagtgttgagaccgtaatagacatcatcggaagtaggaatagctcagctgcttgaaaagagtatgagcctctctcctggtgtttgtcacctcgcat

34441 caacatttttattgaaacaacttcttctttaaacaaaaatggtccagagtcatgtaatttggccaggaagtggtatgaattgctataaatttcctcatataaatg

34546 agcttgaccattcttcaaggtcacatatatatgggtaaaatatttttagctcagctggtccaaaggaccaccggcttatacagcatggtatcatgcagcccatca

34651 tttttagctcacctggtccgaaggaccatgttgagcttatgccaaactgaagcgtccgtcgtccgtccttcaacttttcctttaaaacgctactactcctaaacg

34756 gccaagtagattttgactaaatttgatctggagcatccttaggccaaggggattcaattttgtataaatggtgggtgtggcccccctgggggcagagaggcagga

34861 ccaaataagggaagtagaggtaaaacctttaaaatccttcttctaccgtagggattaagggattttattctaatttagtctggagcatctttgtgccaaggggac

34966 tcaattttgtatacacattgggtgtgaaccccccccccccctgggggcagaggggcaggccctttaggggaaatagaggtaaaaccttaaaaatccttcttttac

35071 catagggattaagggattttatttaaatttagtctggagcctccttgggccaaggggactcaattttgtataaacagtgagtgtggcccctcttggggcagaggg

35176 gcagccgaataggggaaatagagcaaattcttcaaaatccttcttcttctgtagaaacgacaggataatgacaggttatatattatccaaagatatgaagcaccc

35281 tttggtgaaggggaaccaacctgacccccctgggagcagagaggcgggcccaataagggatatagaggaatagctttaatattctttttcttctgttgggatgaa

35386 ggtattttgaccaaatttgaacagtagcatcctatgggaaagggaaatcaattttatatagatggtgagtctgacctccctggggccagagggtggggcccaata

35491 ggggtaatttagcaaattcattaaaattcttcttctgtaggaatgacaggattttgtacatatttggtgtaaagcattctttggtgaaggtgactatattttgta

35596 taaacgttgggcctgaccccctgggggcagaggggtggggcccaataggagaaacatagcaaattcttcaaaattcttcttctgtaggaatgacaggattttatc

35701 caaatttgggatgaagcacccttgggtgaaggggaacctattttgtataaactataaaataaaataaaatatggaccaaatcctttcattcctacagaataagaa

35806 ggattttaaagaataatttgctatgtttcccctattgggccccgcccctcaggcccctcaggcccctcgggggcgagaccaccggtttatacaaaactaagtccc

35911 cttcgcccaaggatgcttcagaccaaatatggaacaaatcctttcattcctactaaagaacaagaggcccaagggccttagcggtcacctgagttatatcatagg

36016 tacgggtagctttattaggtcacctgagctttgctctagtgacctattccaatcacttttcgtccgtcgtcgtccgtcatgcatcatgcgtaaatttttacataa

36121 tcaactttttctcaagaaccagtggatggaatttttcaaacttggtcagaatcatccttggtagatgaggataccaaattgtaaaatttggattcctaccccaac

36226 cctaggggccagggcggggcccaaaagggaaaattaatcaatatttcaaaaatcttctccatactcagaggtattacagtcattttctctgcatggatggaaaga

36331 tcatcaggggctttatttaaattgttaatttcaggacccccgagctatccgttctcccttggggagggggtaaagtttactatagtttatatagggaaataacac

36436 ttttcaaatttcacaaaaccttcggcaaagccttgggtttcaccaaatttgaaaaaccctcgggtggaatttccctatcttacacccctgagccctctttatagg

36541 acaaatcattaaacatattcagagtgttgtaaagaattgaactgtactaaaaaccaagttaaagttctcttgcaatgtcaggctaaacctacaaagtgtcatttt

36646 catggttggcaaattttccatctctgaaatgttcatatgaccaatggtaacttgcttggacaaatgaaaaaagattatgtttggtaatttcatttttccagtttg

130 T K I D L R E N K D A I G Q L A S Q G L Q P V K R E H

36751 ataataaaaattgtctttccagGTACAAAAATAGATCTTCGAGAAAACAAGGACGCCATTGGACAACTGGCATCTCAGGGTTTACAGCCTGTGAAACGGGAACAC

157 G I K L A N K I H A V K Y M E C S A L T Q R G L K Q

36856 GGCATCAAACTCGCAAACAAAATCCACGCCGTTAAGTATATGGAATGTTCAGCTCTCACACAACGTGGATTAAAACAGgtaagtttctgcataaaaaatgtgtaa

36961 tgaccctctagcaaagtacagataacttcagagggctgtatgacatatcacttatatacaaacatgtatttcatctacagatgtagacatgtatgtcgttgatag

37066 acctatttagcatacacatttaaccatactcatgcttagatgttttagctgattgaattgaaattgaaattaaaacaagaatgacttccaaaaatgtatctccat

37171 tttatgcaatggtaacttaggatcactattcccctcctggatgtcactttgtccacttgtttgcttcaaatttcatgaagcaagttgaacagaaatctttgatgg

37276 gaatatatatatatgggacatattgttttaaattgaattgttttgtgtatatctacaatacagaaataggatctattatgaatttttataagtttgtataaacgg

37381 tgggtgaggcccttctgagggcggaggggtgggacccactaggggaaatagaggatttttaaggccaccatcatctgacggtgggctattcaaatcaccctgcgt

37486 ccgtggtccgtcgtccgtccgtaaacaattcttgttatcgctatttctcaagaagtactagagggatctttctcaaattttatatgtaggttcccgttggtccta

37591 agctgtgcagggcaacttttgagactgatcagataaacaacatggccgacaggcagccatcttggattttgatagtgaaagtttgttatcgctattcctcaagaa

37696 gtacctgagggatctttctcaaattatatatgtaggttccccttggtcctgagctgtgcaatgcaacttttgagactgatcggataaacaacatggccgacagtc

37801 agccatcttggattttgatagtgaaagtttgttatcgctttttctcaagaagtaccagagggatctttcgcaaattttacatgtaggttccccttggctctgagc

37906 tgtgcagggcatttttttgaaactgatcagataaacaacatggctgacaggcagcgggggttgattttgatattgaaagattgttatcgctatttctcgagaagt

38011 ctcggagggatctttctcaaattatatatgtaggttccccttggttctgagctgtgcagggcatttttttgaaactgatcggataaataacatggctgacaggca

38116 gccatctttgattttgatattgaaagattgttatcgctatttctcgagaagtatcggagggatctttctcaaattatatatgtaggttccccttggtcctgagct

38221 gtgcaatgcaacttttgagactgatggataaacaacatggccgacaggcagccatcttggattttgataatgaaagtttgttatcgctatttctcgggaggtacg

38326 gttgggatctttctcaaattttaaatgtagggtccccttgatccctcgttgtgtatgttatattttgagactgatcataaaaacaaaatggattaaaagaagaac

38431 taaacagatctttctcatattgcaatgatcgattctgtttctttcacattaagtactgtactagtaaatatgtgaaatgacaaacacactttgaaggttggcttt

38536 catacacatcagtattaagtttagatcaaaagttacctcaaggtgcatttactgttaacatgactcgatcaaacagattagaggaaagaagagaaaagatcgaac

38641 tgacaaagaactaatgaagatggtacaaaggtgggcgccaagatccctctctgggatctcttgttttctcctactatacaagtgaagggatttaaaccaaatgca

38746 gtcgtaactctttccgtaccgttgtcgtgtacacacgacaacaatatgtcaccctggtccccgttgtcgtgtacagatgacaaaataaagggccgtctcaacccc

38851 tttgtcgtgtacacacgacaagagactctgatgtaacctgcctgatgtttagttggttatcgcacgagactgtcgcgaaaaaacaattaattgtcgctaaaatca

38956 gaacccatcggtttggaagaaataattttagttgtgcagagtaaacttttttattatgtgacttaaagttttcgcatttgccgcaagatgtttgcgtttggtgcc

39061 tcaaatgtaaacaagatggcggaaacctaattcgatacgaacactcgtaaattcatcatcagatagttgttgagaattatggaagtaaatttgaatattctaata

39166 taagtcttgatgaaatctacttggtatttagaaaaaaaatcaacgactccacattcgtaatcataatcaaaccccattggaaacctgcactacaacgctgtgtta

39271 aatacaaggaatcgtatcgtttattgccaggcaaatgtcgatttctcagtcataatcagctgccagttagtcattatactgcgaaaattaagaagtgtttgtcta

39376 tgaaccagaacatgaaacaataacaatatttgttcacggttacctgtttaattcttgcggcagttacacctagtctaaaaagtacattaaaaatccgccattttg

39481 taatatgcgcgggggcgagtctaataagtacgtccggtacagaatccggaccggggttgcggtacggaaagagttaaacatgattagggacaaaagaatacattt

39586 tgtataaactgaggtgtgtgggcccccttctagggtgagatgggtggggcccattaggggaaatagaggaaaacatcaaagatccttctccttttgtaccgatga

39691 agggattttgaccaaaattggtgtggaacatcattgcccaaagggttctcagttttgtataaacggagggtatggccccccctgggggcagaggggcggggccaa

39796 atagggggaaaataggtaaatctttaaaaatcttcttctcaaccaatagaaggattttgttgttctagagcatctttggacaaaggatcctcaagtttgtataaa

39901 tggagggtgtggcccctctaggggcatggggcggggcccaataggggaaatagagggatttttttctccaactatacaaacaaagggatttaaaccaaatgcagt

40006 ctgaagcatgcttagggacgaaagaatcaattttgtttaagctgaggtgtgtggaccccgttctttggggcagatgaatggggaccatcaggggaaatagaggaa

40111 aacattaaagatccttctccttctgtaccgttgaagggattttagagcatctttgggcaaagggaattgaatatttataaatggaagatgtcccgcccccccccc

40216 ccccccccagcagaggagcatggcccaataggagaaatagagaaaaatattttcccttctgtacaaatgaagggatttaaagtttaccaataggggaaatggagg

40321 aaaattgggacataacatggaaactcattttcttggtgtctttcccaccaaaacagatttatatgtagttcttcttgcaactttgtttcttgtactcctttgata

40426 aacctgtatggtactcagcaggttttaaattgaaaaataaaataccaggtgagctattaaggccccggtgcctcttgtttgatgtttatagaaaggttgagtaat

183 V F D E A C R A V L

40531 gtttgttaatgtacaatttacatttatttgatgtttatagaagggttgagtaatgtttgttaatgtacaattacagGTATTCGATGAAGCATGCAGAGCAGTCCT

193 Q P Q P I R T K N H K C V L L *

40636 ACAACCTCAGCCAATTAGAACGAAAAATCACAAGTGTGTTCTCCTCTGAttgttgatacatcgtttaaactgtagccatgtgacctaatgtggcaattttaaaac

40741 ttatcatcatcggctgaatctcttctgtttcctgattggctgttcatagtgtcataataacttggatatattctctaaagtgaattcagtttatattcatggtga

40846 cgacagcattttctattgattgcttcacagatgctttggactacagtgtgtaaagtctgggatatctgatcctgtgttttttctgaggatcatcagtttatgaaa

40951 tgtacagataaaatgtaatgggaagagcctggaatcctggccccaatttcttagaaaaaagttcagacttaattttccaataaatttgtttttgtcttattacct

41056 tatatgaatatgaaagtattagaaattattcaaaaatgttattgcgagaaactttcagattttgctctgatgttcttgaccctatttcaaagtttgagggaaacc

41161 agctattattttcaaatagaaaatatggatgattaattttgttttttattaaaagaagtgatttcatggcaaaaacaaaacattggagttaaatggggcaagaag

41266 ccagtagggctgtaacaattcactgtagttacaatttgatggatttttgattattttggttcttctgaattttttttattcaattattttggctgtgaatgttaa

41371 tattggcaatattaaccgatgaaaataactattaatactacattttaagttggtttataggtggttggtggtgcagtggatagcgcacttgcctttcaccaaggc

41476 cgccggggtttgtttccccaatcggacctaaaaaaggtatggggtcacctgcccgatcacattgatttctccaggtattctgttttcctcccacattaagatccc

41581 ctgcatgctaacatccgggccaacaagaatgattactataagttgtaaaaacttgtttcataattgttgtaaaataaataagtttatgtatagtcagtatatatg

41686 cttactatgaattaactcaaatttcaggattttctaaacaagaatcgaaccaaatgaaataaattgtatcttgaactgtattaatcgctacagcactaggagccg

41791 gtggtataaaaggaaccaaaatgatgctgctgattcacatggaaacagcgaacctccctccacagattctcgcggaaccccgttgccagcaaccactgaatgttt

41896 gaactggacaggaatgtagtgtcttatcaatgatgaaatatttgttcataatatgaaggatgaaaaagaaacccaagttatttctattgccatatatatatctag

42001 aatgtttcatgtgtgtatttatatatatctcatctcttgtgactccacagattttgctgaaactttcaaatcaattttataacctgtgtgaaagacgattcttta

42106 aatcagagcaacctgttcaactgtaatgtgatcgttgtacaaccttagttacttacacaggggtggcacagatcctgaattccaccgcccatgtatatatgtgta

42211 ttatgtaccgtgggatttggtatatgtgctacgcccctgtcgttacatggattcagttaaaattagaatatttctacatgtgattaccatgtgttgctatcttat

42316 atcggctagtcagaacatgagtgctgttctctaagatttgatgttcaggaagtttttttttgaaaatagaaaaaaaaaattgaagttagaaatcatttagatatg

42421 aagttatagaaaataagcttggttacatagaaaacacagactcacacacgtaaaggcagcctcttgtacatttatttgtcagaaagatagctatcgcacaattgt

42526 taaagaaaccttaatcactggtggctccacacagaattttacaagctatttcatagaaatattttacgttttaaacgattcagttttaatttagttagtcgatgt

42631 gaacaaatgttgtataattagcatggttgcgctgtgatcagtgtttacaactttaggacacttggatcaggaaattaaaaaatattatctcttgataggttttca

42736 aattttattagtactattacgaactgtagacaaattttaacaatacacaggttattcttggatcatgcttttgatatgtttgtgaaagtatttgttcacttgttt

42841 gatatttccacaaatctagagaaagatttcttgggtgaatgtgttcacagtagtgctggttttctctgccccccctaacctattcgctgtaaacagctcccctcc

42946 aacctgttataagcaccccctctcctttttgggcactatatagaaatgacataagcgcccctttcccaaacatgctctattctggccctctcctgttaacaaaat

43051 atttattagttaactttatatccgacacttcagtctcgtaatctgacaacacagtgtccaagtgttttagaaaaattaaaagaacagttatcaagttctccctgt

43156 gaatatgtccgacagttacatcacctttacagcaacggacacaacctacattttgttaaaggtgtctctgtcatcttatttttaaagtgctctgggctgaaatta

43261 tgatttgaatatgataatgtaaataggtggtttgctcatctacgacttgttaacatcaatagtagtgacgattgaccatactgatcgtgacaatcggtaaataaa

43366 caatctttttatatatgaccaaacctgatgctttccctatttgaattttacacgagcacactgctttcacattttttgttgtgtattgaagagttatctgttacg

43471 ggtatgttggtacatgtatgtaggtacaatgtaggtaaacaattgattttgttgatgttgtcattgaccttgattattgatttccttactaatgctttcatagtt

43576 taaacctggacaaccccacaagaaacatttgataacctttcaatatttctgaattaattaagaattaatcatttctttgaggaaatataatttaaattcccaagg

43681 aaaaaacttgaatagaaggatgaagatgtttaagttagagtgtgtaggggatgactcttaattatgtgaaaatgaagttgtgaggctatgccctggggttgtatg

43786 tccctgtcagtgtggcccctccctatggtaaccctatatcccccggttgtgacaatatatatatatatatggactatttttggttgatatcattagccaatgcaa

43891 gatcttattggtaaatattaatggagtatatgaagatataacataaatcatacccttttattctttagtgatactagcattcaatataaatccctagccatgcac

43996 aaaatatcatattgctataattagtattatatagggtcactgttgcttcatcacattgtgtgttgtgacctatagtgcaacatgtcatcagattgtgtgtatgtg

44101 tcattgtctcatcttctctttgtgcgtttaattactgtcctgtcatcacaatgtgtgtatcgggctgatttaattcaaacatcattgatatcccatgatatccct

44206 agcttatataattacacgctgtatgtatccccctatcatcaactaccattcatatttggtcctaaattgacaattactcatattgatttcatttctcaacaaata

44311 ttcaggattttttatattcaattcaggaaaaaaaaaagtttgggtatctatgtgaattgaattgatagaattttgtaaaagttattttttttactatgccagcca

44416 cagtttttaggtcacctgagcgttgtccgtcgtgtgtcatgcgtcgcctgttgtccgtcgtccgttaacattttaaattttcaacttaagctgaaatactggcaa

44521 aaagatttcattgatacttcataggaatcttactgacaactccctgaccaagtgttgttacttttcgggtcgattagaaatccaatatggccgccacggacgcca

44626 ttttgaaaaactatttcaaacttaaagtcaacttctgctgggccgatttaactgaaacttagtaggaatgatcgactcatggtcctgacaatgtgatgttatttt

44731 tcggttcgatcagaaatccaatgtggctgccacggccgccattttgaaaaactatttcaaacttaaagtcaagttctactgggccaatttaactgaaactcatgg

44836 tcctgacaaagtgttgttatttttcgggtcgattagaaatccaatatggatgccattttgaaaaactatttcaaacttaaaatcaagttctactaggctgattta

44941 actgaaactcggtccaggaatgatcagctcatggtcctgacaaacttaagttaacgttatacttaactatttttactccaattggtacatatttcaacaacagaa

45046 ataaatactgtagactcaggtgaccgttaaggcccatgggcctcttgttgttttatgtagttttaagttttttttacacaatgcaacttgttgaaaatcacaatg

45151 tgttattggacctgaccatgcttgtgcctttgctatgtaaatagaaacatctagatgtatgcactttgcttgtgaaatatgtgatggcattatttgtcactcaga

45256 aaaagaaggggtagccgatattttaaacagaattttatgatctcgagtattttacatgaaatatatactccgcattttctccaagaatttcatatgataaaaagt

45361 tagctgaagcttttgatctctatacccaaggaactataaaaccttctcctacagtacaatgtctctacagctttatactcccttagttataattttatcaaatct

45466 ttacatggcctaattaccagttgtgaggatgattaactttgattaaaaaaaatatgataatatgtgtaattatcaaacatgtactagatgaaattactcaattta

45571 ttaacattgatatggattttattt

**PyMiro**

1 tatgtatgtatgtcccagacagtagtgatacatacatacatggtgaaccacgtaccactactgtgtgatacatacatggtgaaccaggtatccctactgtctggt

106 acatacatacgtggtgaaccacgtaccactactgtctggtacatacatacctggtgaaccatgtaccaatactatgtggtacataccatacatggtgaaccatgt

211 accaatgctatgtggtacataccatacatgatgaaccatgtaccagtactgtgtggtacatacatacacggtgaaccacgtaccaatactgtctggtacatacca

316 tacatgaggaaccatgtaccaatactgtgaggtacatacatacatggtgaaccttgtatcactactgtgtggtacatacatagatggtgaaagacgtatcaatac

421 tgtctggtacatacgtcgtgaaccatgtatcaatactgtctggtacctacatacatggtgaaccatgttccaatactgtgtggtacatacagaaatggtgaacca

526 cgtaccaatactggctggtacatacaatacatgatgaaccatgtaccaatactgtgaggtacatacatacatggtgaaccttgtatcactactgtgtggtacata

631 aatacatgtggtacatagatcgtgaaccatgtatcgatactgtctggtacatacatacgtggtgaaccatgtatcactattgtctgggacatacatacatggtga

736 accatttatcactactgtctggtacaaacatacatggtgaaccatgtatgtatgtaccagacagtattggtacgtggttcaccatgtatgtatgtaccacacagt

841 actggtacatggttcatcatgtattgtatgtaccagacagtattggtacgtggttcaccatgtatgtatgtaccacacagtagtgatacatggttcaccatgtat

946 ggtatgtaccagacagtattgatacattgttcaccatgtatgtatgtaccacacagtataggtacatggttcatcatgtatggtatgtaccagacagtattggta

1051 cgtggttcaccatttctgtatgtaccacacagtattggtacatggttcatcatgtatggtatgtaccagacagtattggtacgtggttcaccatgtatgtatgta

1156 ccacacagtagtgatacatggttcaccatgtatgtatgtaccagacagtagtgatacatggttcaccatgtatgtatgtaccacacagtattggtacatggttca

1261 tcatgtatggtatgtaccagacagtactagagtaacaggtcactatattggtatgttgtgcaccatgtagaccatgtatgtatatatcagacagtttgaatgtaa

1366 acaacaggtccccatatcagtacatggtgcaccatgtagacacaccatgtagacacaccatgtagaccttatgtatgtttattgtatcagacattttgagtgtgt

1471 aagactgtaagtgacgggtcatgatcatcatattatataaaatatatcttgtaattattttacaacaattatgaaacaaattctttcaaattatattgatatcac

1576 tcttgttggccctgttgttagtgagcaggggggtcttaatgtaggaagaaaggagagtacccgagaaaacccaattggttgggcaggtgacatgtaccatacatt

1681 tctgcatctgatcggggaatcaaaccccagccaccttgttgataggcgagtgtgcctaggctatccactgtgccatccaaccattatatagtaatcatattgata

1786 catagcacactatgtaccagactctggtggagtgctagtaacagatcatggtattggtacaatggtgtcaccttataactggctgtagcacatcacttttaaggt

1891 ctagaaacctccaaatttgagtgcatgataaaatctgttttatctctgtatgatatggtcaatggtagctgaagacaccatataaattgctataaaagtgccagc

1996 cttaatattgacagtgctataaaaatggagatatcaaaagtcatttcaacttatttagaggtaaatgtcattttagtttatgaattccatccaaaaatcattgac

2101 ttttcacaacctgtgatattgattttatagtgttacccacatatgaatgacaaattaatgatatatacttagatatgaaatgtgatttatatttgtgcttcagca

2206 ataattcaaacgttattgacaataaaacactaaaaaaaaacactaaaatttaatatatataaaatgagtaagaatgtttcaatggatctgatatgcctcaacaaa

2311 taccatactgttatagcattgacagtgttttgtaacagatctacaggtatatccaggaagtcggtacttatttttacactaaaacttcacttactctacacattt

2416 tactgtaatgaagagtttggagtttctaaatgaaaagttaaaataaatttatatacactgtatatatatatgtgtcatgttatgtaataattaattgataaaata

2521 tcactttaacacaattaatcataccaaagaaaataattccattaactgcaagttaaaaatggtttaacaataaattctctaaaaagaaaagttttcaaattaaaa

2626 cagagactatgacactgtaaatgtacagagagcatacaaaatactgtactgtaatatacacaggccctttaacaaacacgtaacacaaacacatacccttctcat

2731 tggcaaacaataaacaatgattgtgttcaaagtttgataattatcattagtgtagcctaagcaaggtcacatgtgtgggattaatgtggacttgttcaggtctat

2836 caattaaagtgatgttgtaattagaaaggaatgacagggaaattaaagaataacttaccttctctgtttaacctttatcaggagaccgtgttcctgtacctatat

2941 aaatctgtgagagcggtgttttttaaccaccacgtatatagaatagatcctgttctaacaaattgttgaagccaattgaactttaacaattctacaggtgagcat

3046 ttgcatttatttaaagggagcgctattttgaagtgacccgttaataccttcagattgtgttgaggataaatttaagtctgtgtttataccggtagacggtcccgt

3151 ggtcacagacctaaatcgtactgcacaggtatgaacctgtaagctgaatcgtgtatacctaccactaatctggtcttagacacccatcaatctttcaataccaag

3256 tatgtacacaacaatttgtacaaaccagtaccagtgaacacatgtttttaatataaatgttttgtggtatttcctatctggaatatatgggacaaacatgacatt

3361 ttgccttctgaccagtacaaatcaattatgcatttaactaacggtatacacattattaatattgtggacaatcgcaacatttcttgattttaacgtctgtttaga

3466 ggaaagttgaaaataaatgaaattttccaaattgatctaccattcattagacaaggtaaccacagtaacaaagggggcagcttttttctttcacattcaaacact

3571 ctgtgctcttccataatgaccaaaaagagtgataatgtaagtttatacttgttatgcagtaatgttgctaaaatatatgatttctttaaaaaaattaaagggcag

3676 gatcgagtcaagagggaagcttccatttctatgtataagaaattgatacaaatgacagaccaatattgtgaattgtattattgtcaactgataatataaatactt

3781 gacacttcattctctgaccttgataagtaacttggtctagaaatgttcattgtacattgtatcaatgtatctaatcctatgatcagttgatctagaaatatgcac

3886 tggtcataaatcaatacatctatcagttgatagaattgtttgtacacaacactaaagagctgttactaaatacagagagatggatggacactcaaaacaatttta

3991 acatctgtgttttttattgtgcatttttttttttttttaaatactgattttaaaataaacctgacaaattaagtattcataattaataatttgtaattcatggtc

4096 ccaaatctcccaacaaacttctgtcaaccagatcgaggcccagtattgctcacctggatatttaagcactcattgcaaaatattctcaataaggtaaaaggtacc

4201 aattaatggatcagttacggtctatatccatttctcagttccagagaacaggccacttaaagtaatatggtcaaatagtgcttcttaaatactctgcccctccat

4306 atttgtacttcttttttatccccacctcctaagaatgcttcagacaaatatagttaatttctatcaaatggttctagagaagaagttattttaagcaatctagcc

4411 aaatatcctcttttttggttaaattccaatcaatggttaagaagaagtcattttaaagaaacagatgaaggacacgtaagataacacaatgcaacccatcgttga

4516 tggacatataaaaaacatcacatgtggtgaataaaaatttcagaaatgtgttgtgatacaaatcaagtgttacaattttatagagagccttccatatttttgtca

4621 atccctattgcatgtatcaagaagaaatatatgtttttctttaaaatactttaataaagatgtacaatagactggtttcctggtgacatgacatcacagacatca

4726 catgaaaatatcttcaacttgtgaatttttacaaacaggctagctctttattaccacaatcaactataatcttcagtgacagcacttaaaccttttgaaaagaaa

4831 ggcacccagtaagtgataaatttgattaccgacagaaatatcattttgtgaaattagtctgtaaacaaattgaagtctcttcgatcttttgcttattttgatata

4936 aaaacagggttaatccggccagggacactaaacttgtatggtcttacatttatacacggtgaaatcagagagggataagtccctgaccgttctcaggtatccaat

5041 aaaccaagaaaaccacttggtgcttatacattccttaatacggtacaaatatgtccagacagatattcaatgcatttaatcacttatacatgcccatgctaaaaa

5146 aaggtacagtgtggcatgcctatttcatgcctaaaaggtgcctaaaatgtacctctacttctggggtattttttaagtatacctcaaatgtgccccgtttggact

5251 ctaattttatcaatgtgcctcaaatgtgccccatttggactctaattttaaaaatgtgcctcaaatgtgccccatttggacttaggtctcttccacatgcctcaa

5356 atgtgccccatttggactctaattttatcaatgtgcctcaaatatgccccatttggactttgatttttttaatgttctccattaggacttatagatttcttccac

5461 atgccttaaatgtaccccatttgaactctgatttttcacatgtgccacttttagactttgatcagatttgacatatacttttaatgatatttttttttatataaa

5566 attaaccatcaaaatgtcttttttgaatgccatttgcaaaatataaattatatgacaagctaaacgaatgaataaataaacttgaattctgtgaatttacatggt

5671 ctaattcagagacatgtaaagccacgtatctgtcttatataaaacagccatcttactctgcatgtcatgtttgagaatgtaatgcttatagtcaggttatgaaac

5776 tcgatcaatatgcacagtctaaaaatttagaattggggcaacatagatataaggtctatttgatatagatttctaatagacttacagaaaagtttcacagaccag

5881 accataacaaagaacattttacttggtctataagtaaataactccagtagacctacagtgattctatagtgattgtctatgggtctaccattatgttgtgacaaa

5986 tatgacattgtatgtacaaattcattaagtatcttgtatcaggaagtgcaaagtttacctacagttcttgttaacaacatcaaaatatttcaaaaacagaaaaga

6091 atctgcaattaaatacaacatacagatatgcatataaaccaggcccaaaactgactgcatataaccaatcccggagcgaactattaattatatataactgttcta

6196 atggaatgtaaagttattaaataaccagtagtaatagttagtaatatgaagcccatgaaccgcagtttatttcactgttatggtactataatcgccccacggtga

6301 agtcatctatttttgttcttttcaacttcaagttcattataaaaaaagtcacttttccttttattgaatttttccaatttgttgatctatatgtggaaatatcag

6406 gaattaggtatggggtggaaattttagggttaggcattggttattcactttatgcatggagtcaaataaagtctagcttcaaccatgaattttgttataatcatg

6511 caactctttttgccagaaaagactgtctaatgctctctttgatactaatactttttagaaaaatatgaacgattcgattggatccaagtgtatcaatatgctaat

6616 catttgacatactgcatgttgaatactttataggagactgacatgtcaggtttataatttatggatcaaattattcaaccccatcaaagaaaacagaaacagaaa

6721 tgttactttttctaacatttgaaatcatcaaataaacacagccacatgtcatgcacactggaactggttgattgcatggaaagggctataactctgtaggacatg

6826 gcaatatgacaaataatgttgtatcaattcaacattcaaggcaaaacacaaacataatcttcaaaccttcaagcttataaaatgaagtattcaagcattttcacc

6931 ctttcttggggggaaagagtgagagcatatattttgtgtcaaatcccctgtcccacattctcaataaaaagtgctgggttattttgcttcaaaaatgtcctaaaa

7036 aatttcataattactttttattacttgatgtggttagattttttttttataatttctgaaaattcccaaaactgtgaaaaacatcctgctgtgagacaaaggtac

7141 atgtactacttgatcttttagtatttaagtcctttactaaaaacaataatacccgactgaaaaaaagcctgtgaagcaattttttgtggtaaattttgcaagtat

7246 tgaagatatttcatttgaattaagtttaacagataactggttcccttctaaataatttaacagaaaaaatgtagcaccatttaacgaaacctatcccccatattc

7351 cagtatctattaaataccacatgtgtcctctacacgctgagatcactgcctgtaccctactatttttgtatttacctttattaacctcaagtgatcatgttggcc

7456 aggtggtgagtttatgctcttgttggtcttattcaaaatcagtctttactgtgcatgcaactaatgatggttgtattgtataaaaagtctagctgactgggtaca

7561 catatacaatgcccaaagttgggggtgcgcagaatacataagtgtaagcaatttcaagttttttcatgaaaagatcaacgattctggcagaccgccaacttggac

7666 acgagataatctcccttggtacaaggaactttttgtccttatccttccagtctgagttaaagttgctcttgaacatgttaatagaacttgcagtcacagttgatt

7771 ctgataatgaatatttgattctttgaactgatgtgtgatgttattccattactccagaggtgcctgcatcagctctctgagctgagtgacggtttttgggggtat

7876 tggcttgtttttaacctgcctgtccaactaataccacaaatctggactgtatggtagccaatcaagaagtagtacattttttgggggctggaaaagcatgacaca

7981 ctcttgcgcaaatgtcccaatgtgtggcctatagctggcattaatattgtcttgcaggaaggttagatttcattgatggataagagtaatgatgtttggctagag

8086 gaatgatttacaagttgaactgcttcataggtgggtatgcccaagatgcttgaattttgacaatgttcttaaaaatgtacagcaaggttttgtatttgggttttg

8191 cacaaaggagccagcaccctaggcttatgagtgcccagcgcaatcactaccaaatttaaatgaaatgtttgatattttgaaatttgtaaacaaatattaaggtat

8296 gagccaaaaccaaagttgcattactttttttccataagtataaatacaaattaaaatactgatcacttagataacacaggaacctagctagtacaaatcttctta

8401 ctttgtgcggcaaaaaaaatgactctcacaataagaattcatttttttacataaccgtggatctagcagacaccttaccaatcaggtcagtgtccaatgattacc

8506 gagttttcataatttcatctgtgcataaactacaataatcatcaacgattaacaggtaattaaattaaaggggccaaaaaggttgtgattctcctattaaaaatt

8611 attgcaccgcttgtctccttctcagaagtttttcttatatctatagaactatgacaaagagaaaataactaaagaggtaactagggtggtgggtattttttcatc

8716 aataccggtatatacaaacacaattttatgagtcctttgatttggactgatcatttactcgtagaatgggtatattgatttaaggcatgatttataactaatcaa

8821 gtcagaattaattggtaaacacttaaactacccataatactattgatttgtaaccgagtgtttcaatattatgtgacgcatggatgtgagctctacaatgtatca

8926 ttgattgatccatgtacattcagcggcctacattcagctcaatttcaagtgacagttcatggccggccggcatggtgttgtgggctaggtcgagtgtaactgata

9031 acacatgatgatcgtctttatcgctttcgcagagtgttaaatccaaatgttttaaatactataaagtacttaccattcaatctgccacatatcacacctgcgtct

9136 cgttgctgcagacgctagccgtatttccgcaagtgcgcgcgcaaataggaagcttttatcaatcggaaagtttctattttgagacatgtatctccgataaacctc

9241 gaaaacctccgtattgtgaaccaaaaatggcgacatttggtgtcttttaacgaccatgcagttgcacttcattcataaaatactcttcgaataagtaccaagttg

9346 aagcatacggatattgtttaaatttattggcatattatgctgtagatagtgcattcaagacattgtttgccgttaataatgtgaaataatccacatgtgatgtag

9451 cgcgagtttgacctttcttggcgtgctttcatcatcatgagagagtaaacattacataaattcagaaaagtttacaaggcactgtctcgtgcatccaagattata

1 M S S K R D V R I L L V G D P

9556 tactgcagtATGAGCAGCAAGAGGGACGTCAGGATTCTCCTTGTTGGAGATCgtaagttttcgtttgtttcaattgtcagtataagttatatacagatacagtag

9661 agactagaggaaaatgttatttcttgtgcacttgttgatgatgatatctttgtaacgggacgatatagtagtatggtatagttataagttatatttctatatcgt

9766 ttattattatactatatcgtcccgtaacaaagatatcatcatatgttattaaatactaatatgaacgaaatagtccagcacttgacggtctgagcacaggggttt

9871 gttacaaatgtctaatatacatatatatatgctatactgatatgggtcacagcacaccttaacatacactagcagtatatgtgtattagacatttgcaatgtcag

9976 aacaccttttgttttgttttgacacatatagtgtcatcccaatggactagaaccacaagagataaaaaaaatgtacagtataggctatatccaccctattttcca

10081 aacattgattgaacatgtgatctcgtgtgttttaattaagtaccacatccatcaggaatcaacacagtagctgcccagcctaccaaaagtgctaaaaggaaatta

10186 cctctcaaactagcccgagtttcttctggtcctgacaccagacttagacattttgacagatagatacatgtatgttgtggggccagagtgcactattatatgaaa

10291 acatggaattctccgacaccgtttggtatcagagagttttgaggtgttgacaacaaaaatatgctttgtgactaatcaacaaaccgtactgatctggaccgaaac

10396 agagactattactctcgtttagatgatttccgcaccacatctttttgttgattatagtcttgtgcactcgccaataccaaacggtgtcaaagagaattccacatt

10501 ttcatatagtagtgcactctggtcccacaacagacatctatctgtcaaaatgtttaagtctggtgtcggggccagaggaaactcgggctactcttaaacaaagcc

10606 agaagaggatagccagaagaggatttatcctgattgttttgttatccaaatctgtcactttaaaaccattgtgtccttttaaatatgttcatccaaagcaccatt

10711 tcaaatcaggttcaataattcattgtcgccacatggagggccatggggcgcctcagcagctgtgatcgctaatgcacacagtcattgtactactttgtgcaatca

10816 agcataaggtcagaccattcgctctcccaagaatttcagttcgatcccaagcggtggcagaaaaaagagagtgatgtctgttagggttagttgttatccctccga

10921 tngtctttttttatacccatttatcctttgtatttttctgtcaataatcagttacatgtcatcatgaggagagtatctgctgcaaaaatattttaaaatcctgta

11026 tatcatacatgtgtaaatgtagatgttgaaaccaaaatacaaaatgctgtcttcatctgaaataaaactcaaaccatgaacattatagtgtatccttgaggctta

11131 aaacaatacttgtggaatttgtagtggaatacaaaggtagatttgatggaaatatttggaataaattacaagtatatgattagccccaacttagcggggctgaca

11236 aaagtacaatgagcttatggttagcaggtttttttgggggggactttttggagcaaataatgggcctcgttccaaatcgaactatatttagttaatatttatgcc

11341 tgattcccgaaatttgaagcttactcctgaaaatttccttaccaattggtttattttcttccaaaaatgagacaaaaggaccccttccaaattaacgagagagaa

11446 aacaactaattactcaatttaggtatgcaatctaaaacttagatggaccttatgagtgtatttttgcttggccttatgtccattatttttctcactaatattatt

11551 gatagcaattttaacttgcctatttagtaaagcctacctggttctacagaaataaactacgttgcataaattttaatgaaaagtaatagatacaatgttaagaag

16 G V G K T S L I L S L V S

11656 cataagacaaaataaagacttttatcaaaaccactttaaataccggtatattttatgattacagCTGGTGTCGGGAAAACATCTTTGATATTGTCCCTGGTTAGT

29 E E F P E E

11761 GAAGAATTTCCAGAAGAGgtaagtgtttgacattaataatagtggctataatgtagttaatttgagacacaggacatgcacatttgtctaaaagctcttttcacc

11866 acatagtaacaattccattgtgttttaattgacataaacaggaatgtacaacagccaagaataccatatatgtaattcagacagctctctggaacttgtccaaat

11971 atcacaagtgaaaattgatagaagtactggtgaggagtcaatgactcttgtttgatgttttccccgacaagggttaaatgtagcagtttcctgttatcatgtact

35 V P S K A E E I T

12076 tcatgtactttaatttgtcagctgcaatgcttcttgtgtttgtagtttaaaatattgtgtgtgtttttatctccatagGTTCCATCAAAAGCAGAAGAGATAACA

44 I P A D V T P E K V P T H I V D Y S S

12181 ATACCTGCTGATGTTACCCCAGAGAAAGTACCCACACATATTGTTGACTACAGTTgtaagttgatcaaatttcatgcatcatttatgaaccctttaatgtattat

12286 agaactactgtctaccatgttgtttattgccaattccattttagttggtacttaaaaaaaaagtgtataactgtttattaattttaccatatttaacaaacaaaa

12391 ataacaacctttaaatgtttaaaaagaaaaacatatttatcttgtaaacaagcatttgtcttcataaaagcttatttcatatttggaatcatatgagaaggaggt

12496 aaatttaattatgaaaaatcaaattgattactgtaatattatgataagattttagcagaataaattgataacaaatcatgaacaactagtatagttacatgtact

12601 accaggtgaggttataagtgtgactcactaattaatattatgtcaagtagtctgatgttaacaaaattaaactcagtctatacacatttaatattataaacatga

12706 aatatagggattttactttttttttgggggggggggggtgatcaaatgaagataaaacacatcaacaaaggtatctgtgctgatatacttaccagatataataac

63 Q E Q D E D R L Q E E L A K

12811 ataacaacaaaactatatcttactgatctgtatttacagCACAAGAACAGGATGAAGATAGACTTCAAGAGGAGCTTGCTAAGgtacgagcctgtggtatatcag

12916 ataatacatagtggtgtaggtttagtaaagagccgatgatgggattagaatttgatcaataaacaacttgtgaagacttgtcagttctgaaattgatgtcacttg

13021 ttaagacgttacaatcacaaacaggttaaagaagaaaatcatgttttactttatagagaagctaataccaaaaggttgggaaaacattcagatgaagtgaggcat

13126 aaaacccatgttgtgtactacatatgtgtagtgctaagtactacaattgcaagtctactacaagtataatcattaatgtgcaatagttgaaataagagtgtagta

13231 ggatatctatcagggtgaacattgtgttgcctgcagacaccacatactatgtattgttctgttgtcccacgcccaaggcggaatccagatggtttccagcttgca

13336 tttaaaaggaagtattatgttttatgaaactgggtaagagtatatattacccaggtagtaccggtacgcaatctgctgaatttttaagaaataattttgatatgt

13441 taagttttttttacaaaataataaaaatattcttgatttgatttaaatacatagtctaagagtaaacaaaagattgttctgttgtcccacgccttaggcatgtcc

13546 tatgggaagaaggaaccttcaagtgttaaacagcttactttcagtacagttatactacatgtattactgaaaaaaaccttctgcatcccttccattagtgataaa

77 A N V V C I V Y S V D

13651 tattcagtttattacacaattaatactgttgtgtggaaagaggtgttatatactgaatgattttgtattgcagGCCAATGTTGTGTGTATTGTATATTCTGTGGA

88 D E D S I E R

13756 TGATGAAGATTCCATAGAGCGGgtaagctcaaacttcacagctaaaatattataatttatcacttacttacactataaaacctgtatctattgaactgtctgata

13861 attatgttcagccattacttttcacgcaaaacaatttcattatttgaaaaatattgtcaatgtgtgctttattggttggctgctggttatttttgaagaattttg

13966 ttgtctgtttgtcagttacaggataactgagaaaagtatgaatttgtaaatttaataaaaagaaattgtctgttttatccatgatatattttgagaaataattat

95 I T T F W L P Y L R N C L G Q D H R T P V I L V G N K T D M L D F

14071 tgacagATCACCACATTTTGGTTGCCATACCTACGTAATTGTTTGGGGCAGGACCATCGGACACCAGTCATCTTGGTGGGAAACAAGACAGACATGCTGGACTTC

128 S T M E

14176 AGTACAATGGAGgtctgtacaagataattttaaatcaaagtaaacacagtaggatgtgtattatagaatgaaaagttacttgctggtgtgacaaaatttcaaaaa

14281 gtacactttgtgaaaattgagaataaaaaaaatctttggtaaaatgcttgaggttttcctattgttttgatacatgtaataggtttcatatttaatgaatggttg

132 T M M P I M N D F A E V E T C V

14386 attcagactttttttaatgtgattttatagtgttacaaagtgacgtttactgttacagACGATGATGCCTATCATGAACGATTTTGCAGAAGTGGAGACTTGTGT

148 E

14491 AGAGgtaagaaaggggatctgatgaaatcattgatgttggaatattggaataaatgttgaaacattgcaataaaacttattcaaattcgaagactagaatgttta

14596 gctataaaatacttgatgtaatatctcatttgtattgtgaaatttaatctcttttttttcttttaatcattctgaacaaaccaaataactatgaaaaagtgaacc

149 C S A R T L K N I S E M F Y Y A Q K A V L H P T A A V

14701 atttgtatatctttttctttaacagTGCTCAGCAAGAACACTAAAGAACATAAGTGAGATGTTTTACTATGCACAAAAGGCAGTGCTCCATCCCACCGCAGCTGT

176 Y N P E E K E

14806 TTATAACCCGGAGGAAAAGGAGgtacagcacttcatgttatttacagtcttacccatctaaaccaatgttatattttacacatttttacctaccctgtctcttgc

14911 tcataatgcttgcagcctgcatacagataagttgttgttttgtttttgtatgattaagacaatggttttgtgttaattcagccttgtatactcaaatttcttcat

15016 agtacggtatatattcaaatcatgaaatggcactagtcaaacaccagagagcctggttgtcatgctattattttttttacgcctctgaatgttggtattttgtct

15121 taaagagtactgtttgactaattaataaaaaaaatataaaaatggtgtttgataaaattgaattatcganattgactaattaataaaaaaaatataaaaatggtg

15226 tttgataaaactgaattatcgagacatattgatgatagaaaataagttaagaaaacaaacagtgccatcaactaatcaacttttggtgactgttataaagtattc

15331 tgtctgttagttgttatttttacaacatgaacaactagataaatacatgctatttagtgtggtcatagatcaaaacagtggtatatatccaagatggcggacatt

15436 tgtgaacaggggggttgcttgcaattaagccccctccgaacttttggcccatcttttttctcaggaggaaagggggggggggcttattggcggtaaaatatggta

15541 tgtagcatgtgagttaaatattggattcagatgtattattatctgcattatatttcaatgtgtgtcatagttttaacaattattgcgttattataggaaaatgct

15646 gaaattatttgacactgctatgctgacagcagctatcatttacaaacacggttttgctggaaggaataagaatatgttagtttttagattctgtttaaaagcaaa

183 L T P Q C K K A L T R I F K

15751 aagctttcatgatcacattatttttgttacagCTGACGCCACAATGCAAAAAAGCCCTTACAAGAATTTTTAAGgtatgatacagacatgcacaaatatattaac

15856 ataaagtttagtcttatatatcaaggtaattattgggaaaactctgttgcataaaagtaatgaaatgttgttaatattgatttgtcatttgaggttaaataattg

197 I C D L D N D S I L K D D E V H L F Q

15961 ttgacatataatatattttgtgtatatatgtgttgttttacttcagATATGTGACCTTGACAACGACTCAATACTCAAGGACGATGAAGTACATTTATTCCAAgt

16066 gagtccttgcactttattacagattatcttattgagaaatgacggggtagagagccattattatcaggtttgactggagaacacatgtctgtaacacccacttgt

16171 catgatgctataagttatagaacacatgtcttgtagttcaagttaaaattgaaagtacctaaagacttcatcatgatatccttaaggtttagaatgttgactaca

16276 tggtaaatgatttcccctgtatgacctataaggtttagaatgttgactacatggtcaatgatatccctgtatgacctgtaaggtttagaatgttgactacatggt

16381 caatgatacccctgtatgacctataaggtttagaatgttgactacatggtcaatgatttccctgtatgacctataaggttaagaatgttaactacatggtcaatg

16486 atttccctgtatgacctataaggtttgaattttgactacatggtaaatgatttccctgtatgacctataaggtttagaatgtttactacatggttaatgatttcc

16591 ctgtatgacctataaggtttagaatgttaactacatggtcaatgatttccctgtatgacctataaggtttagaatgttaactacatggtcaatgatttccctgta

16696 tgacctataaggtttgaattttgactacatggtaaatggtatccctgtatgacctataaggtttagaatgttgactacatggtaaatgatttccctgtatgacct

216 R K C

16801 gtaaggtttagaatttcgactacatggtaaatgatttccctgtatggcctatgtactagtgagatgcagtggtaagtgtattgctgtatcattacagAGGAAATG

219 F N A P L Q P Q A L E D V K S I V K R N I T D G I I N N G I T L K G

16906 TTTCAATGCTCCTCTACAACCTCAAGCCTTGGAGGATGTCAAGTCAATAGTGAAGAGAAATATCACAGACGGTATCATAAACAACGGGATCACACTCAAAGgtag

17011 acagcttattttggtcaagacgagttcatcttgaagtagattttggttgttattacctgtttcttcactaaattttcactgaaacaaggtggcaaataaaccaag

253 F L F L H T L F I Q R G R H E T T W T V L

17116 ggttactattttaatgtctgactatattttcttgtttgtcagGGTTCCTGTTCCTCCACACACTGTTTATACAGCGAGGCCGTCACGAGACCACGTGGACTGTCC

274 R S F G Y D D G V L L S K D F L S P R

17221 TCAGGTCGTTTGGCTATGATGATGGTGTCTTGCTCTCTAAAGACTTCCTCTCACCAAGgtaaaacatgccacactccgtctaatgtttacgtgatacttcaataa

17326 gttttacaatttttgtaaactcctaagcatcaaaggaaaccgtcttccaacatgaaacttaatgactgttatgtaaagctgttcaagtagtgaaaaaaattaatt

17431 taagagcattcatgtctaacagatatttgtagctatagttttcatgtaaattgccattcgtgtactgcttattgagaatcaaaatattttcaaattaaaaactta

17536 tgcaaatatgcaaataacttattcatctgcaaaagaaaaaaccaaaaaaacaggtagtgtaaccatgatttgcctgtggttgaagcttcattttttttttatgta

293 V Q T S L G S T T E L S T Q G I Q F L K M I F D K

17641 tttgacagttacatgctgttgtgtttcagGGTACAAACTTCCTTGGGCTCTACAACAGAACTGTCCACACAAGGAATACAGTTCCTCAAGATGATATTTGATAAG

318 Y D D

17746 TATGATGATgtaagtaggacccatctttgctaaatagctgtatatgtatgtgagaggactgtttaatagtaaacagtttcgttttattttaatgacatgcttttt

17851 atttcttgaattgcccaggaagatgttgattaattacatgtaattcaggtttgtgtgttaaagaattgttaactccatttacagttgctacatgtattaagtatg

321 D R D G C L S P T E L Q N L F S T C P V M P W G S D V N N

17956 taatctgtattgattgaagGATCGAGACGGTTGCCTATCTCCCACGGAGCTCCAGAATCTCTTTAGTACATGTCCTGTAATGCCATGGGGATCCGACGTCAACAA

350 T V C T N H N S W I T L Q G Y L A Q W A

18061 CACTGTGTGCACAAACCACAACAGCTGGATCACACTGCAGGGCTACCTTGCTCAGTGGGCgtaagtgtcagcagtggatataagcattattgattttaaaactga

18166 tccttttttaagtcaatgcgtgttaatggatggtgtgaggtgtgtgttgggtctgtattgttccacccatgcttattctctcggtattgctccgtgtgaatctct

18271 tgctggcacactcagtgtaatcttataaagaacactataggaaaggtgccactgtttacatgtctttattcctagcctggggtaatctgtaaacctgttttctcc

18376 gacagatatcacatacaagtcccagtttaagctggtttgattggggttttttggcaaatcagtagctgattacatatttgctgattccattctatgcaattagaa

18481 cagaaaagaaaatgtgtttccctggcatgattgagattcccttgtcatatttctccatcctagaaatgttgtgcttccactagccgcatcccacagctgccagat

18586 tttcagcttggcctttttaacatacaggcatactttggcccaatttacaacataactttgaaaatcaatactaaccatcaaaatagccccctttttttgcagcat

18691 caatctctttgatttgacataagcatgtccaccatatttgttttgagtagccagatttgattctcaggagcagtcaattgtttttagattcacaagaccgatttt

18796 ttttaacctattatattatgagtgtcaactagccctggactagtgtctggaaaaaaaatgtgcatctgttgcttccctcttgtacttaaattactgcagtcgtat

370 L T T L L D V P R T V E N L A Y L G Y H Y

18901 tttttatgtaaaatatgaactttttttcatttttatttcagATTAACAACACTACTAGACGTGCCGAGGACAGTGGAGAATTTAGCATACTTAGGTTACCATTAT

391 H Q E S Q L S A I T V

19006 CATCAGGAAAGTCAACTCTCTGCAATAACAGgttagttgtggtgttcatcttgaccagaaagatcaaagataaagataaatattattgatgtaggtgttcattat

19111 tggtcatactgttcaatatttagctcacctggtccgaaggaccatagcatccttggttgaaggggaaccaattttgtataaaccttgggtctggcccgccaggga

19216 cctgaggggcagggcccaataggggaaacatagcaacttattctttaaaatccttcttcttctgtagaaatgaaaggatttgttacataatggtgtggaacatcc

19321 ttgggtaaaggggaaccaattttgtataaacagtgggtctggccccccagggaattgaggggcagggcccaataggggaaacatagcaacttattctttaaaatc

19426 cttcttcttctgtaggaatgaaaggatttgttccataatagtgtggaacatccttgggtaaaggggaaccaattttgtataaacagtggggctggtccccctggg

19531 ggctgggcccaaaagggaaaataaagcaaattttttaatatccttcttcttctgtaggaatgaaaggatttgttccatatttggtgtggagcatccttgggtaaa

19636 ggggaaccaattttgtataaacagtgggcgcctcccttgggacctgaggggcggggcccaatagaggaaatagaagtaaaacctttataatccttcttctaccat

19741 aaggattaagggattttatcctaatttagtctggggcatccttgtgcaaaagggactcaataaaaagtccattttttttttttttgcaactgtggttcagccaat

19846 tacaaatatgatattgctgttaaatttaagtacatgtacatataattattataatatcccataaccattccctttcaaggattttcacgaaaaattgtgcatttc

402 T R D K K I D L D K K Q T S R N V F R C Y V L G T K G V

19951 ctttctttttttccccacagTAACTAGAGACAAAAAAATAGACCTAGACAAAAAGCAAACCAGCAGAAATGTGTTCAGGTGTTATGTCCTGGGAACGAAAGGTGT

430 G K

20056 TGGCAAGgtaagctacacgtctaacatcttactgaaggcttttacagttgttacactccagtttaatttattccaagcaccaaaaacaggtatcaatttatataa

20161 taagaaagctattatttctttggaagttttctgtacaaggaataaaagatatttgagctgacattttacaaactttgatatcagatatgtttaatgctgcagaat

20266 tttcttgttacatttgaaatattcaccaaacagtaatgttaagacttgtttacacaacatgaaatgtttaagatgtttgtcataaatatatctatttttgcctct

432 S T F L Q G H L G R N L R Y I A T L N K E H L S S F T I N T V

20371 attttatttcagAGTACTTTCTTACAAGGACATCTAGGTAGAAATCTGAGGTATATAGCGACACTGAATAAGGAACATTTGTCCAGTTTTACAATTAATACAGTT

463 Q V Y G Q E K Y L L

20476 CAAGTTTACGGACAAGAAAAATATTTACTGgtaagtaccatctaacggtaagatcagaaactgatataaataggctcaaatgtacctttttttttaaacattttt

20581 aataattttaatattttagacttaaaatatctaatagaatatctgaaataattcattttaatttactagtggatgtttcgtttaatatgcagctaatgcactcgg

20686 taggttttgaaatacttaccttatccattcctttttgttagtgtaaatgtaagtgcaatacataccagtgaagaatatgtacttcaatagcaggacgagtctgtg

20791 taaaacatgtttatgaagtcaccccaacagtcgctagttatgatgggtcttttactgagtttgaacataatgatttctatattgaattactgactatctaccata

20896 catgttaatgttactaacacatgctagtctagagtgtcactgtgatgatggccaacagtaggtgggtacttgtgtggaaggagaaatcatacagaggaacaaaag

21001 cgatacgtgtttaagaatttcttaaaagtattgtgctacatgagaaagcaggttttttgggatatttgtgaaattgtaaggaagactgtgatttacatatctgat

21106 attcaaataaatagaaaaatagaattttgcttagagaggtcgaactttaaaatgttttttcataaaatcatccttcattgttgataatgtttgtatatgttttat

473 L H E V D V S V C D M L N P T E M N C D V A C L V Y D S T N P R S F D

21211 agCTACATGAGGTGGACGTGTCGGTATGTGACATGTTGAACCCTACTGAGATGAATTGTGATGTGGCTTGTTTAGTATATGATTCCACCAACCCCCGCAGCTTTG

508 F C A R M Y L

21316 ACTTCTGTGCTAGAATGTACCTGgtaagtacagaatgggatcagtttagaatctaaaatctccgttataacttatactcagttttgaacggattaacctaaaaca

21421 gatttaatataaaatttaatgttgttagatgcatctgatttcattcaatattttttgctttcattaatcaaaaatacttatttgaaacattattttccagacata

21526 gcagtcctgcttaatacagttaaaattctacctactgtccaaataatctgtctactcaaaattcatcttcattgatgacgtgttgttatgaaaactgatgttata

515 K H F L D S R I P T L V

21631 gatttgattaatcaaatctttaaaatctggtaagtaatgactatttgaagaccttatgttgacttgtagAAACACTTCCTGGACAGCCGAATTCCTACCCTGGTG

527 V A A K T E Y Q A V R Q D Y E L T P A Q F C S K F K L P P P Q S F S A

21736 GTCGCGGCTAAGACAGAATACCAGGCTGTGCGCCAGGACTATGAACTCACACCTGCCCAGTTCTGTAGCAAGTTCAAGCTCCCACCACCTCAGAGCTTCTCTGCC

562 I D K V N R E V Y I K L A T M A A Y P

21841 ATTGACAAAGTGAACCGGGAGGTGTACATAAAGCTAGCCACCATGGCTGCCTACCCgtatgtatctgtcttactattcctggctcaactcctatcctttgataac

21946 actgcagtcacaccacagacaactgtgttccttgcactgttcaacaattattattttctattttaaagtggcatacccaatttattttgtacttgaagtggaaat

22051 tctaacaagaaacaagctagctttgccaaaatcctcaccagatcagtaggtttcagcctaatttgtgaatgaacactggttcaactggaccattaatttggaatg

22156 tgatcaatgaacatgtgctgttatatataactttgtaacatgtgctgttatatataactttgtaacatgtgctgttatatataactttgtaacatgtggtgatgt

22261 atgttatgtctggatacgatttgataccagaaggttaccatgttgtgttttactctcattttcattcattcctttttcataatttgtcatgtttatttttcatcg

22366 tttcgttgcttcatgcaaaaagccaatatatttcaatccttattactccttagaaaaatggcttttgatgttttaaattgatgaatatttatcttttttgtgtgt

22471 gttttatttatgtttaaagacctttttaggcctgaccaattttcctcaagatattttgaaatatgatattatacccaggtcttgaatttatagtcttattaattg

22576 ttgatgaacaatttgttaaccttttggaggtattgagtacttattgaagcaattccatattaatcatccgtcacaaagcacttccttatgcatcctcattcaaat

22681 ttcactacaatcacactatatctgaattttaaagctagttttgtatttgatgacatactttttactctgggttacattcatagtgtgacctttttattttgtgac

22786 accagtctgtcagtctattagtcttgtattattatcagaccaatatatgttagaaacccatgttatgtgagtgtttctctacagctggtagtatactctgtagtt

22891 agaagaggttatcggttctccgatttcctgttaacttctggactacggctgagagtaaagtagtttataggttaatattattggccagttctccgatttcctgta

22996 attataagcttctcaactatggctgagagaaaagtagtttgtaggtaaatagcattggccagttgtccgatttccaatgaacatctggactacggctgagagaaa

23101 agtagtttgtaggtaaatagcattggccagttgtccgatttcctcataactcctggactatggctgcgggtatattatatagtttgtaggtatatagtcttggcc

23206 agttgtccgatttcctgttagcttctagactacggctgagggtatattaccggtatatagtttgtaggtatatagtactggccagttattcattttcctgtttac

23311 ctctgaactacgactgagggtatattaccggtatatagtttgtaggtatatagtttatttgatttcctgttaacctctgaactacgactgagggtatattaccgg

23416 tatatagtttgaaggtatatagtttattcgatttcctgttaacctctgaactatgacagagggtatattatatagtttgtaggttaacagtagtgtccagttgtc

23521 cgatttcctgtgagcttctggactatggctgagagtaaagtagtttgtaggttcatagtattggccagttgtccaatttcctgttagcttctggactatggctga

23626 gagtaaagtagtttgtaggttcatagtattggccagttgtctgatttcctgttaactcctggactatgactgaagtttaagttgtttgtaggttacaataggtac

23731 cagttttcccgacaaacttttgctctaaaaggattctttaataggtctaactctggcctttgtagtttgtgagtatgttactaaactactaaaaaggagttgttt

23836 ttggtgaagttgtgttattttgtacaccaacatattattgaaataaaagtttcaataagtttgggttataagattgctatcacggaagttcatgtattgcctgat

581 N L K R L V H M L L V R Q N

23941 atatataattccttaactttagtagtttagtgtactttgactcgccccttgattattttcagCAATTTGAAGCGCCTGGTACACATGTTGTTGGTGCGGCAGAAC

595 P L W L E E K F S

24046 CCACTGTGGCTGGAGGAGAAATTCAGgtcccctatcgttctttgtgtttatattgtacctattttatgtagcatgcctgcttgttctgtcttactgccctccatg

24151 taaccaccaggtgttgtgtctgttgtatctatgggataggaaatcagatttctatatatgtatacatatgtactcggtaaatattaacccaggttacactgctgt

24256 gttctctctgagaaacaagtgactataaagcatgcatttataaaacaagtccaattgactgtaaaaggggaaattcttggtttttgaaatattccttttatgact

24361 ctttaaactgatcatgtgcacggtgtggacattttcatattggcaatggttttaaaacatatttatatattactgtgtggatatttttgtgttaattcactcaac

24466 atggaaatcgtgagtatttccacactatgaaaacttcaacttttacggttctccaaggtgattcataagaatagcgattttgaagcgaattttaaatcctgagtt

24571 tgttataaaaattatcttcaaagggaagtaatattatacaataattaatttctaaattactttgtttctaaaaaaagacccagaagcttttaaaaactgagtatc

24676 ttgttatttgactgttcagtttgtcacttgacatctacagagaacactgtatactaaaacaagatagcccagaccatgcatccagtcacttaactcattagaaat

24781 tcccttttgtttatttataactttattcttaacattgtagatgtagaacgttgatgtaagtcatagcaatactactcaaattttgattttacgctgggacattga

24886 gaaatcggaggataatgtatcttccatttcaaacatgttcacatattcactgtatctctattctttatcacaagggtggagttgtatactaattaagaatggtga

24991 ttatcatgttgtaataacttccctccttaaagtacagcatcagggtcaggaagcttcattggtatactaatgtattaaacatttaaaaaaatcatttatttttgt

25096 aaaatgtttgaaaattttgataaatttgtgtttattatcagtaatttaaaatgtgaaatttctttatagaaaatgtatagattgatattttccatcaatgtgtta

25201 ttatgaaggatatgagacttgatatagagctggttaaacaagaacatatacttgtctgtgattagaaagggcagggagtagaggccataagcaataactttaaag

25306 aattaacaatagataagaaagaatatgtcccgtcgaaatatccttattgtggtgcagccttttccagtaaaggtatgttcatatatagggttgtaatctagaata

25411 ctgaaacttttcatttactgttatgtacatgttttaatccaggagtaaatcaacttttgtttacaatatatgtgatgcatgcttttaatattgcagtgttagaac

25516 ctagcatgactcttttttccagttttgatcagctgttattttttccaatttctttctgcataataccacagaattagtatctaagtccatgataatacattttat

25621 ctaaagaaaatgatctattatgcaaaaaacccacaaaatattacaatttaaaactgcattcttttactgtactttagttttggattcattcataatgtgcagtta

25726 agtgtttgattgtgcgcactgctaggtcattagttgtgagtctgtggccatttataaggctttacctgtaacaggtctgtagttaacttttgatttttaaagtaa

25831 tgcacttacctacctatatttgacactttctgttgatcaccaacattataactactgcaccagaatgagtaaagtgaagctgtgaaacctgtctaaacaggataa

25936 atcagaacttcagcgtaatcccttgctatacctttctatgtaaacttttgtaatcagaaatcatgtctgttctgaataaaactacagtttattggtgcttttagt

26041 tccttttttggtggacattttgtgctgtggaaatagtttgtaaacttaatgtttgcttaatctgacaccatagaattacaaacacgaataattagacacattctg

26146 aaataggtttagcagaagcactgccattgtgtacatttccacaccacatatactaataccatgttatactacaggtctgcttttacagtggtcatattcccaagt

26251 tcaaatgaagacaaataattgaatgtaatgtgcaccttgatttgcttacatctagatgtatcttgctgcatggatggaaacagtcttaggatgatacactgttca

26356 gtaaactgattgaccagacctacttgtctgtgcttgtgacctccttatacagatatatgtgtaatgtctcctgaaatgaaaactacatgtagtctctgtaatatg

26461 gtgatctataatttttaagcttgttgatatacttcatatacttagtaccacattcaatttcaagcccaaaaggccaatgacatgaaataattcatcctactttga

26566 taaactcccatatttggtaaacaaacattaccatcatttgacatactacattgtatgatggtttgatactttagcgtacatgatctttaggttactatttctata

26671 aacagaattgagtttgaagataattgtaaatgctatgtactgttagatttccaatgttgatgtgtttttgccttgtgtatttacactgttagatttccaatgttg

604 H M K G V V M K E E N G Y L R V G I G V A V L A S V

26776 atgtgtttttgccttgtatatttacagTCATATGAAGGGTGTTGTGATGAAGGAGGAGAATGGGTATCTACGTGTAGGGATCGGTGTGGCTGTACTTGCCAGCGT

630 G F V L Y R I L R R G S *

26881 GGGATTTGTGCTCTACAGGATTTTACGACGGGGATCATAGtcactgccacacttaaatatagtttatggacgaatgcaggtgcatgttgtaacgccccctagtgg

26986 acaagaggaagtttgtgagggtgtttcacacaagtcatggagctcaccatagaatataggaataacagaatgtagattgacttgatagatagacatggatttgat

27091 atttaggcattgaatgcgaacgtttgaatatctgcagtcgtaggtgtgatctgatcaaatatccatgtgaatgtctaatctgctgacatttttcctattaaatgt

27196 gaatgtctgaataccctcagacatagcattgatgttgaatgtgaacatcttgataactgtggacatatgtttgatatttagatattgaatgtaaatgtctgagcg

27301 atgacccctgtggagaacagagtactgctgagagcttcctgactagtcctatgtgatgtcaatccctggtgtagctaatagcaccagctgtcagcatggtaatcg

27406 tctcccttgaaaactgccagtatcaagatttacagggaatatatatatacatcgtcatacatgctttgtgcatgttaaattaagttaaattaagtctaagttctt

27511 gagatatagcacaattttcgtaaatatttattgaggggaacaagttctggaaacttttagttacacagatcatatccattcatgtacaaagtgtagaagacaatt

27616 caagtttatgtcttattgcatgtgtgctgtataagatacagtcaactttttggatagttaaaattctagacattatcagtttccaactgtttaatggagagagaa

27721 ataattcttaaactagactttttataatcaagtttgtgtagaactataaattattcgtttgtgagagtttcagtgtgtcctataccagaccatgtgtgtattgag

27826 aggtagtgaatatgaaggctagctagggaaacacaatgtgattctttaataaattaaacacaagatactttgcttttttttcataatttcataatgtgggaaaag

27931 accttacatatcacattaattcatcattataaacacccatatttacataagtcagaggtgtcggtccaagtttcaaactttaaggacagagtaatttcatttacc

28036 tctgtatctcctgttggaaacaatcagttgatgtgtattacctaatacatgtaatagtaagtaagtaagacattaaagacattgtttctggtttgagaatagtgt

28141 gacagttaattaagattgtgtgtaagtcaaccagatgtttgagcctgtaggataagtatttaaatttttctgtattgctttaatttgaaaaaaaatcatttgatc

28246 aatttattaatttagcaaaaacaaaaaggtcctcattggttttaaggtgtgaaatgggattatggataaggtcaatggcttttgtgtatgtcagtcatgtttgta

28351 agtgtgcagcacgatttgtaatataccattaaccaagatcaaggacactatatgtgctttctgtcagtactctatgtttatgtttttaacatataaggcatgcat

28456 gtgtgttgttgggacttgttaatctaaaatcaggtgctggacacacaaaatcgtattatgcatatacatgtgtgtatataatatttatatatacacgtataaaca

28561 ttaagtattacagaaattatatagtacagctatctacacattatgcatatttgtatgtgtgtccagcacctgtatataagatacatactcttgtgtaagagcaat

28666 tgtaagcttttgttccttgcaatttatttgaaaattacttaatgcttaactgtttaaaactattgttacatgtatgagacatattttgtcctccccataaatgat

28771 tttctcccctgtcaacacataaattattagattatctcccctttggtaattcataaatgctattctagtactggaatattaaaatcaaatccgattacaatgctg

28876 caaacaaaacatgaattattttatcagaatacataagtatgctgccagtatgctttaataaacacaaaaaggtgtagacagctggtgtgtagactggtgtgtgta

28981 cagcttgaacgggtcatgtacgagattatacaatgtgcaatctagtcttaagagcctaagagtcatatcttttattaacatcagagtggatggttgtatggatta

29086 tgaaataaataaaatggatacttcatgaaatgataaatatacttgaaagttaaaaacaagggcattcagacatgtgtctgtattcagacatgtgtctgcattcag

29191 acatgtatctgcattcagacatgtatctgttttcagacgtgtatctgtaatcagacatgtatctgtattcagacatgtatctgtattcgaaatgtatctgtgtta

29296 gtaagttcgaattctaaatatatgtaaaactcatgatgtttcatatttacctgaaactgcatacaactttttaaaatgtgattttatttatccaaatcacaaatc

29401 aaattgtattattggagaatcatgtgtttataataagtgtttttcattgttttctatttatttgtatagattttcgaccaaatggtggcagtaaaacaaggttta

29506 actttgtaagactggtatgatgttttaactgatataccatattaacaggaatagtgagagaaagaaatattaatggtatacagaatattgtgtcttagatagagg

29611 cgctataccaaagtatcatgagttctagaagaacctcaataaattttcgaatcaacttttaaacttgcattatgtaagaataaaataatgttgatgctctgatta

29716 tgcgagcttaaatttgctcgccattaatgaatcaaaatttgttgcatggttttgatgtccagttcagtcctttactatttatgtgggtctttggtatggtacagc

29821 tcaattaattttcaaaatttaagtattttacaatgtttgtcatgtacttacaaaacacctagttaggagttggatgttattgacaggaatgttgttataaatgta

29926 taaaacatggatgtttagtaaatagtaagttatactcatttatcattaactggaaaatgtttcaattctcctttttttcatgaaaaacagtcactaaagcatcta

30031 aagatcttgcaaacaaagtaatattttatgtagaaacatattgtaacatttttctggatacataggattgtaaccccataggccatatactcggctataaaatct

30136 tttcagttcttaacatcaatttctgtgctattctattttataacgatgcagtgcaactgttactacatacagttttcttgtatacaatctaactacatacaatgt

30241 gtaagcatacaatgttattacatacagtgttactacattcagtgttcctgcatactatgttgctacatacaatgttttagcatataatgttaccacattcagtgt

30346 tcttgcatataatgttactacatacagttcttgcatacaatgtcactacattcagtgttccagcatataatgttactaaatacagtgttccagcatacaatgtta

30451 ctacattcagtgtacctgcttacaatgttttatcatacaatcttactgtatgcatacaatgctgtattgatgcctcatacatcatgatgtcataagtcacttgtt

30556 actcaatcactacaatcacattctcactttttaaaggggtacagtgtgagtcaaatgtaatggtacaaatccatgaatatatgcatggtttgatagatgtacaca

30661 gctactcaatactcatcatgtaactaaggcttgcctcctctcttataactatatactctgtgttgattaaaatgtttatcagtctgaaaccttaggaacacaatg

30766 aaactggtgtgttatatttatggtggtttatacaa

**PyRac**

1 cttggaggttgcatttgtataatactatttatatggttaactggtatagagatgatactcagcaaataaaaataagagttgatacatataaattataaaagacga

106 ttcatggcaaacaaatgtaagtttaaatagttatgacacatttatcattttcgaaaaacggggggtaaaaaaaaagaggaattatattgtactagcatgcacaag

211 caagtgggttcagacccaccagccttctaccagtagttacatagtagtatatatataggtcttacatgatagagttttagtaggtcttttagactgctgatcatg

316 agtgctgcattgatttttacccagtcgggaaaaaaagttctacctttgccaaactgaatcatcaattataatttactattttttgtcaatcaatgcatcctccct

421 acttggtcaacttgttaagttatactaattttacaagttttctttataacttctaagtgctatagttataacagtggcaatgatcatgatgttggacagtgggag

526 tgactaaggtgatcactaacattataaacatagcaatgcatgtccctttgcagatgaatataagtgtatttattatatttgtataaacaataacgagttcttact

631 aaactggggattatttggtgtcctgtaggcgttgtattttgcatctaaggcaagtgcttgttgccagactgctgccatgtttacattttggaagaaaaggtcgct

736 tagaccatagcacaatggggtttctaaaagtttgactattcggctaataatatccggataaaaaaatcatgtagcaagaacaattatgcccctatagctatacac

841 atgaaaacgaatatcatgtgaatctgaattatttttgtttaaaatatgatacgaccaataaattataagagttagaactgttttatttgaaaagttggaagttat

946 tgcattgtttcgaaaataccatataatgaaattatagaaatttgagttgcaatgttttctattatatttttggccattcttgatgtgttcatataacgtaatgga

1051 tgaaaatgtccatgtcattcgcttattaacaggtgcaggaatagacattccaaaaataacttgattaaaacgtctttaaatagtaacatcatggatctttaaaat

1156 gtttgtgttaacgtagtcgaaacttaaagcgtcgagatgatgcacgcaccggtgtcgcctgcatatattacaactcggctaagatcgtgaatcaccggagatact

1261 ataaatagttcacgtttaatcgttcctatataagaggcgacagccatattttctataggtattaaactgggagaggtcggcccataataaggccgtgatacttga

1 M Q A I K C V V V G D G

1366 catcaggattggacgttgaataaccattgtgtacaacagaatATGCAGGCCATCAAATGTGTTGTAGTGGGAGACGGgtgagttaaaatggactataaatctaac

1471 taaacggtgtaactttttctcttccgtctgtcatgagctaaaaaatgcactggcctcaaaaatgctctggactaactcaaaacgatttagtatgtataactcata

1576 ttaggcctaacactgattaagctgaaacatttcatatgatttgaagtaagtatcatagttccgcatttgaatatgggataaaggatatttgagacaaattggact

1681 gattttgttccagtgtgaactttgccaagtttatggacaatagtcactcttagtaatcacttcctgtcacttgtgtgtggcttaggactgagtaagtctaatgac

1786 acgagcatcttttctgtgtcttagcattgaccagagtgagactacagtgatgtggggattccagcatcaaggaagtagttgtttatagttgaggattgataatgg

1891 ggtcaatagatgactaagtcagaatcaaaagccctccccagtggtgcatgtctattactcatgtatttaacacatgtgttctgttcacacaagtgtgatgagcta

1996 tagtttccacacttgaccagtacatagttaatataatgccattcagagatacattagatttgtgtgttgctaaaattctagagctcttccagttgaatatttata

2101 gaaatgtttcatggtttaacttttccacagcaattgttttgataatgatatattgtgctataaatacttcaatgcttttgtgtgttgtatgctttggtgaaacgt

2206 tccaatttaaaaaaaatcttatccaagcatttggtgaccgctacatgtattacttattaaataagaaatcaggctatgatgaatataaactattgtaacaggaat

2311 gagaatagagtgcttccactgggggtcaaaccggccatccccaggttcctagtcggatgctttaccactaagccaaaggaaaattccctctagctcgaagctaga

2416 attccgaaggtgaccgtaatcctatgtatgtaattaaaatctgctacactactccccctttttagatgtgttcgccctcgaacatcacaacccatgttctaactc

2521 cctgtcaccacagggagggccacggacaccacttgtaataggaaagataatagtgtgcttccacctaggatcgaacctgcgatccccaggttcctagtcggaagc

2626 tctaccactgagctaaagagaaattccctctaggtcaaagctagaaggcgaccttaatacaatttaaatctgccacactgttatttataatcaacagggtatatg

2731 ttagaggtattgacttcaggaattccaatatcattccttttgtttatcataggtaataattaatgcataggaaattattatcaataacacaataatgtaggggct

2836 attttttcataagtttaaagtatatgtacatttgataaaattgaagatgtttcaaattttgtatattttaacatatactgtgaatgtgaattattcatcacagta

2941 aaaaggggtgaaattatcataacagtgattatgaatttattattatatattcagtttgtagatattctttaatataaatcaggtttatgaccaattataaatttt

13 A V G K T C L L I S Y T T N A F P G E Y I P T V

3046 ttatgtttcagAGCTGTTGGTAAAACATGTCTCCTGATCAGTTACACAACAAATGCCTTCCCGGGAGAGTACATCCCCACAGTgtaagtattccacacttgatcc

3151 cagttcatataatatcctcccaggagagttcatccccacagtgaaagtattccacacttgatcccagttcatataatcttctcccgggagagtacatccccacag

3256 tgtaagtattccacactaaatccaagttcatccccacagtgaaagtattcaacactaaatcccagttcatataatatcctcccaggagagttcatccccacagtg

3361 aaagtattccacacttgatcccagttcatattatatcctcccaggagagtacatccccacagtgtaagtattccacacttgatcccagttcatataatatcctcc

3466 caggagagttcatccccacagtgaaagtattccacacttgatcccagttcatataatatcctcccgttaagtacatccccacagtgaaagtattccacacttaat

3571 ccaagttcatagaatatcctcccgggagagaacatccccacagtgaaagtattccacacttaatccaagttcatagaatatcctcccgggagagaacatccccac

3676 agtgaaagtattccacacttaatccaagctcatataatatcctcccgggagagtacatccccacagtgaaagtattccgcactaaatgattaatcccagttcata

3781 taatatcctcccaggagagttcatccccacagtgaaagtattccacacttgatcccagttcatataatcttctcccgggagagtacatccccacagtgtaagtat

3886 tcctcacttcatccccgttcatataatatcctcccgggagagtacatccccacagtgaaagtattcctcactaaatgattaatcccagttcatataatatactcc

3991 caggagagtacatccccacagtgaaagtattccatacttaatcccagttcatagaatatcctcccgggagaggacatccccacagtgaaagtattccacacttga

4096 tcccagttcatataatatcctcccaggagagttcatccccacagtgaaagtattccacacttgatcccagttcatattatatcctcccaggagagtacatcccca

4201 cagtgtaagtattccacacttgatcccagttcatataatatcctcccaggagagaacatgcccacagtgtaagtattccacactaaatcccagttcatagaatat

4306 cctcccaggagagtacatccccacagtgtaagtattccacactaaatcccagttcatagaatatcctcccaggagagtacatccccacagtgaaagtattccata

4411 cttaatccagttcatataatatcctcccgggagagtacatccccacagtgaaagtattcctcactaaatgattaatcccagttcatataatatcctcccaggaga

4516 gtacatccccacagtgaaagtattccacactaaatcctagttcatgtaatatcctcccaggagagtacatccccacagtgtaagtattccatactaaatcccagt

4621 tcatgtaatatcctcacaggagagtacatccccacagtggaagtattccatactaaatcccagttcatgtaatatcctcccaggagagtacatccccacagtgaa

4726 agtattccacactaaatcccagttcatgtaatatcctcccaggagagtacatccccatagtgtaagtattccatactaaatcccagttcatgtaatatcctccca

4831 ggagagtacatccccacagtgaaagaattccacactaaatcctagttcatgtaatatcctcacaagagagtacatccccacagtgtaagtattccacactaaatc

4936 ctagttcatccccacagtgaaagtattcaacactaaatcccagttcatagaatatcctcccaggagaacatccccacagtgaaagtattccacacttaatcccag

5041 ttcatataatatcctcccaggagagtacatccccacagtgtaagtattccacactaaatcgtagttcatccccacagtgaaagtattcaacactaaatcccagtt

5146 catagaatattctcccaggagaacatccccacagtgaaagtattccacactaaatcctagttcatgtaatatcctcacaatagagtacatccccacagtgaaagg

5251 attgcacactaaatcccagttcatataatatcctcccaggagagtacatccccacagtgaaagtattccacactttatcccagttcatataatatcctcctggga

5356 gagtacatccccacagtgaaagtattccatacttaatcccagttcatataatatcctcccgggagagtacatccccacagtgaaagtattccacactaaatccta

5461 gttcatccccacagtgaaagtattcaacactaaatcccagttcacagaatatcctcccaggagaacatccccacagtgaaagtattccataattaatcccagttc

5566 atataatatcctcccggcagagtacatccccacagtgtaagtattccacactaaatcctagttcatccccacagtgaaagtattcaacactaaatcccagttcat

5671 agaatatcctcccaggagagtacatccccacatgtcctttagatgttgtccagacactggaatataatgtttacatgatgtagataatgtctattacttcaaata

5776 gttcaacctatttgtctgtgctggtttatgataattatgatagttgaaattattttttatttaattggagagcaatctttaaagtcatgatcatgatttataaaa

5881 cagcttattaataaaactctttgatgcctttaaattgaacaaaattaatacaagaatgtaaagcttaatatatagtatgtatctatatttgtttgtcatcagtag

5986 ccatgcttaaacaactccatcacaaatactaacttcatcaaagacatgtctcctacataatcagaaactatttcatcaaaaacacacattcctgaatatatggct

6091 gtctggtacgtacacatgtacaatgtattgtgtttcacagcaatataatatacaataacaatataaaacatttgatatagcaccctatatctagctaacataagc

6196 cattctaaagcacagtacatagatatacaattaatacaactagcatacatatatacattaatcaatacaatttttgaggaattcacacaggagctgccttatcag

6301 gcgctaactactagtcctatgccacctggcttatatctccctgtatgtgtgtgctggagatgtattgtgttctgtttgtagtgggaccatgcttccaaaggtatc

6406 attaatatttgcaatataatttcactgtgaagtcgagagttagagctcttttcaagaaaacacatacaattattggaaatcaaagtgtgaaatgttttcagtatg

6511 agtagaagcaactattaattaaaaacttgatattgcattattgtgacactgggttcggttcctgtctgtcctggccacagtgataaatatattccatactaggat

6616 atttatgtacaagttagtcatatttcagagagatggttgttagtagaacactatatatttttgccttgtactttaccccattacctggatactaatcactacctg

6721 atttcagtacagattcagtgaaccatgagttatgatggttgttgggctcgttatttaatggaataagtcaggtgttatgtaaacttcctggacgtttcactgaac

6826 aatacagggtcacatatatatacttgtgccagtttaccagagatgtgaggtttccaacatgtgttatatttatacattatactgtgccagtaatgcttactgtat

6931 tgttgtgtgtattctggttcaaattatatttaatatttttgtgaagatgaagtaaccggtgtcaaatataacacactgtagtcaatgtatacagatttcatattc

7036 aaagcaattcagttttagttctatttgtacataaatgtatcaaatatgaagggaaactgattaacattattgttctgtattaattgtgaagtgttcttataggaa

7141 ccattttcgtttgttggactatacatataaaacccatgcatatcttgaaacatatatacatatatgtacatgatattgtatactagataaattggacctgaaatt

7246 tacttcctgtttttatcagtttgtgtcgcacatccgagatgattgtagcaatgatgagctctgattgcgattaagttaaaatactatgcaagaagctctgattgc

7351 gattaagttaaaatactacaagtaggaccaacagatttaatttactgctttctagattgagtgtgcctatatatctagctactacctataggactatgtaggaca

7456 aacagctggactgctttctagattgagggactatgtatcatggtaggaccaacagatttactgctttctagattgagtgtgcctatatagctactacctatagga

7561 ctatgtaggaccaactgatttactgctttctagattgagtgtgcctataaagctactacctataggactatgtaggaccaacagctttaatttactgctttctag

7666 attgaatgcctatatagctactacctataagactatgaaggaccaacagatttactgttttctagattgagtgcctatatagctaccacctataggactatgggg

7771 aacaacagattaactgttttctagattgagtgcctatatagctactacctataggactatgtaggaccaacagatttactgttttctagattgagtgcctatata

7876 gctactacctataagactatgaaggacaaacagatttactgttttctagattgagtgcctatatagctactacctataggactatgtaggaccaacagatttatt

7981 gttttctagattgagtgcctatatagctactacctataggactaagtaggaccaacagatttactgttttctagatggagtgcctatatagctaccccctatagg

8086 actatggggaacaacagatttactgttttctagattgagtatgcctatatagctactacctataggacaatgtaggaccaacagatttactgttttctagattga

8191 gtgtctataaagctactacctataggactatgtaggaccaacagatttactgttttctagatggagtgcctatatagctaccacctataggactatggggaacaa

8296 cagatttactgttttctagattgagtgcctataaagctactacctataggactatgtaggaccaacagatttgctgctttcttgattgagtgtgcctatatagct

8401 agactacctatagaactatgtaggaccaacagatttactgttttctagattgagtgtgctctactgtaacatgaccatatatgtggtccaatattgattgtgctt

8506 tttctgaaagcttaagtttgacaattccaattgtaatgtgctcattctgaagacttagctgtatgataatagtattactaaatacgactatacaatccatgactt

8611 agctgtatgataataatattactaaatacgactatacagtccttgacttagctgtatgataatagtattacgactatacaatccatcaggaagtttatctattga

8716 tatacgtattaacaagtacacaaagattgaagtatttacacaaagacatgttgtagataataatctgttcaagtcttcatggtacacatgacttgtggtaataat

8821 gggttttttggggggttttatgtatatatatcgtaattatacataactttatttagatgtactactgaaataactagtacatgtatatatgtatacgtactacag

8926 tgcatatgtagaaacaaagacatatgtatatttttttgataaaaatgtattcactaatatgttgacattaaataaaatgaatcaaataaatgtgactggtcatct

9031 atttatgattttaatcaatctaaatagttagaatattctaactggacggagatttgaaattgcttatttgagtgaatgtgtgtcaaaatgtaatgcagtgtgcac

9136 attgtagtatgaattgtaaaaaaaagacctattgacacaatctacctacatgtacattgtttccagcctttcacctctgtcaatgctctccagggggtcaatgct

9241 ttatttggggaaatactcattttgacaggacaacaacacaatgtagaaaatgatgatgtcatacttacaatgcataatgaaataatgtacccacaaacaatgatc

9346 gattatgtcaaaataaacaaaaagctgtaacacacctgtccataccctcaatgacaaggtcacagtaacatacagatcaaacctctgatcaacaccatgtttgtc

9451 cggaccatactcaactgttaaattttattgataaaatttcagagatataacagctggtatataaattacaagtcatataactttagcatcaaggtcatagcaaca

9556 aatcaaagattaattctctgatagactcgactaattaaagaaacctataataatatcccaaatactggttattgtgataatatcattgtcaacaatgagatatca

9661 cagtacaagttttaaatatcagcatttaaatttttttcatacaattttttttatcttataaaaactgaaagaatctcaaataaaaagaaacttcatacctgtcct

9766 gttaacatttaaagttgattctatagttcataatgattttcttaatgatcaaacaacctacccactatcatcataattcactgatgcatgagtgtgaaaattagt

9871 cctggaatctatttataggtcttattgagaacctgcagggaaggggtagactgaccgtctggacattttagaaaatgtcacaacgataaactatcaattacaaaa

9976 aacattttaatctttataactatctctatattaaaaatatacattcagtcttaaatgatttactacttttaaaatattttttaaacaaaattctaaatcttttac

10081 cagccacagattacatatttaccatgcaatttttcaacctccctgcagcatagctgctgctttgaaaaaatggttcaaagaaccagacaaactaggagatagttt

10186 tcaaagaaatacataccaaaaccccagagtgagagcattttgaaaaagatgacaggttcttgtaaaatattgctgtgtgtaaaatatgtgtggcatgcatctgta

10291 aattattcagtgtttgaatttgtgtttatcttgttatttttggtaagagttttttcacaagtctggtcagtatatcacatagacctaaatattggatgaattata

10396 gcctgaatattcattgattcattgatgacacttggttaatgtattatgagaagacagtgtggcatatcagataatatcaaggggacagataactgcagcgattgt

10501 cttcataaatataaggattgatgctgaaagtttctaggtattgaatgtaaatgttgtgtatcacacaattctatgtgaataattaaggtcaaaggttcatgaaaa

10606 cagatttacagtgtataaagcttattagggagtatataaccactactctgttttgaaagtacatgtatactggtagtagtaatgtttggtaatataactgtactg

10711 catattaaatttttgtagccttagtttatgcatacgacattaaatgatttgtttgatgttttgatgaagcaaatttgagttcattcaaacttctaagttacacta

10816 tggggtatgcatattttatagcgctatcttctgaaacaagcttccaatcaaactaatacccacttggttatgttagaaacagctgaatctcatacagctacactc

10921 aaacacagctgcatatagtgcacagcagatcagcgataggccagcacatgcctcggtcagttatacacatctgtttagccacctcagttttgactagaaacacct

11026 tggaaaattaccatacacagtgcaatagggttttaaggtgtactttttaacagcaggccaaaggttaatacatgactgcaaatcaaatgtaccagtatacaaatt

11131 attcccttgcagatttatgcagctgttagattattgtttgtagggtaaatgtgttcaggttcttttttatttgtcaaaacaaaattaattattgagattacaatg

11236 cttttaataccttaacatttcttattactgataaatcagggtacacttatatgtacatatctgtcagcagtggtaaacagaatatcaaacttccatgctaattaa

37 F D N Y S A N V M V D G K P I N L G L W

11341 tacctctattctagccattacacgtgttgtgtgtttgtttgcagATTTGACAACTATTCTGCCAATGTAATGGTGGATGGTAAACCCATTAACTTGGGACTCTGG

57 D T A G Q E D Y D R L R P L S Y P Q T

11446 GATACAGCAGGACAAGAGGACTACGACCGACTACGGCCATTGTCCTACCCCCAGACAgtaagtcatagaacactttttactatgcatgaatttaattacattttt

11551 aactttttcaatacatttctgtatcagttatagttactgggtcaatatacattgattactatagccaatgttttccttagcatggtatttcattgagtgcctacc

11656 ctagtttttgttaagaagtggatgtttactacataaattcaactatgattcattcctatccaactgtgtttgattgtaattcatatttgaaattgtttacttgaa

76 D V F L I C F S L I S P A S F E N V R A K

11761 atttaaatcgcattaaaaatattgttcaccttactttttgcagGATGTCTTTCTGATCTGTTTCTCCCTTATAAGTCCAGCAAGTTTTGAAAATGTGCGAGCAAA

97

11866 Ggtaagttaaatcttattcactattaatgtttaaattcgcaactgtctaaatattgtatttgaaattgaaatctttgactgtgtttcattgactgtataaagtaa

11971 acaatatttcattaaatgtatgaagattgattgtttaagatagactgtatgttatattcctgacatgttgtatcttagtgattttccttgctatctcggatgaag

12076 aaaatttgttgtctttgattcataattttgctaaggaacaggtgtagttaatgttcaaacacataaaagatactgtgaaaacatttttgattcccagaacaataa

12181 tccatttccaaaaccttagtccctatttaagtatcctggatgtatagggggggttactctatgccaatacctggaggggaaagctgtaagtctgttttaattaaa

12286 ggtcagaggtcatcttctggtaagaaatgtatccaactttacaaacatgtattggcagacttatttcgttgaaatttccctattacagtcaaagtgtacattgac

97 W Y P E V S H H C P N T P I I L V G T

12391 aaaattggaaagatacataattgactgacaatatgtcttatgttttagTGGTACCCAGAAGTCAGTCATCACTGTCCGAACACTCCCATCATCTTGGTTGGAACA

116 K L D L R E D K E T I E K L K E K K L S P I T Y P Q G L A M A K E I S

12496 AAGCTTGACTTGAGGGAAGATAAGGAGACCATTGAGAAACTGAAAGAGAAAAAGTTGTCGCCAATCACCTACCCTCAGGGCTTGGCCATGGCCAAGGAAATCAGC

151 S V K Y L E C S A L T Q K G L K T V F D E A I R A V L C P K P K P K N

12601 TCTGTCAAGTACCTCGAATGTTCAGCTCTCACCCAGAAAGGCCTCAAAACAGTGTTTGACGAGGCCATCCGAGCTGTCTTATGTCCAAAGCCGAAGCCGAAAAAC

186 K K K C I L L *

12706 AAAAAGAAATGTATACTCCTTTAAttacataaacatttatttcaaagaattataagggagggatatcaaaacataaactgaaaacaaaatgagtttgaagaagag

12811 tgtgaacctccattgtttcataccctgttatcaaataggatacaaagttcaaggaaaggaaaccagaagatatattatggttacatggatgagcaaaatttgaaa

12916 attattaataaatttgcatcaacaacatgcctgatgtataactggttcatcagcagctgtatggtaaaacctgtataaatcctgtaccagttttatatataagat

13021 gtaatcacaagtatgagacatataatatgcagcattgtatgcaatgaaagttaacttgccttggtaggagttgaaatgtaggacttgtatgatgatgagtggaga

13126 gctctccgggattaacataacagcgttaattgtcgtacatgttgtggatctgcaaacaatgtttaagtgtccttctgtcctggctgtaagctcagctttcatata

13231 agtgtgctgctactctttgttagatcataggtttgctcacaatatttctttgaaatcgctatcttttatcagtgttctttctgtttagcaaacccattaacattt

13336 cattcatagttctgtgttttttttaaatctgcttatttcatagatgtagtttgtgctaagcacatcttattctatgatgtcttcatccagaaaaatgatttttat

13441 tggaagcttaacaacagtttgtattttagtctgaactatgttcctttggtagccatagatacaatctaaagatgtatcaattataacatgcatgattaatgacat

13546 gatatcaatttggaacacatatatcaaaaacaaatcctagatttttagaagcaaaagtattgtctttttatgaaatttatattgactggcattaattttgtatgt

13651 gattaataaaattaatttatgtcttttagcacagcttcagttgtgcccttttttatgtagtgtttgggcagttggtggcctttcatacaatatagatttgtgtgt

13756 taaagttatcccccttactgtacatcttttgttactgccacttctccctttaaagtgatgttctaaacctgcttagcatataacatttctgcttcattgttgcaa

13861 acacaaacatatttgaagaaaggggatttcgacatatattcatacttattttgattccatttattcatctgactgatgttttagatatatagatagactctctac

13966 ttgttatgtttcactttgtaaataaaattttactttttaaatttaatttttgtcctataaaatctcttgcatctcttacattatatatatgaataaacttttatt

14071 agattataaaagtacatggcgtgtaccactatatttacattgtgcatgttaaattgtggtgaagatgttagagagtgaacagcatacatatatatgaatgtggtc

14176 agaatatacttgttattatattttcaaattacttgttcatacattgaaggtcaacaatgctcattttatctaaaaaaaatccacaaaaaactgataatcgtctga

14281 tgcattgtgcgtttcttgtcgaggaaaatgcaaaaaaatacaacaataatgtaatacactttctgtttttgttattgcatgtgatattcaagtttagtttaaaca

14386 cactcactgtatttttgtttgagtaatgttctttggcacagatatattattaataacatctacacttttgggtattttgaattactttaaataatatgaattgtt

14491 ctatcgatggaacacctattatataatctatatacagacttcggacaagtacagcagtcaaagttgtaaagagccacttgaaatcttttgaaaattctcagaaaa

14596 aaaaaatgctttgcaagcattctgattttttctgtacaggtgtatgaaatgatagatacatgggaaggtttccatgaagtgagttggcattagttaacagctgct

14701 gtgtaagacccatttcagttgtttcatgtgtgctgttcagccattgttttattaaagtgcttggttttacactagctatataccgcactaaaggtgtataatgtg

14806 tttgtatagtttgtactagtgtatatgtgattataatactatatagctttataaggtttaattaaaagcaagccatgtattgtcttctttttcgtcctggagatg

14911 agttttcttttcatcgtttctgttcccaaggtatacatttctccagctgttcttccaatctttctcgtgactgtgtgtagtggtattcaaacctatgtgtctcgt

15016 acaaaatcttgttaagattgtcagtataaaaatgaattttggtggcagtttcaggtatcagaacctgacatcagttgtacttatgaatgacaaactggttttcta

15121 tcttatgctcaatgtgcgttactagaatattcaggtgttttaaagtaaatgatatactttagttctgtattatagcataaagaggggcaacctgtccacccataa

15226 cgctaaccaaggagggcgacagaaataattgtaaacctctaatgatgatgttaatggaatatattgggacatatgtactggtactgatatacagagaagttgtaa

15331 ctaatcctaatcaatgttctgtatcacatcaaggtctgctacgtttacaagggctacttcctagacttgttgctggaacagtgacagattttgatcatatatagt

15436 tggtatagagattcctaatccatctctatgttaataaaatcttagcctaacaaagtattaatttaataagagctacatcagtgttttagttgtacattttattgg

15541 tgcttgtatatgtttatggtaagagatttgcctcaactctgcagatgttgcagtcttgacaaatccaacaccctgacagtataactgctgcgtagaattacttcg

15646 ataccaacaagtcgttatcagcaacactgtattgaatcatttgtagtggcattattgtaacttactaacaagtcagcagcaatttttatgtttaccgtataccgt

15751 gaaatgttcaccgtagtttaactttccattttgtctccccctgtagacggggttaatttatcctgacagcaaaaattagtcaacaacataggtacttatttcacg

15856 tgatgagtgacattaactttaggcaaacttacaaattaatctgtgaaaggtgaaagtttaatgaggcaaaagtttcccaatatacaaagattgataatttatagt

15961 gacaaattttagctgaacatatggcactgtattaaataaattgagatttatgctttatttcttacaaatgatataattttacaccttttgcagctgttctgatga

16066 ctagtaagacgctgtgttttctgttttccttttgataacgtttttgttggaaacactttggcgatacggtagtaggttatggttaagctagcatacattaagtga

16171 ttgtcttttccatggaatgcagtgttagcactatgccagacatataagtattgctttcaacaatcattggggagattgtgtagataaataatatcacgtgtaaca

16276 tacaaatgttaaaatttcagctctttatgtaataaaatgataatacttgctaaaattgtagtttgatttttttttcttcttagcttaaacctcttcccccatgta

16381 aggtaacgtatttcaattatgtgaaaaatatacacattgttggtagacaattccaagtttgaatatattcaataggggtgtcacattacgcctggagagtgtcac

16486 aacacactgcaatacagaaggaatgtattgtgatactaaaccagacataacaccagtagctgtatatagactcattgatatgtactctcaacaattttttgggca

16591 gatcaatactctggattaaagaaatagaaaaagtcatttaaagtaatttgatgcctgaataattagcatacatgttattatctcatttcaaatgaagagacatat

16696 tgtttatattcaattcagtgtatttcaaatgcagtacagcttataggagttttacaacataataacagacatgtgccaaattaatatatcttccatttcaatatt

16801 tagactgatgaaagcctgtatttacaaataattttcttatatttatcagaccagaattacttaaataatattgcaatttaaagtatcttctaagtgcttgtaaat

16906 ataacactttgaggaagtggtaaaatgcattaaagtgttttgtacgaaacaatcttttactcttcgtgcgagttcagatataaacaagtcttcattaaatatatt

17011 ctgagcataccatatctcagcaaaaccttaagtaaatggaagctcttttacatttgttacccaattaacattacccgacatacagtcatcaaatgatgctttgta

17116 acatgaagttaagatacaattatttgatccgaggagcttcagccaatattttacaattttacttattcgtgtcacatacatagctaatctttgtaattcaaaata

17221 taactaagcatgtgatgaagtcttgcatacagatattttttcttacaaaaatcagtttgaactttttcgaccattttgccatgtactgcttatggttcttccaca

17326 tcctcacaatgtgaaaggctcatcctcgcaaaagaatagagaagtataataagaggccctgtatcactcatttggatatttaaacaatcatggcaatatttctca

17431 ttgaaagtagtaaatgttttcccatttcagccttctctctggttccaagaatgcttcatggacaattttgctgagatccctatgcatgtctggttacaagggttg

17536 ggatcttgattgtttctaagtaatgacctgagaataagtcaccagggttggggctagatcctggtaacaggcttgagttcttttcttcatgccaagcagacatat

17641 gaatggttgtataatgggtggtatctccactgtttccaagtaaagatcctaacaaagaaaagtgttttcactactgatcttcaaaatgtgtcaaagtatagtggt

17746 tctagacataatgattcttcagttattgtgtcatttcatgtatttaacaggttttatgcaagcgaccttgaaagtaggtcaaggccattgatttttttgaatttt

17851 ttttcacattacaataacatatacatttcattatacataatgaattccactttcacccatactttcatactaattaatattgctctttttgtaagtggcatttta

17956 ttttttgtaatgttatattagaaaggaaggaaaatcagataagagaaataagaaaacaaagaaatagaagaaataaagacaggtaaatggacagaaagaaagaca

18061 gacaagagaagtaccagtattaaacaaaccaatgtaaaggatagaattctggtaatatataagtgtcagtagctaagtaattaaagataaaaagtttcaatattg

18166 tctcttttttttttcaaataaatttaaataagatcaaacatataatcggcaaaatttaggtttagataatatcagggcaataacttttgcaaaaaattttgaatc

18271 aaaattcttttcaaggaaacacaacaacatatcatgatcacttagtctgcttagtttcaaagaaatccgtcaaaaaatgtaggagatctccggacaaaaaatcaa

18376 atactgaaatgaacaaaggcaataacttttgcaaaaattgtccaatcaaaattcttttcagggagacaacttcatatcatgattataatgtataccaagtttcaa

18481 agagatcagtcgaaaaatgtaggaggagattgcccgacaaaccaaagtttcgcatctattaagaacttaacatgttgtaaaagattttgtacttcgtcacattcc

18586 cataatacatgctctagtgtgcctatttctatgttacacagttcacagaaagaactgtctactattcctgccttaaacaaatatgcattagttgtcaaaatgtgc

18691 tgattaattctgtactgaaatcactgtaatttggtatttttcgtaattttaaatgacattttaaatatttttttccaaatttcatcatcgaattataatatatca

18796 ttccattttttttagctgttgaaatagtatcatttttacaaaataagctttaaaaatctcatatcccctttttatttttcaaaatcatttctatattaattggta

18901 tgaatggatagttaacacctacttttgacctatcgcctcgtataatatactcgtaaaaagtttgtattaatatcataaatctgtttgaattcttgaaatgtgtaa

19006 aatgcataggtatttctatctctgattagatcatatgattgatatgatttaattttataaaactccatccaagacacatacctcagtcctgtcatatatcataca

19111 cctatagaacaaaagaaccttttaatgcattgtaccagctatcaggagtgatgtcctcaataggagaccaaagacaaggcaatctcatttcacagacttgaatga

19216 aaggtggtttcaggtataagggttttatgtttgctggaaaaataacggccgttagcagttgatattgcattactccgagtatccccgttatattctgttgatatt

19321 acattactctgagtatcccagttatattctgttgatattgtattactccgagtatctccgttatattttgatgatattgcattactccgagtatccccgttaaat

19426 tctgttgatattgcattactccgggtatccccgttatattctgttgatattgctttactctgagtatccccgttatattctgttgatattgcattactccgagta

19531 tccccgttatattctgttgatattgcattactctgagtatccccgttatattctgttgatattgcattactcgagtatccccgttatattctgttgatattgcat

19636 tactccgagtatccccgttatattctgttgatattgcattactctgagtatccccgttatattctgttgatattgcattactccgagtatccccgttatattctg

19741 ttgatattgcattactcgagtatccccgttatattctgttgatattgtattactcgagtatccccgttatattctgttgatattgcattactagagtatccccgt

19846 tatattctgttgatacattgtactacattactagagtatccccgttatattctgttgatacattgtactacattactcggagtatccccgttatattctgttgat

19951 acattgtactgcattactagagtatccccgttatattctgttgatacattgtactacattactagagtatccccgttatattctgttgatacattgtactacatt

20056 actcgagtatccccgttatattctgttgatacattgtactacatt

**PyRho**

1 gctaaaatcacaccacaccaaagacagcagtgtcatgctaaaatcacaccacaccaaagacagcagtgtcatgctaaaatcacaccacaccaaagacagcagtgt

106 catgctaaaatcacaccataccaaagacagcagtgacaggctaaaatcacaccacaccacatggcacaagacagcagtgatacgcttaaatgacaccacaacaaa

211 gccagcagtggcaggctttgctcgagataacttaataaataaaaataccatataaatagatggagaatttataatttatcaacaaatatttattgcagcttgtca

316 cgtgtccatataggggcatcgtcacgttatataggttcgccagtgtcacagaataaatttgtttttcgaggaggaggaggaggaggtggaggcagttacattata

421 gactaaaccttccgaaatctaggtttgttatgttgatgacaaaagttggacaagaatggcatgaaagattacactgacctttagagtttgagtgaccagcacatc

526 aagttcactgtggagtcggaggatgagggtgatgaattaacatttcatttctggatatatatgtccacataaatgatggcactcccaacaaatttacagtgtaca

631 gaaatcaaatagtatataaacgatctgttgtaagaatctttttccaccaggctgagacactagtaattgaggaaaaggtcaaagcgaagtagttggaacacctca

736 gaccagtgttgcatgcatcaagtactgttcctgggtttttgttaaactccatgatggaaaagcctccacgtccaacaaaaggaaagagtggccaagggaatgtga

841 ccagctttgctcttacgtgtgtccgaggcgctttaaatagacaaactcacaaaatggttttcaaaaccagggaaacaacacgtatcataagccctcttggatcac

946 agtaagtccgaactcaagaccacacacctatggagacgtgtagggtatcgtacaaggtcaagtataatgactttagcgaagattacatcggagagacagcaagaa

1051 cgttcgaaacctgaggttcaaggaaaatgactacgccaggagaacttccatcacattaggccaaactgctaaatactggccatacaagtagacatggacaaaatg

1156 gtgacaagagagggaaacaccctacaacaacaacaaaaacacaagaaaacaccgatcgaagtgtcaacgaaaccataattcaaactcaatatcaataaaactatg

1261 gtaaaacataaacataggaaacatttacatgaaaacactttaaaaaaagtacaaggagggtacgatcaaacgtgttgagttggtaaacgtctgctgcttcatcga

1366 cgacacctttcttgtaaattcaggtcatttcaaagtaatgtcatgtcctcatagcgagaacgggggtagtgacaccaaagaaatagcatatatagtttccgatga

1471 gagcaagatggcgacgaaaaaattgtccatagtcttcagctacctgcatatatggaaaccagtttgtcaaaaataatgcgaatggccattaatgtcccgttccaa

1576 gtcccaaccctttacgaagaccgggtttatgggcttctatcagccttttaggattttattttcacgtgacaattgccaatgttgatcgtgtgaaaagtacttgag

1681 aaaatacaccgggagagttcagaatattttctttctagtatcctagaacctgccatgtaatttaattattaccagtatgaacttaaccatcctcgatagttgaga

1786 ggttacgctcacttttgctttctgcacccctggtctgcacccctggaatacgtcatagcaaaattgaaggcacatggctagctattttgttggtctcgggtaaag

1891 aaaaaactgatcaatccctaggctaatacctgttctttgaagattacagaggggtgacacccaaagatcgaactagtctgatcttgtgtcatccccagtgtggca

1996 cacactgagccttcaattttgctgacatattccaggagcctggagtatcaagaatgattccaggagcctggagtatcaaggatgattccaggggcctggagtatc

2101 aaggatgattccaggggcctggagtatcaagaatgattccaggagcctggagtatcaaggatgatttcaggggcctggagtatcaaggatgatttcaggggcctg

2206 gagtatcaaggatgattccagggacctggagtaacaaggattattccagggacctggagtaacaaggattattccagggacctggagtaacaaggatgattccag

2311 gggcctggagtaacaaggatgattccaggggcctggagtaacaagggtggttccaggggcctggaatatcaaggatgattgcaggggcctggagtaacaagggtg

2416 attccaggggcctggaatatcaaggatgatttcaggggcctggagtatcaaggatgattccagaagcctggagtgtcaaggatgattccaggggcatggagtgtc

2521 aaggatgattacagggacctggagtcacaaggatgattccagtggcctggagtgttaaggatgattccagagacctggagtaacaaggatgattacagggacctg

2626 gagtcacaaggatgattccagagacctggagtaacaagggagattccaggggcctgggatttctaggatgattccagggtcctggagtgtcaagtatgattccag

2731 agacctggagtaacaagggtggttccaggggcctgggatttctaggatgattccagggtcctggagtgtcaaggatgatcccagggacctggagtaacaagggtg

2836 gttccaggggcctggggtgtcagggatgattccaggggcccggagtgtcaaggatgaatccagggacctggagcgtcacggatgattccaggggcccggagtgtc

2941 aaggatgcttccaggggccttgagtgtcaaggatgattacagggacctggagtggcaaggatgattccagggacttggagtgtcaaggatgagtccagggacctg

3046 gagcgtcacggatgattccaggggcccggagtgtcaaggatgatcccagggacctggagtggcaaggatgattccaggcgcctggagtatcaagaatgattccag

3151 gagcctggagtatcaagaatgattccaggagcctggagtatcaaggatgaatccaggggcctggagaatcaaggatgattccacgggcctggagtgtcacggatg

3256 attccaggggttacaccatcttctatactccaggggttacgctcacttacgccctctgcatccctggagtgtcaaggatgatctcaggggcctggagtaacatgg

3361 gtgattccaggggactggagtgtcaaggaagattccagggacctggtgtgtcaagtatgattccagagacctggagtaacaagggtgattgcaggggcctggagt

3466 aacaagggtggttccaggggcctggggtgtcaaggatgattcgagggacctggagtgtcaaggatgtctccaggggcctggagcaacacgggtgcttccaggggc

3571 ctggaatataaagaatgatttcaggggcctggagtgtcacggatgattccaggggcccggagtgtcaaggatgagtccagggacctggagtaacaagggtggttc

3676 caggggcctgggatttctaggatgattccagggccttggagtgtcaaggatgaatccaggggcctggagtattaaggatgattccagagacctggagtaacaagg

3781 atgatgccaggggcctggagtgtccaggatgattccagggaccttgagtgtcaaggatgatcccagggacctggagtaaccaggatgattccagggccctggagt

3886 gtcaaatatgattccaggggcccggagtgtcaaggatgattccagggacctggagtgtcaaatatgattccagggacctggagtgtcaaggatgattccaggggc

3991 ctggagggtcaaggatgatttcaggggcctggagtatgaaggatgattccaggtgcctggagtgtcaagtatgattccagggacctggagtaacaagggtgattc

4096 caggggctttgagtggcaaggatgattcaaggggcctggagtgtccaggatgattccaggggcctggagtgtcaaggatgattccaggggctttgagtgtcaagg

4201 atgattcaaggggcctggagtatcaaggatgattccaggggcctggagtgtcaaggatgattccaggggcctggagtatcaaggatgattccaggggcctggagt

4306 ggcaaggatgattccaggggcctggagtatcaagaatgattccaggggcctggagtgtcaaggatgatgccaggggcctggagtgtcaaggatgatgccaggggc

4411 ctggagtgtcaaggatgatgccaggggcctggagtgtcaaggatgattccaggggactggagtctcaagaatgactccaggggcctggagtgtcaaggatgattc

4516 caggggcctggagtatcaagaatgactccaggggcctggagtgtcaaggatgatgccagggccctggagtgtcaaggatgattccaggggcctggagtgtcaagg

4621 atgattccaggggcctggagtatcaaggatgattccaggggcctggagtatcaaggatgattccaggggcctggagtatcaaggatgattccaggggcctggagt

4726 atcaaggatgattccaggggcctggagtgtcaaggatgattccaggggcctggagtgtcaaggatgattccagggacctgttgtgtcaaggatgattccaggggc

4831 ctggagtatcaaggatgattccaggggcctggagtaacaaggatgtctccaggggtgcagagcgaaagtggacgtaaccactgaagtttcgaggatgaaacataa

4936 cgggatcacgtatcatgaatttccaaatgaaattaaaaatgaaaggtatcacataattataaaatgtaaacatatgcaggatatgagaactagtagcattattac

5041 ccaccgagaaagaaataatcatatgtttaataagaacgtgtaaaaacatgggaaaacgtagagtcgattatcttccgtctcggaagtcgtgatggaaacagattt

5146 tcgagagaatctgtattaggaattatgctgacctacaataggaaccttcttgcacggcattgtgacaaaagtggacaaaaaatctgccatctagatttatttatt

5251 gaaaatatttcatggtaataaaggattgcttacctgtttagaatttgatttatatctttaatactccatgatgagaccttttgatcgtatatataacgacctgta

5356 cagaccctgggattggtgagtacagattctgaatgttccaggaacgttgaaactgcaacttaagatcatccgaggtgggttttttcagcattcttttttacatta

5461 ttattcatcaaaacccaactattatcaatgataatgaaatttaagtcaggaaactgggcaggtcatggcaaaatgggaattccatgttctgatcggccttccaca

5566 gttttgtttgataggtggcatgaggggtggtgttctcatcttgaaacaagtagggtttgtctgaatttttgtttttaacaaccacaggctataaattggaatcta

5671 gaggctgaatatacatttcactgttcaagtttccgtctgcaaacattgaagtctcccgattgttttacaacttcagaccattggcttgaacagactgcatggcag

5776 atccagaggggttttttggtcggtttctatgtacaatcatcatgcagcaattatacagtgcttggctcagtactttcaggccgacaacaaattattttcaaatat

5881 aaatctagaacattttgagatggtgttttaatcaaagtgttgtaagatggctaaatattagacacaagcaatactggttaaaaaaaattctaaactatacacatt

5986 gcatgaaaatggaaaatgaattatcaaatctgtccgagcgtcatttccataaacaaccattgggtcgtaagtatacatggtatataccaaagtacacttgtaaaa

6091 aaataaaaaaaatacttgcattgacggctaagtcgacgataaaactgcaagtacagtagagatattggtattttcaatatgtatgcaatgcatttacactaaatc

6196 acaaccattaggcctcagattaatgagtttagttcacgagatgtccgtcgcacagaccaaatttttaagattcgttgtgttacaagatatttcataactgttttg

6301 attaataatataacttttcccttcttataccacttcttgtaaaaatgtttgaaggcttaaactgtgttgaagactatggtggcgttatttatagtcacatcttat

6406 gtattatgacgtcatatctaaaaagtgattttctctcttttaggttatatttttaagttttcttctccatttatttcgtgtgaatgtcccagacacccaacctgt

6511 gagctaaactgctaagatccccaacattgtaaatatttgctaaagtatgaagaaattaattttaatcttcacagtggtcacaaccatgtacagaataaagttata

6616 aagctcaataactcttaaatttcatggaatggttcaataaaaaaagtcaaacccaaaggcatcagggagaaatagaggctttaaaatattaaattatattgatgc

6721 tgagttttcaagaatgaaaactgattaaattattgatgtcattaataccaccagcgaagtacaacagataagaatcagaataatacatgtgtacgttaaagatga

6826 aaaattaagttacaaatagtttttagacctaattaagctatatctttggttataatttagatgcatgtaacttgatatgtctctggagaatcataggaagattgt

6931 tcgaaattaagctgaagtatatacacaatacatctccagcacacacgcaggcatacacacatagggagatattagccaggtggcataggactagtagtttgataa

7036 ggcagctcctgcgaattcctcaaaaattgtatatcctaattgtattaattgtatatctttgtactactgtaaagcgctttagagtggcttatgttagctagatag

7141 gacgctatatcaaacattatatattattattattattaagtatatatatatatgtactttcttgtgtaaacatgcaactatgacctcacaattggtaatctagct

7246 tgatctagtgcaatttatattaattagcctaattgctggggaaaaataatgttgttgaccaggtgtatcggagacgaattcaaagtatttgagtacatatgacaa

7351 tatgtgtcaaatccatgcaattacattatatttattttactcttaccttttcagtccccagcatgcagggaccatttaacatttatttaacaattcgttacgtta

7456 atttctaagttattttacgttgttgttgaaatattgtgttcattatttctcaatattgaatataataaatctactttaaggctgtcttgttttatacaaaacgaa

7561 tggcagatatatttaaatgttagtcattattcagagtacgcgtattatcaactgtaaaaggtagcattctatttatagtttctggctgtggcaaagaatcggacc

7666 aatcagattttagctcactaagtttctggttatttttagacaagcaggccctagatatataaacaagagtcatggcctacaatacaaatattcctgagagtaatg

7771 tcattactgtgttgctcaagaaacaacggtaagtgtttttaaagctatttttgttttcacgacgttgtttacaaaaaaacttattttaacaaatatgcatcttta

7876 aaggctctttttgagaaatataaatgcataaatttgtacatcggctcaaaatgaacatccacttgatcttttaaactaaataaattttatttgtataaaaattca

7981 gacaaaataggctctaacaaaataaacccggaaagagcagatttcgtctgctgtttctgacgtcgcttgaacgtttgaatcgtatagatatatgaatcaaacact

8086 aaaataacacagtcaaatacttcccatgatgcttcagacaaacatccgactcccacaattcctgagcaattgattcagccctctaaggacaattgaggtgtggtc

8191 ggtgcttgaatgtatcagtagatgaacgtttttacttagttgaaaccggtaaattacatacggtataaggtaagaaattgtctacaaaacttctgttatgtatac

8296 tgaaagaaagatgatcgataaaaatctttgaaacgatgaatattattagaattgtggatataattattcacttccctatttggcaatgacctcagcatgctcatt

8401 gtcattacgatttgacggcgaattcggttataaaatgcataatgcgttgccagatcttgtcctgcaataactaagctgtcctaatgagtaacgatcaaatgtgta

8506 tgtgtggtgaaatatgtatagttcgtatgtttttgctgtgggtttgatctttgaattccatattcgccatcttgatgacatcatcgttgcatctgctgtctacta

8611 gaatgtctggaaaaccctctttttggatcttattcgatgtttaacggttgtctaatcgaacgtttgttttggtatgttttgtttggcacagtataggttgatgta

8716 ttctatgaaaatgttttgaattctgctgaagatgggttcggcttcagtgaaagaggaagcctgctccacacgtctagaatctaaccacatactgtttttgtttgg

8821 tagccccagatgaatattcgataaaaccgttcacagtgatttttaaggtctatttgttggagccttacattcccctgcataatgtttagctggtttccttcccga

8926 aatctggacaaacaaaaaataattgtggatgatattttctaaaatcaacacaggcacatgcccctgtagaaaagaggaagacaaatcactcgcgtatagtccatg

9031 atttactaattggataactagttaaagtccagagttttgtttgtgttgagtgattagtatcggacttgagatttatcgtgttgaacaaacaggcaccaaccagaa

9136 aagacttattgaggaaaagataattcactgttgttgataaaaatctttgttgtttagactttatttttaatattttaaatactgattatacataatatcaggtcc

9241 atttatataattaacagaatgggatcaaccaagcattagttggtgtcccacaacactaatgtagtcgttaaatcagctgtattcctcattaaaagtaacttaata

9346 atttgagcagacactgtattaaagacgaaagttggaccaaccgtttgtcgggacaaacttgtatttacacttttgataccaccctgcatcacatgctgttactta

1 M A A I R K K L V I V G D G A C G K T C L L I V F

9451 ttgtgtatttcagattttgatgttagagacATGGCTGCTATAAGAAAGAAGCTGGTCATTGTAGGAGATGGTGCCTGTGGTAAAACATGTCTCCTGATTGTGTTC

26 S K D Q F P E V Y V P T V F E N Y V A D I E V D G K Q

9556 AGCAAGGACCAGTTCCCTGAAGTCTATGTGCCAACAGTATTTGAAAATTATGTAGCAGATATAGAAGTAGATGGAAAACAGgtttgtatatttcaaaaagtcctg

9661 cagagtaggtctattatataacactgtgttttcgacacgggaacattgtgctggttacagatatggatgattgcttcagattggtattcaaggaaagtcggggct
[truncated: 201,645 more chars]
